# Supplementary figures and images for: SPIN1 facilitates chemoresistance and HR repair by promoting Tip60 binding to H3K9me3 (part 1 of 2)
Source: EMBO Rep. 2024 Aug 1;25(9):15. doi: 10.1038/s44319-024-00219-1 (PMC11387427; doi:10.1038/s44319-024-00219-1)

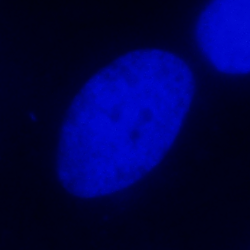

Supplement: Supplementary file 3 — Source data Fig. 1 [file 44319_2024_219_MOESM3_ESM.zip › Figure1/1A/DAPI.tif]

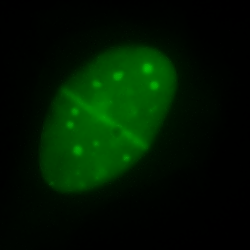

Supplement: Supplementary file 3 — Source data Fig. 1 [file 44319_2024_219_MOESM3_ESM.zip › Figure1/1A/SPIN1.tif]

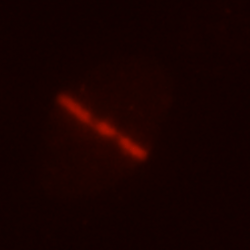

Supplement: Supplementary file 3 — Source data Fig. 1 [file 44319_2024_219_MOESM3_ESM.zip › Figure1/1A/γH2AX.tif]

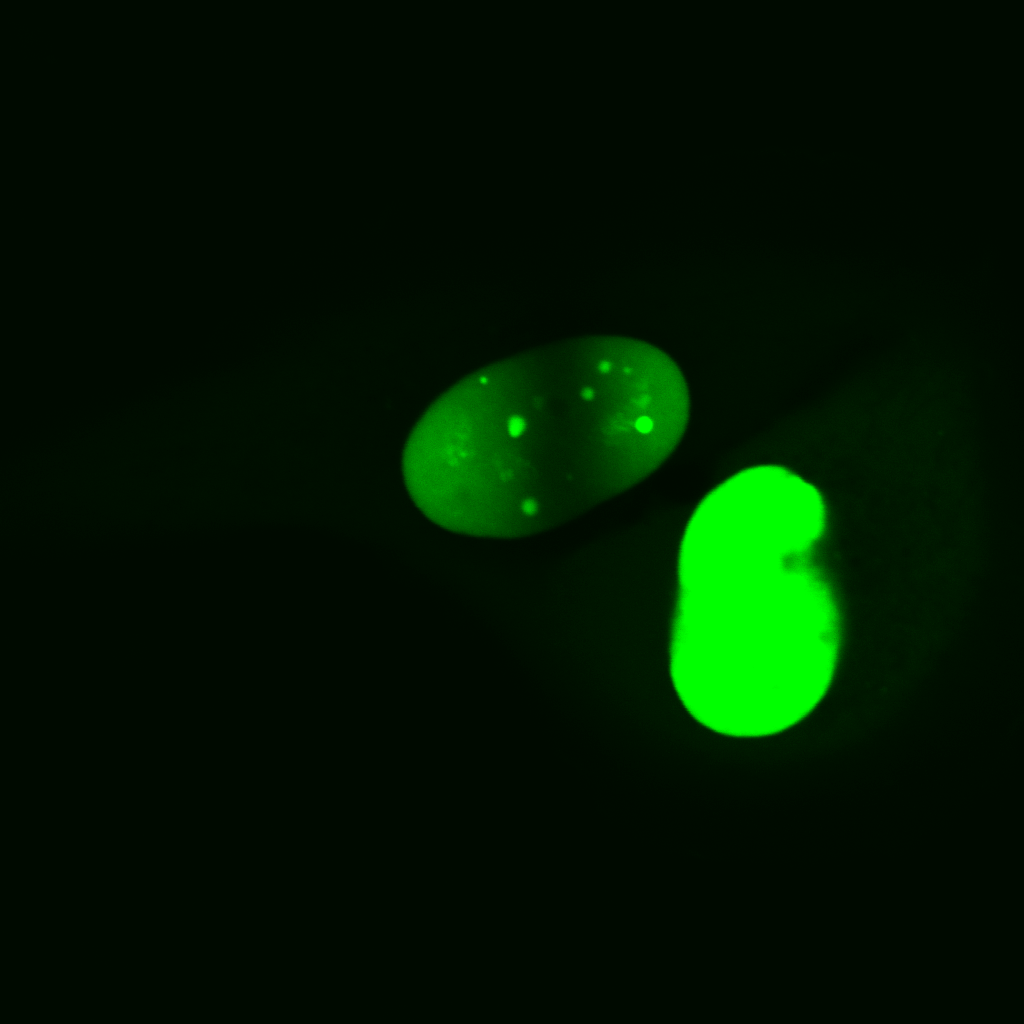

Supplement: Supplementary file 3 — Source data Fig. 1 [file 44319_2024_219_MOESM3_ESM.zip › Figure1/1B/0 sec.tif]

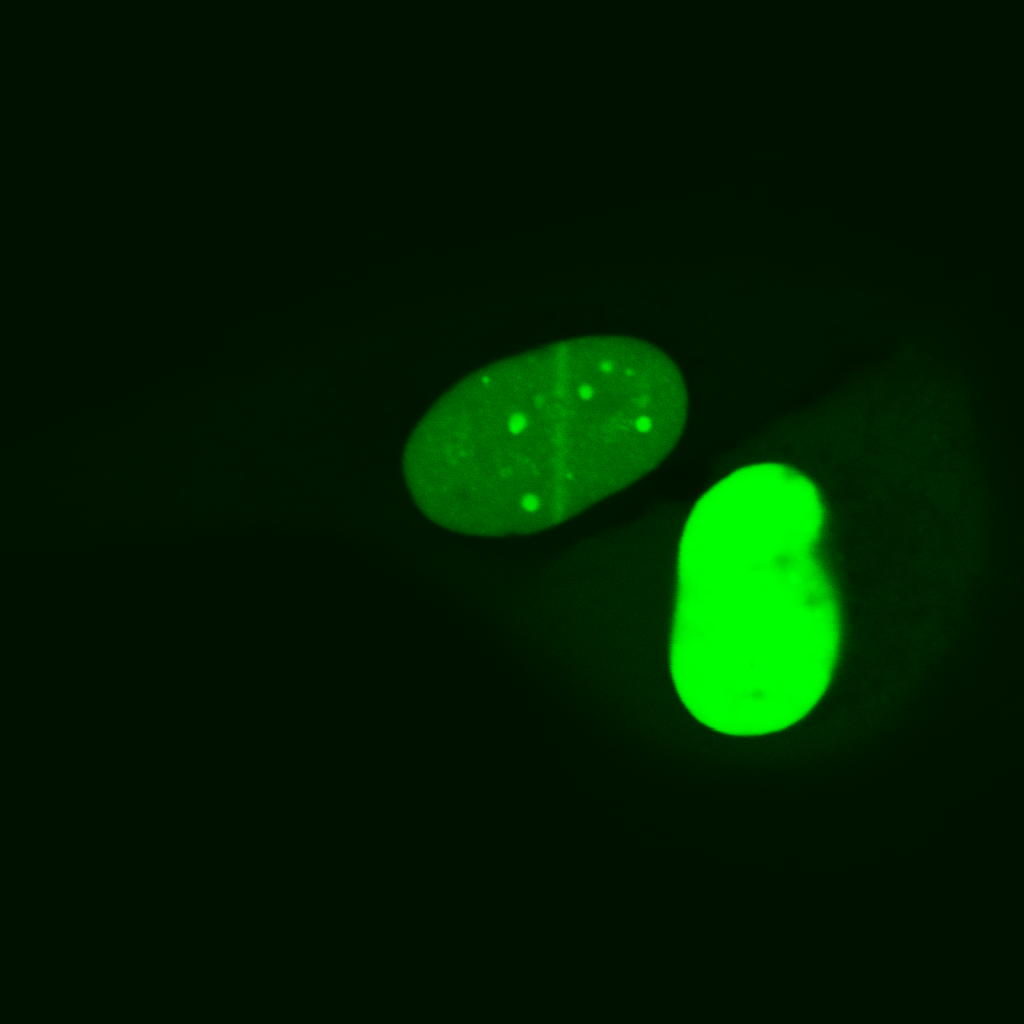

Supplement: Supplementary file 3 — Source data Fig. 1 [file 44319_2024_219_MOESM3_ESM.zip › Figure1/1B/1 min.tif]

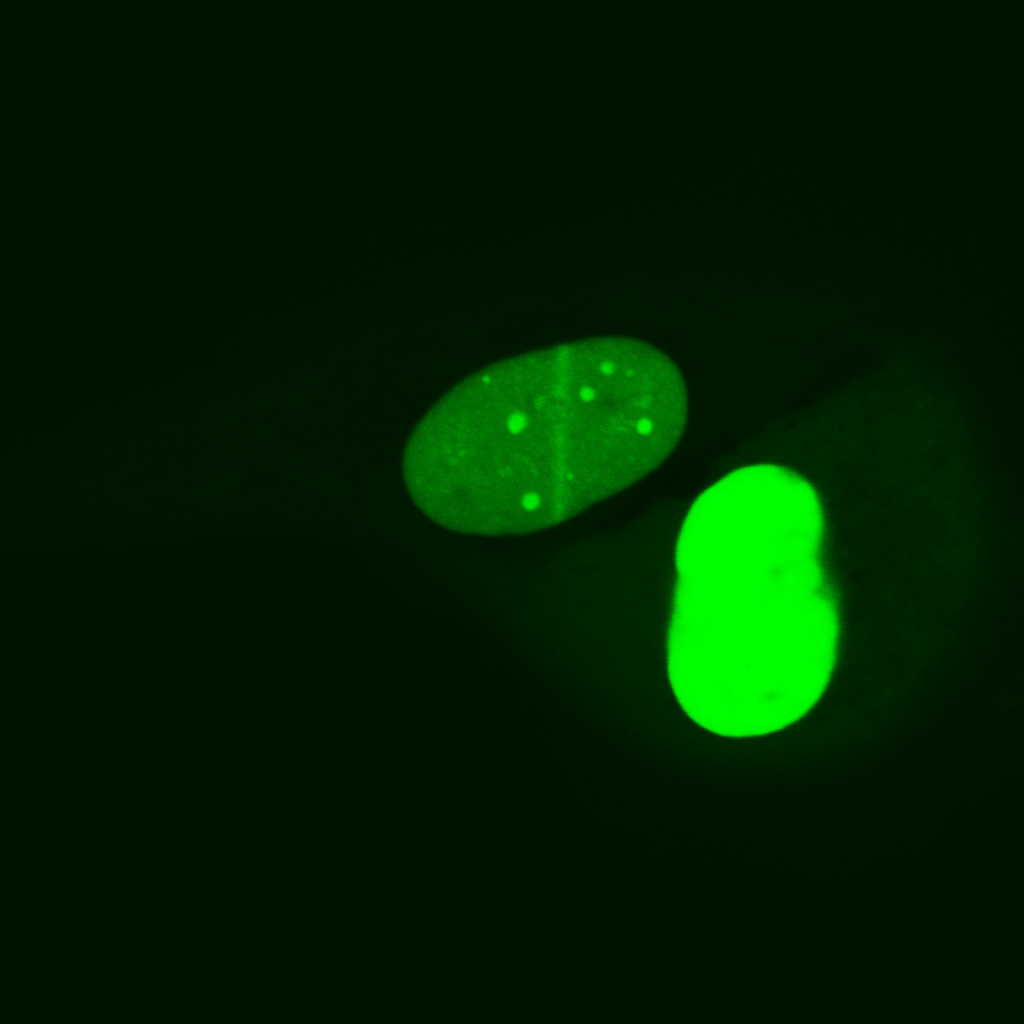

Supplement: Supplementary file 3 — Source data Fig. 1 [file 44319_2024_219_MOESM3_ESM.zip › Figure1/1B/10 min.tif]

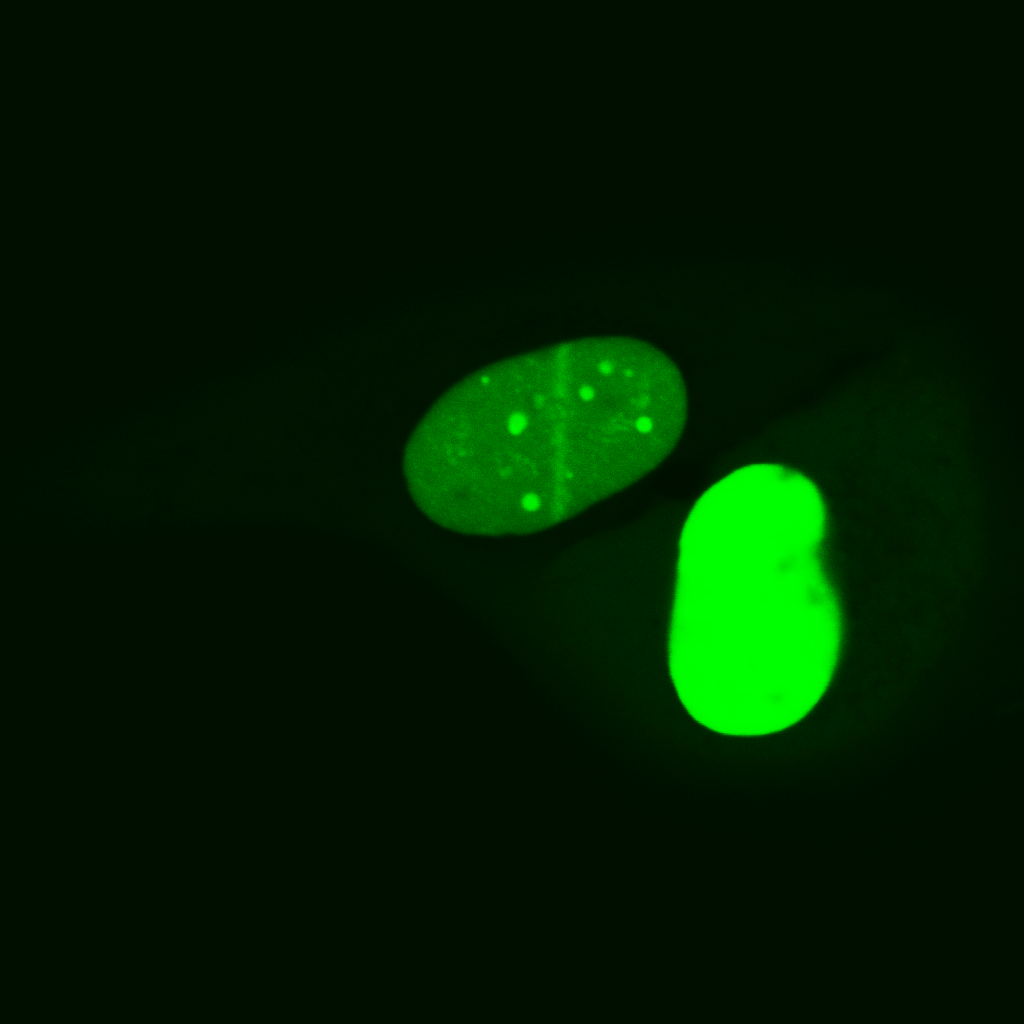

Supplement: Supplementary file 3 — Source data Fig. 1 [file 44319_2024_219_MOESM3_ESM.zip › Figure1/1B/3 min.tif]

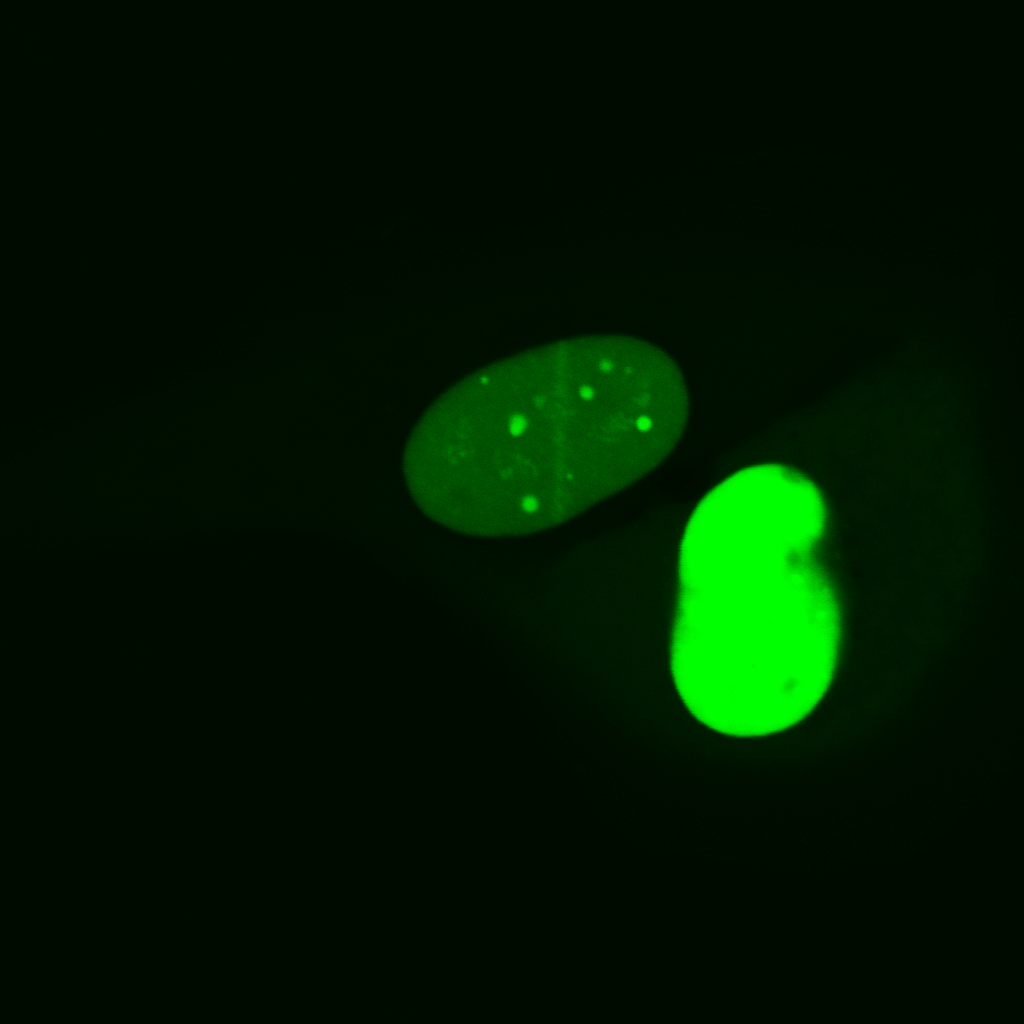

Supplement: Supplementary file 3 — Source data Fig. 1 [file 44319_2024_219_MOESM3_ESM.zip › Figure1/1B/30 sec.tif]

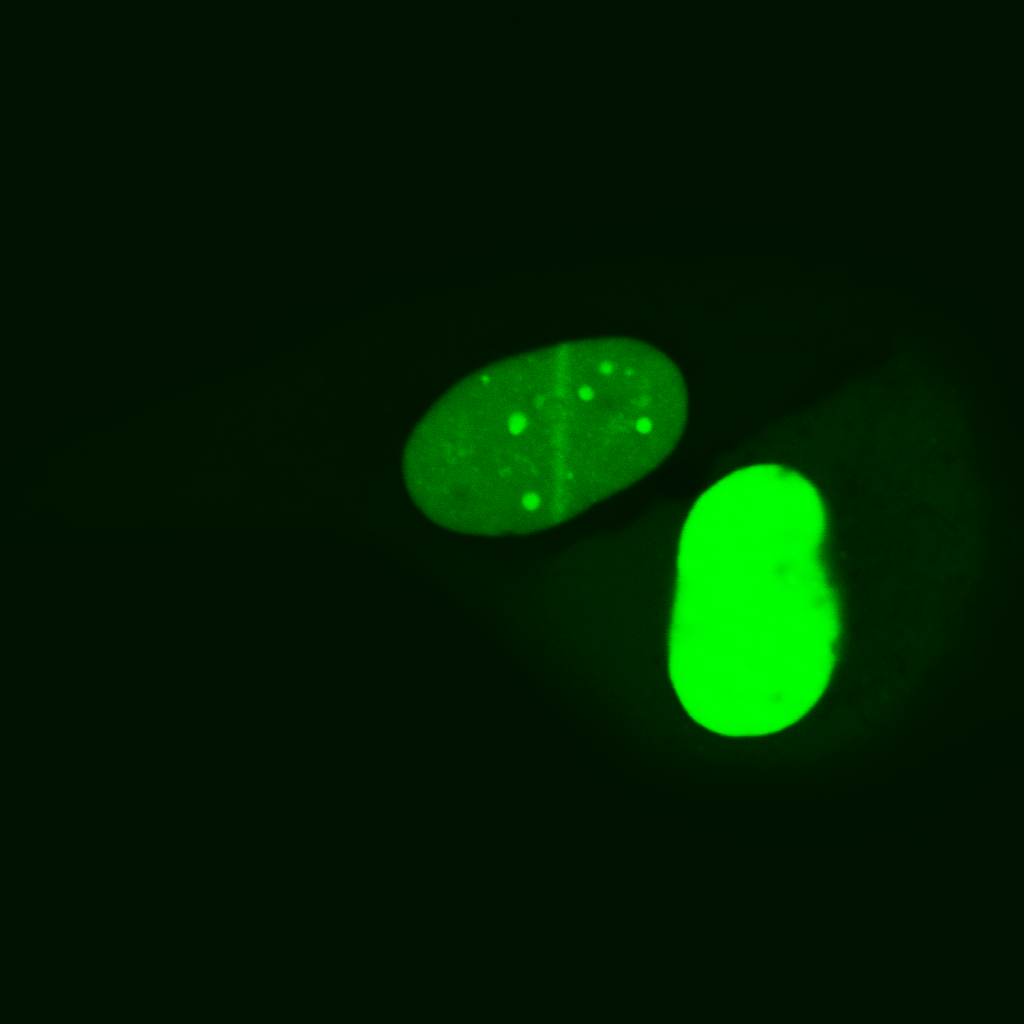

Supplement: Supplementary file 3 — Source data Fig. 1 [file 44319_2024_219_MOESM3_ESM.zip › Figure1/1B/5 min.tif]

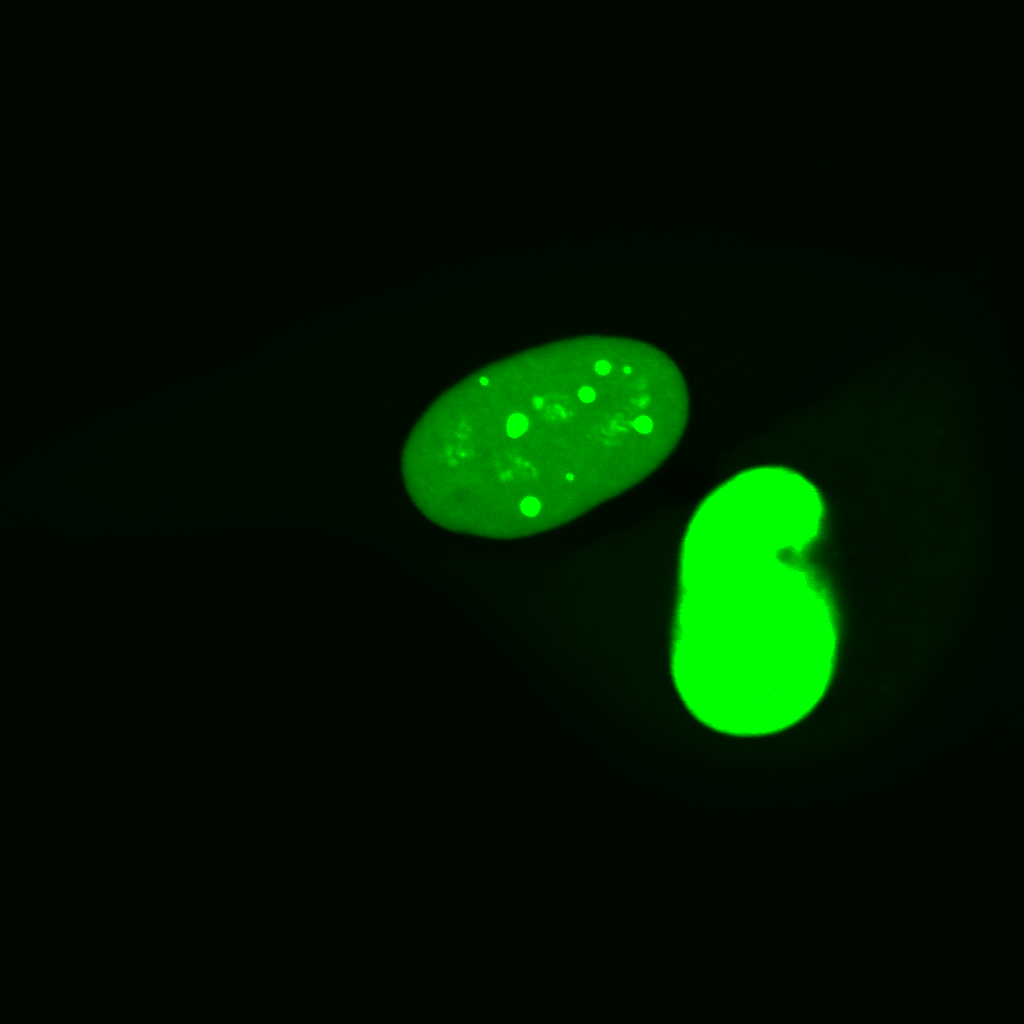

Supplement: Supplementary file 3 — Source data Fig. 1 [file 44319_2024_219_MOESM3_ESM.zip › Figure1/1B/Before.tif]

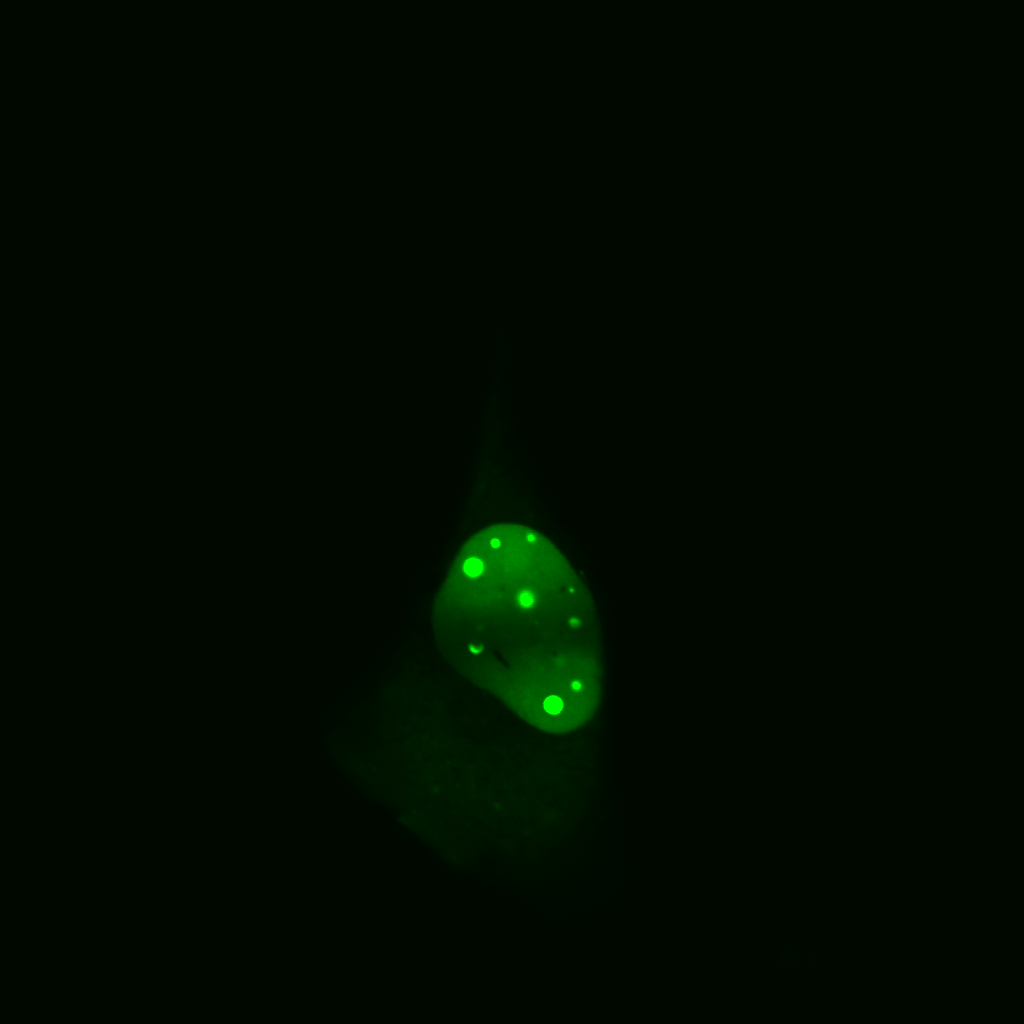

Supplement: Supplementary file 3 — Source data Fig. 1 [file 44319_2024_219_MOESM3_ESM.zip › Figure1/1C/WT/WT-0 sec.tif]

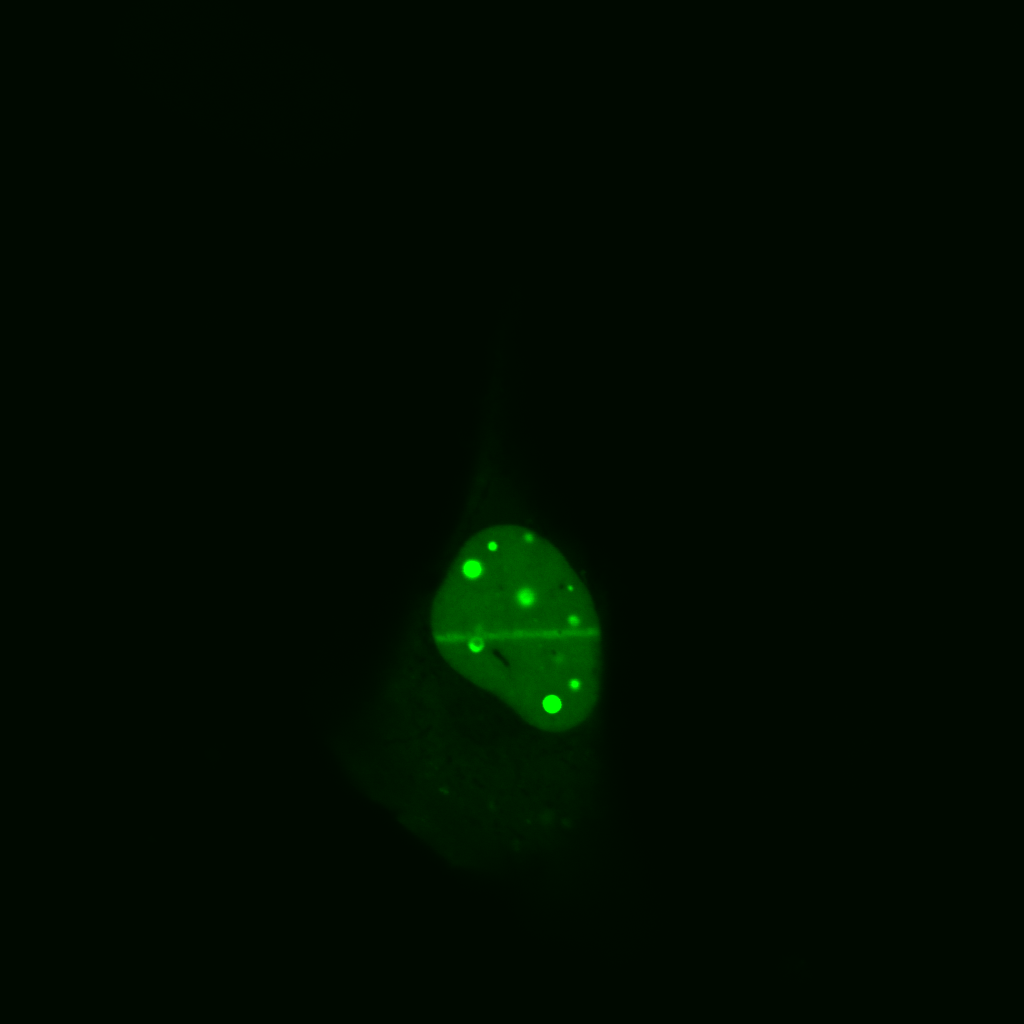

Supplement: Supplementary file 3 — Source data Fig. 1 [file 44319_2024_219_MOESM3_ESM.zip › Figure1/1C/WT/WT-1 min.tif]

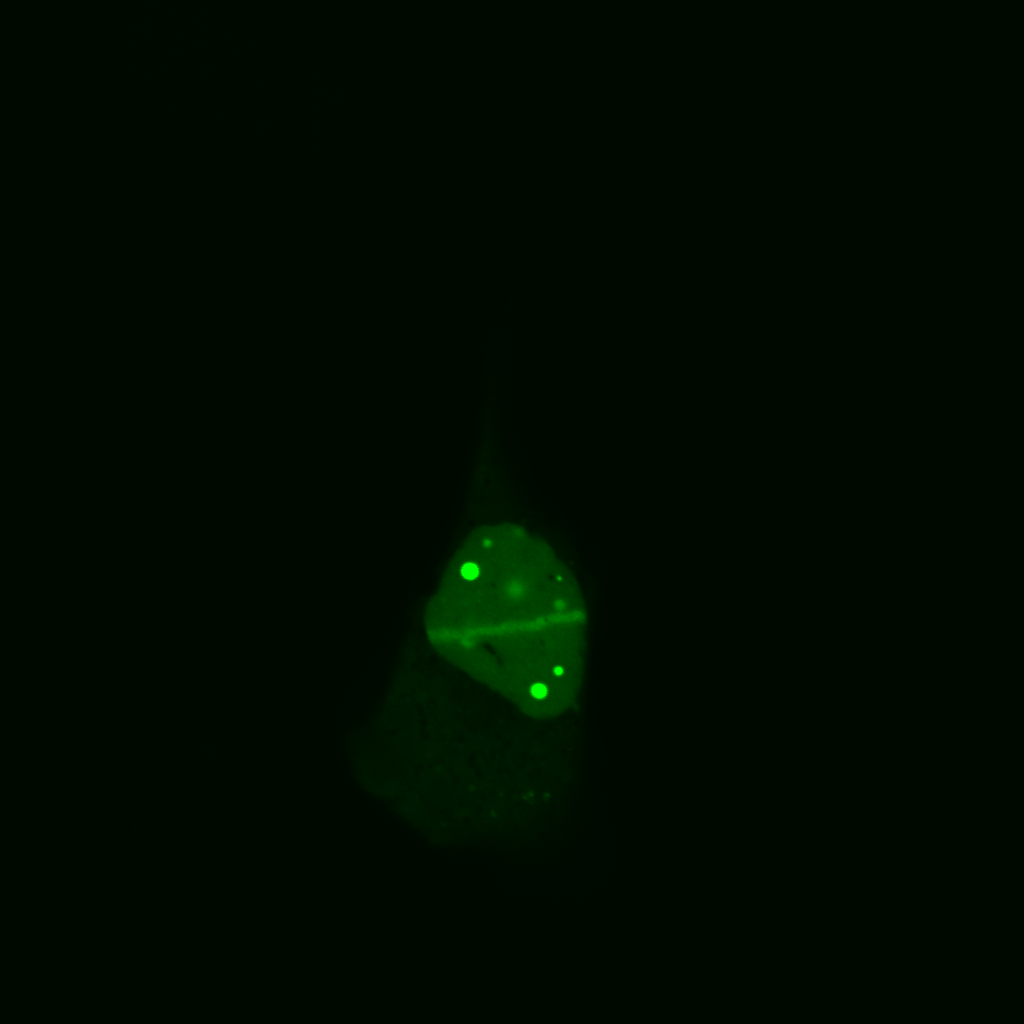

Supplement: Supplementary file 3 — Source data Fig. 1 [file 44319_2024_219_MOESM3_ESM.zip › Figure1/1C/WT/WT-10 min.tif]

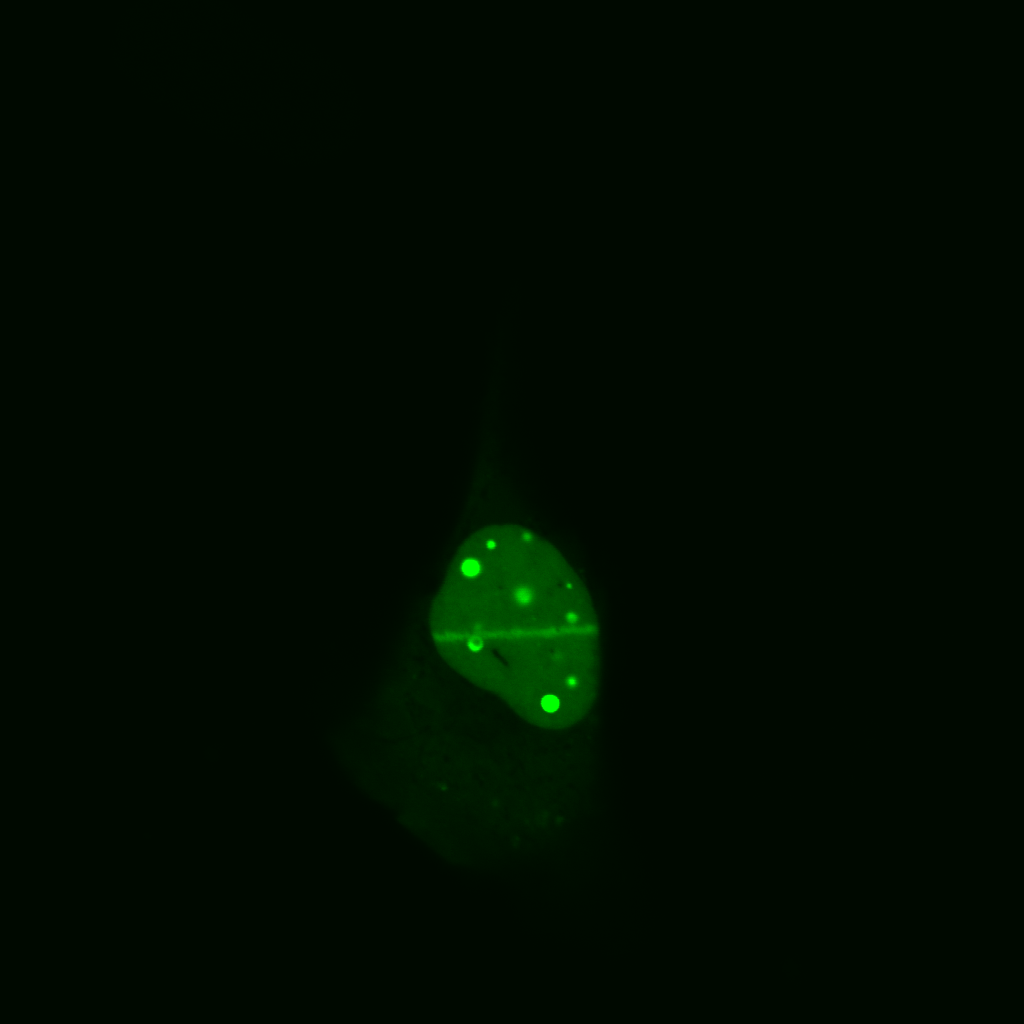

Supplement: Supplementary file 3 — Source data Fig. 1 [file 44319_2024_219_MOESM3_ESM.zip › Figure1/1C/WT/WT-3 min.tif]

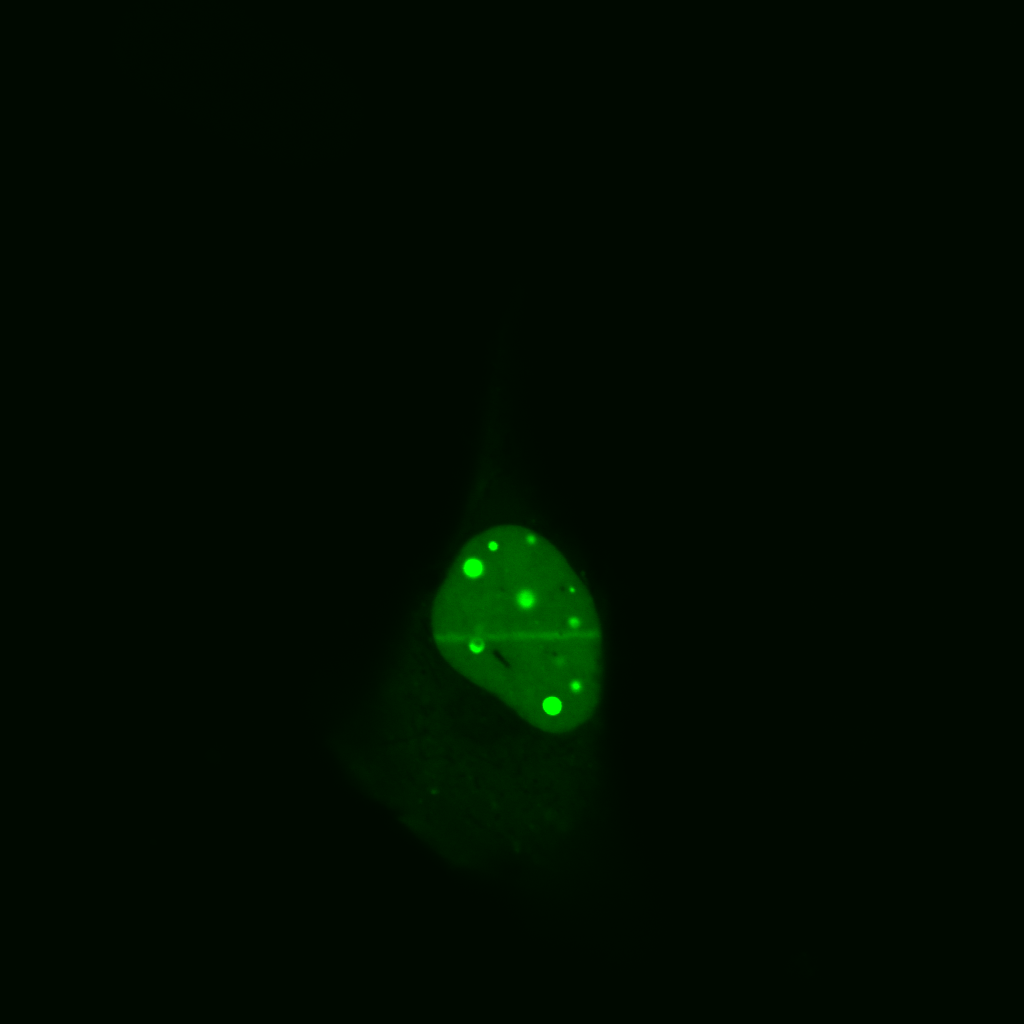

Supplement: Supplementary file 3 — Source data Fig. 1 [file 44319_2024_219_MOESM3_ESM.zip › Figure1/1C/WT/WT-30 sec.tif]

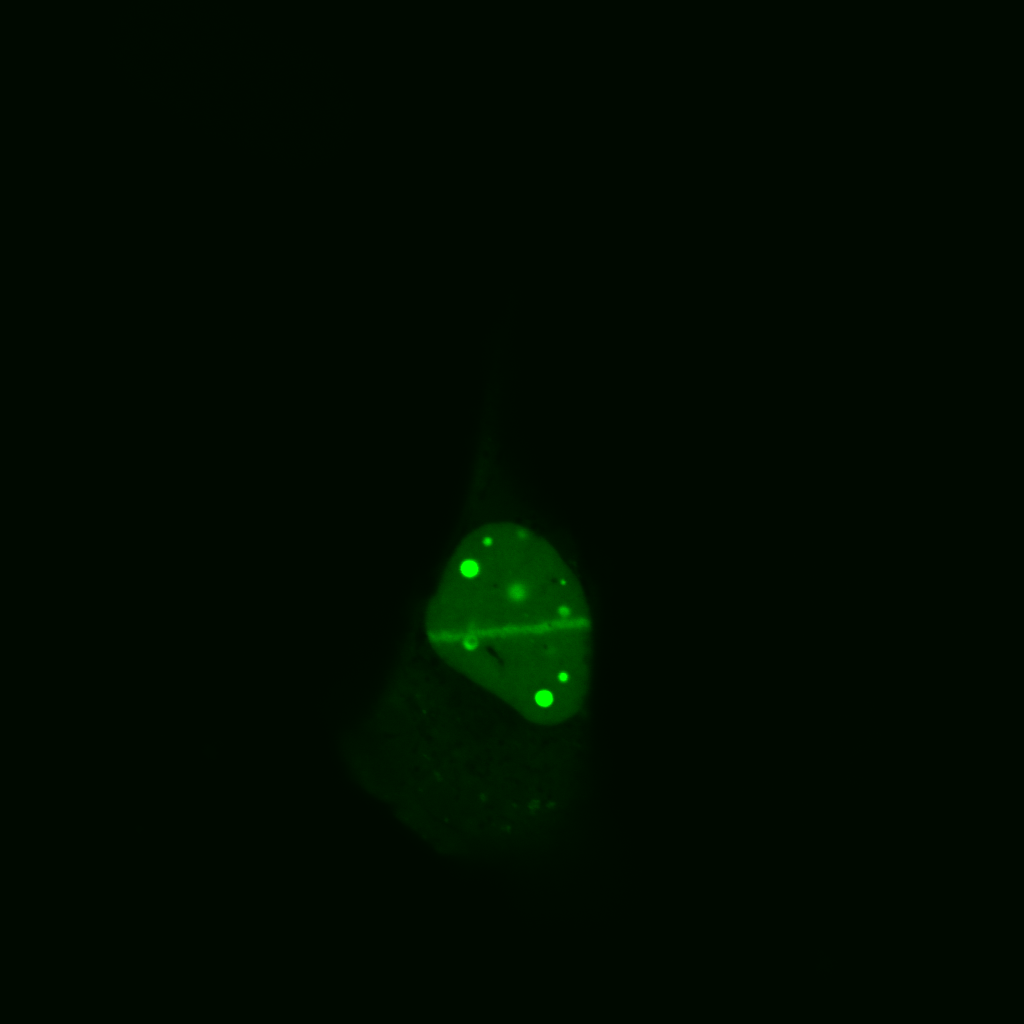

Supplement: Supplementary file 3 — Source data Fig. 1 [file 44319_2024_219_MOESM3_ESM.zip › Figure1/1C/WT/WT-5 min.tif]

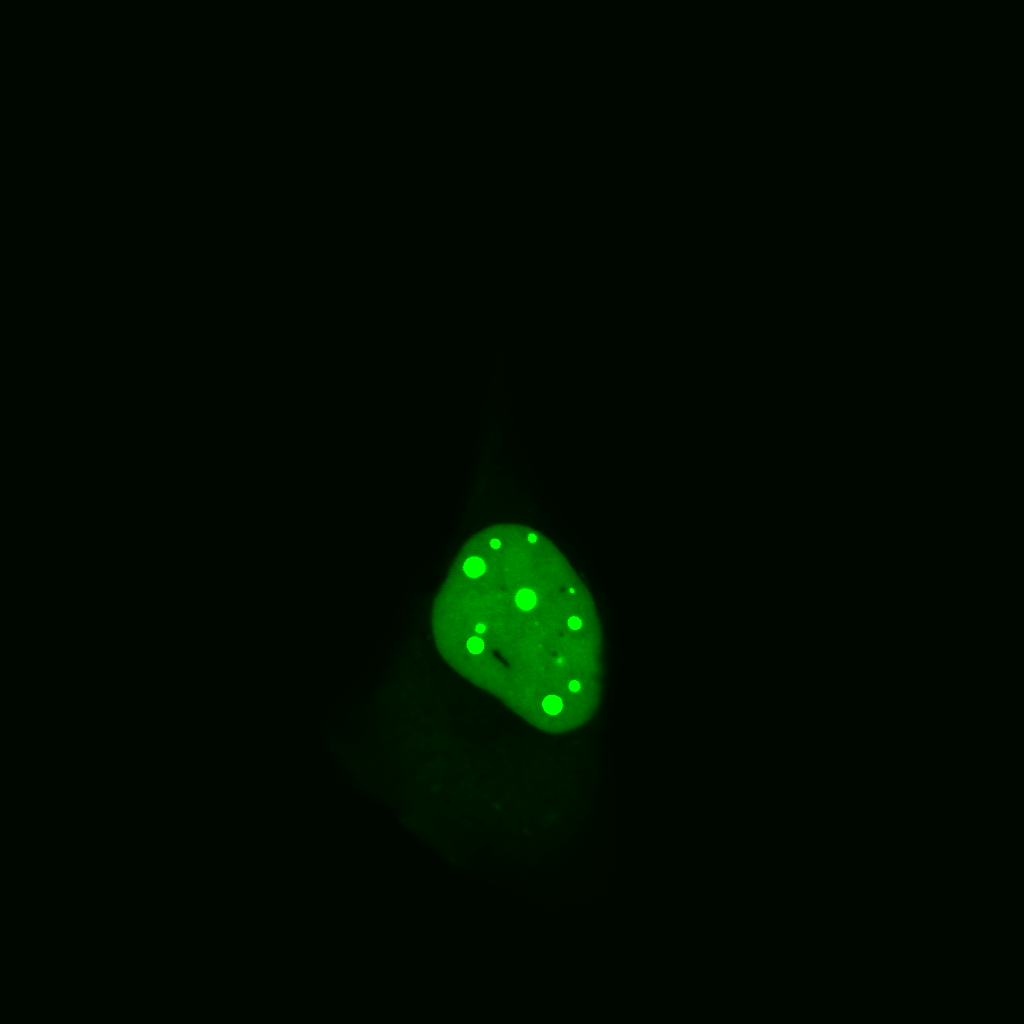

Supplement: Supplementary file 3 — Source data Fig. 1 [file 44319_2024_219_MOESM3_ESM.zip › Figure1/1C/WT/WT-Before.tif]

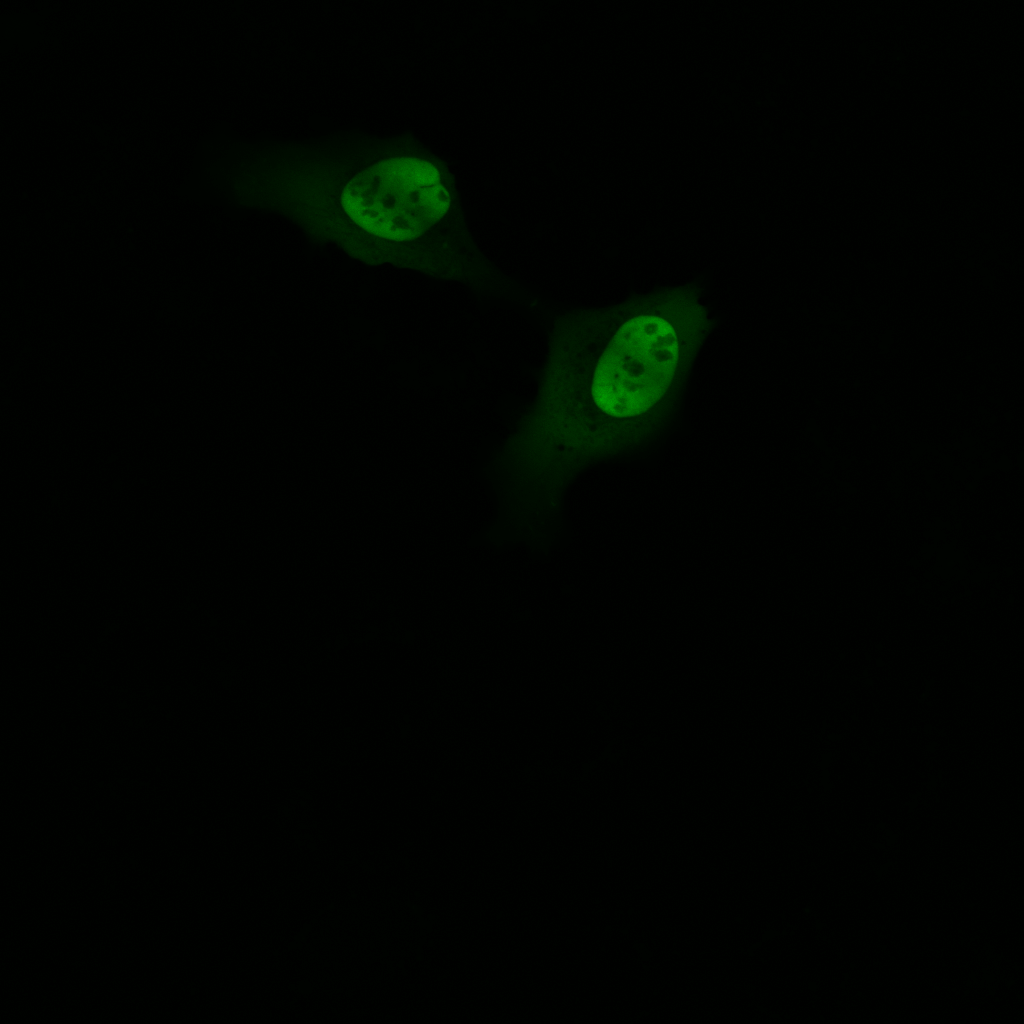

Supplement: Supplementary file 3 — Source data Fig. 1 [file 44319_2024_219_MOESM3_ESM.zip › Figure1/1C/△1-50/△1-50-0 sec.tif]

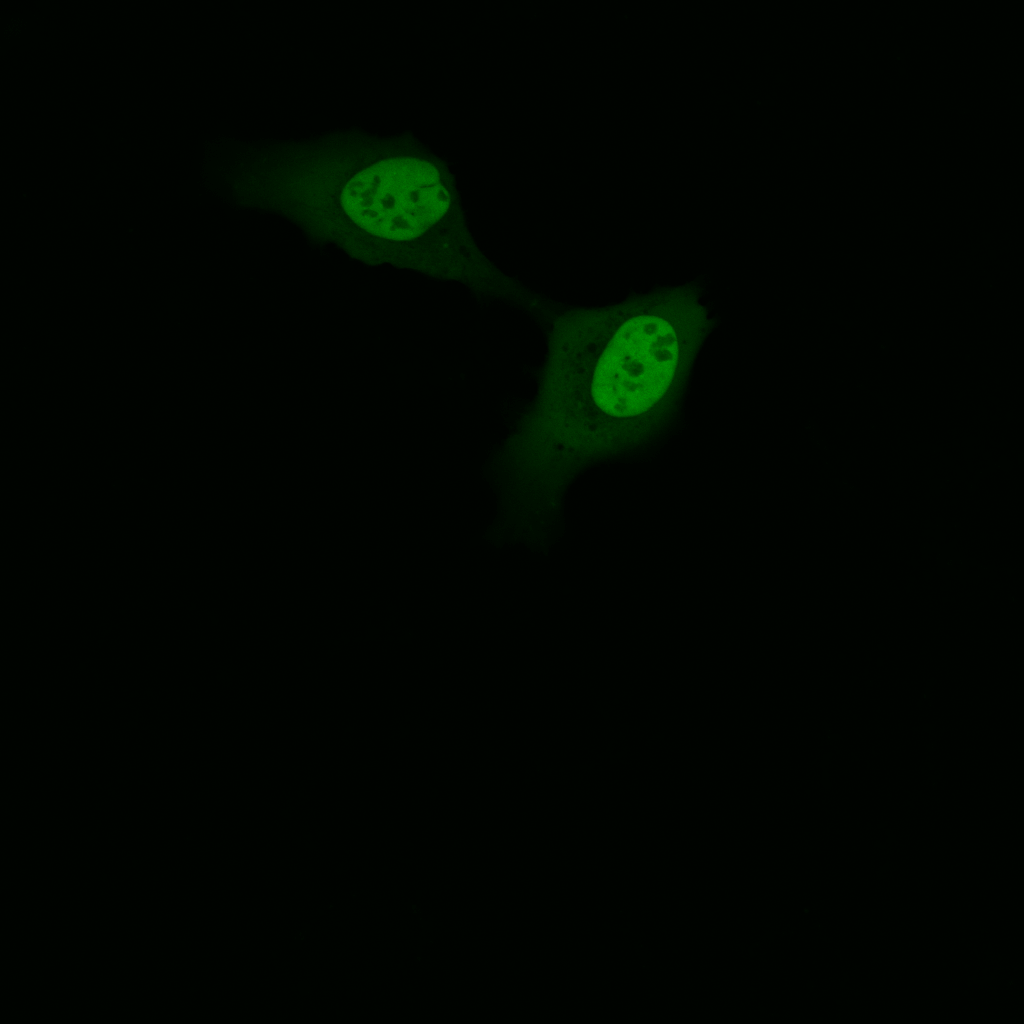

Supplement: Supplementary file 3 — Source data Fig. 1 [file 44319_2024_219_MOESM3_ESM.zip › Figure1/1C/△1-50/△1-50-1 min.tif]

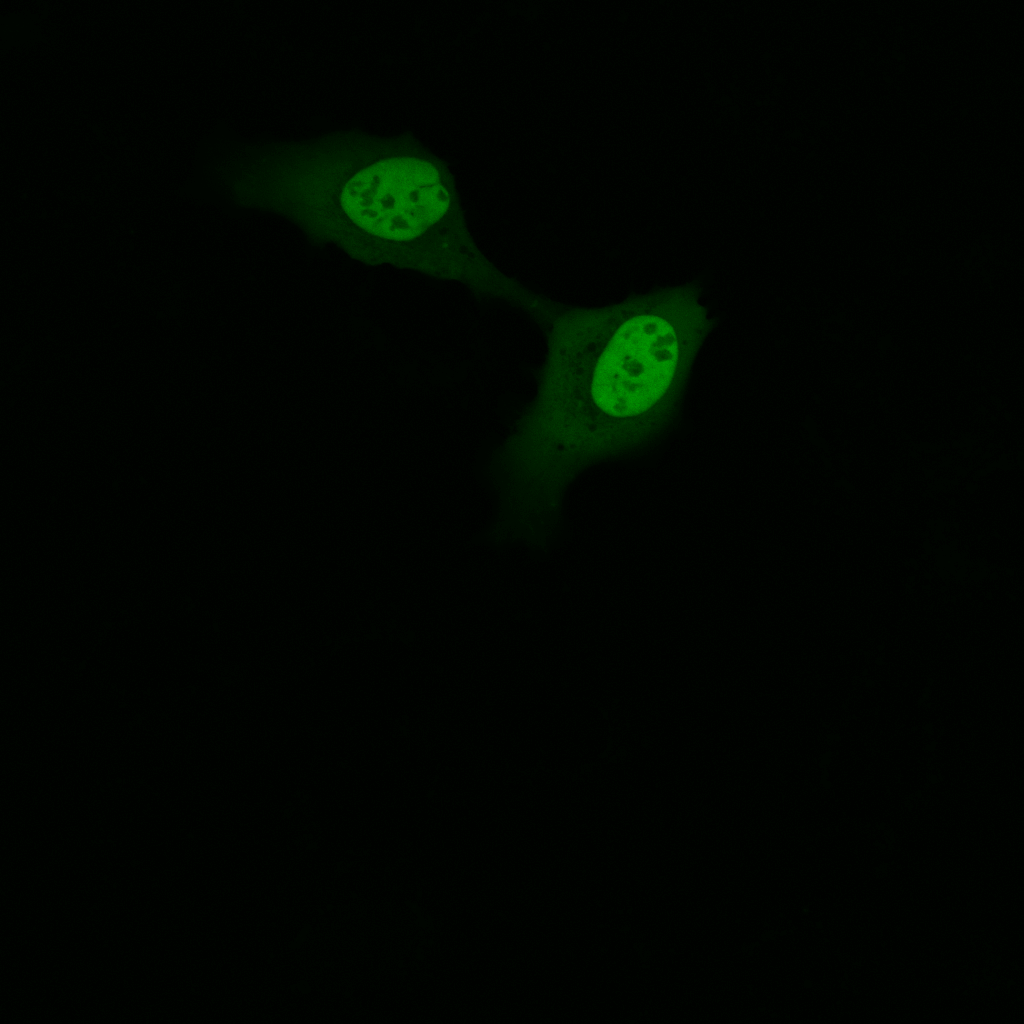

Supplement: Supplementary file 3 — Source data Fig. 1 [file 44319_2024_219_MOESM3_ESM.zip › Figure1/1C/△1-50/△1-50-10 min.tif]

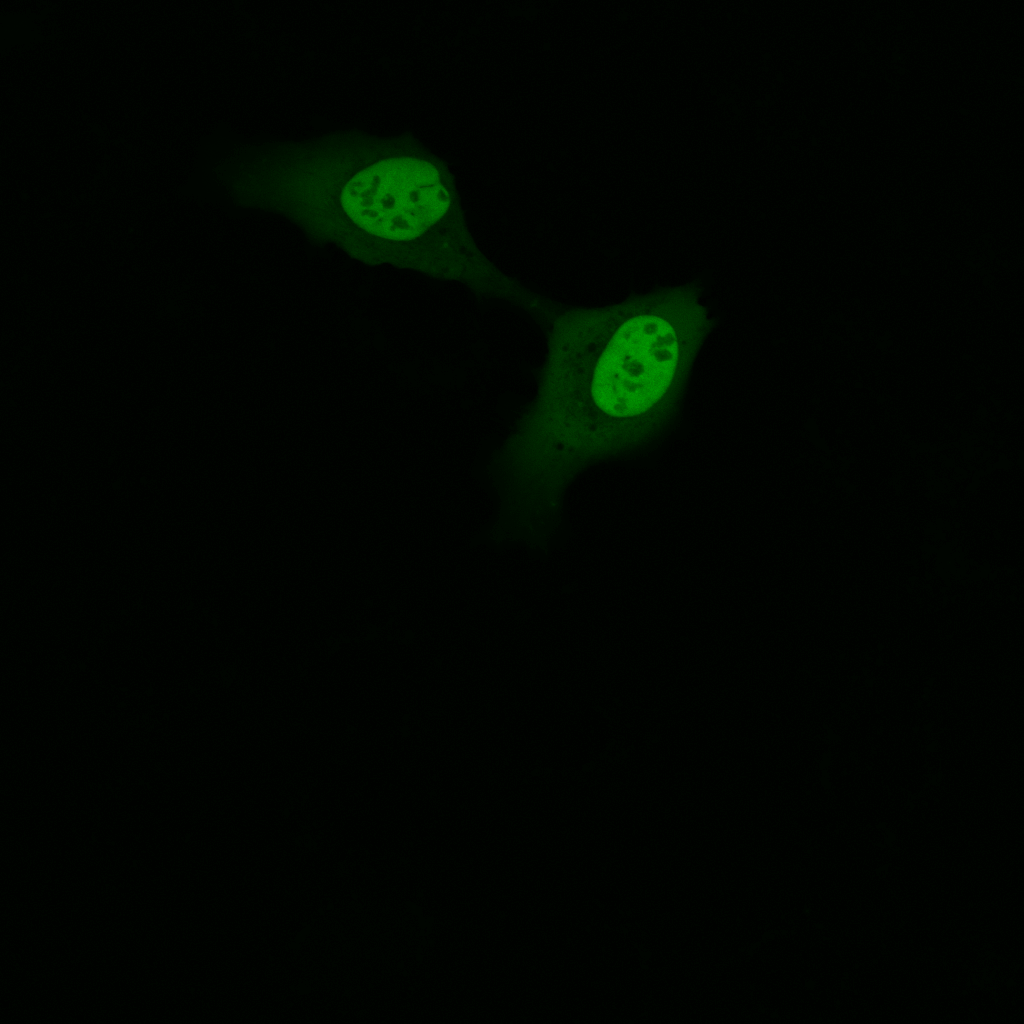

Supplement: Supplementary file 3 — Source data Fig. 1 [file 44319_2024_219_MOESM3_ESM.zip › Figure1/1C/△1-50/△1-50-3 min.tif]

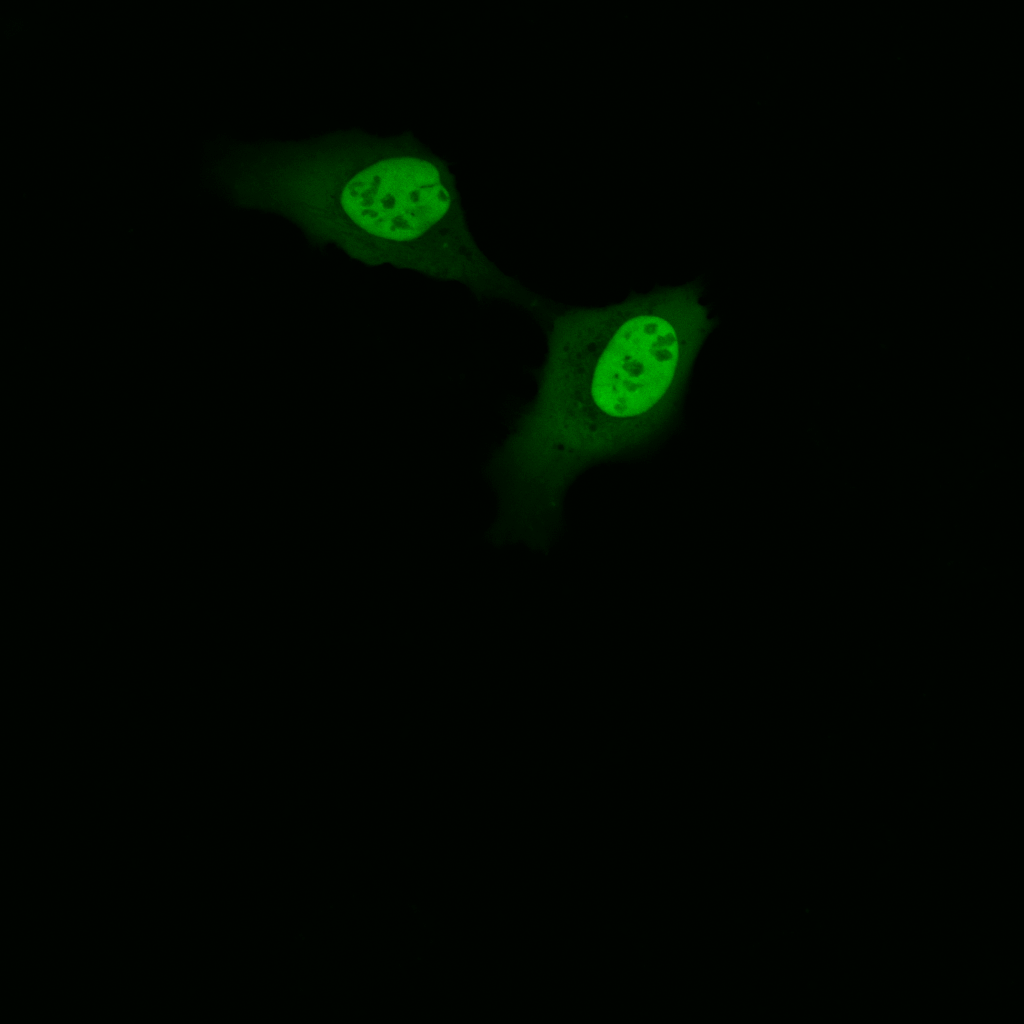

Supplement: Supplementary file 3 — Source data Fig. 1 [file 44319_2024_219_MOESM3_ESM.zip › Figure1/1C/△1-50/△1-50-30 sec.tif]

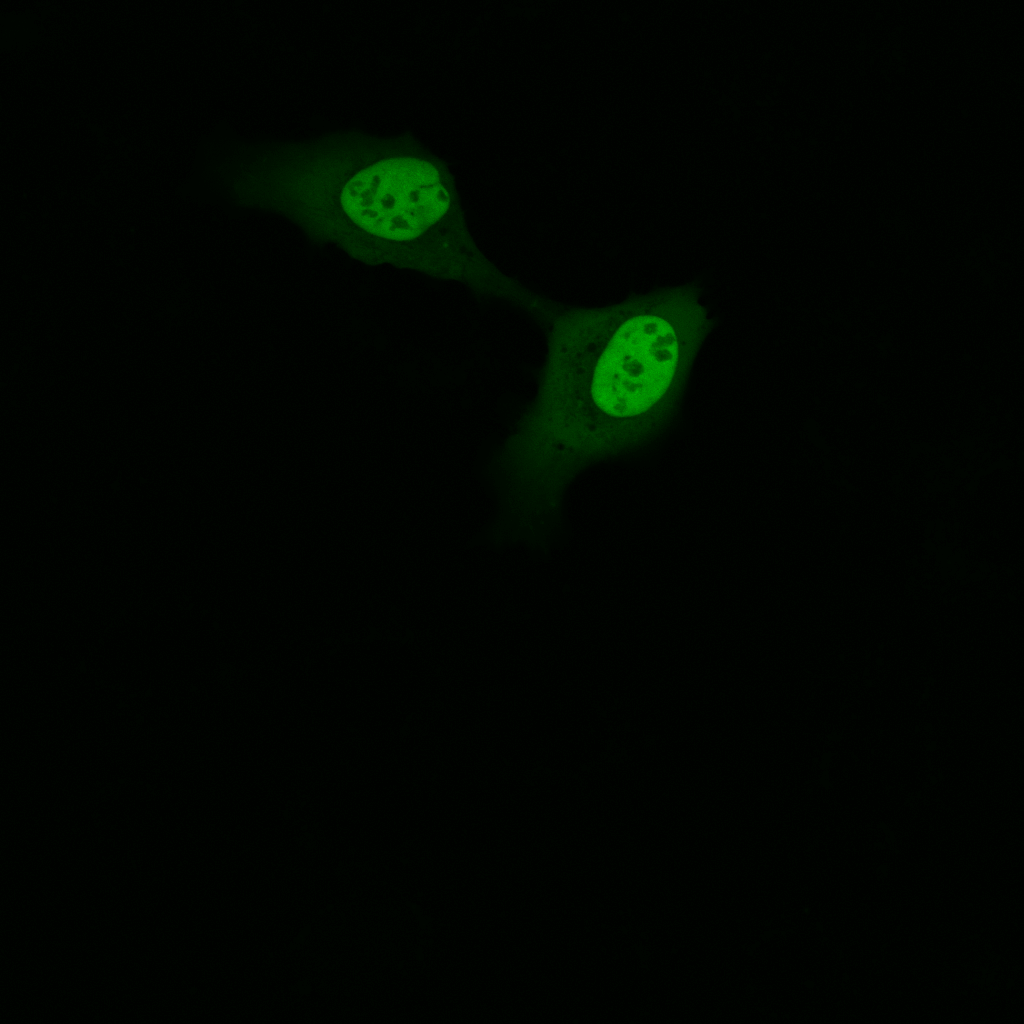

Supplement: Supplementary file 3 — Source data Fig. 1 [file 44319_2024_219_MOESM3_ESM.zip › Figure1/1C/△1-50/△1-50-5 min.tif]

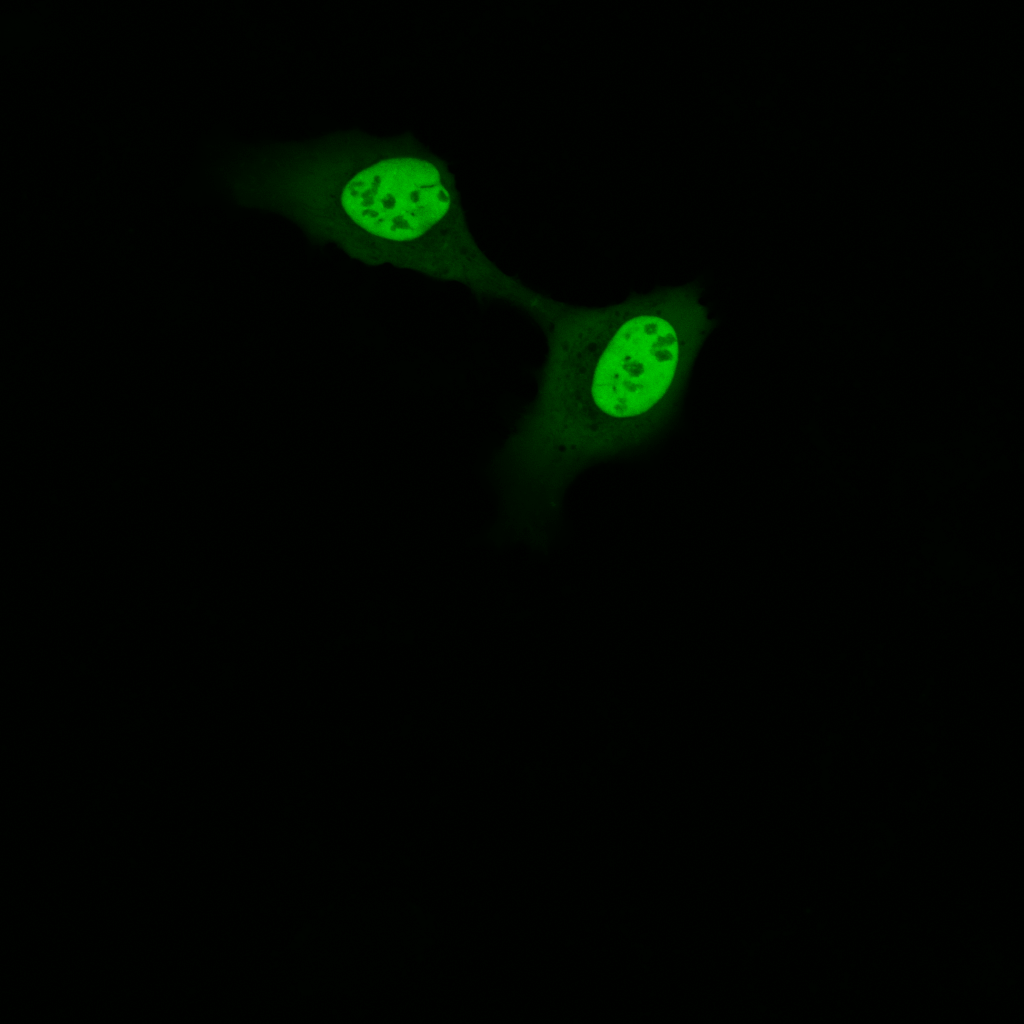

Supplement: Supplementary file 3 — Source data Fig. 1 [file 44319_2024_219_MOESM3_ESM.zip › Figure1/1C/△1-50/△1-50-before.tif]

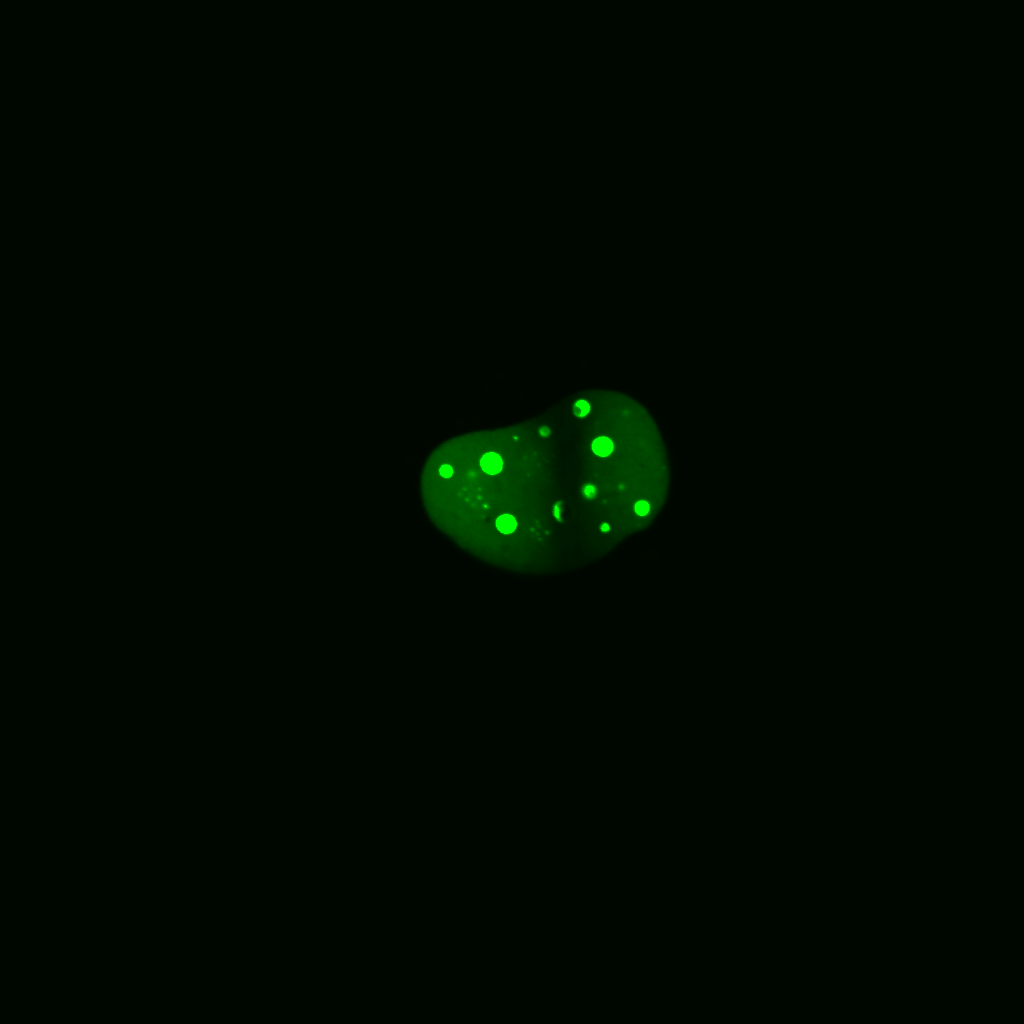

Supplement: Supplementary file 3 — Source data Fig. 1 [file 44319_2024_219_MOESM3_ESM.zip › Figure1/1C/△125-190/△125-190-0 sec.tif]

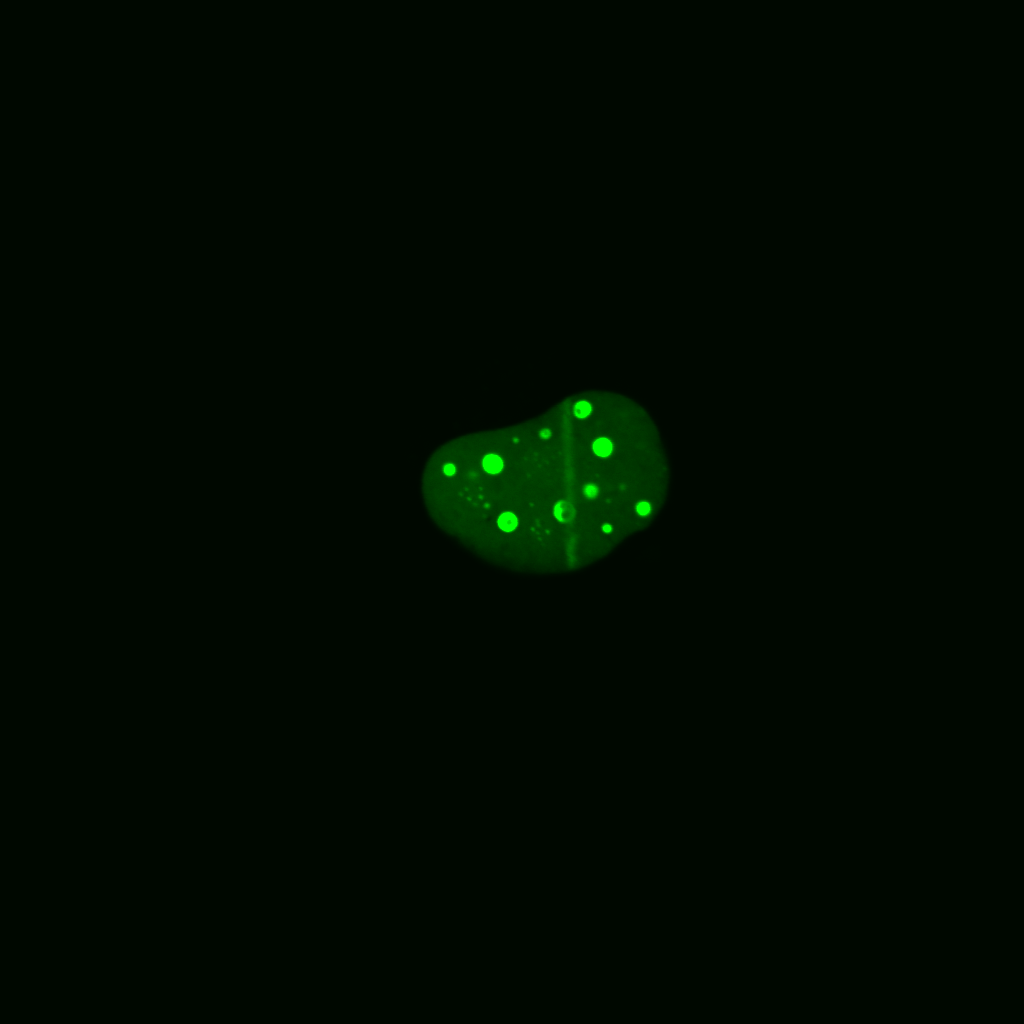

Supplement: Supplementary file 3 — Source data Fig. 1 [file 44319_2024_219_MOESM3_ESM.zip › Figure1/1C/△125-190/△125-190-1 min.tif]

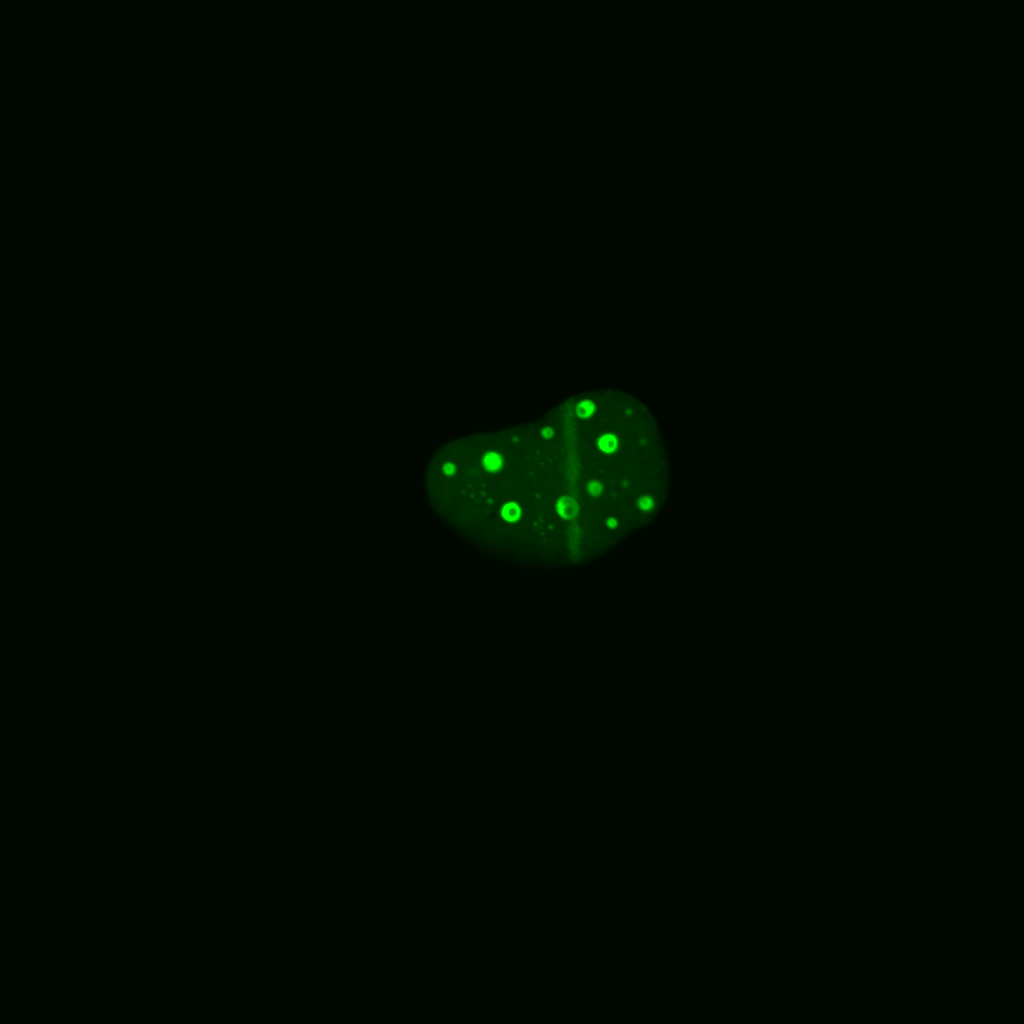

Supplement: Supplementary file 3 — Source data Fig. 1 [file 44319_2024_219_MOESM3_ESM.zip › Figure1/1C/△125-190/△125-190-10 min.tif]

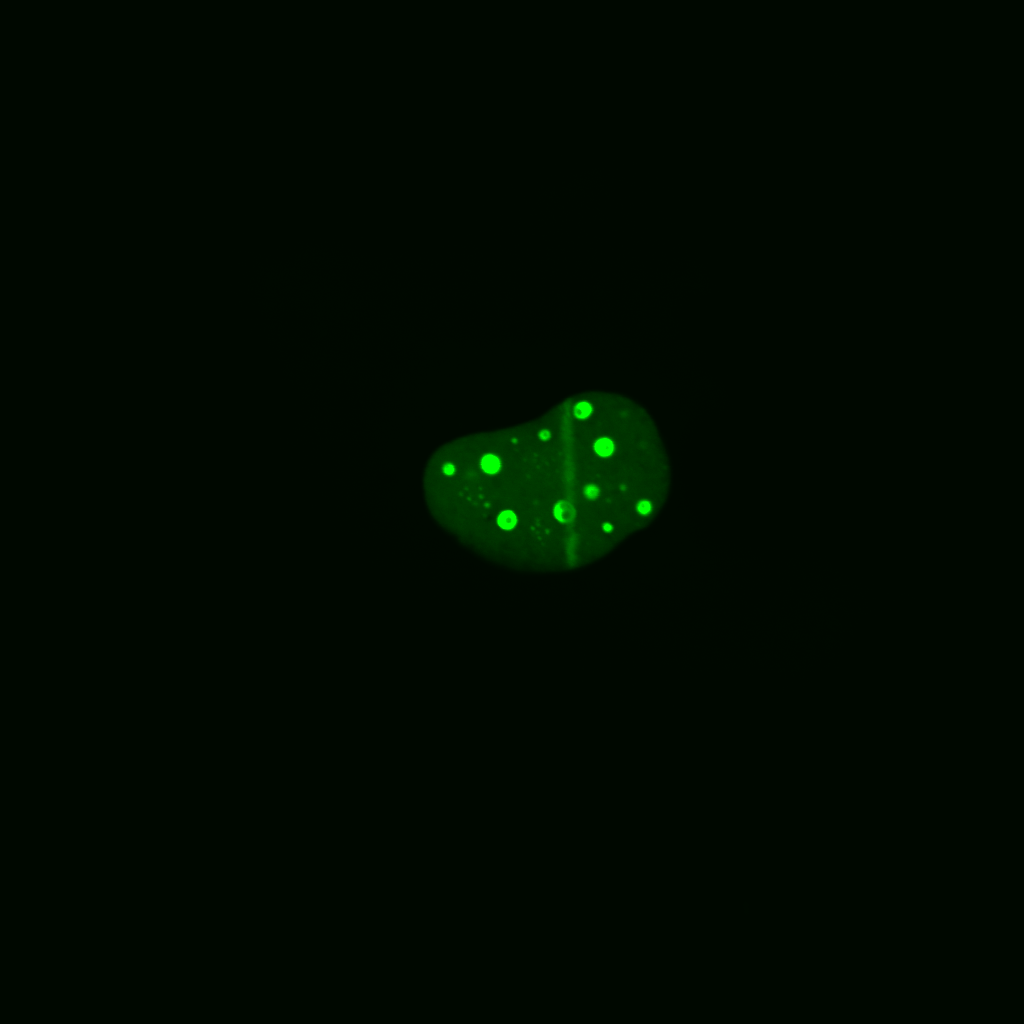

Supplement: Supplementary file 3 — Source data Fig. 1 [file 44319_2024_219_MOESM3_ESM.zip › Figure1/1C/△125-190/△125-190-3 min.tif]

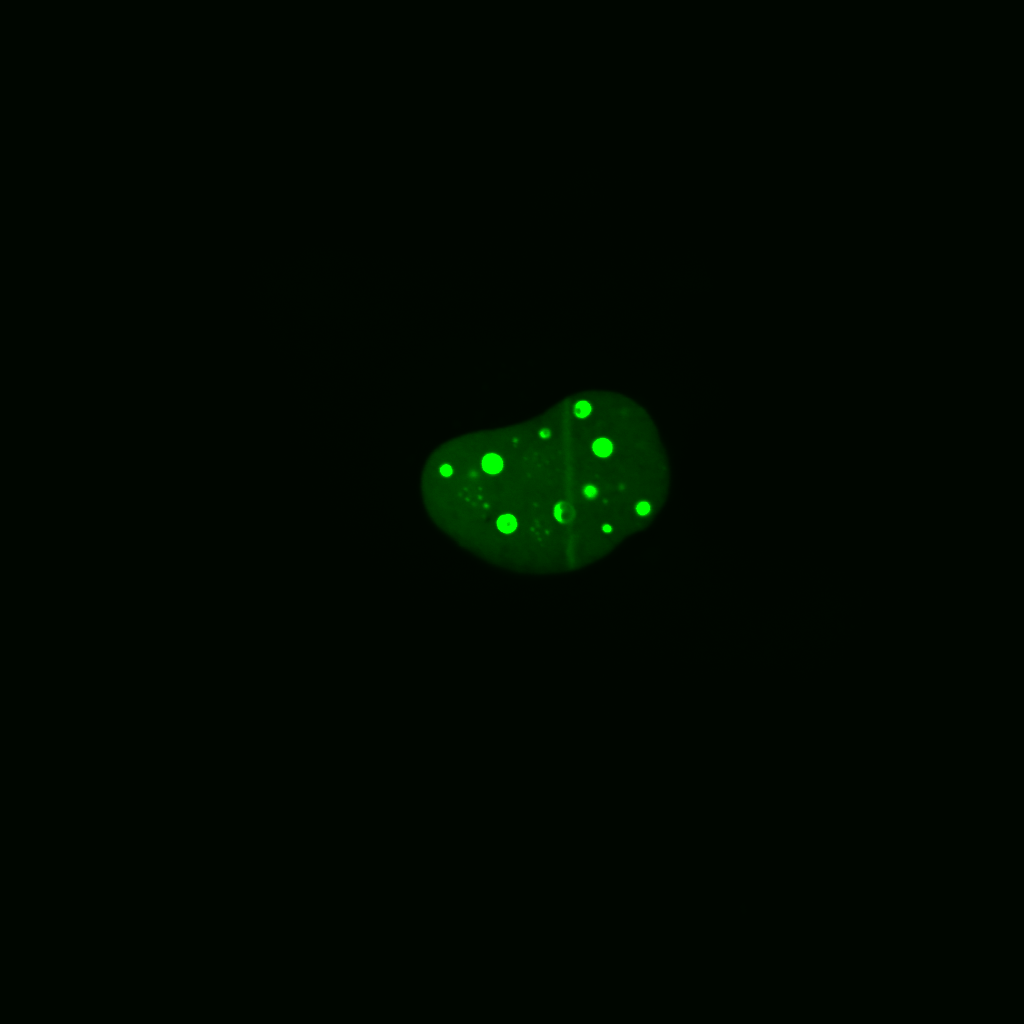

Supplement: Supplementary file 3 — Source data Fig. 1 [file 44319_2024_219_MOESM3_ESM.zip › Figure1/1C/△125-190/△125-190-30 sec.tif]

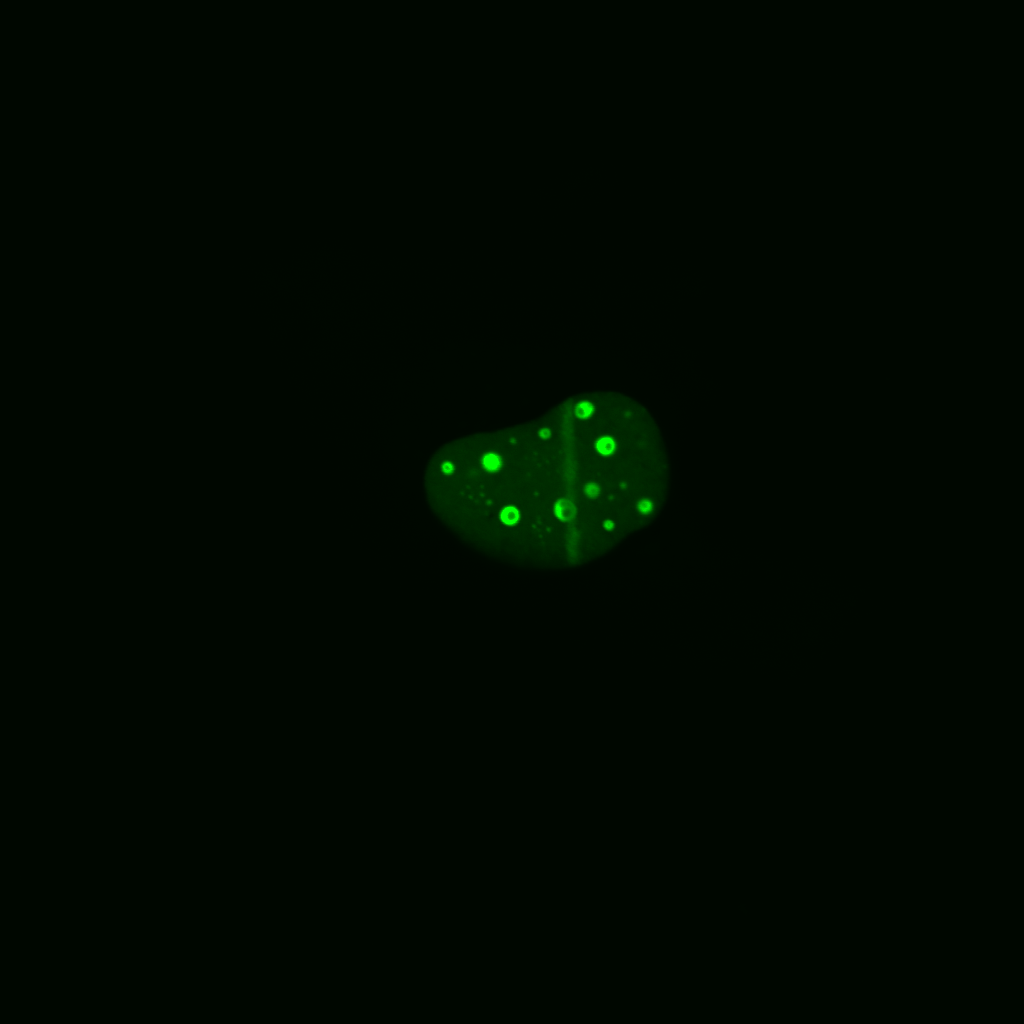

Supplement: Supplementary file 3 — Source data Fig. 1 [file 44319_2024_219_MOESM3_ESM.zip › Figure1/1C/△125-190/△125-190-5 min.tif]

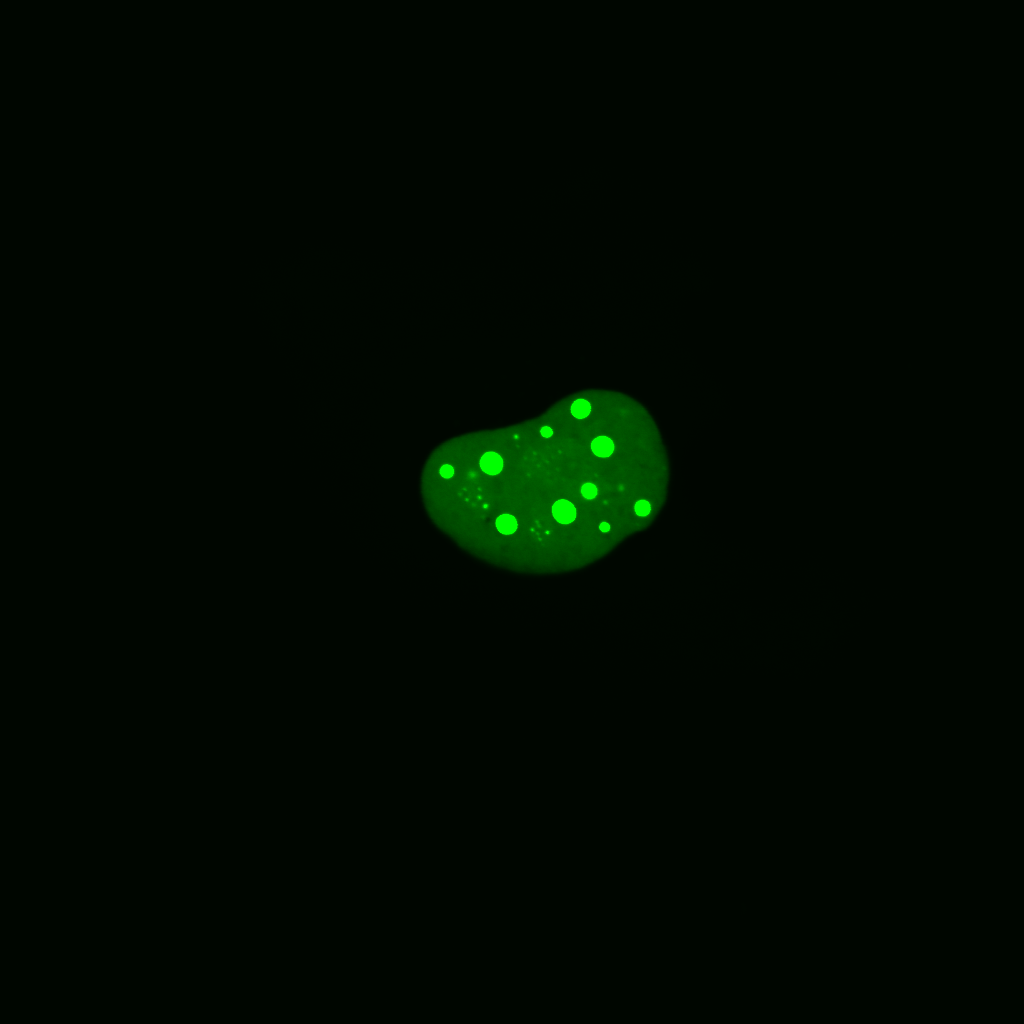

Supplement: Supplementary file 3 — Source data Fig. 1 [file 44319_2024_219_MOESM3_ESM.zip › Figure1/1C/△125-190/△125-190-Before.tif]

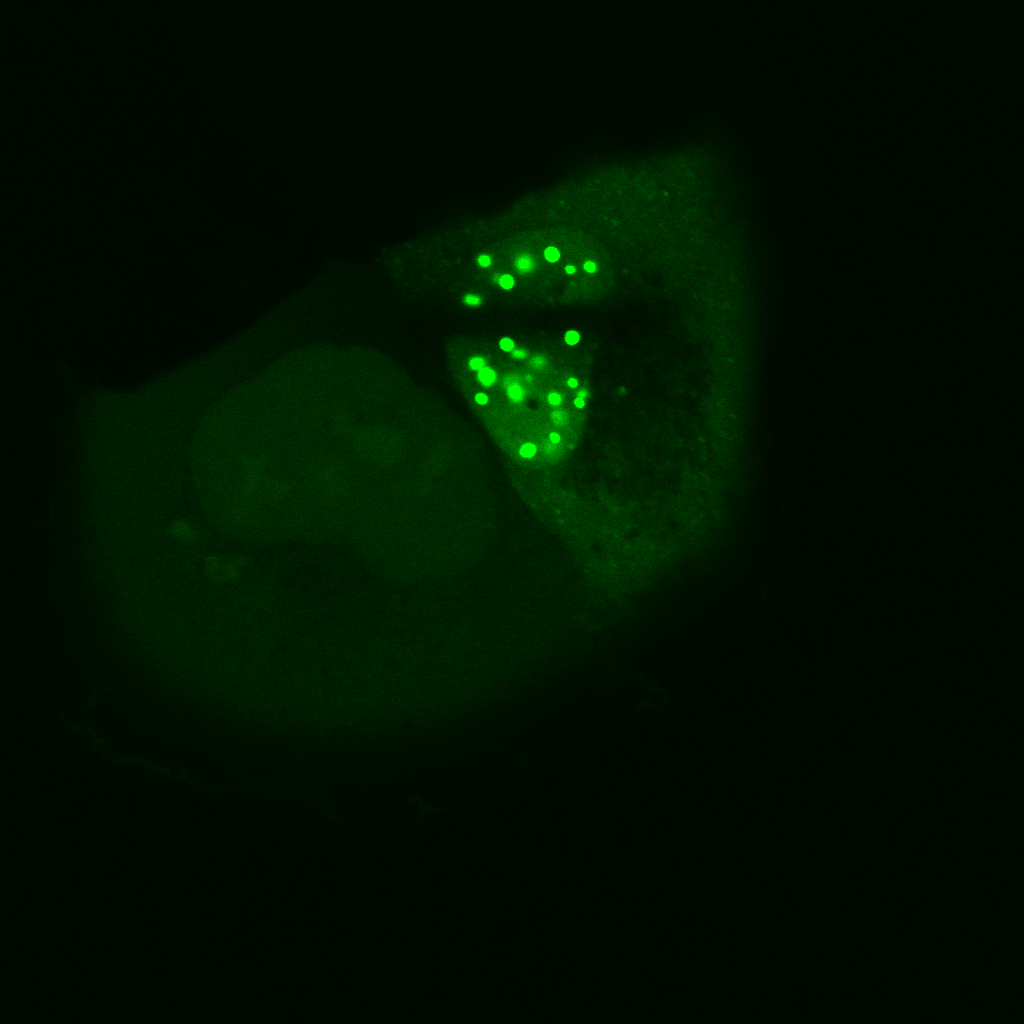

Supplement: Supplementary file 3 — Source data Fig. 1 [file 44319_2024_219_MOESM3_ESM.zip › Figure1/1C/△190-262/△190-262-0 sec.tif]

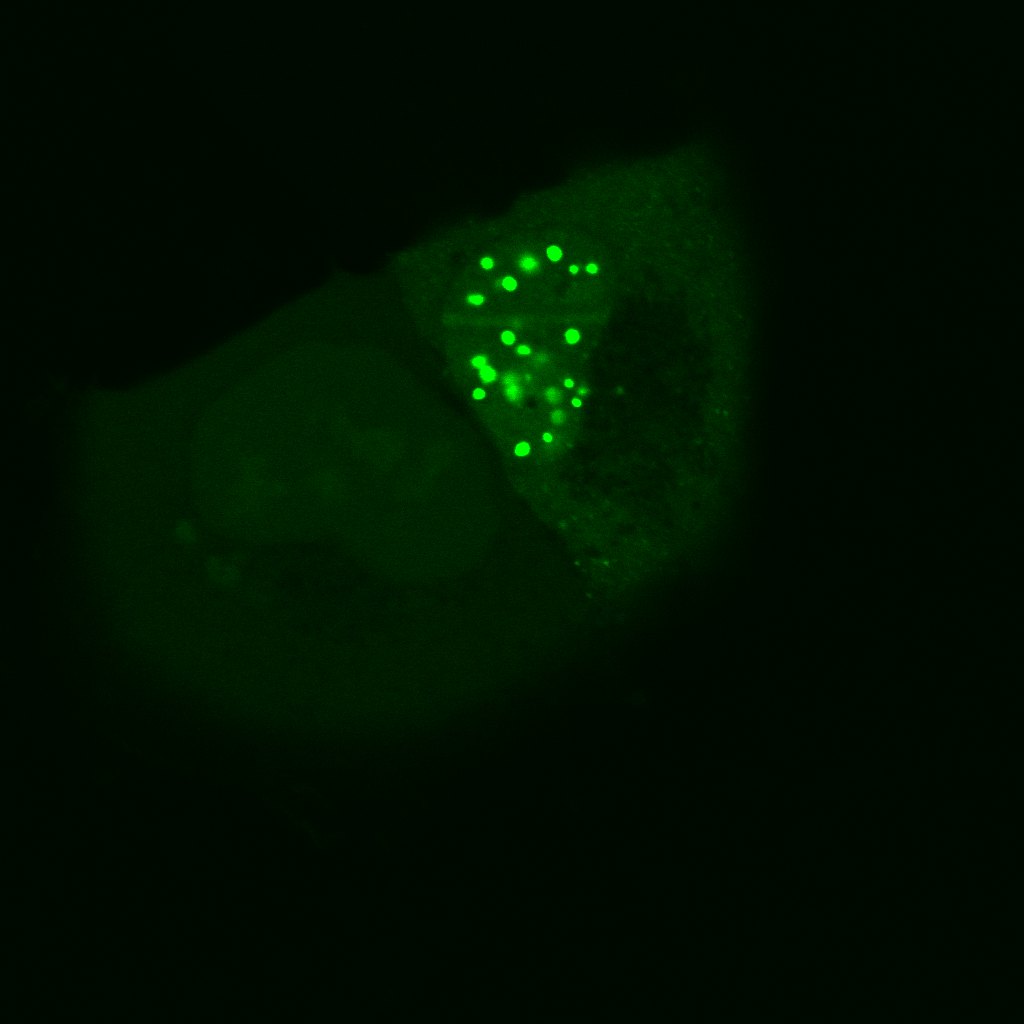

Supplement: Supplementary file 3 — Source data Fig. 1 [file 44319_2024_219_MOESM3_ESM.zip › Figure1/1C/△190-262/△190-262-1 min.tif]

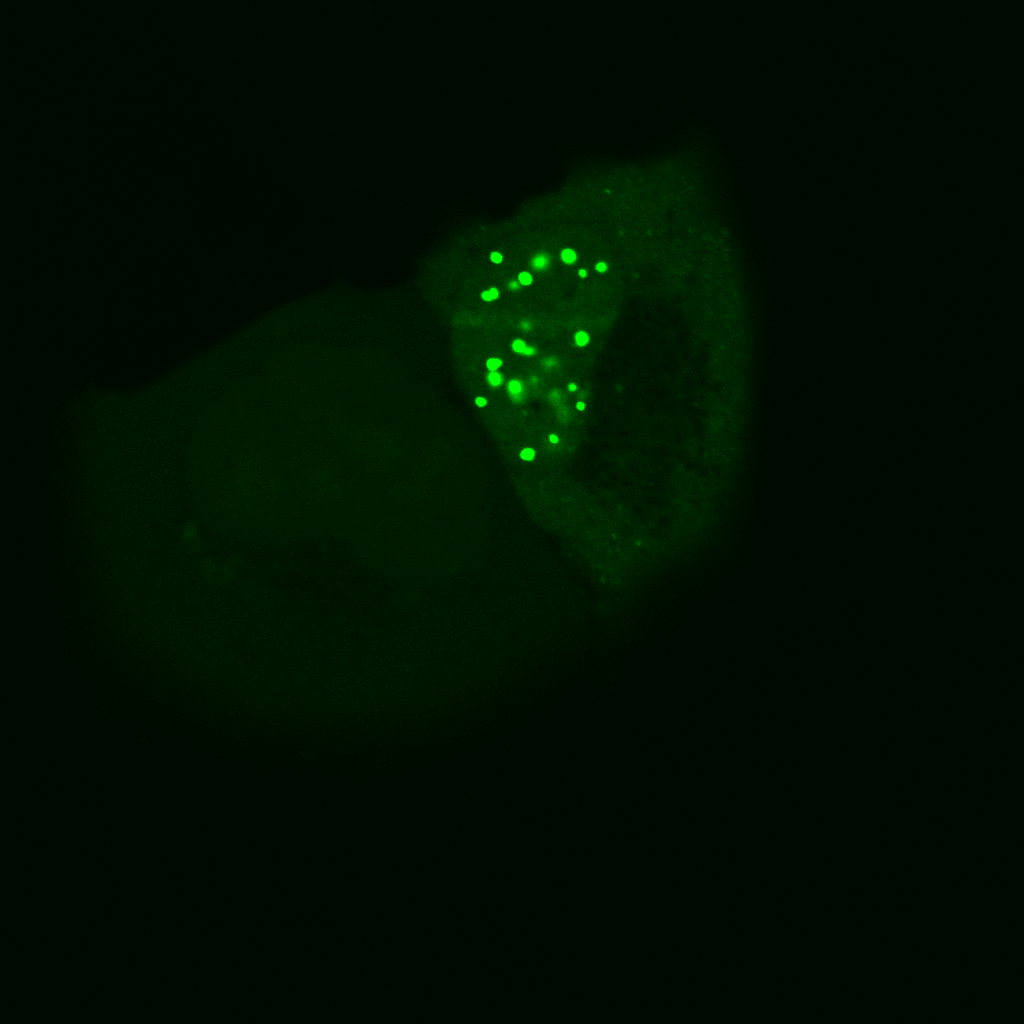

Supplement: Supplementary file 3 — Source data Fig. 1 [file 44319_2024_219_MOESM3_ESM.zip › Figure1/1C/△190-262/△190-262-10 min.tif]

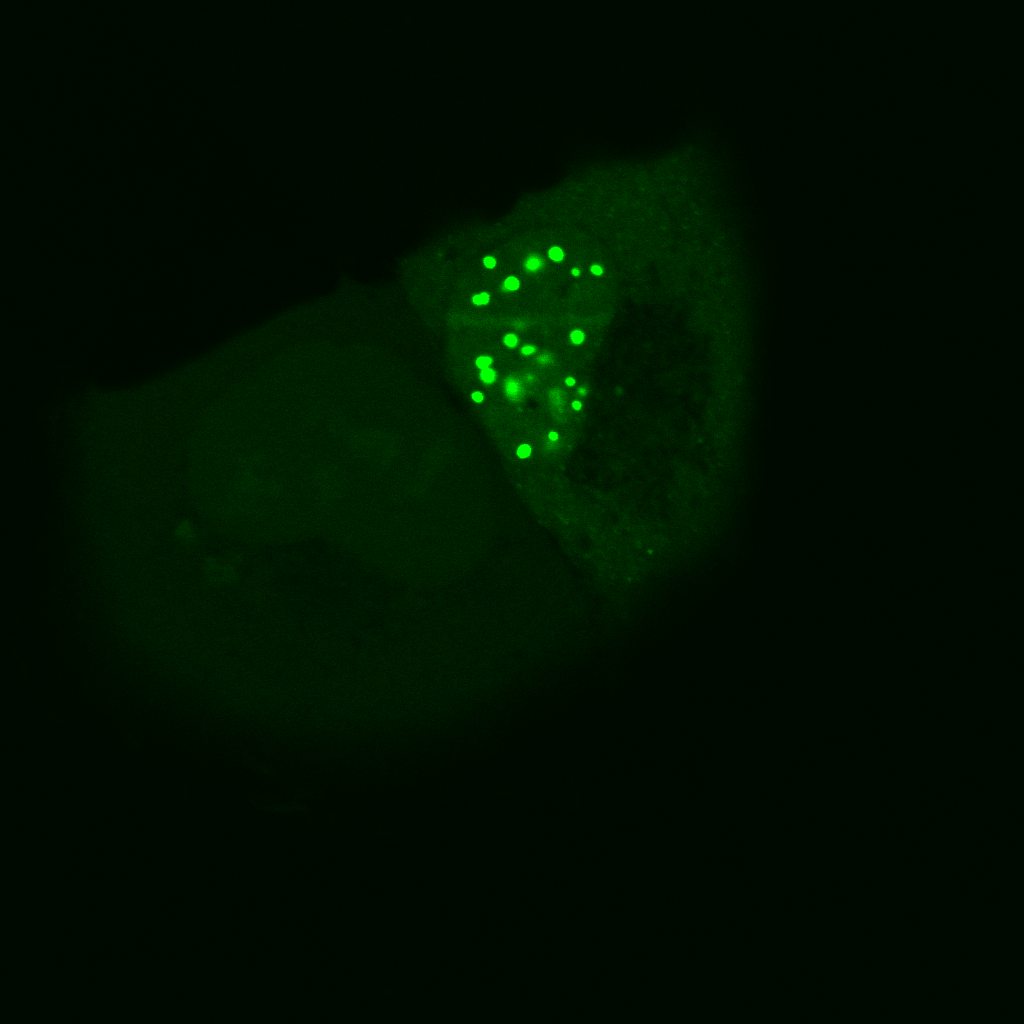

Supplement: Supplementary file 3 — Source data Fig. 1 [file 44319_2024_219_MOESM3_ESM.zip › Figure1/1C/△190-262/△190-262-3 min.tif]

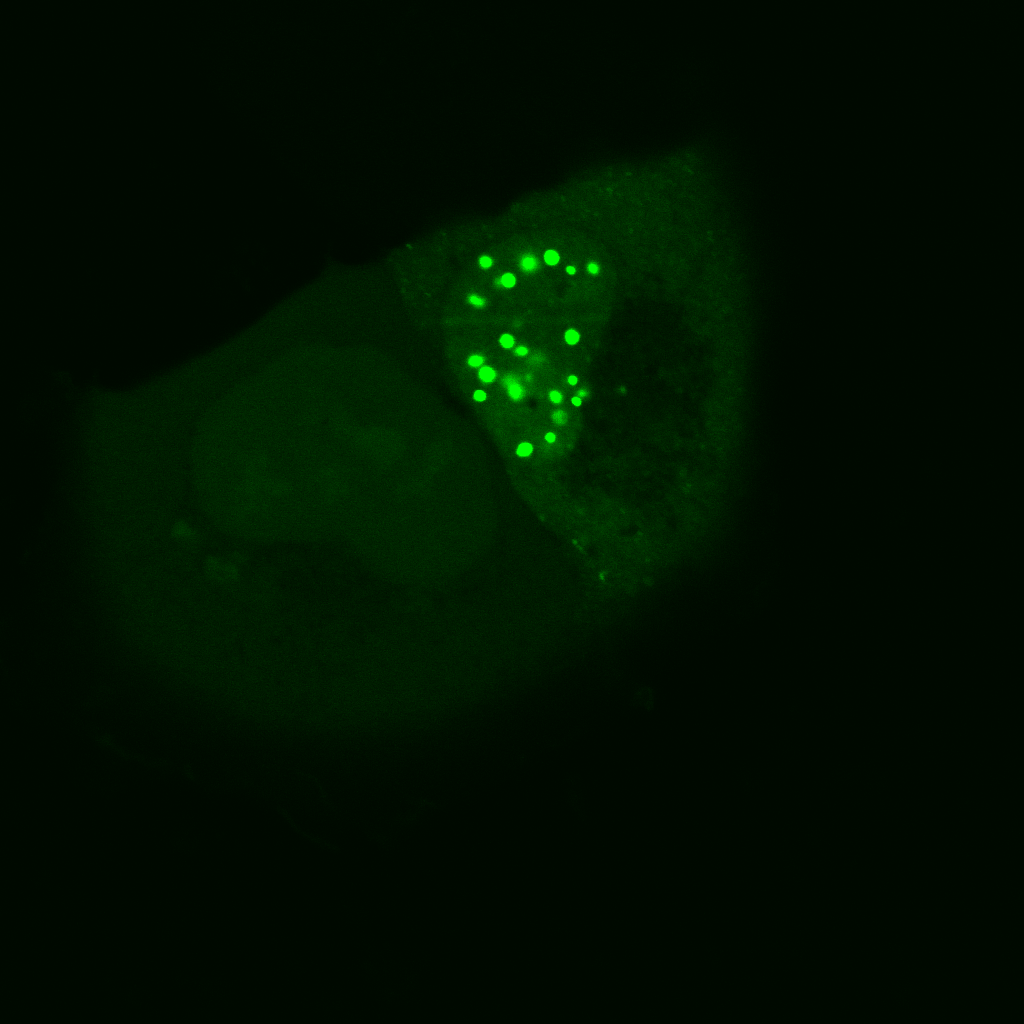

Supplement: Supplementary file 3 — Source data Fig. 1 [file 44319_2024_219_MOESM3_ESM.zip › Figure1/1C/△190-262/△190-262-30 sec.tif]

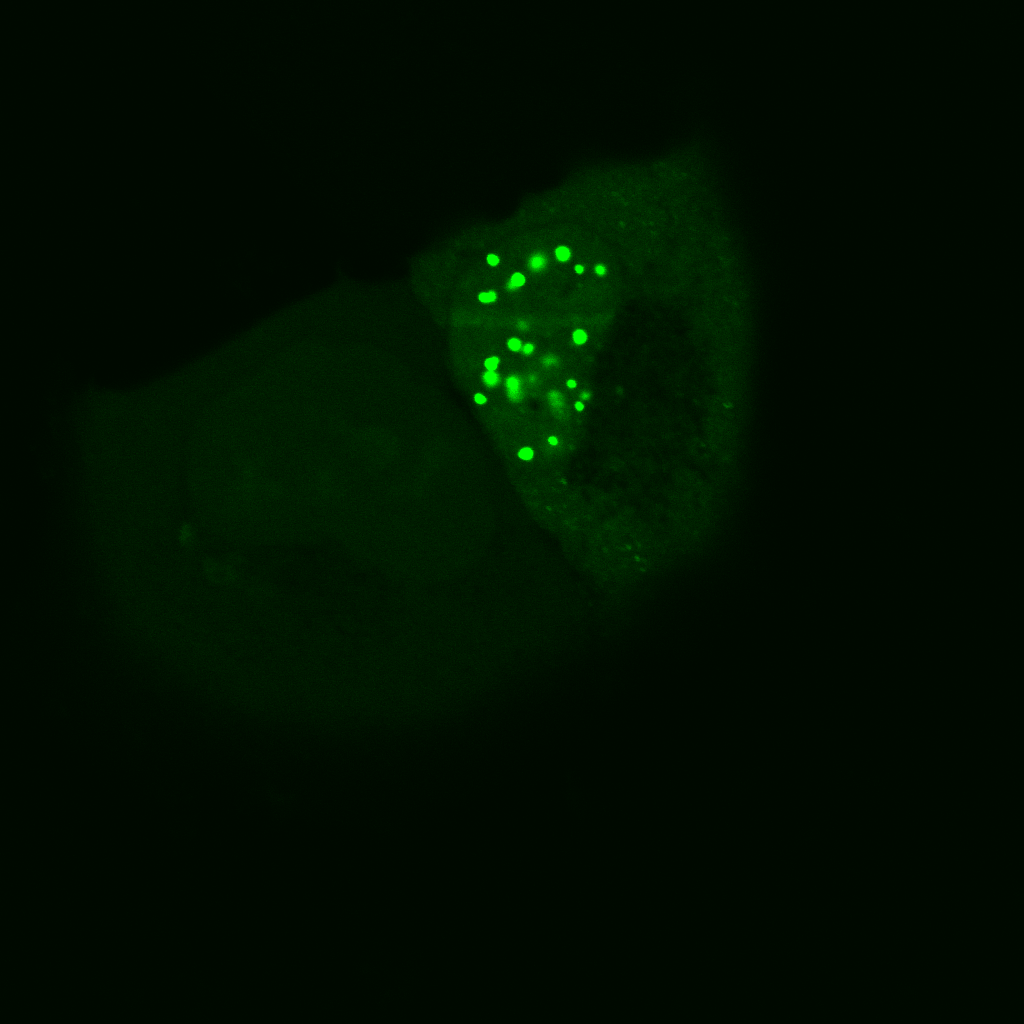

Supplement: Supplementary file 3 — Source data Fig. 1 [file 44319_2024_219_MOESM3_ESM.zip › Figure1/1C/△190-262/△190-262-5 min.tif]

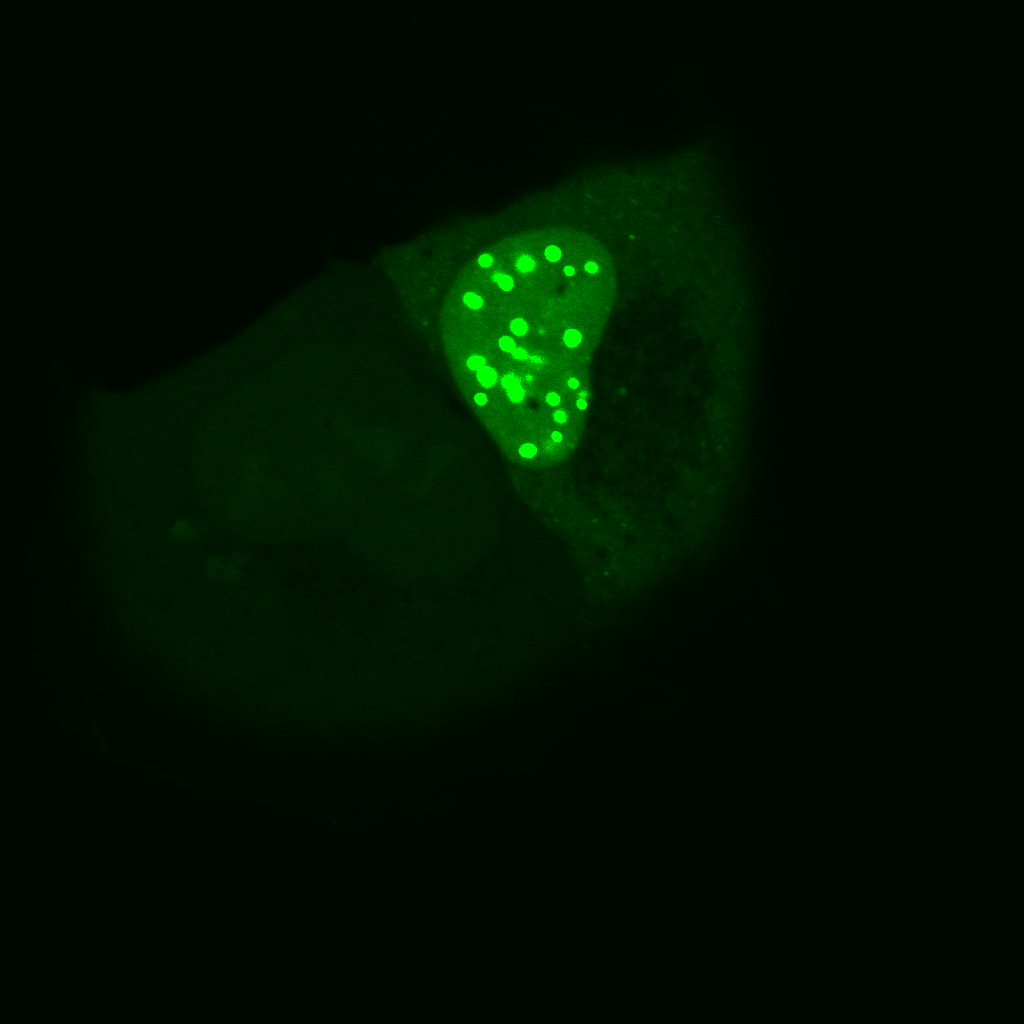

Supplement: Supplementary file 3 — Source data Fig. 1 [file 44319_2024_219_MOESM3_ESM.zip › Figure1/1C/△190-262/△190-262-Before.tif]

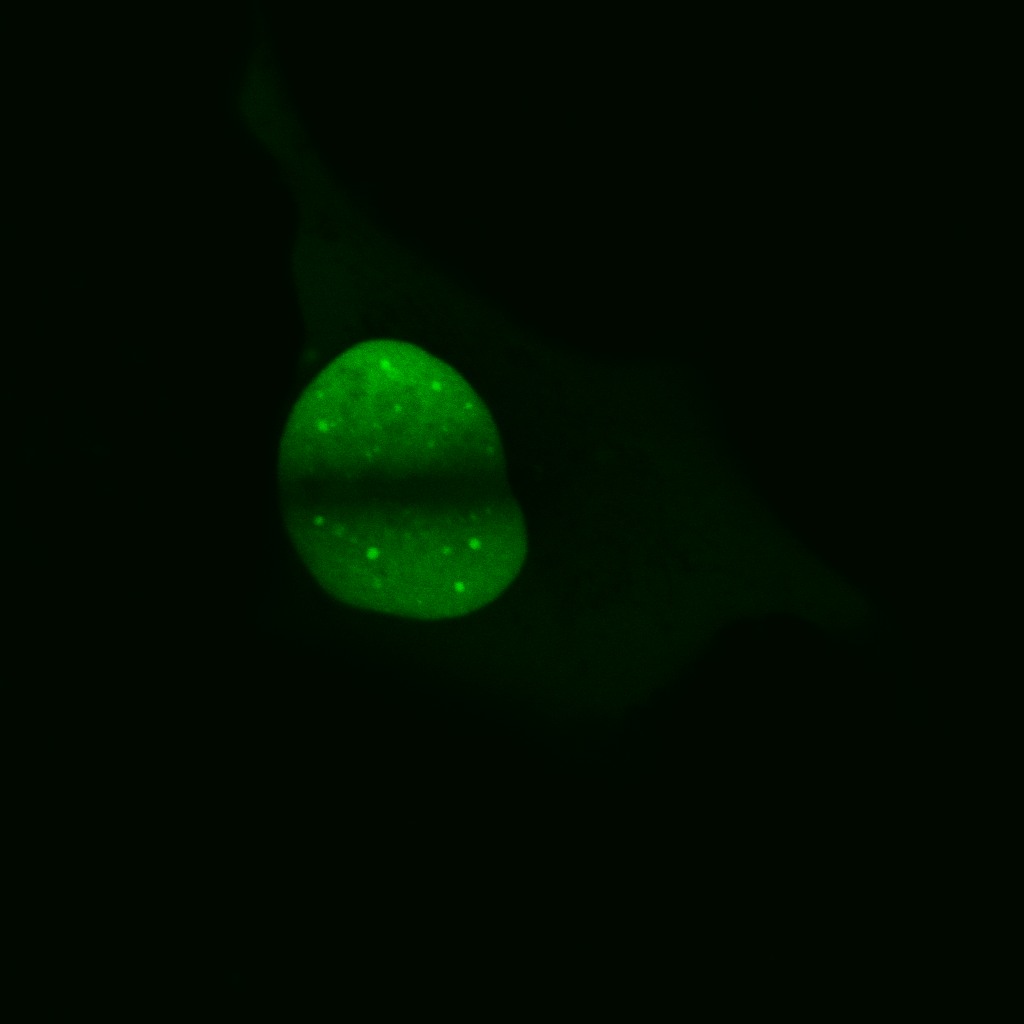

Supplement: Supplementary file 3 — Source data Fig. 1 [file 44319_2024_219_MOESM3_ESM.zip › Figure1/1C/△51-125/△51-125-0 sec.tif]

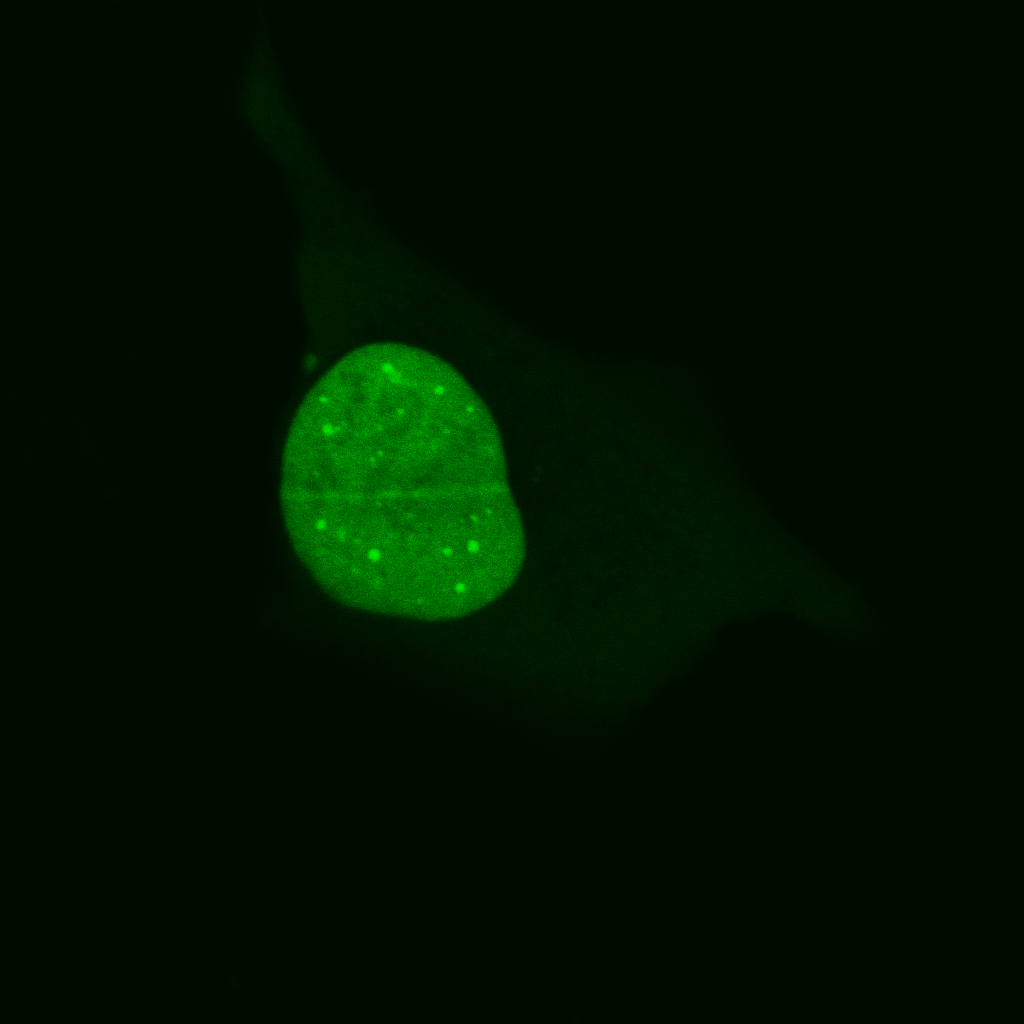

Supplement: Supplementary file 3 — Source data Fig. 1 [file 44319_2024_219_MOESM3_ESM.zip › Figure1/1C/△51-125/△51-125-1 min.tif]

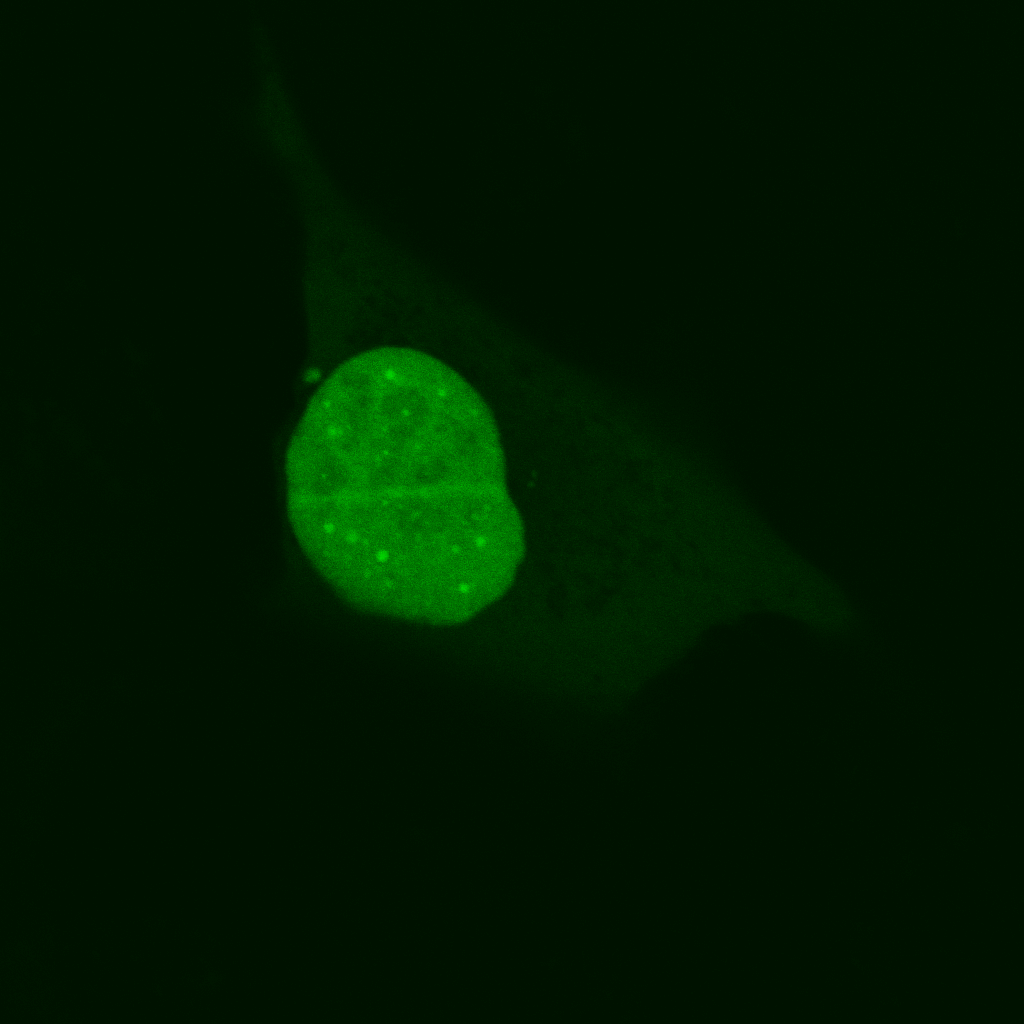

Supplement: Supplementary file 3 — Source data Fig. 1 [file 44319_2024_219_MOESM3_ESM.zip › Figure1/1C/△51-125/△51-125-10 min.tif]

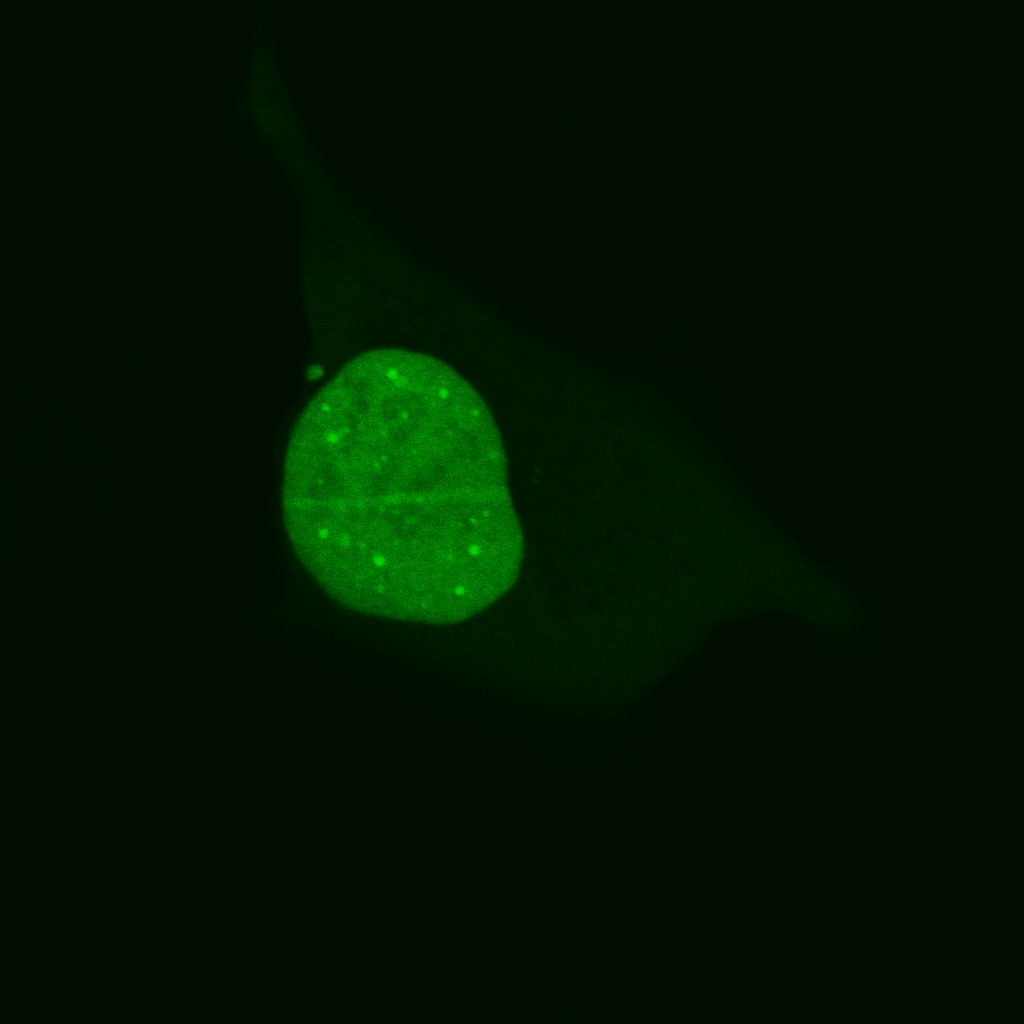

Supplement: Supplementary file 3 — Source data Fig. 1 [file 44319_2024_219_MOESM3_ESM.zip › Figure1/1C/△51-125/△51-125-3 min.tif]

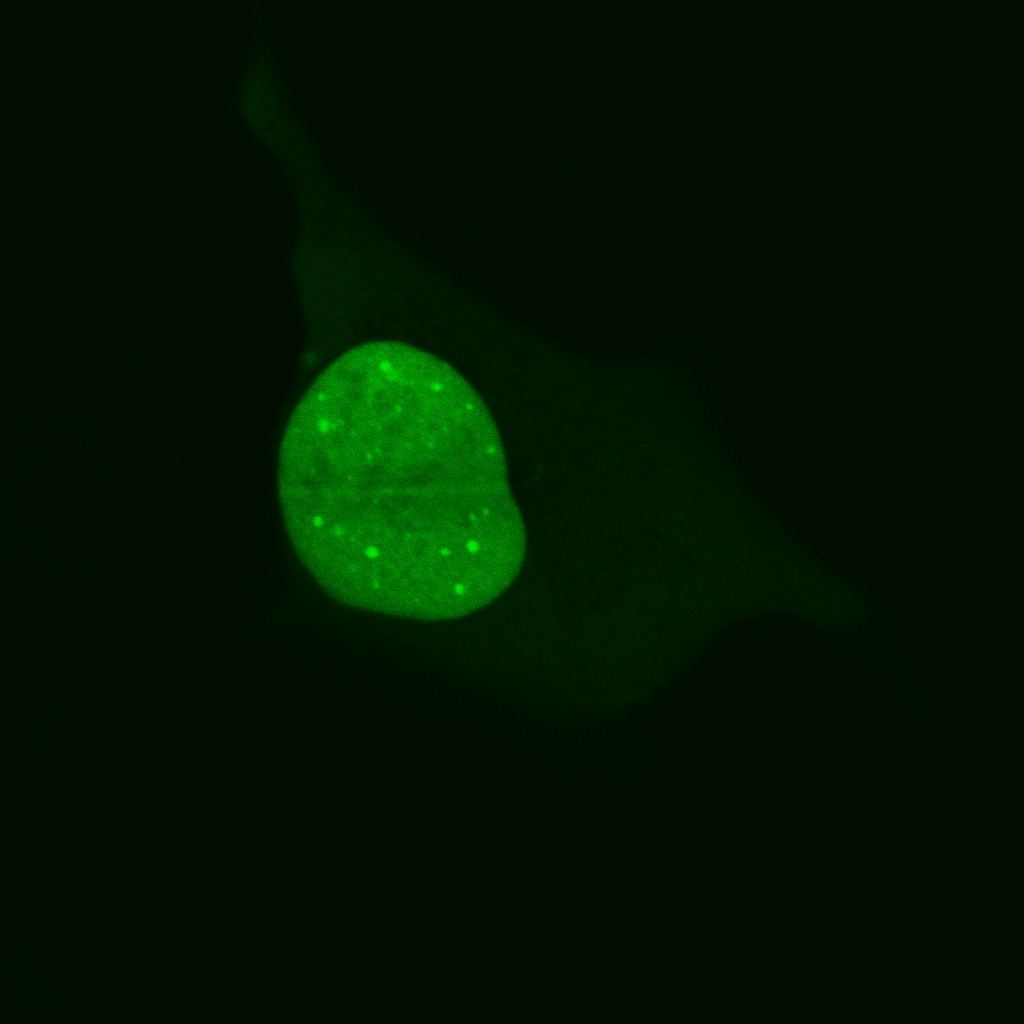

Supplement: Supplementary file 3 — Source data Fig. 1 [file 44319_2024_219_MOESM3_ESM.zip › Figure1/1C/△51-125/△51-125-30 sec.tif]

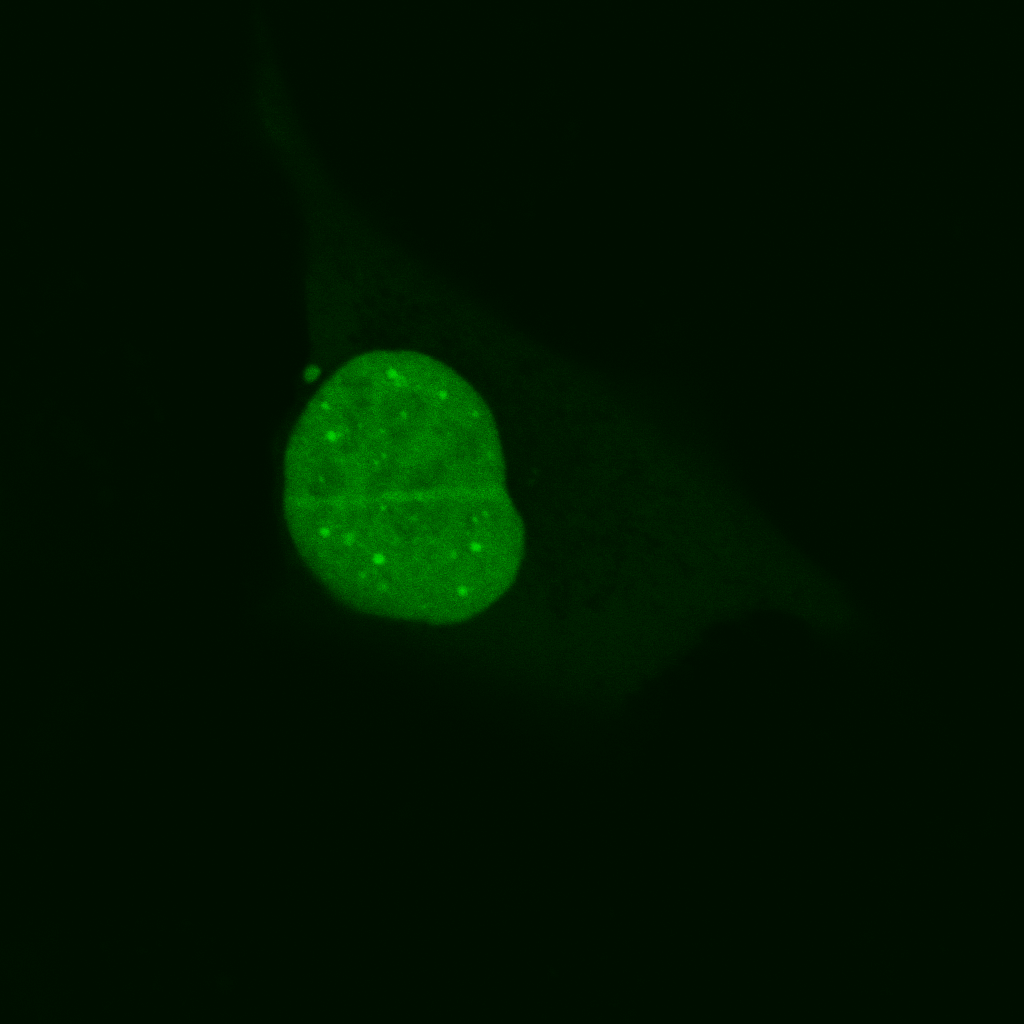

Supplement: Supplementary file 3 — Source data Fig. 1 [file 44319_2024_219_MOESM3_ESM.zip › Figure1/1C/△51-125/△51-125-5 min.tif]

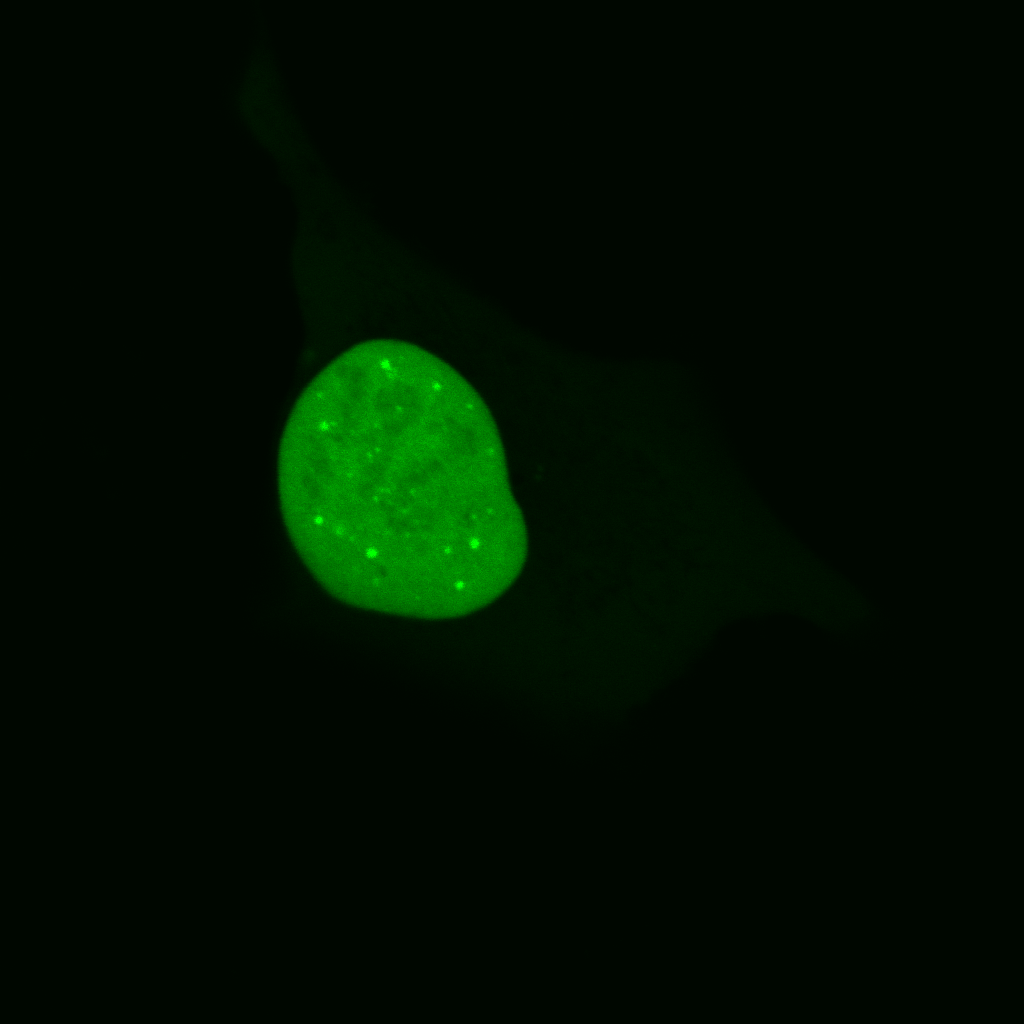

Supplement: Supplementary file 3 — Source data Fig. 1 [file 44319_2024_219_MOESM3_ESM.zip › Figure1/1C/△51-125/△51-125-Before.tif]

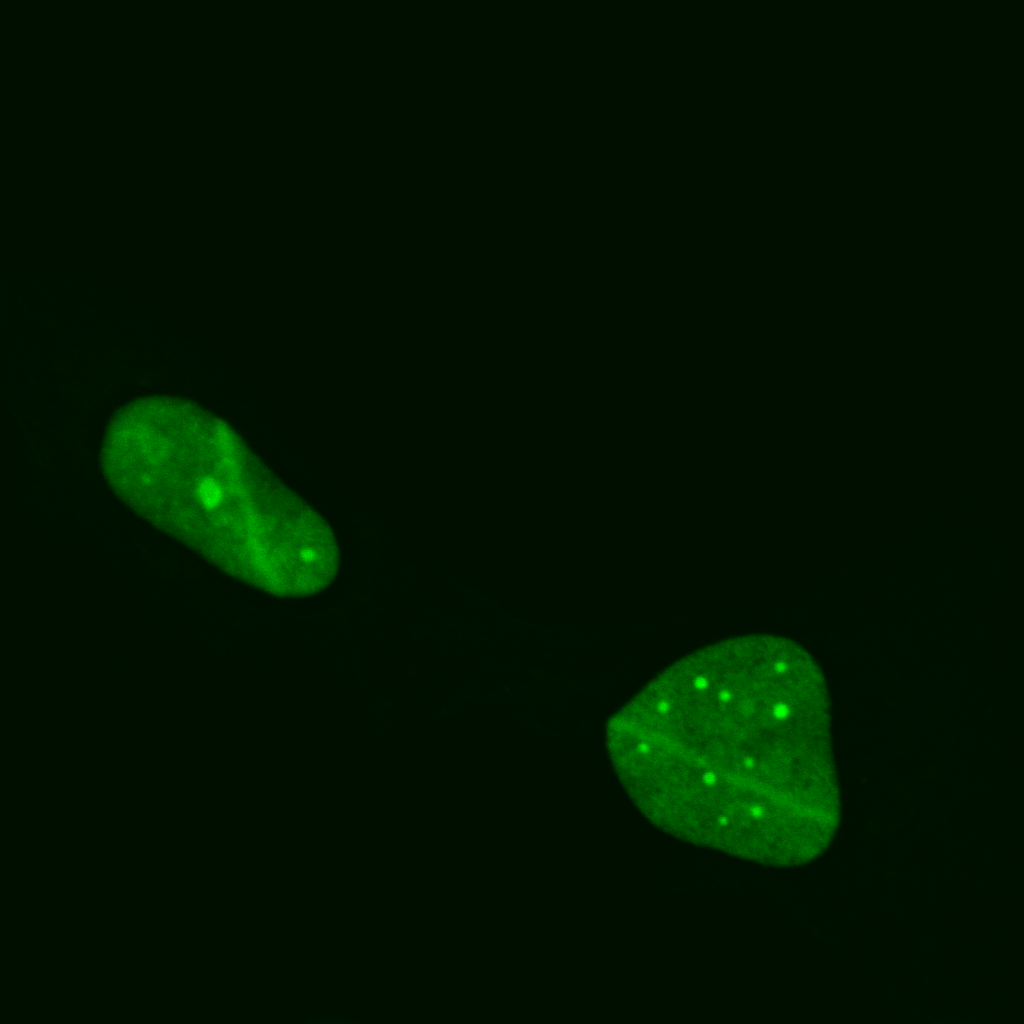

Supplement: Supplementary file 4 — Source data Fig. 2 [file 44319_2024_219_MOESM4_ESM.zip › Figure2/2A/GFP-SPIN1-1 min.tif]

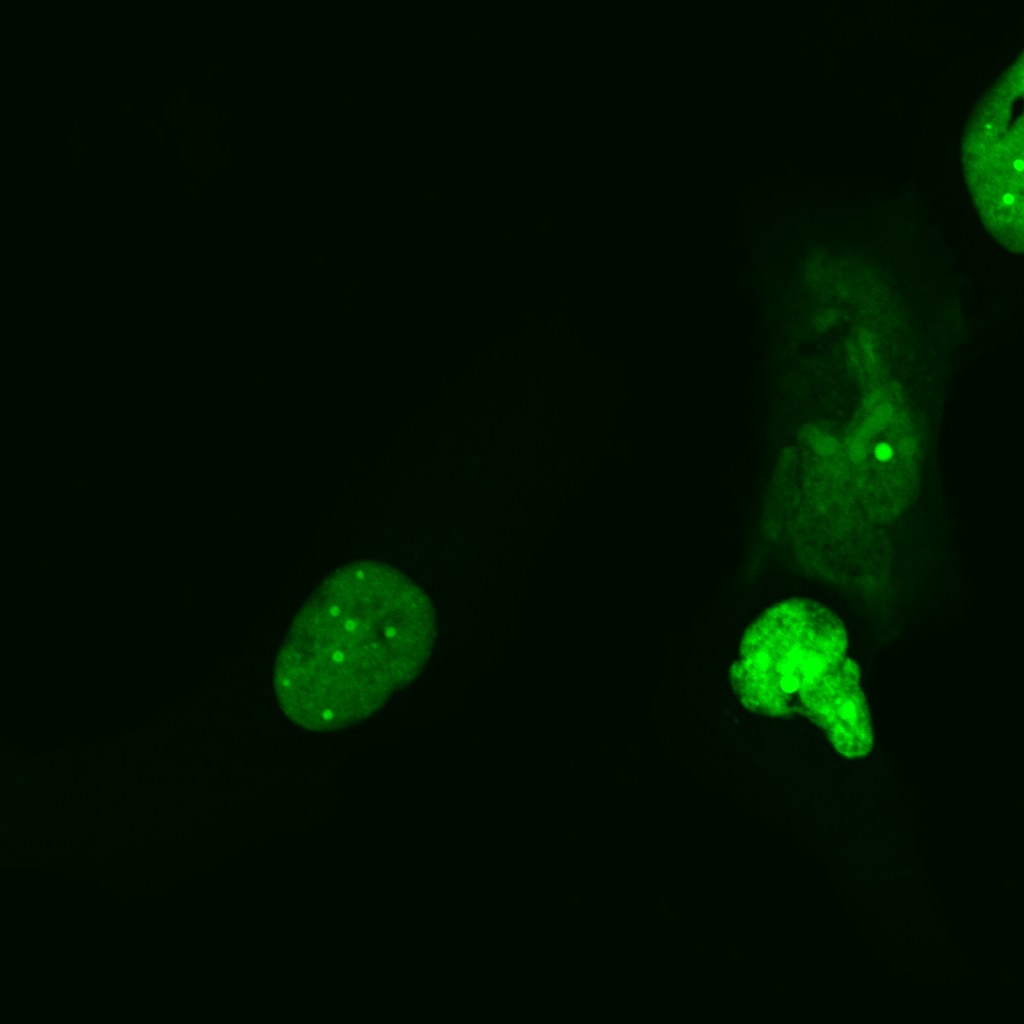

Supplement: Supplementary file 4 — Source data Fig. 2 [file 44319_2024_219_MOESM4_ESM.zip › Figure2/2A/GFP-SPIN1-10 min.tif]

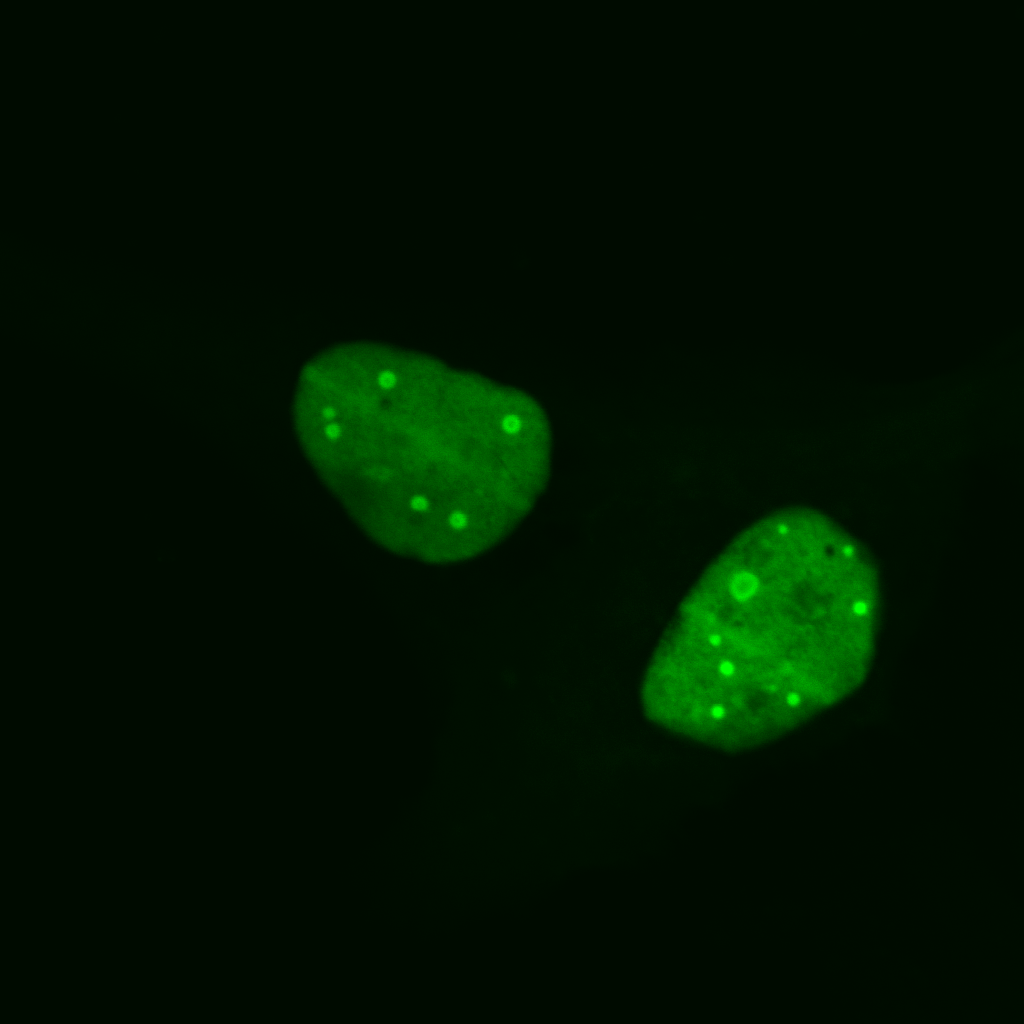

Supplement: Supplementary file 4 — Source data Fig. 2 [file 44319_2024_219_MOESM4_ESM.zip › Figure2/2A/GFP-SPIN1-3 min.tif]

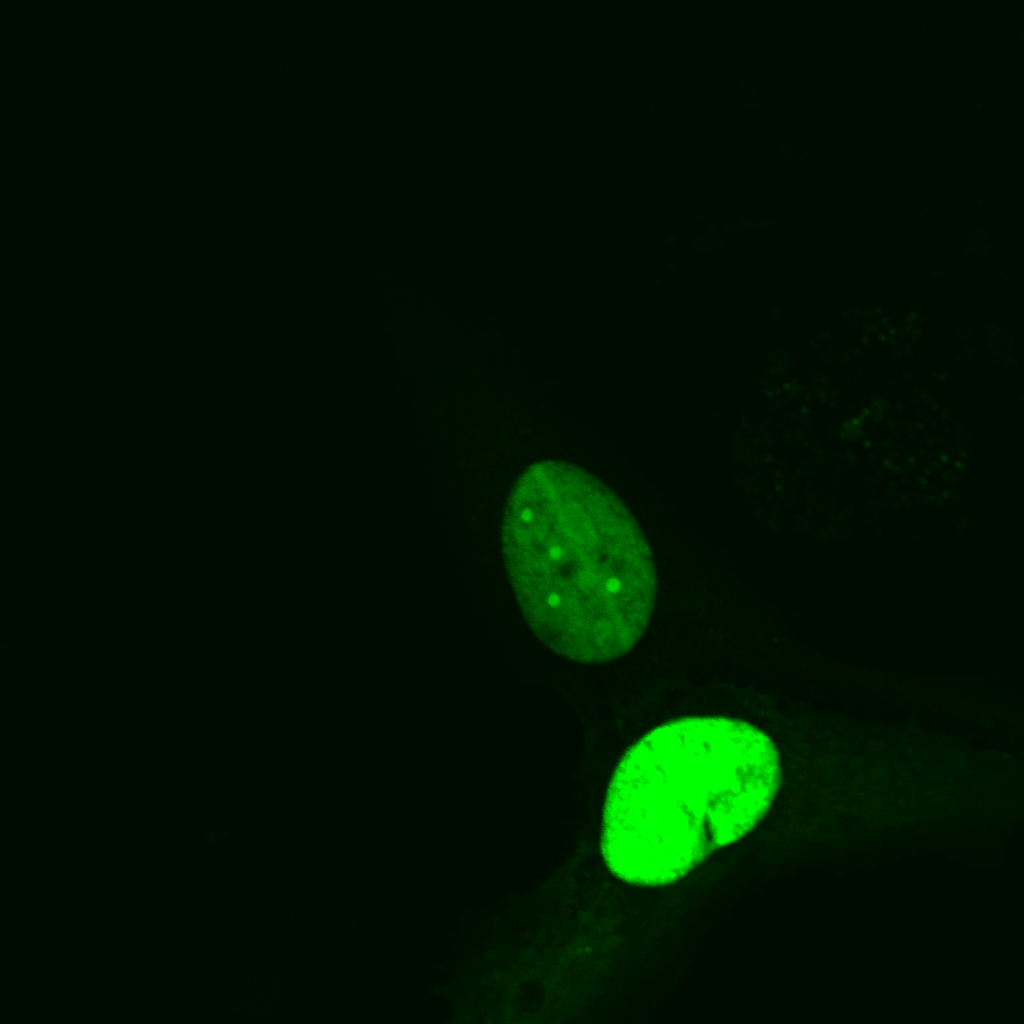

Supplement: Supplementary file 4 — Source data Fig. 2 [file 44319_2024_219_MOESM4_ESM.zip › Figure2/2A/GFP-SPIN1-30 sec.tif]

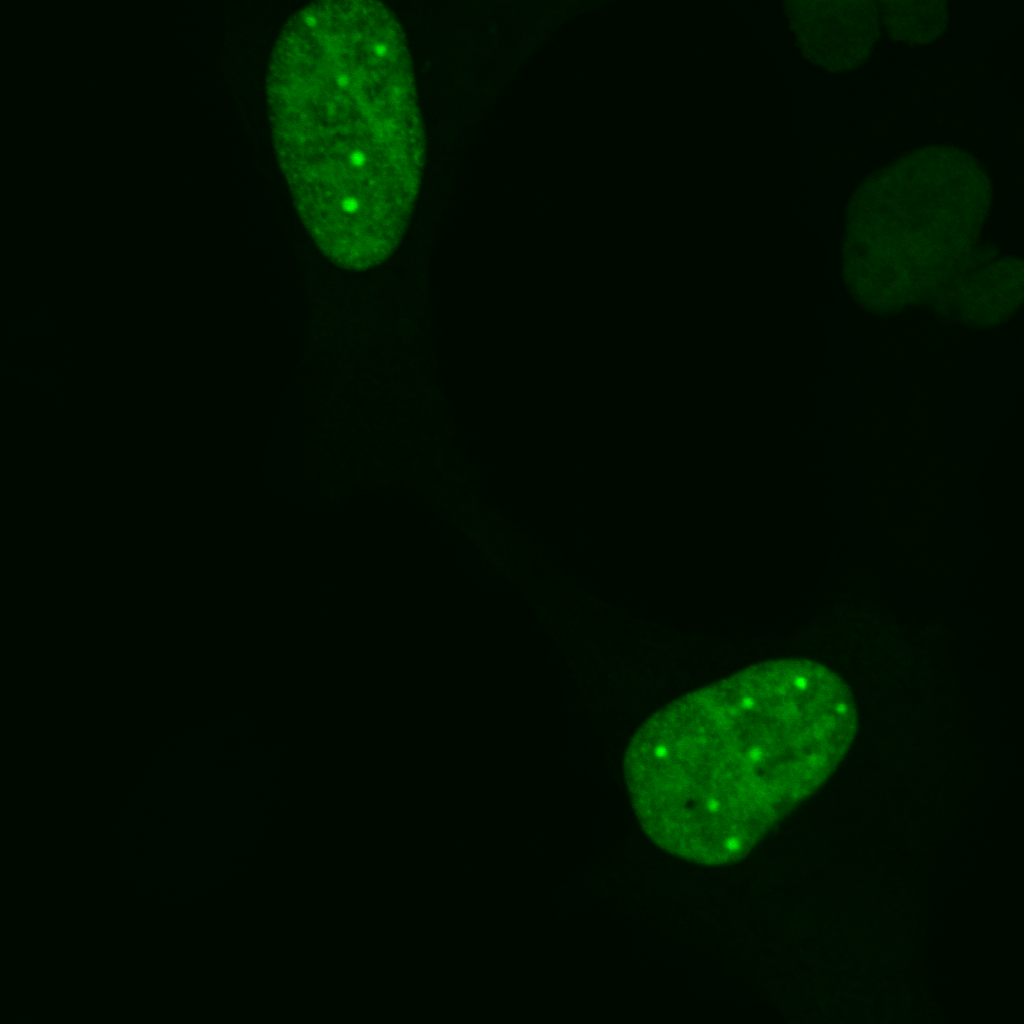

Supplement: Supplementary file 4 — Source data Fig. 2 [file 44319_2024_219_MOESM4_ESM.zip › Figure2/2A/GFP-SPIN1-5 min.tif]

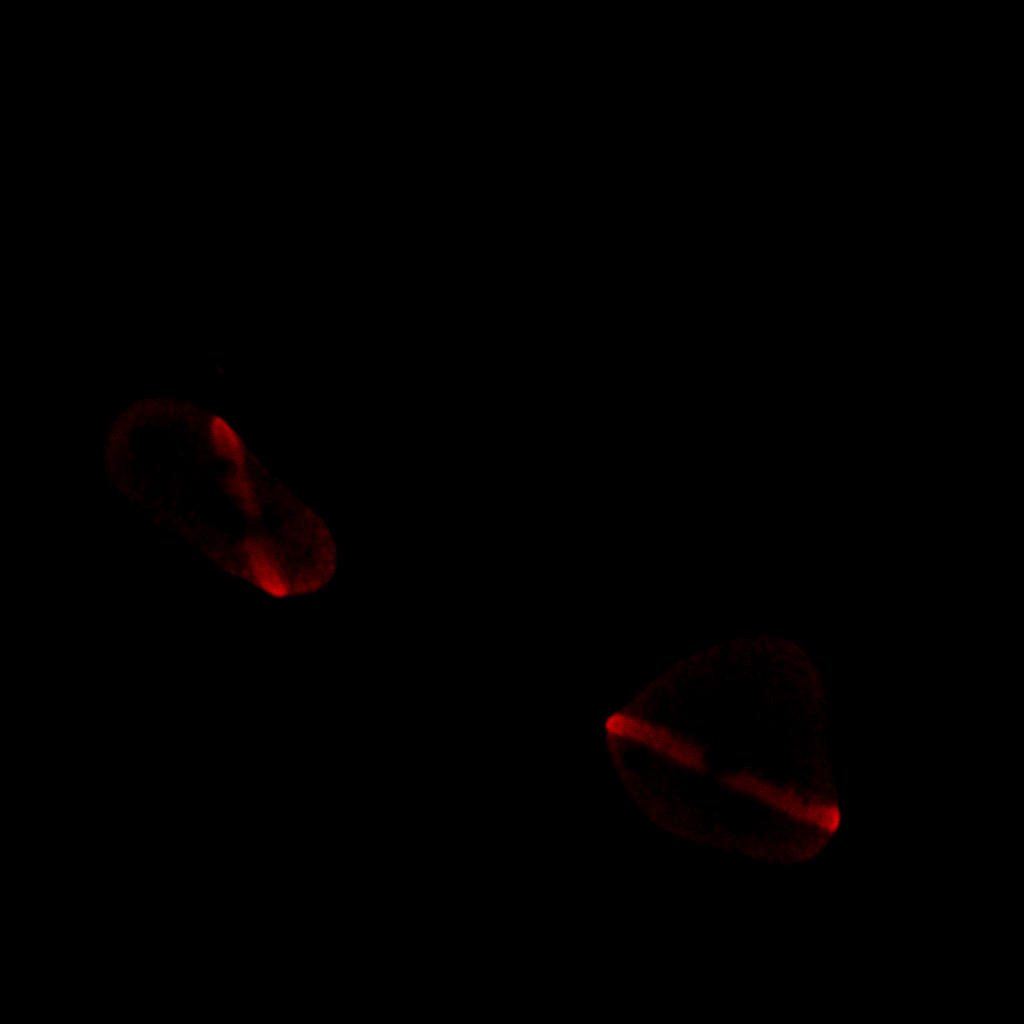

Supplement: Supplementary file 4 — Source data Fig. 2 [file 44319_2024_219_MOESM4_ESM.zip › Figure2/2A/PAR-1 min.tif]

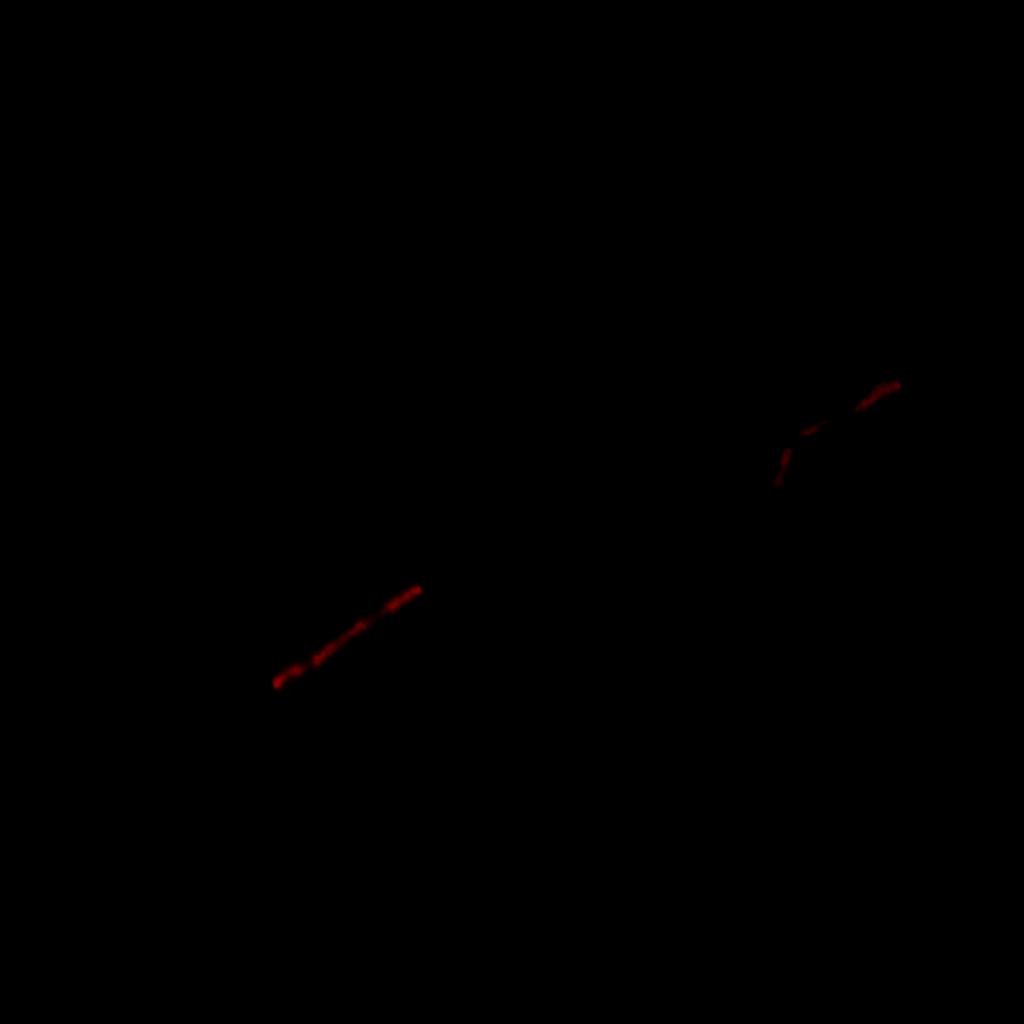

Supplement: Supplementary file 4 — Source data Fig. 2 [file 44319_2024_219_MOESM4_ESM.zip › Figure2/2A/PAR-10 min.tif]

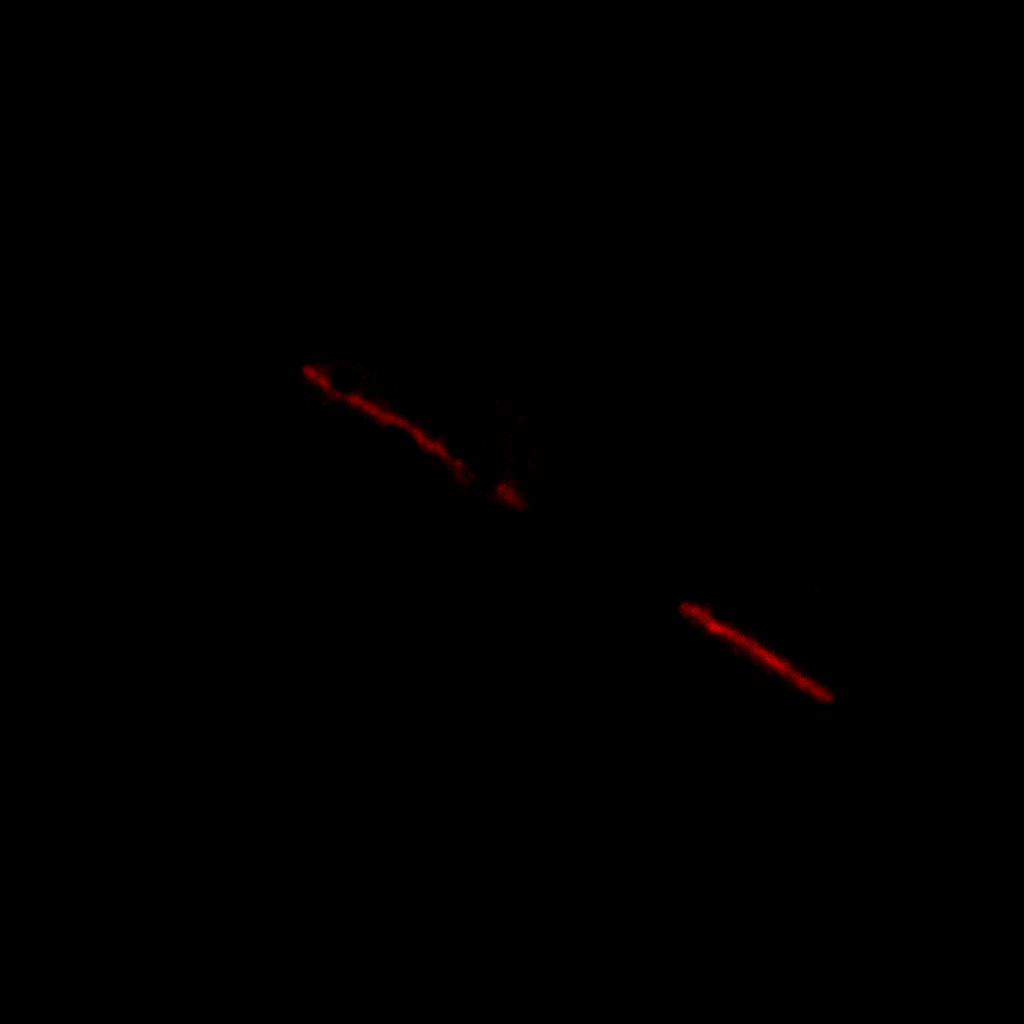

Supplement: Supplementary file 4 — Source data Fig. 2 [file 44319_2024_219_MOESM4_ESM.zip › Figure2/2A/PAR-3 min.tif]

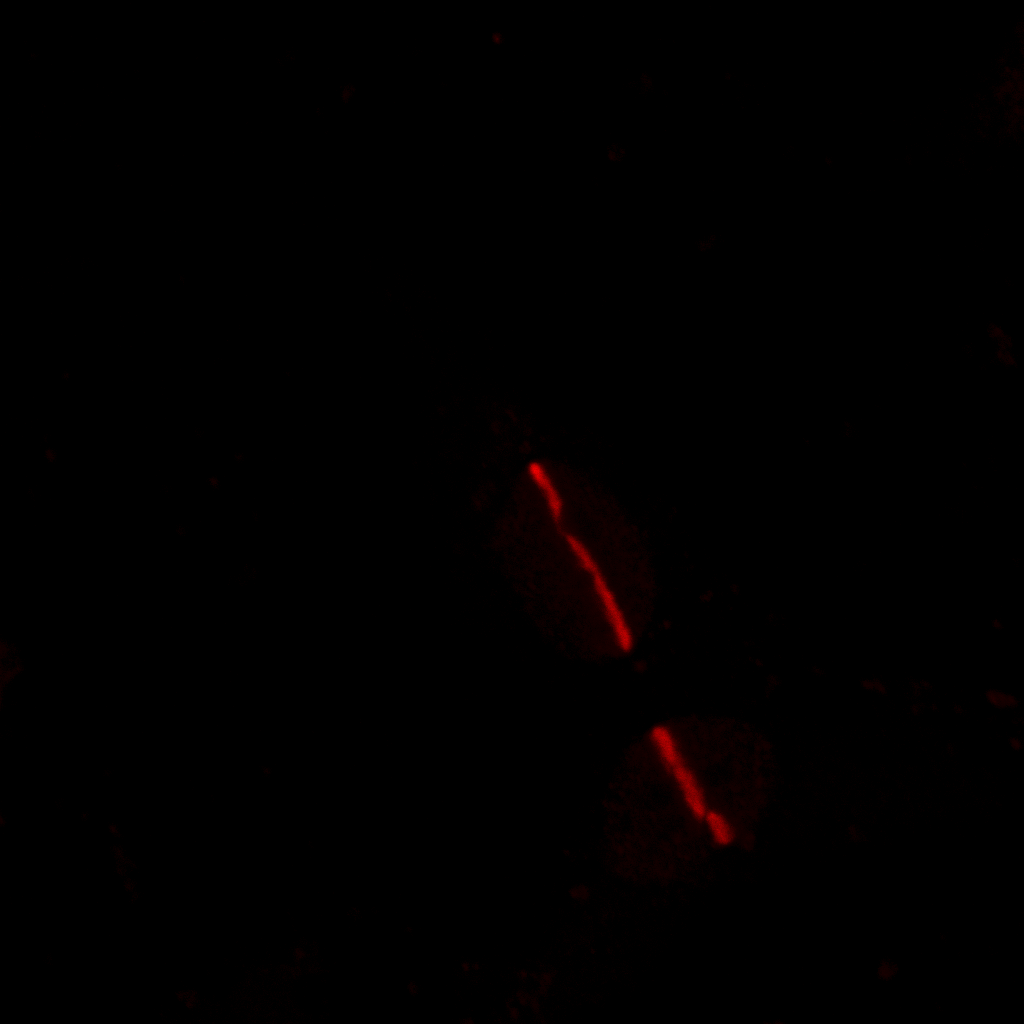

Supplement: Supplementary file 4 — Source data Fig. 2 [file 44319_2024_219_MOESM4_ESM.zip › Figure2/2A/PAR-30 sec.tif]

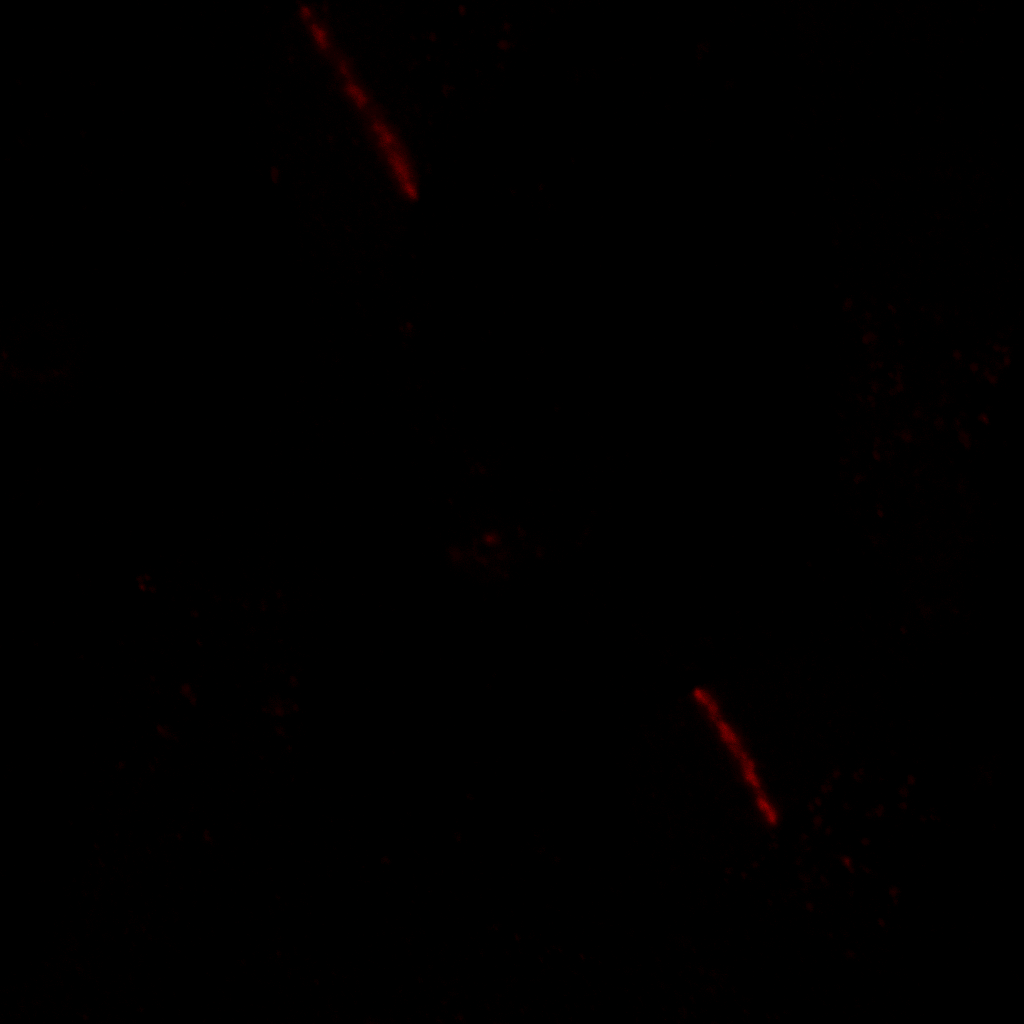

Supplement: Supplementary file 4 — Source data Fig. 2 [file 44319_2024_219_MOESM4_ESM.zip › Figure2/2A/PAR-5 min.tif]

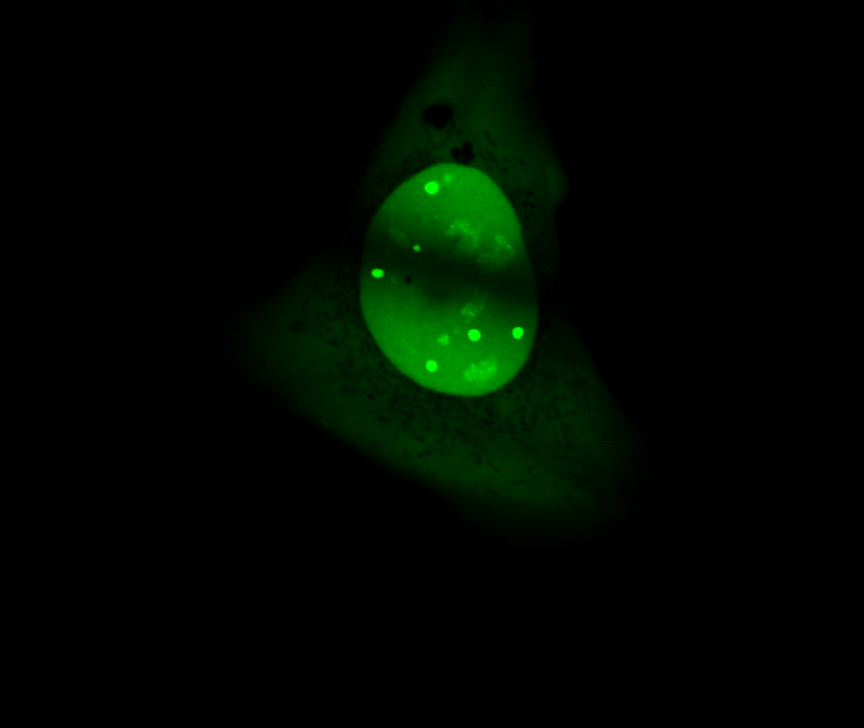

Supplement: Supplementary file 4 — Source data Fig. 2 [file 44319_2024_219_MOESM4_ESM.zip › Figure2/2B/Mock/GFP-SPIN1-0 sec.tif]

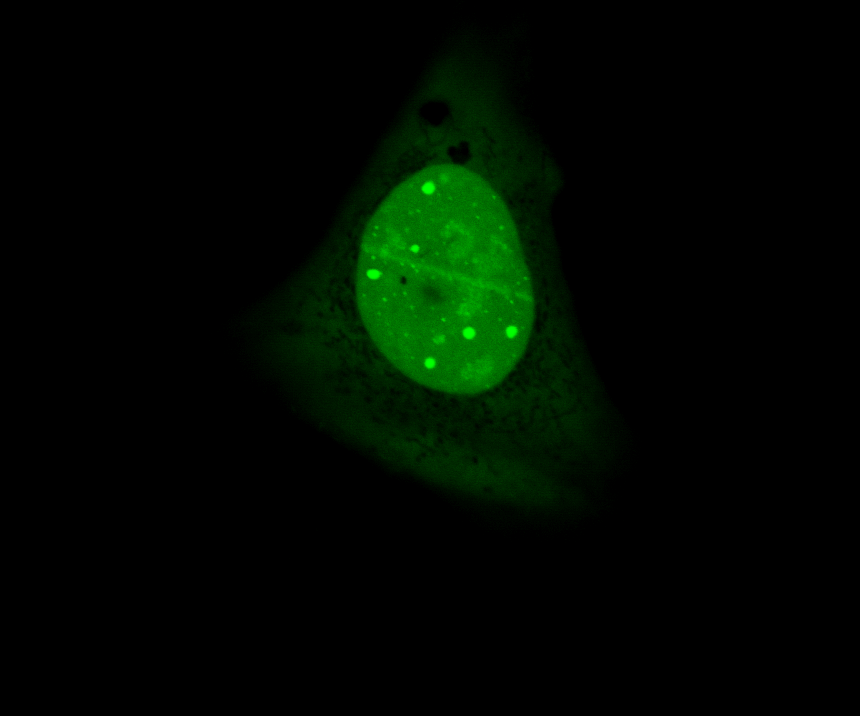

Supplement: Supplementary file 4 — Source data Fig. 2 [file 44319_2024_219_MOESM4_ESM.zip › Figure2/2B/Mock/GFP-SPIN1-1 min.tif]

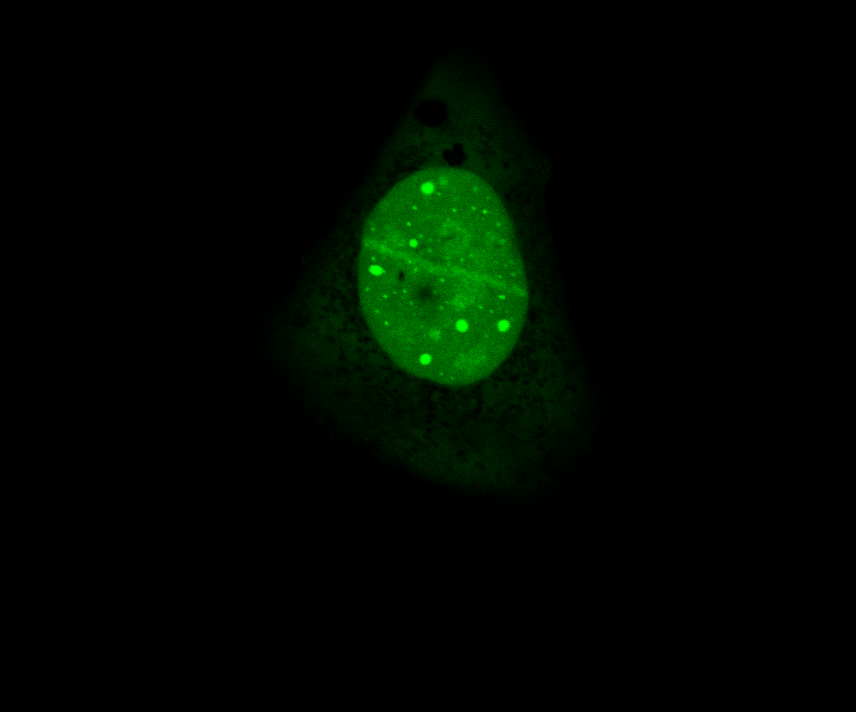

Supplement: Supplementary file 4 — Source data Fig. 2 [file 44319_2024_219_MOESM4_ESM.zip › Figure2/2B/Mock/GFP-SPIN1-10 min.tif]

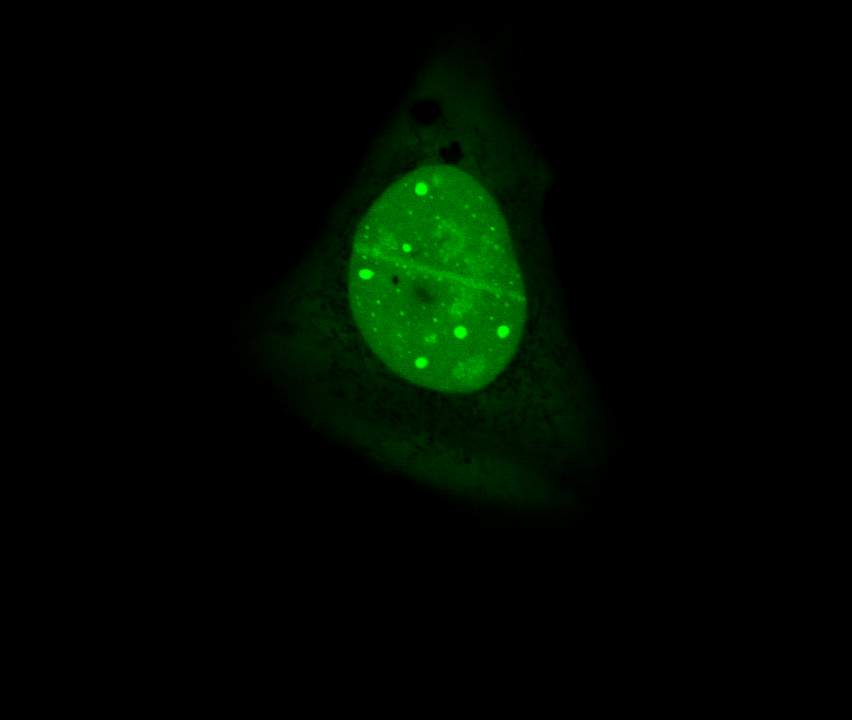

Supplement: Supplementary file 4 — Source data Fig. 2 [file 44319_2024_219_MOESM4_ESM.zip › Figure2/2B/Mock/GFP-SPIN1-3 min.tif]

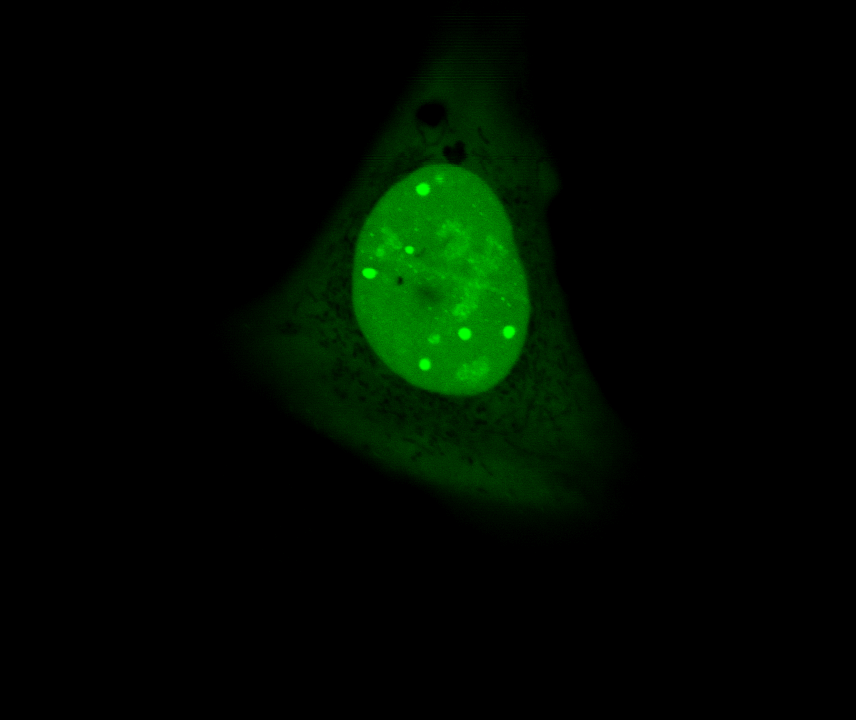

Supplement: Supplementary file 4 — Source data Fig. 2 [file 44319_2024_219_MOESM4_ESM.zip › Figure2/2B/Mock/GFP-SPIN1-30 sec.tif]

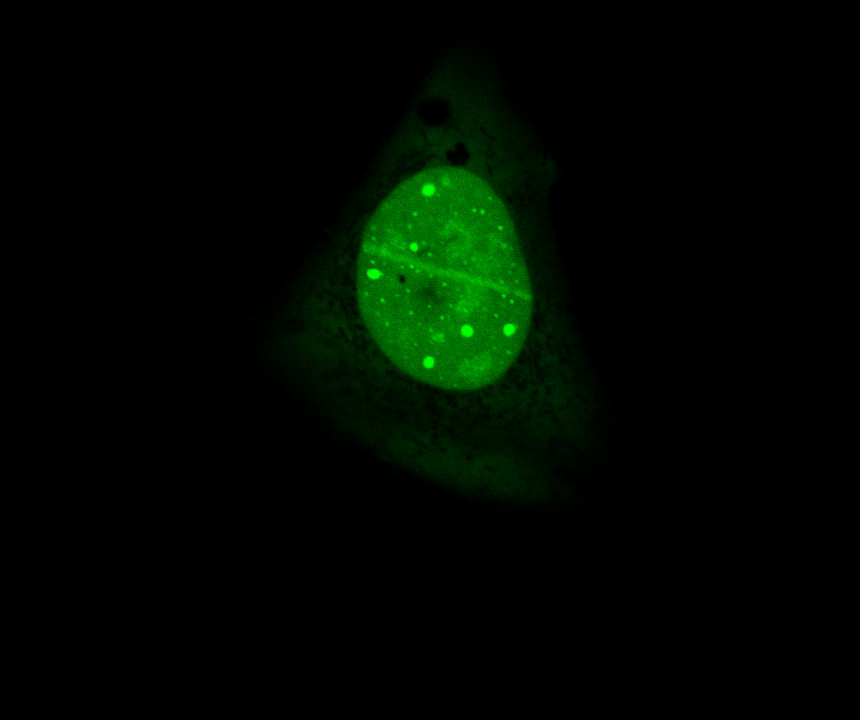

Supplement: Supplementary file 4 — Source data Fig. 2 [file 44319_2024_219_MOESM4_ESM.zip › Figure2/2B/Mock/GFP-SPIN1-5 min.tif]

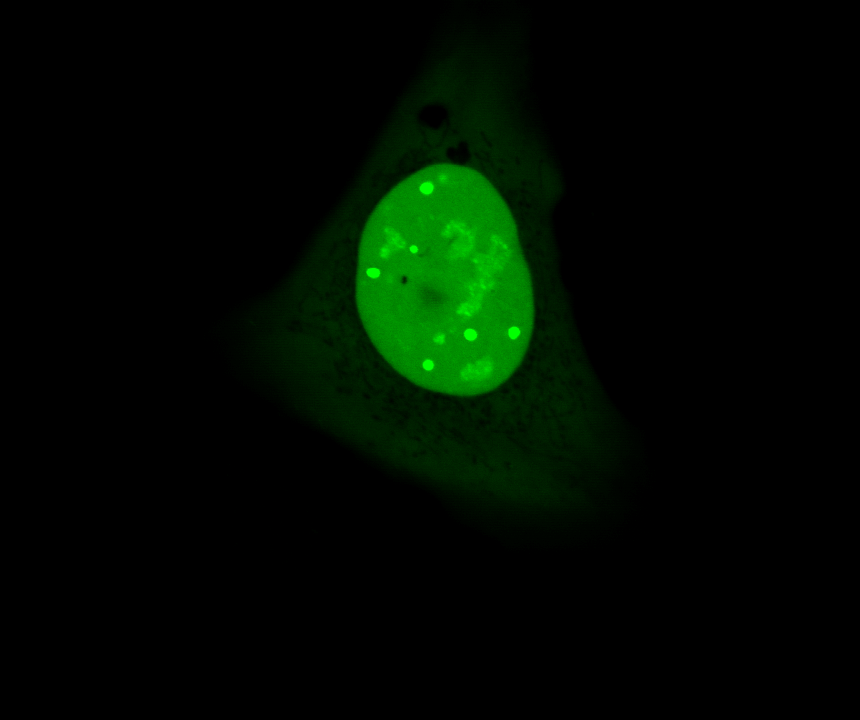

Supplement: Supplementary file 4 — Source data Fig. 2 [file 44319_2024_219_MOESM4_ESM.zip › Figure2/2B/Mock/GFP-SPIN1-before.tif]

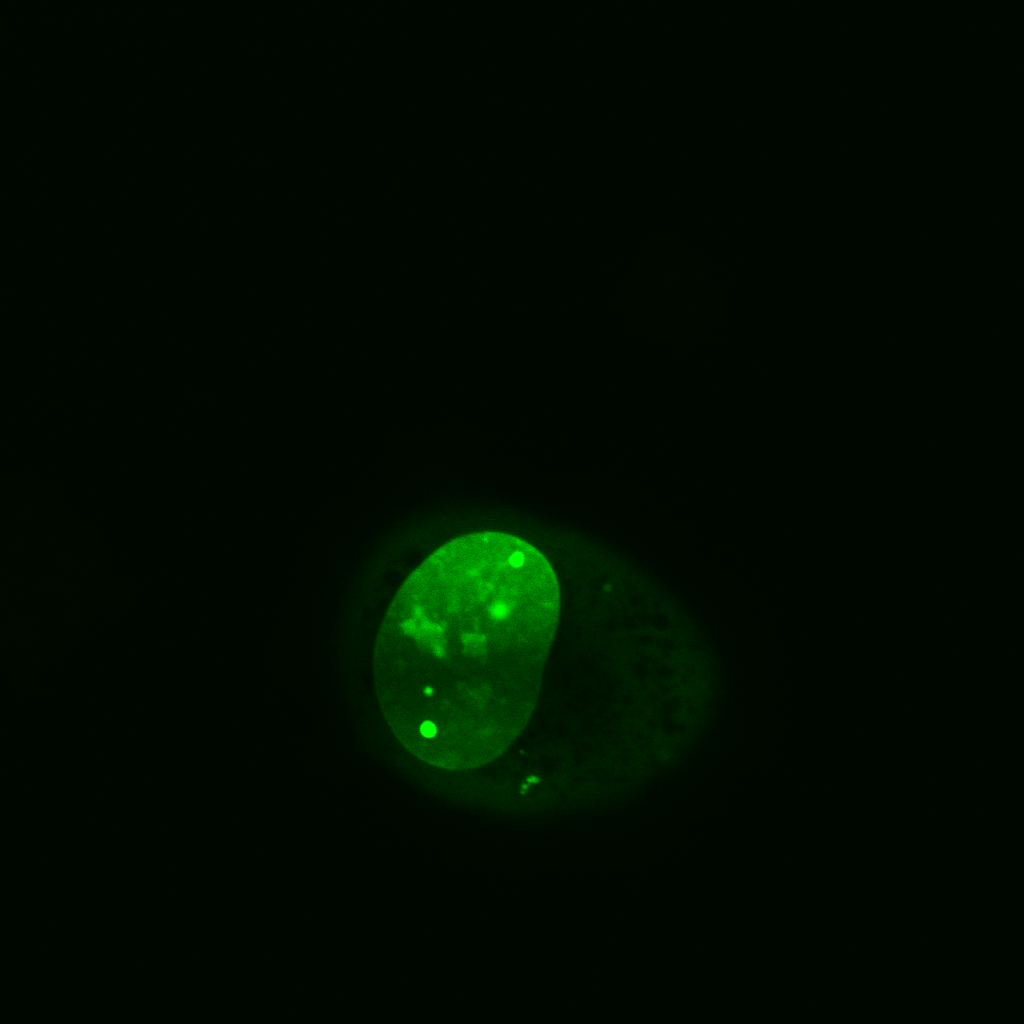

Supplement: Supplementary file 4 — Source data Fig. 2 [file 44319_2024_219_MOESM4_ESM.zip › Figure2/2B/Olaparib/Olaparib-0 sec.tif]

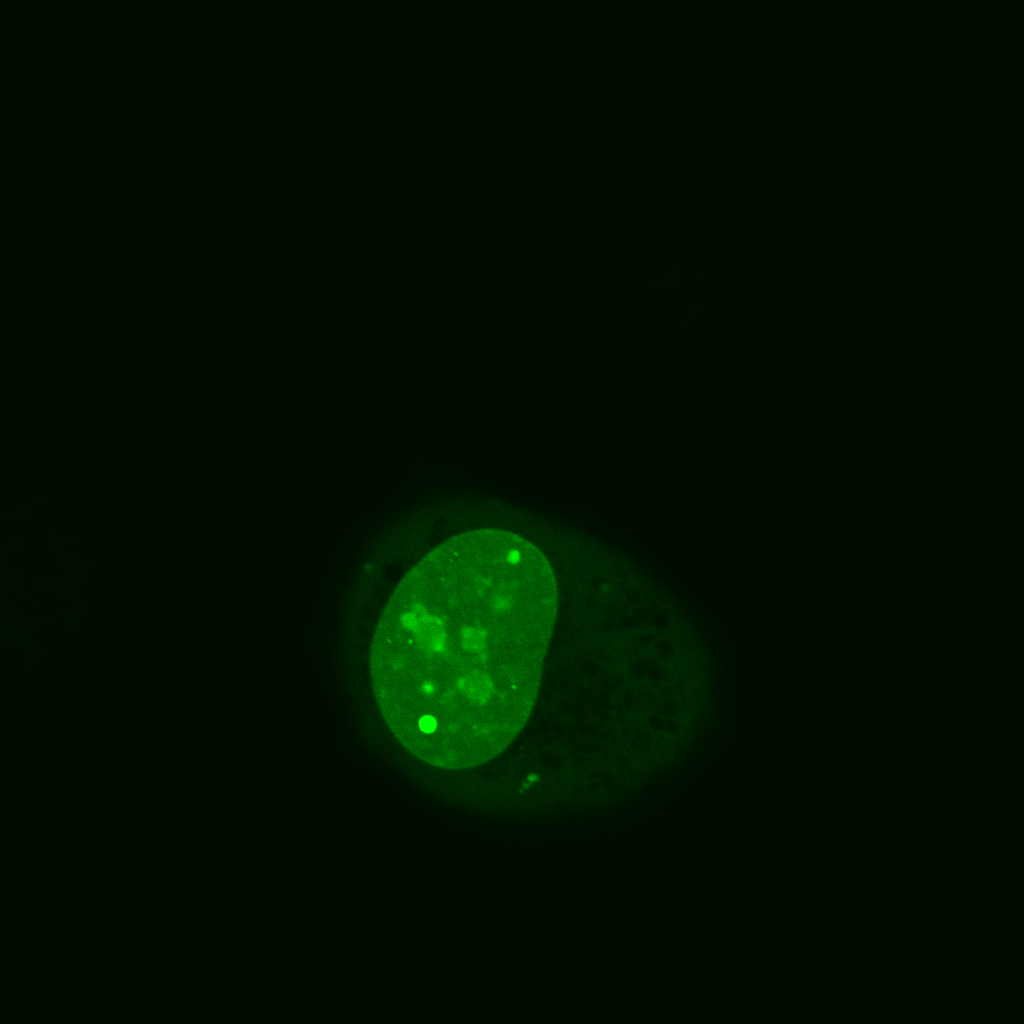

Supplement: Supplementary file 4 — Source data Fig. 2 [file 44319_2024_219_MOESM4_ESM.zip › Figure2/2B/Olaparib/Olaparib-1 min.tif]

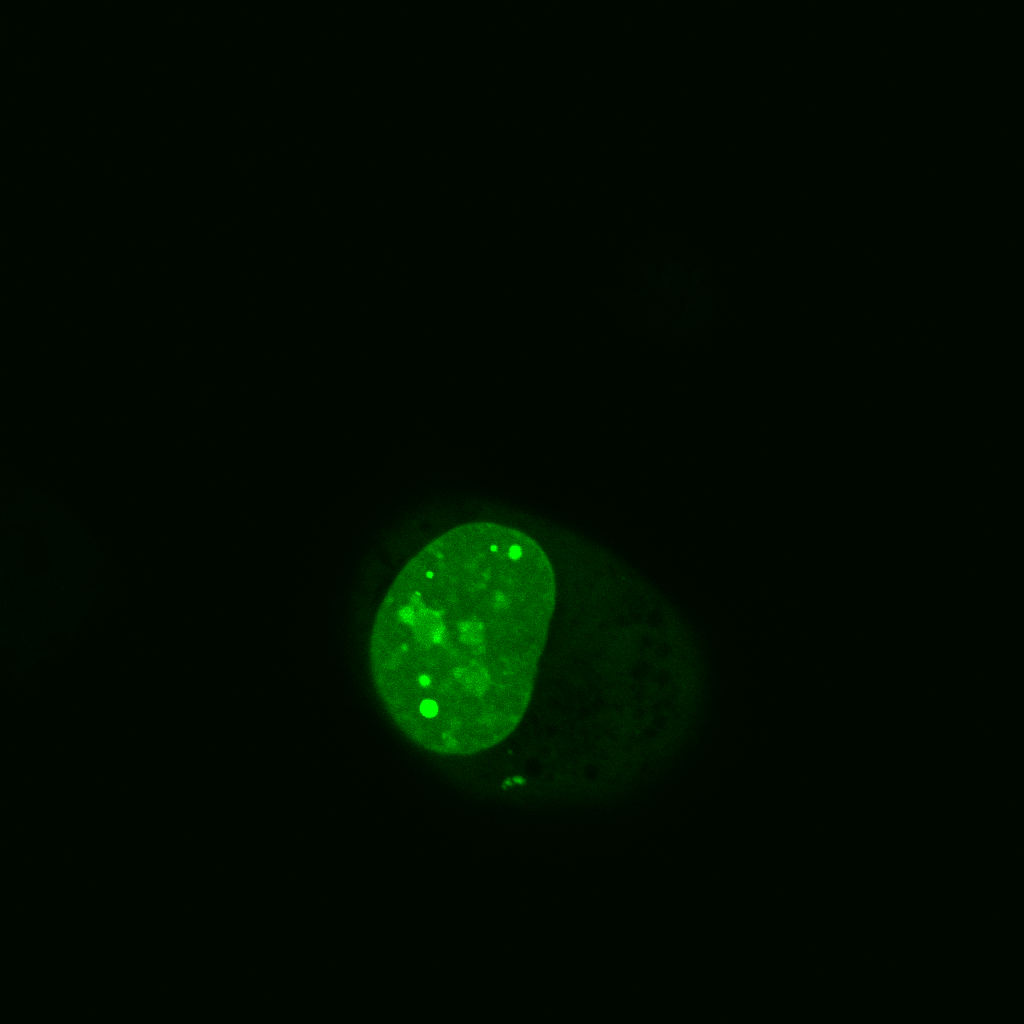

Supplement: Supplementary file 4 — Source data Fig. 2 [file 44319_2024_219_MOESM4_ESM.zip › Figure2/2B/Olaparib/Olaparib-10 min.tif]

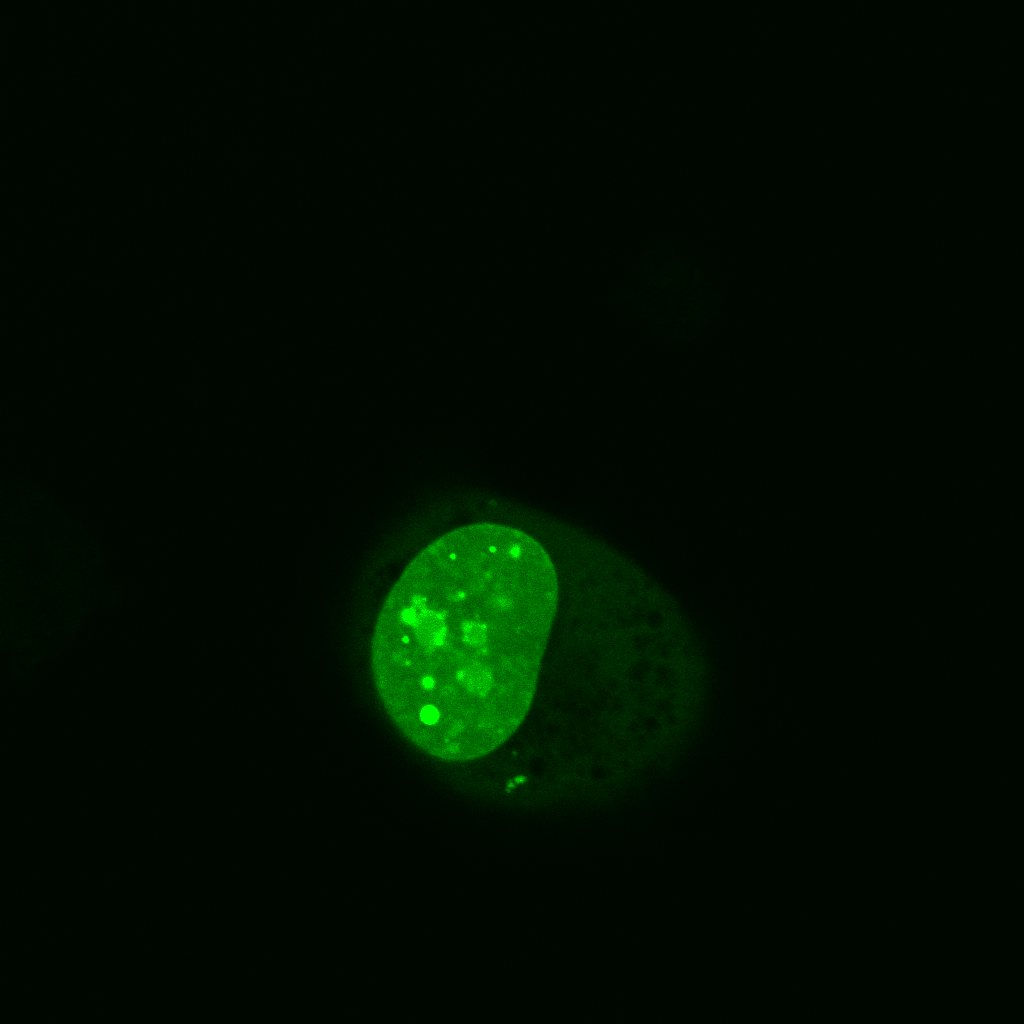

Supplement: Supplementary file 4 — Source data Fig. 2 [file 44319_2024_219_MOESM4_ESM.zip › Figure2/2B/Olaparib/Olaparib-3 min.tif]

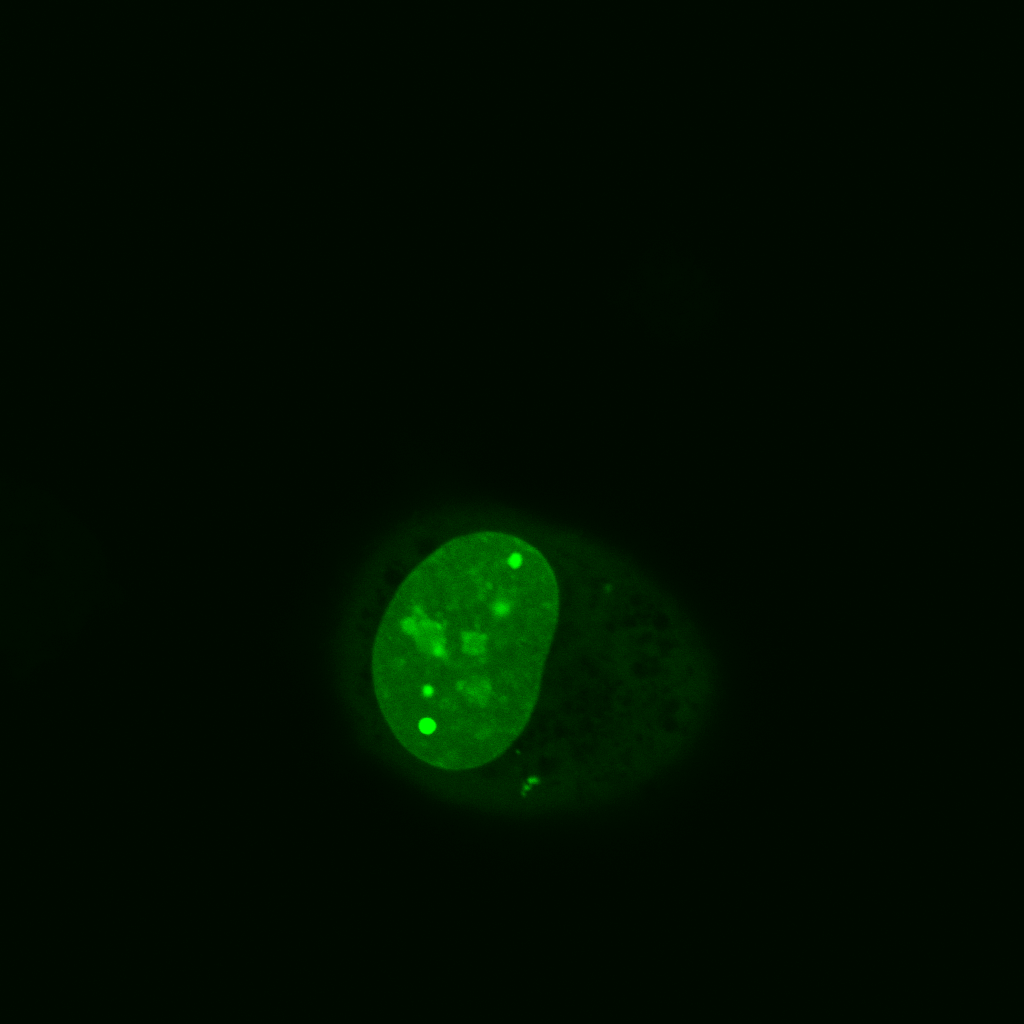

Supplement: Supplementary file 4 — Source data Fig. 2 [file 44319_2024_219_MOESM4_ESM.zip › Figure2/2B/Olaparib/Olaparib-30 sec.tif]

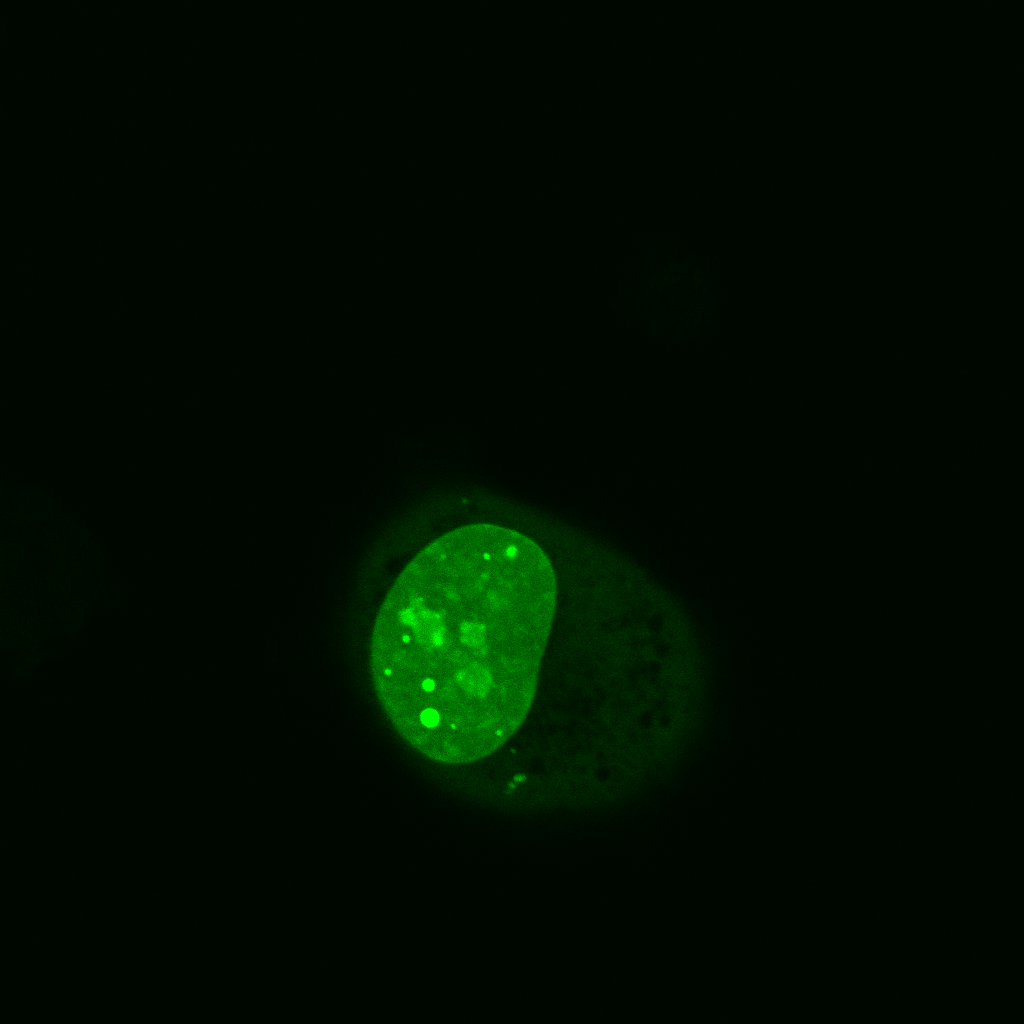

Supplement: Supplementary file 4 — Source data Fig. 2 [file 44319_2024_219_MOESM4_ESM.zip › Figure2/2B/Olaparib/Olaparib-5 min.tif]

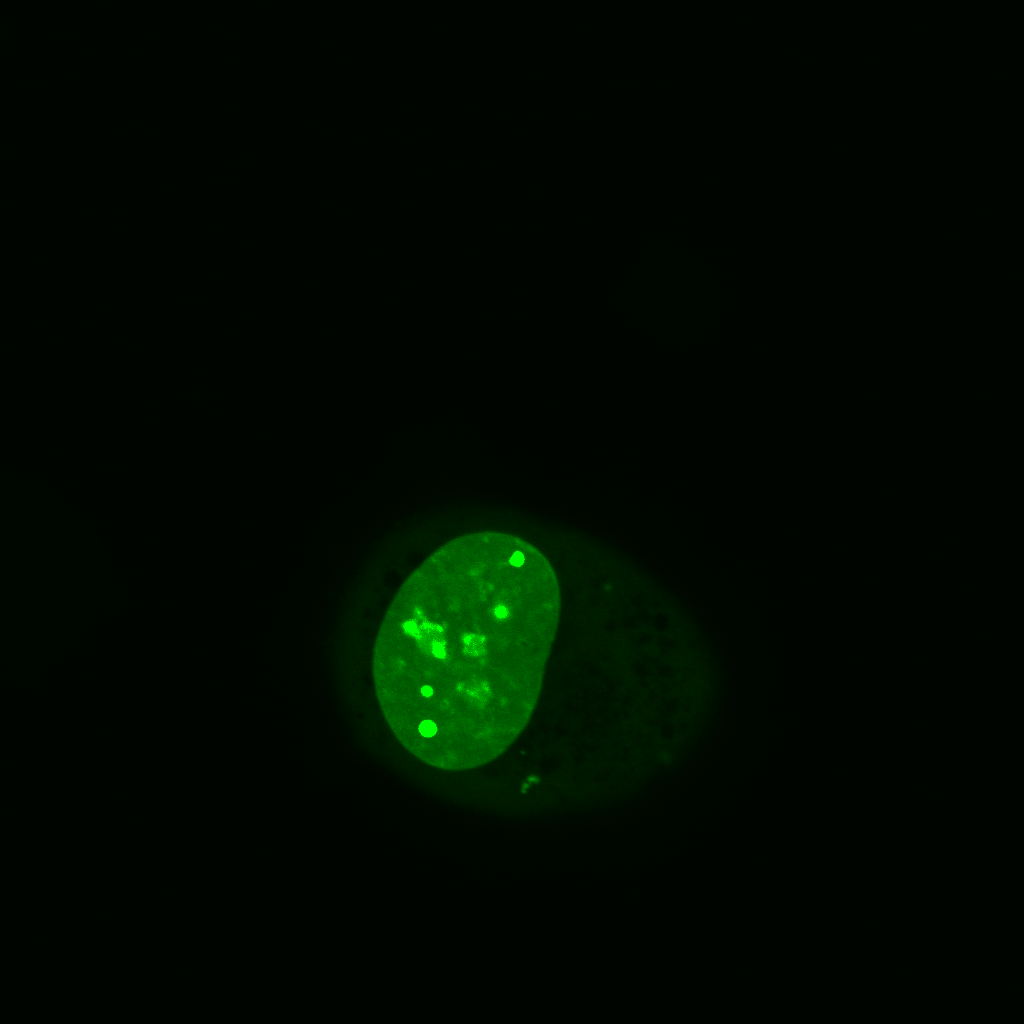

Supplement: Supplementary file 4 — Source data Fig. 2 [file 44319_2024_219_MOESM4_ESM.zip › Figure2/2B/Olaparib/Olaparib-before.tif]

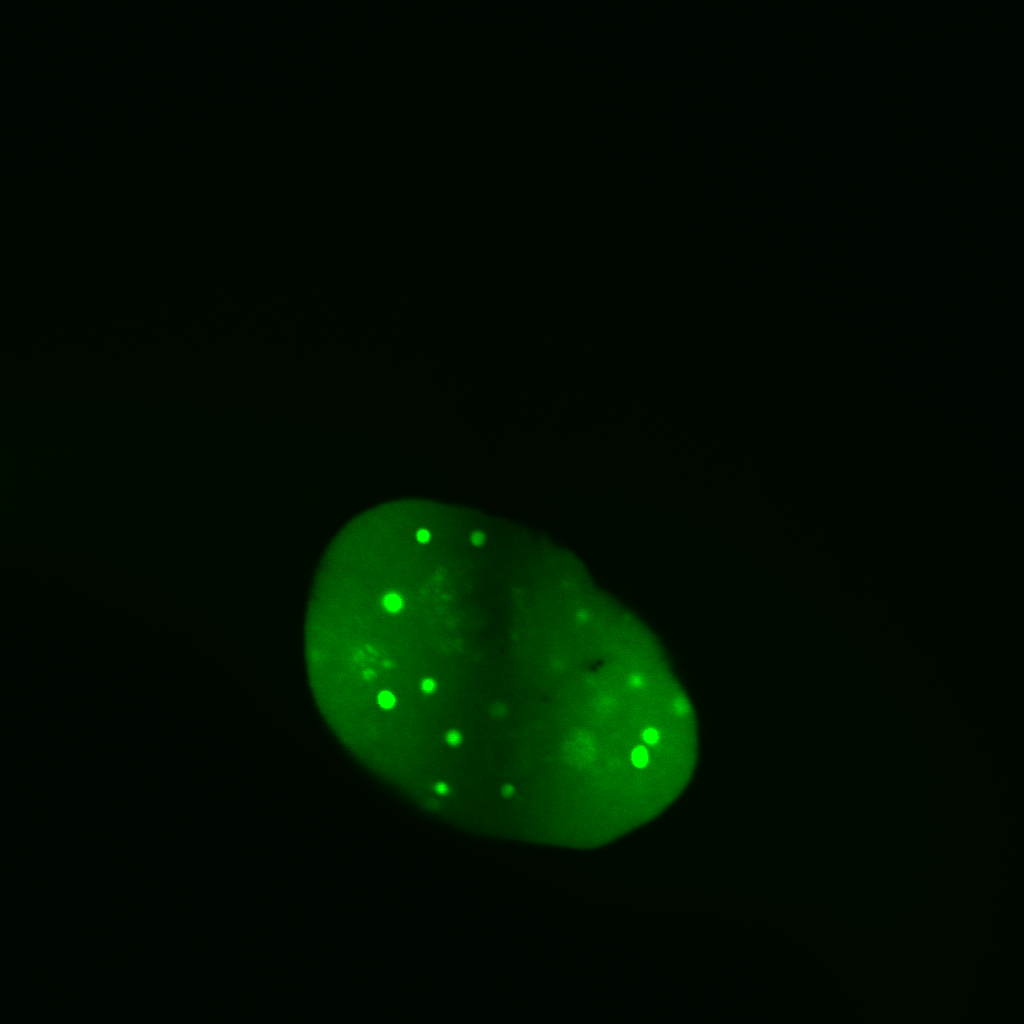

Supplement: Supplementary file 4 — Source data Fig. 2 [file 44319_2024_219_MOESM4_ESM.zip › Figure2/2B/PARP1-/PARP1-0 sec.tif]

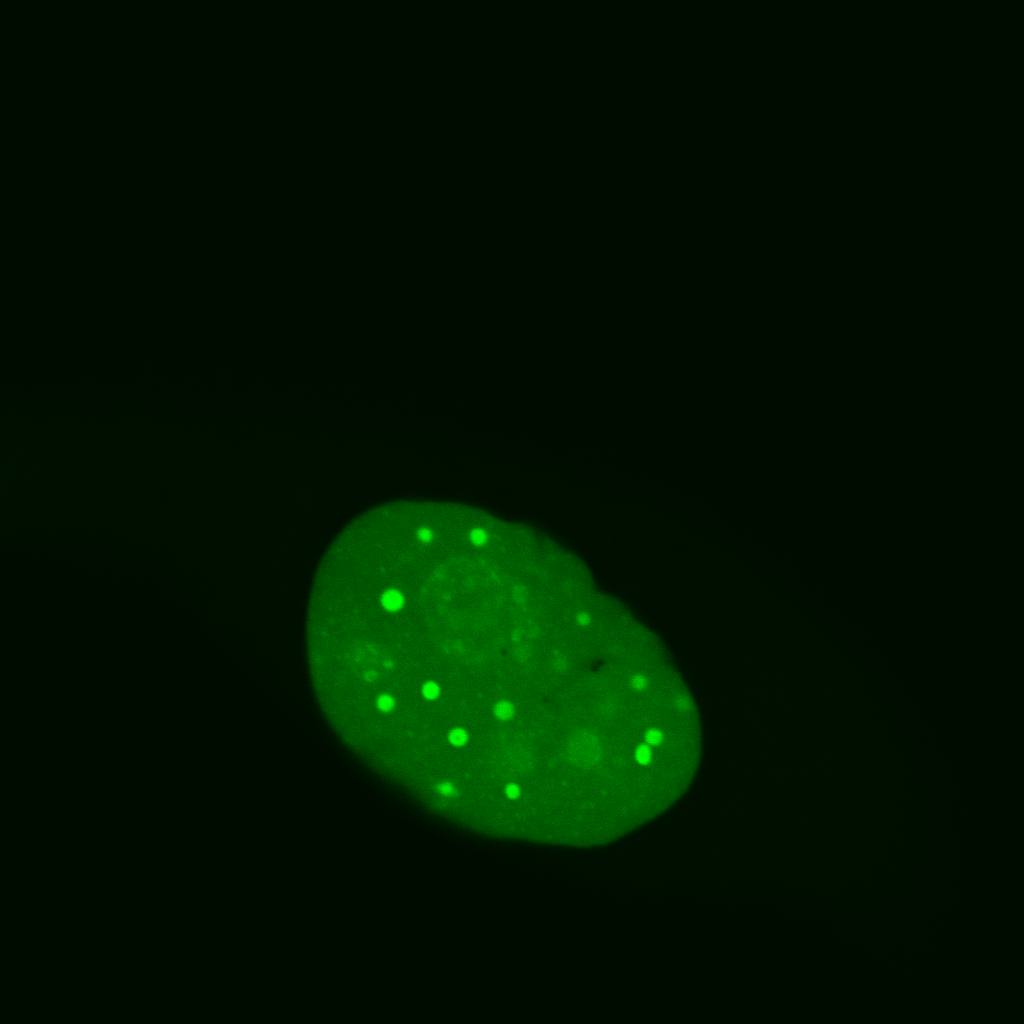

Supplement: Supplementary file 4 — Source data Fig. 2 [file 44319_2024_219_MOESM4_ESM.zip › Figure2/2B/PARP1-/PARP1-1 min.tif]

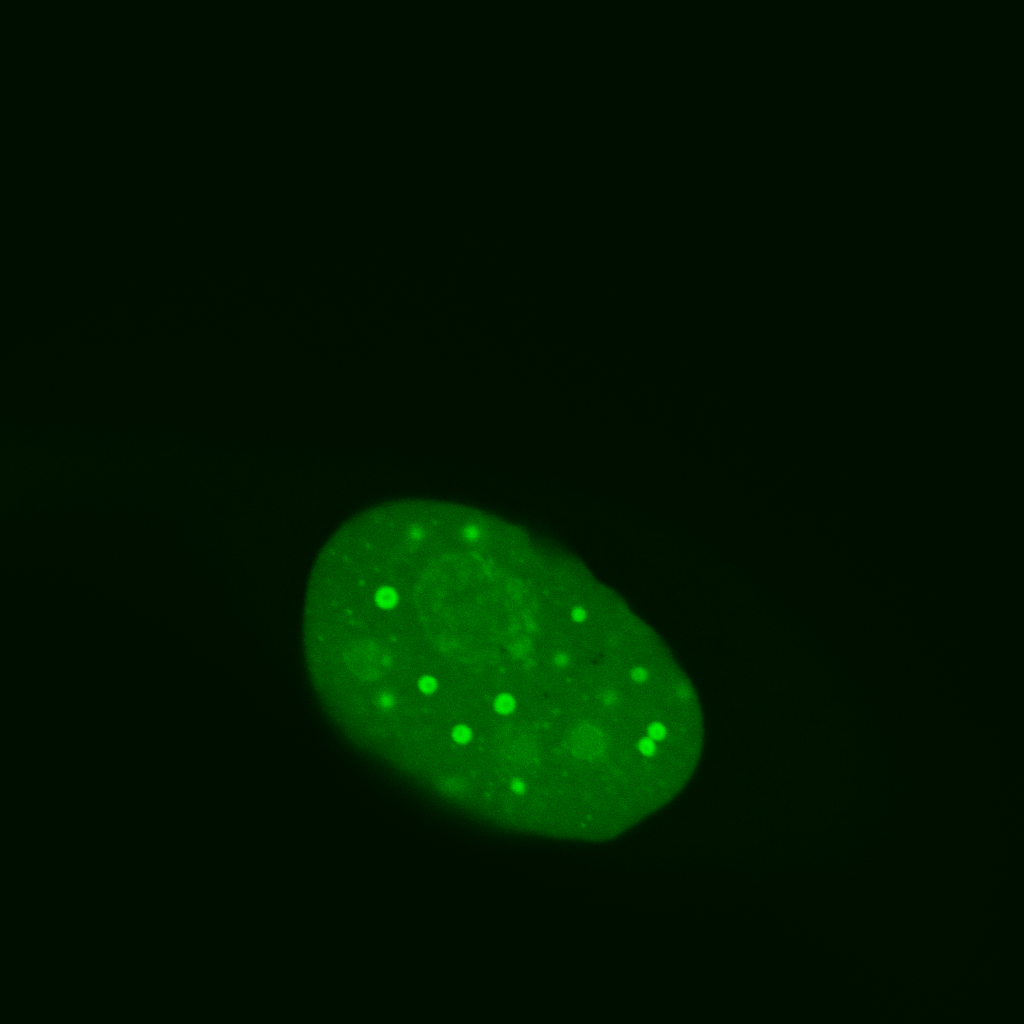

Supplement: Supplementary file 4 — Source data Fig. 2 [file 44319_2024_219_MOESM4_ESM.zip › Figure2/2B/PARP1-/PARP1-10 min.tif]

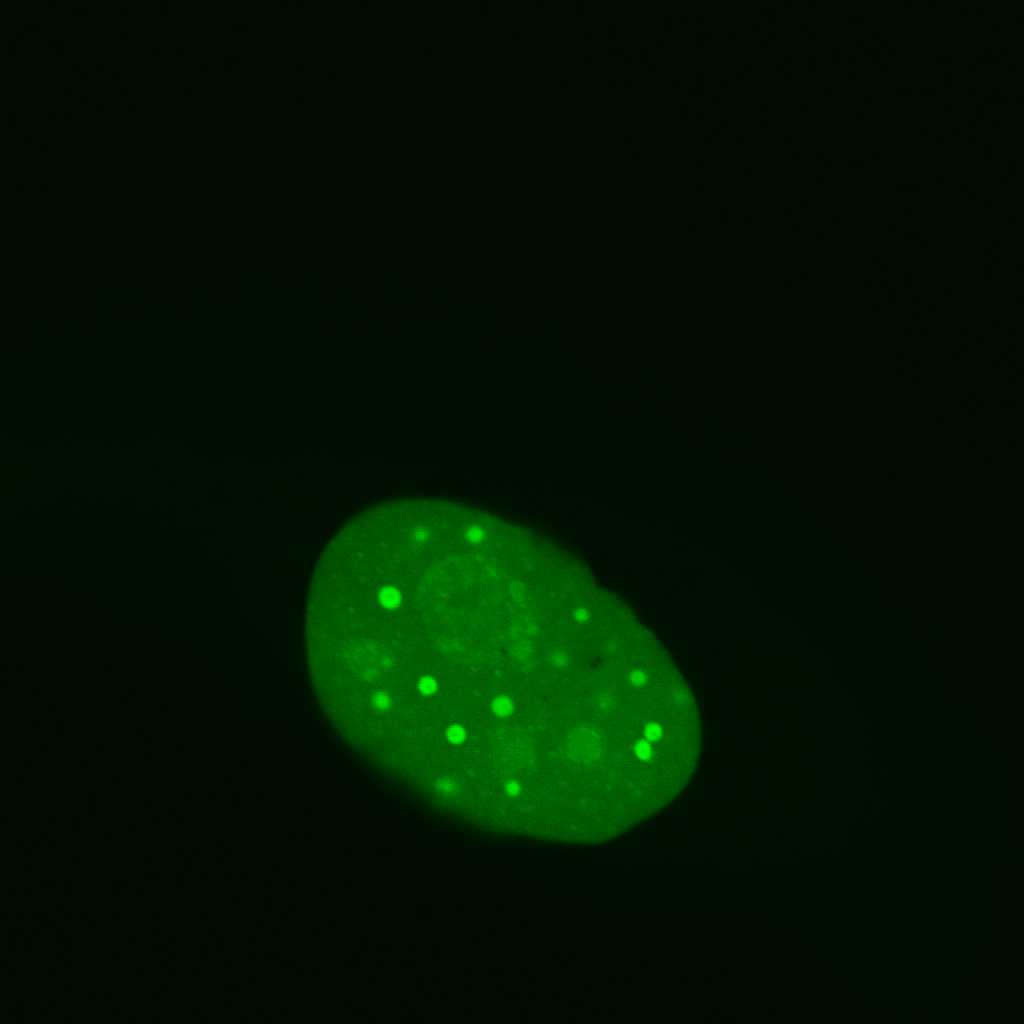

Supplement: Supplementary file 4 — Source data Fig. 2 [file 44319_2024_219_MOESM4_ESM.zip › Figure2/2B/PARP1-/PARP1-3 min.tif]

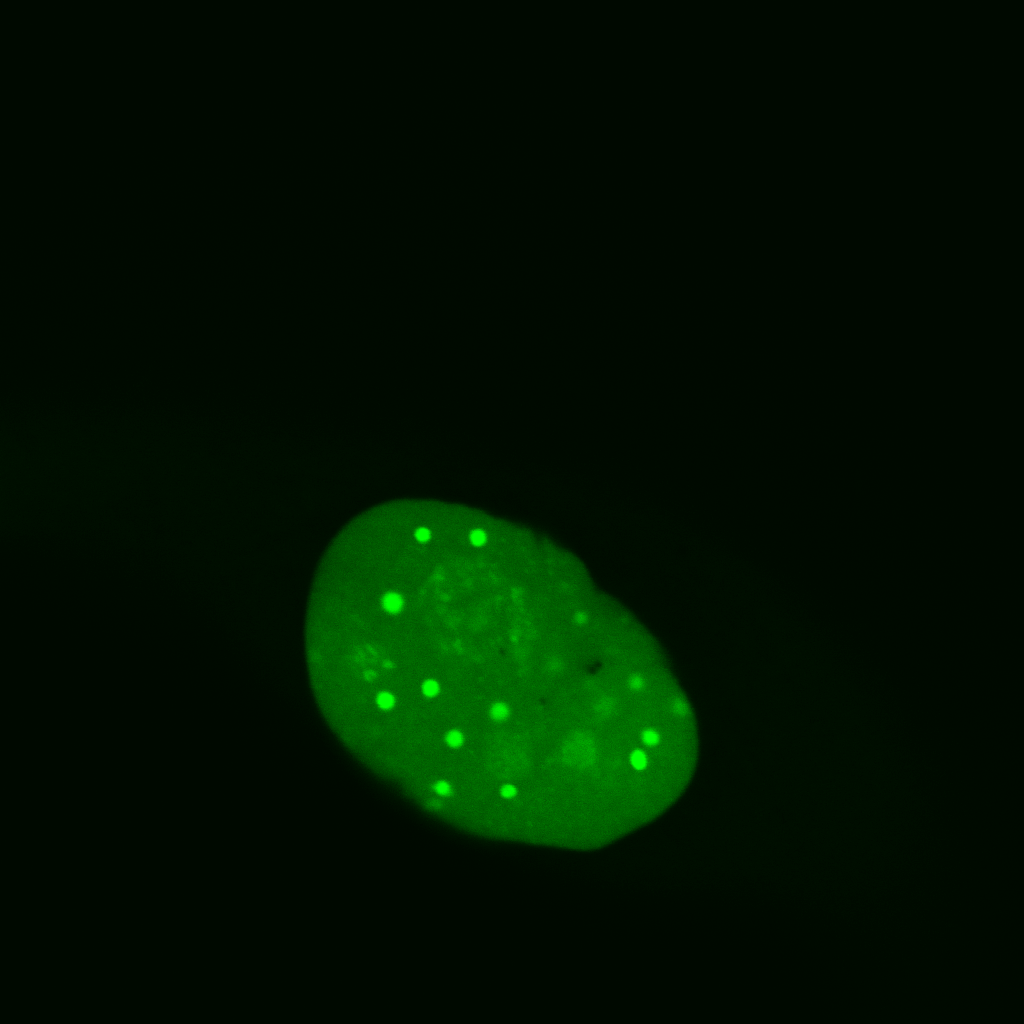

Supplement: Supplementary file 4 — Source data Fig. 2 [file 44319_2024_219_MOESM4_ESM.zip › Figure2/2B/PARP1-/PARP1-30 sec.tif]

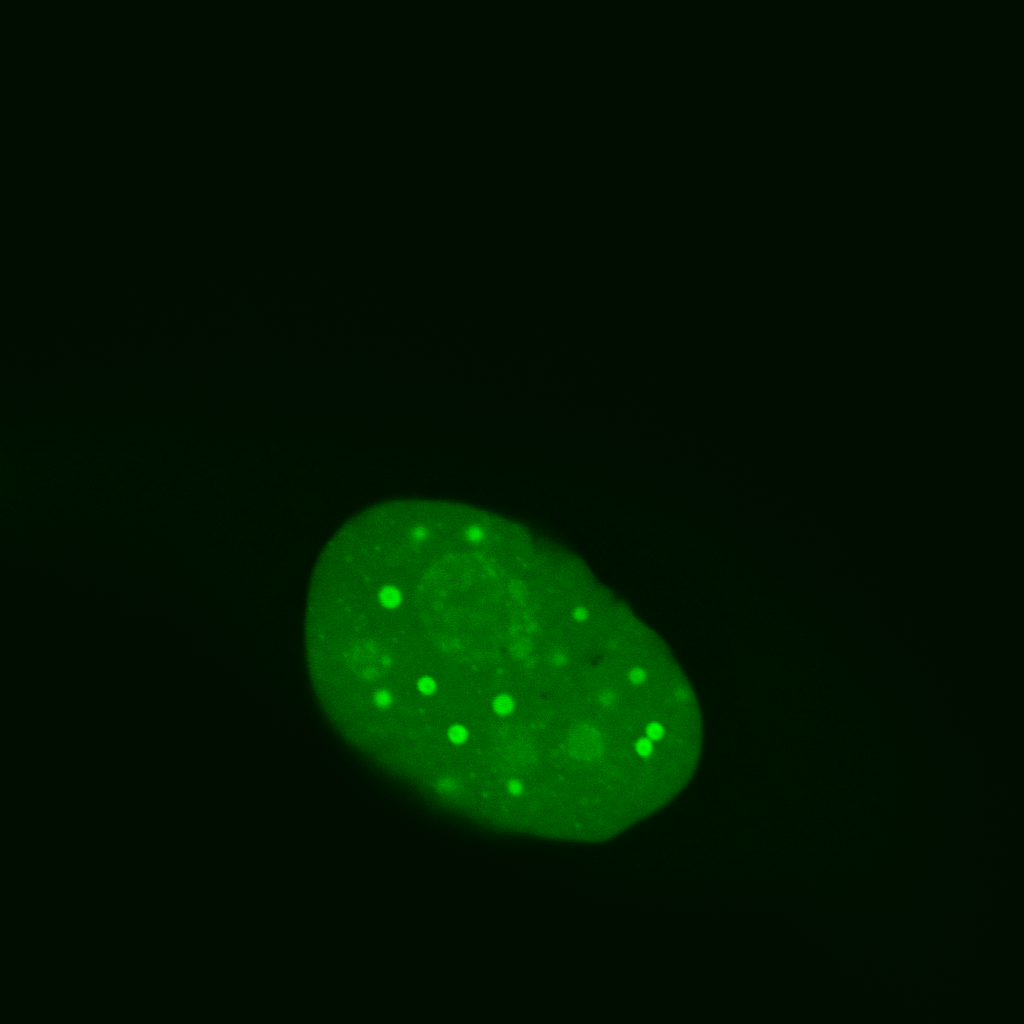

Supplement: Supplementary file 4 — Source data Fig. 2 [file 44319_2024_219_MOESM4_ESM.zip › Figure2/2B/PARP1-/PARP1-5 min.tif]

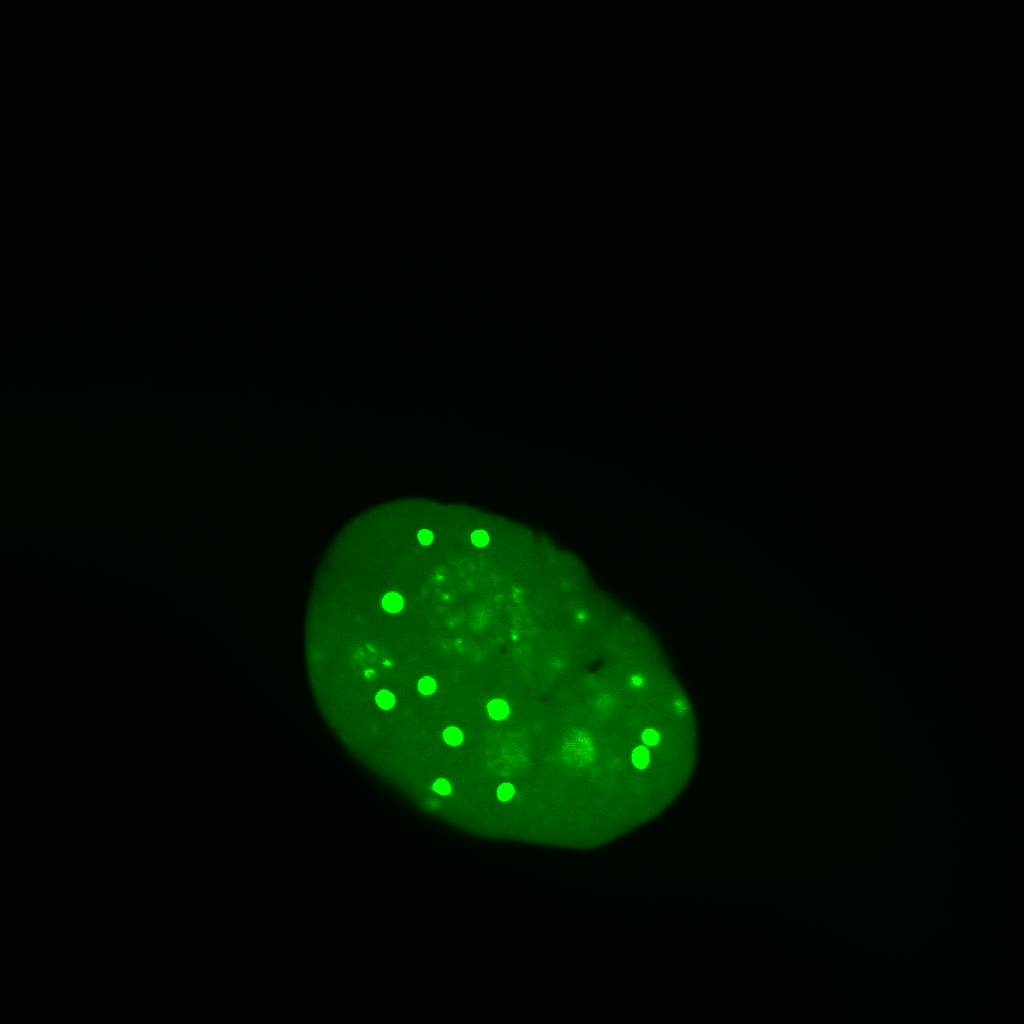

Supplement: Supplementary file 4 — Source data Fig. 2 [file 44319_2024_219_MOESM4_ESM.zip › Figure2/2B/PARP1-/PARP1-before.tif]

Figure 2C

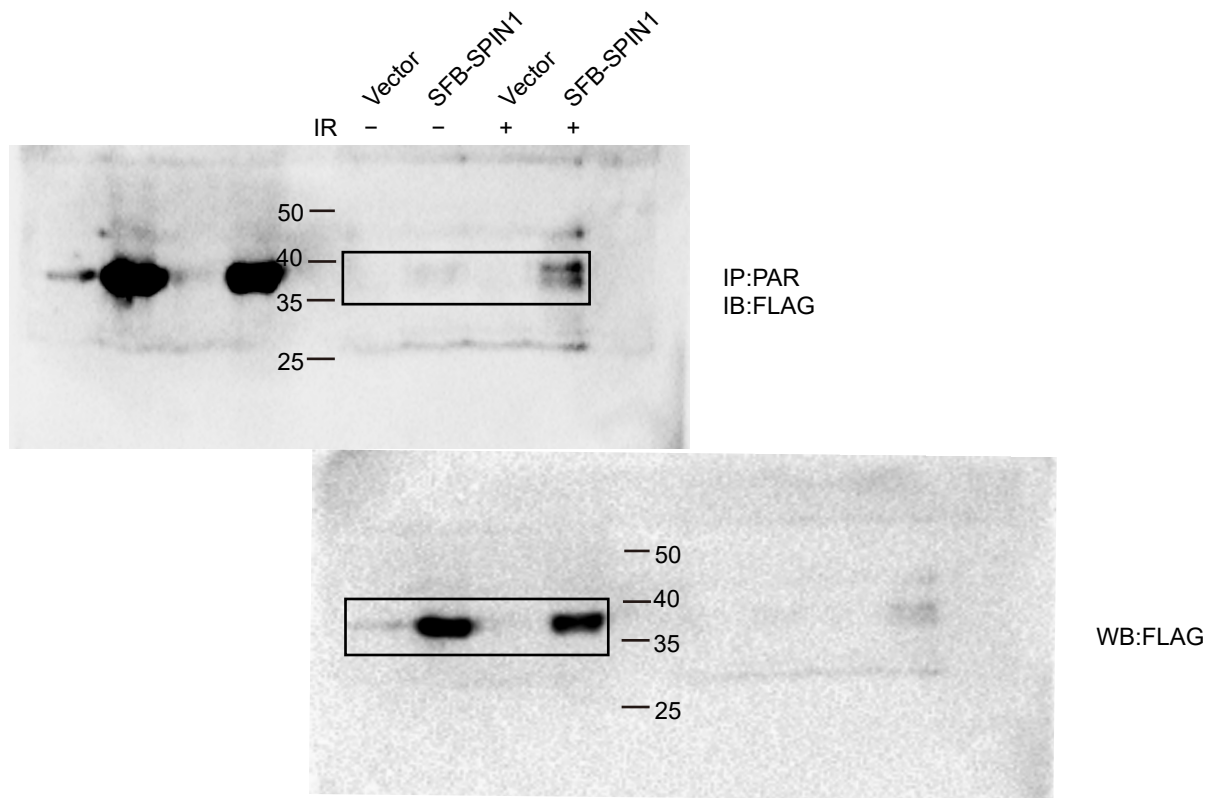

Supplement: Supplementary file 4 — Source data Fig. 2 [file 44319_2024_219_MOESM4_ESM.zip › Figure2/2C/2C.pdf]

Figure 2D

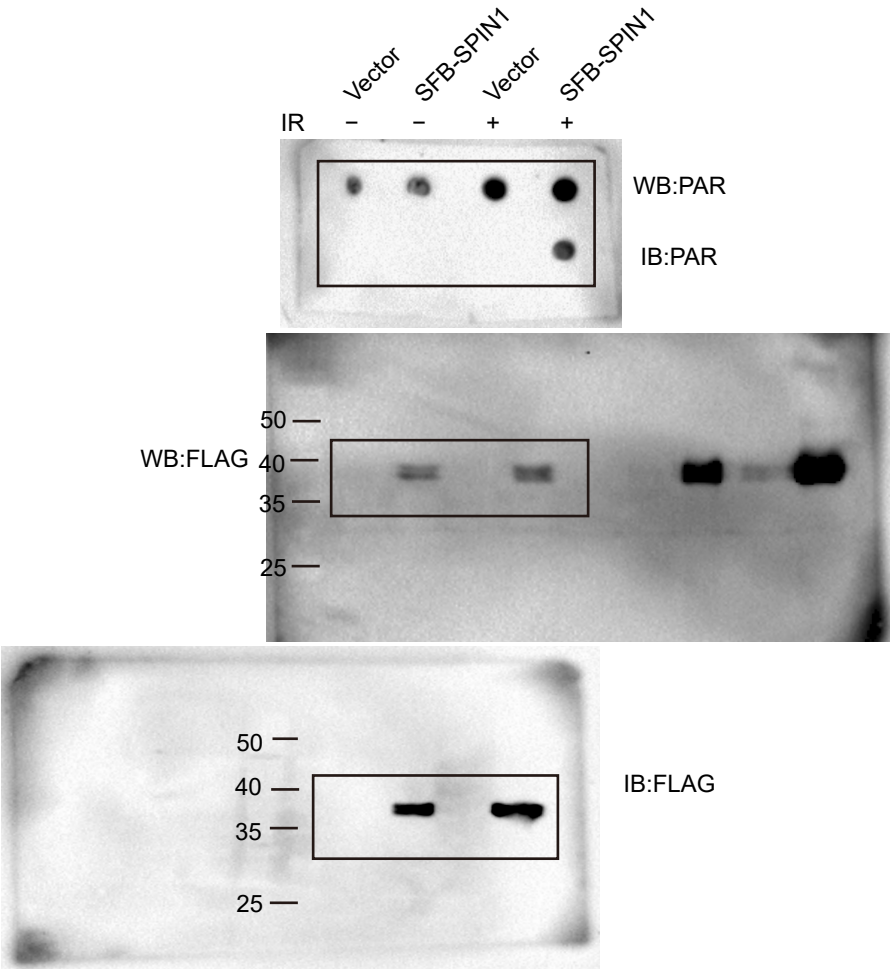

Supplement: Supplementary file 4 — Source data Fig. 2 [file 44319_2024_219_MOESM4_ESM.zip › Figure2/2D/2D.pdf]

Figure 2E

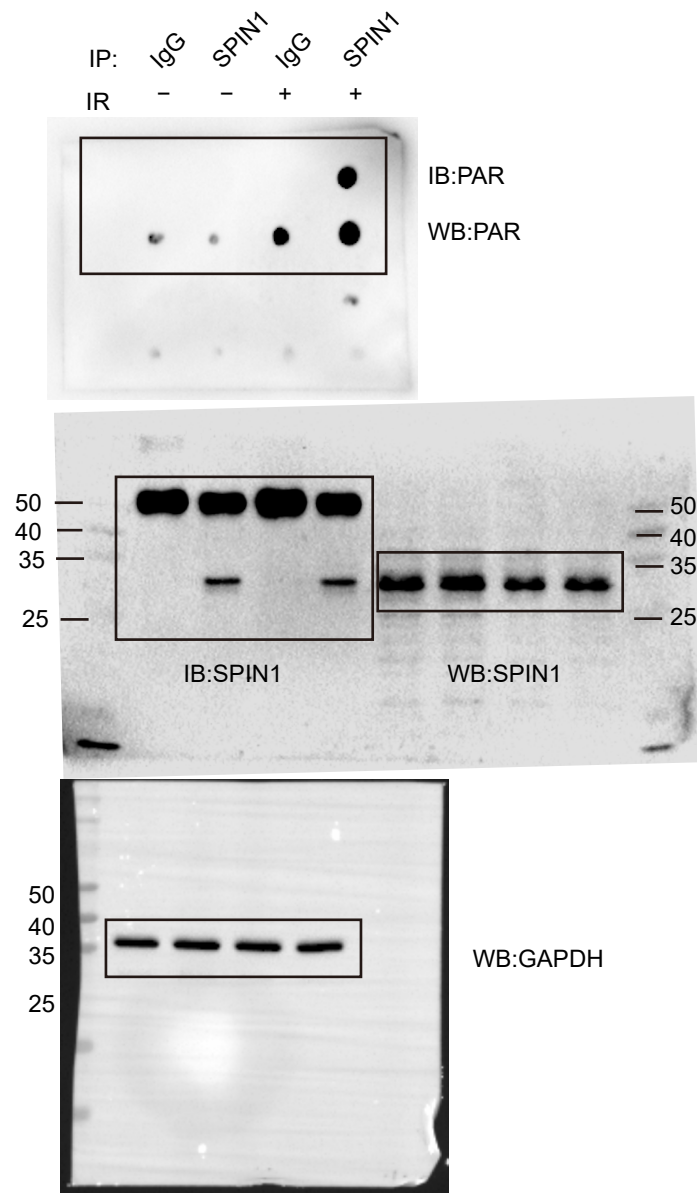

Supplement: Supplementary file 4 — Source data Fig. 2 [file 44319_2024_219_MOESM4_ESM.zip › Figure2/2E/2E.pdf]

Figure 2F

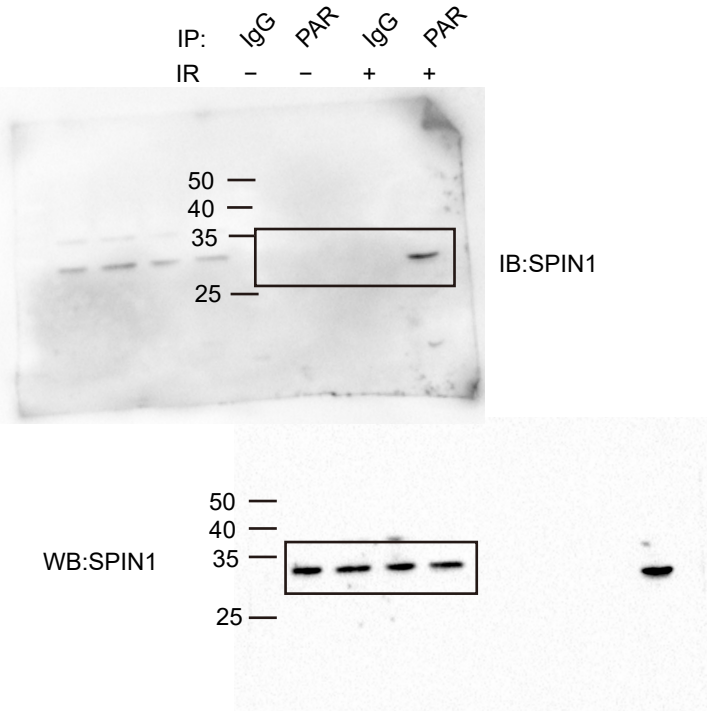

Supplement: Supplementary file 4 — Source data Fig. 2 [file 44319_2024_219_MOESM4_ESM.zip › Figure2/2F/2F.pdf]

Figure 2G

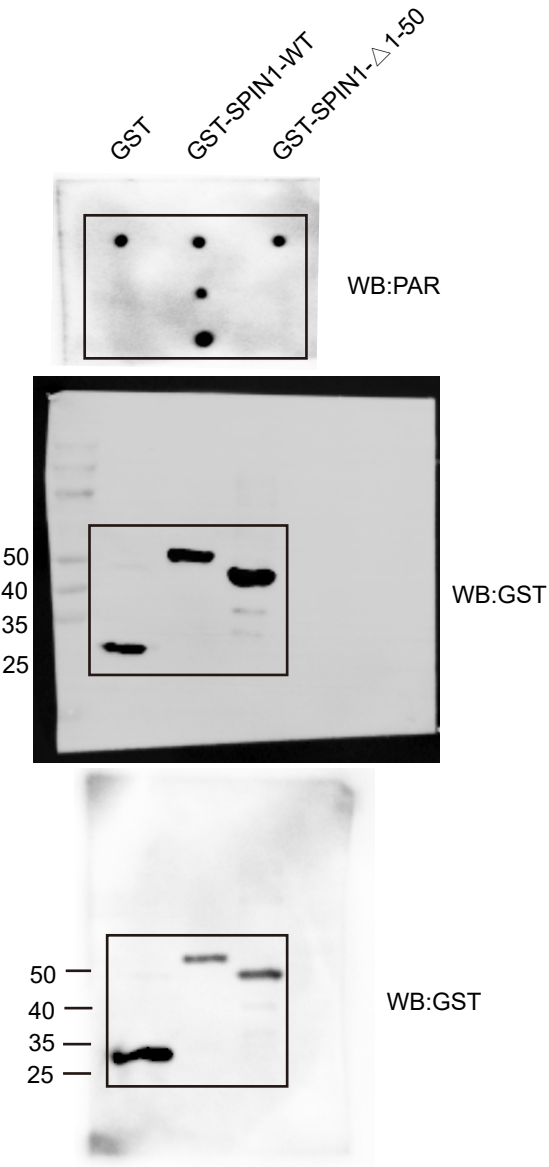

Supplement: Supplementary file 4 — Source data Fig. 2 [file 44319_2024_219_MOESM4_ESM.zip › Figure2/2G/2G.pdf]

Figure 2H

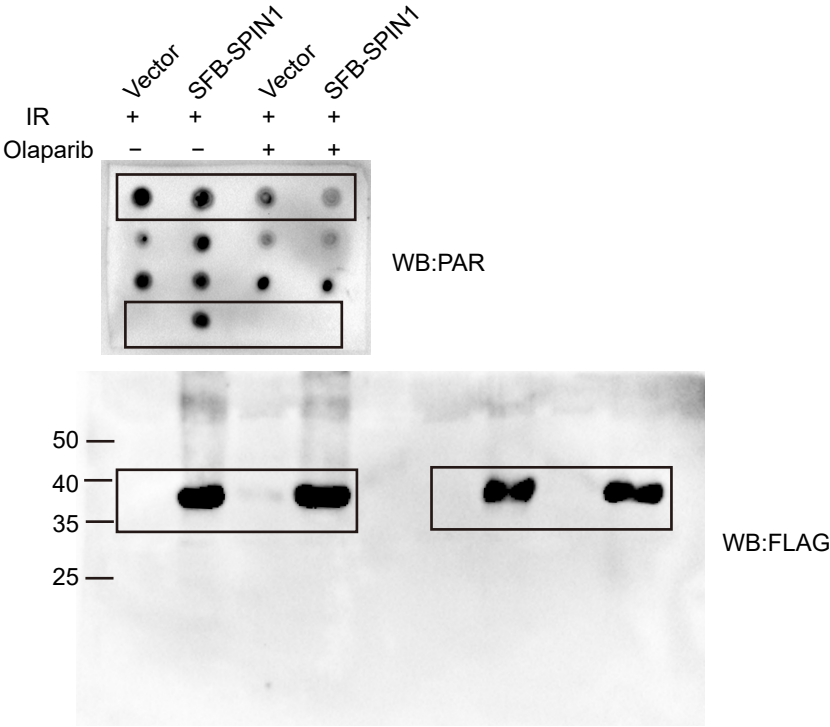

Supplement: Supplementary file 4 — Source data Fig. 2 [file 44319_2024_219_MOESM4_ESM.zip › Figure2/2H/2H.pdf]

Figure 3A

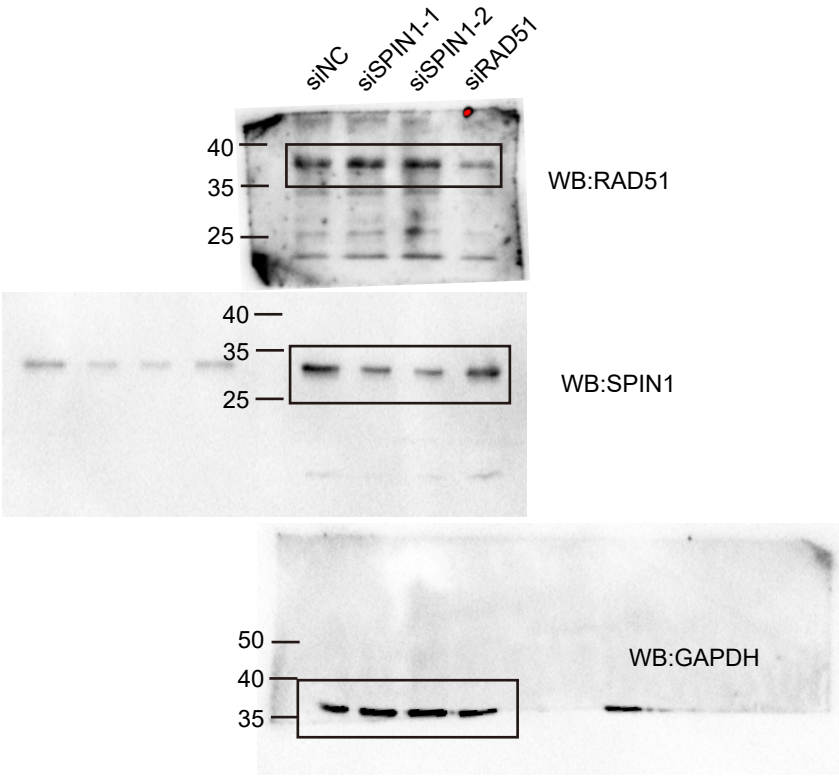

Supplement: Supplementary file 5 — Source data Fig. 3 [file 44319_2024_219_MOESM5_ESM.zip › Figure3/3A/3A.pdf]

Figure 3B

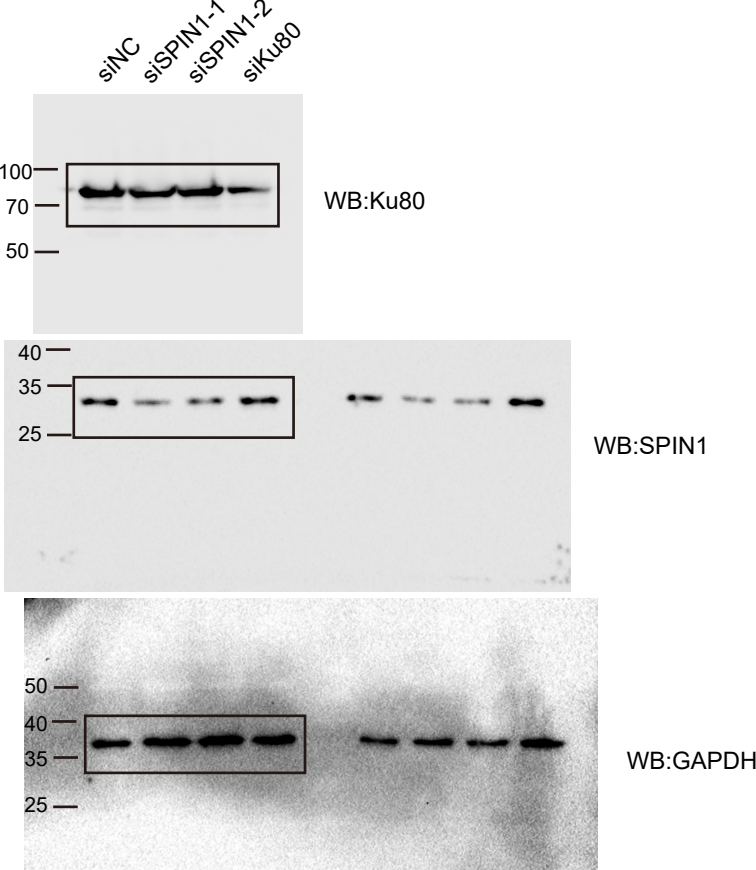

Supplement: Supplementary file 5 — Source data Fig. 3 [file 44319_2024_219_MOESM5_ESM.zip › Figure3/3B/3B.pdf]

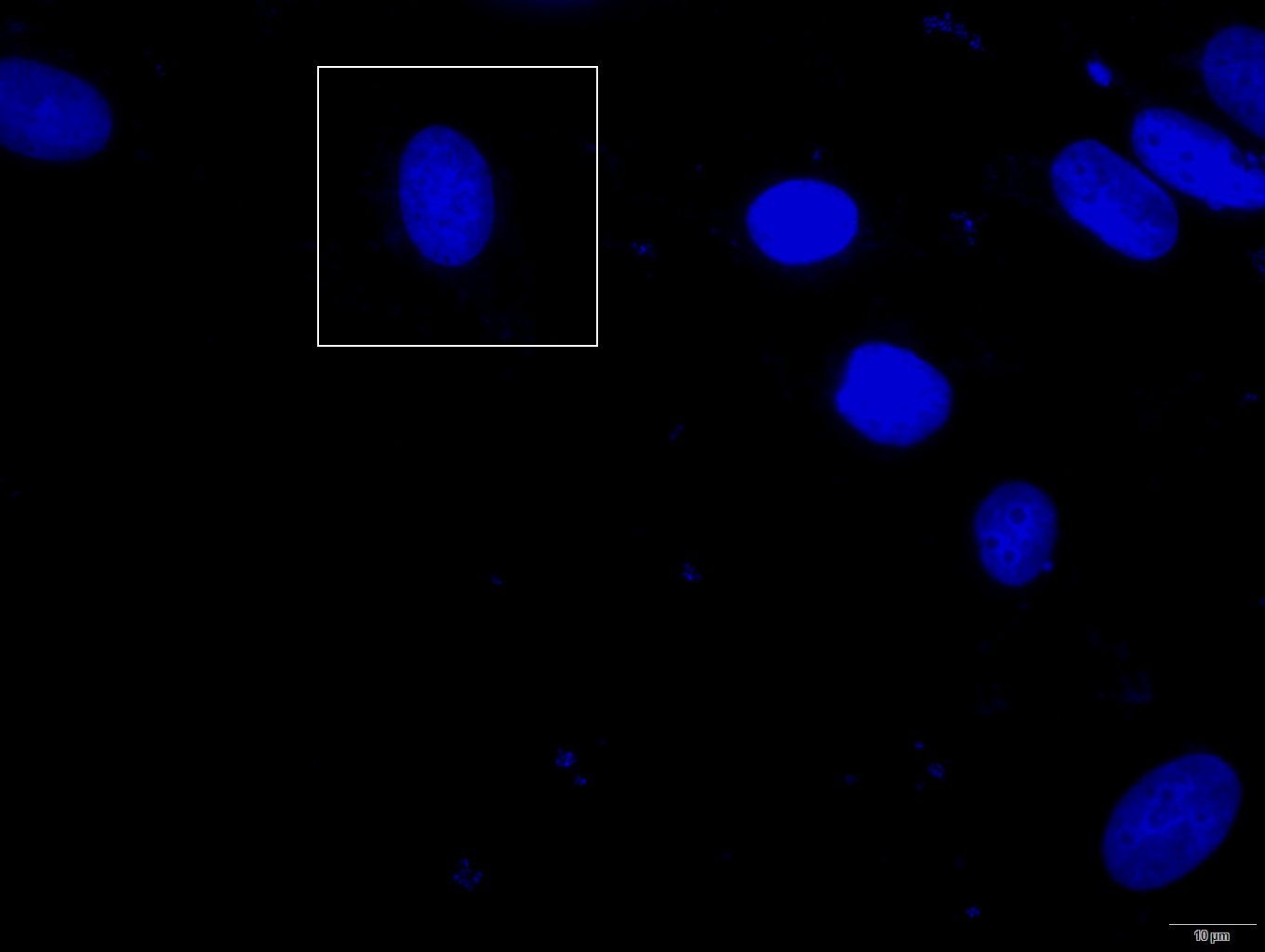

Supplement: Supplementary file 5 — Source data Fig. 3 [file 44319_2024_219_MOESM5_ESM.zip › Figure3/3C/siNC-DAPI.tif]

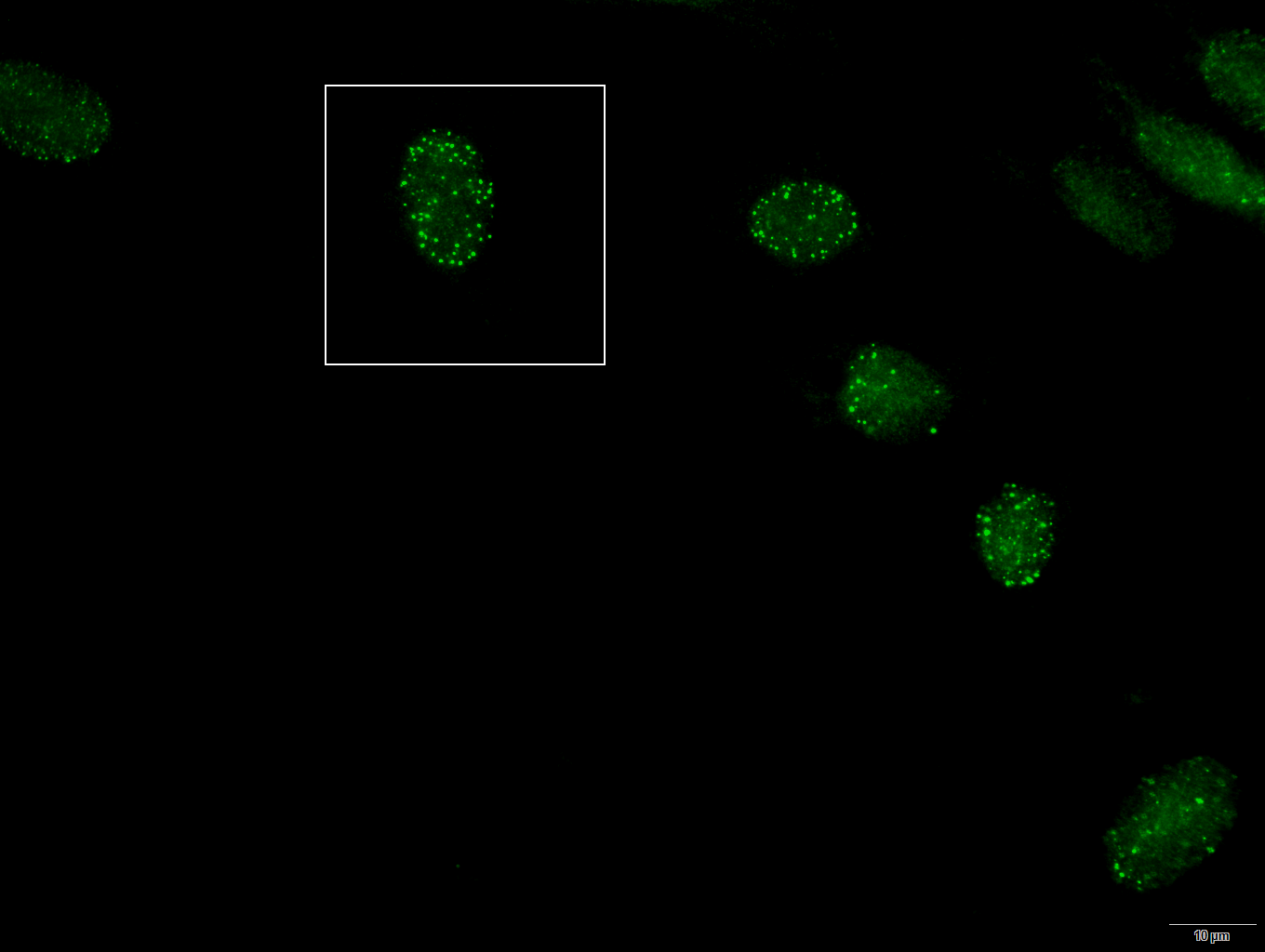

Supplement: Supplementary file 5 — Source data Fig. 3 [file 44319_2024_219_MOESM5_ESM.zip › Figure3/3C/siNC-RAD51.tif]

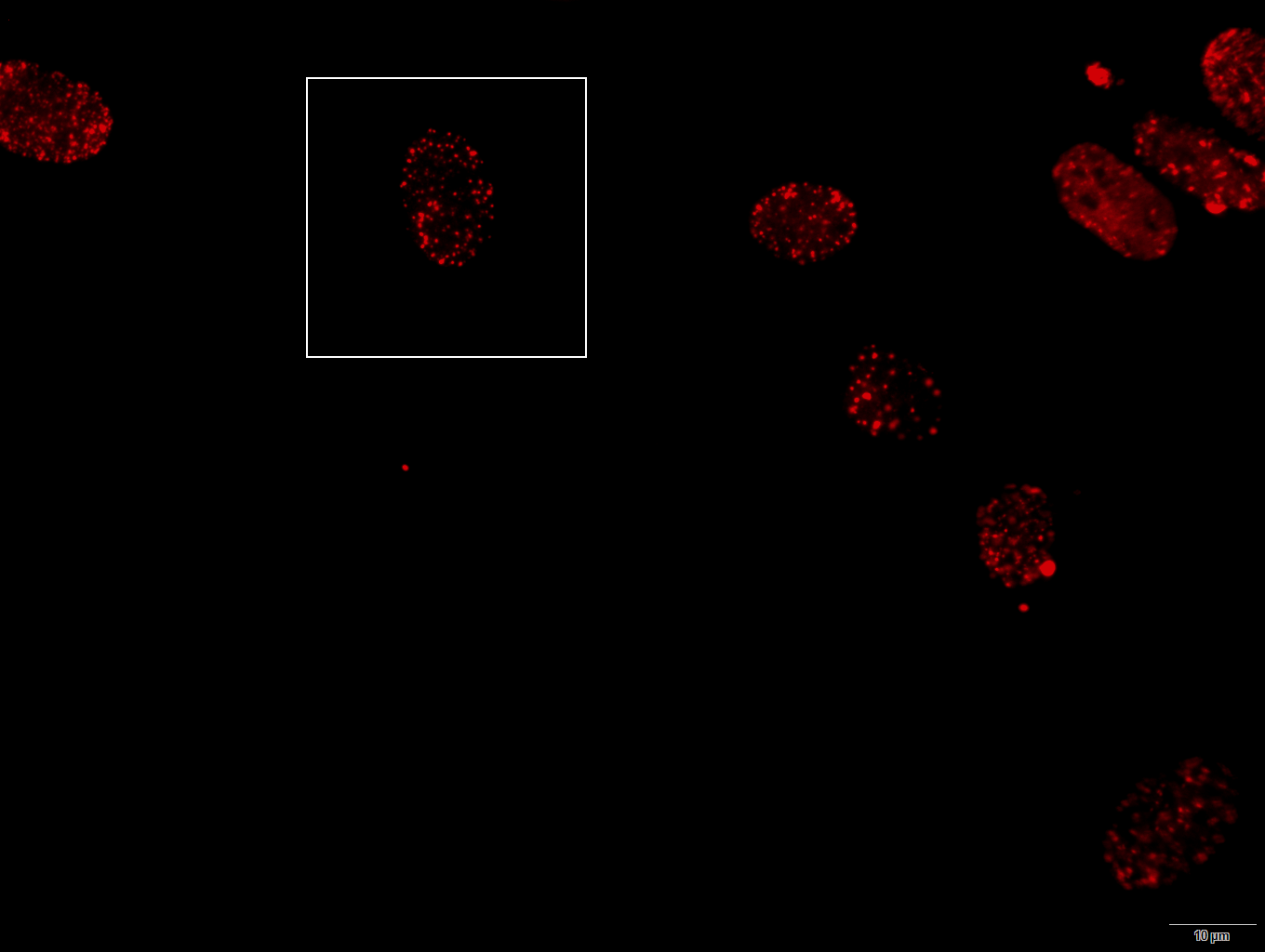

Supplement: Supplementary file 5 — Source data Fig. 3 [file 44319_2024_219_MOESM5_ESM.zip › Figure3/3C/siNC-γH2AX.tif]

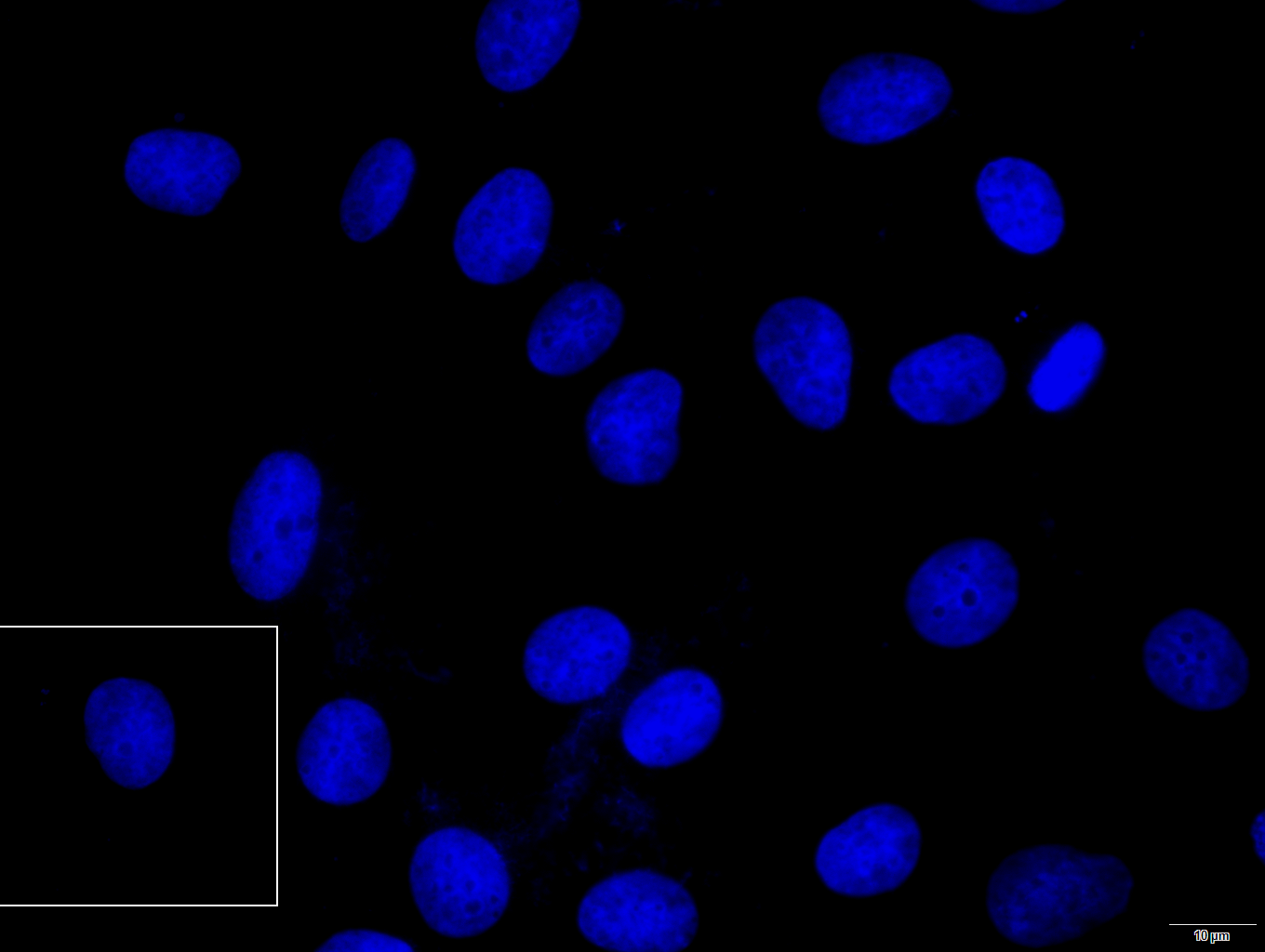

Supplement: Supplementary file 5 — Source data Fig. 3 [file 44319_2024_219_MOESM5_ESM.zip › Figure3/3C/siSPIN1-DAPI.tif]

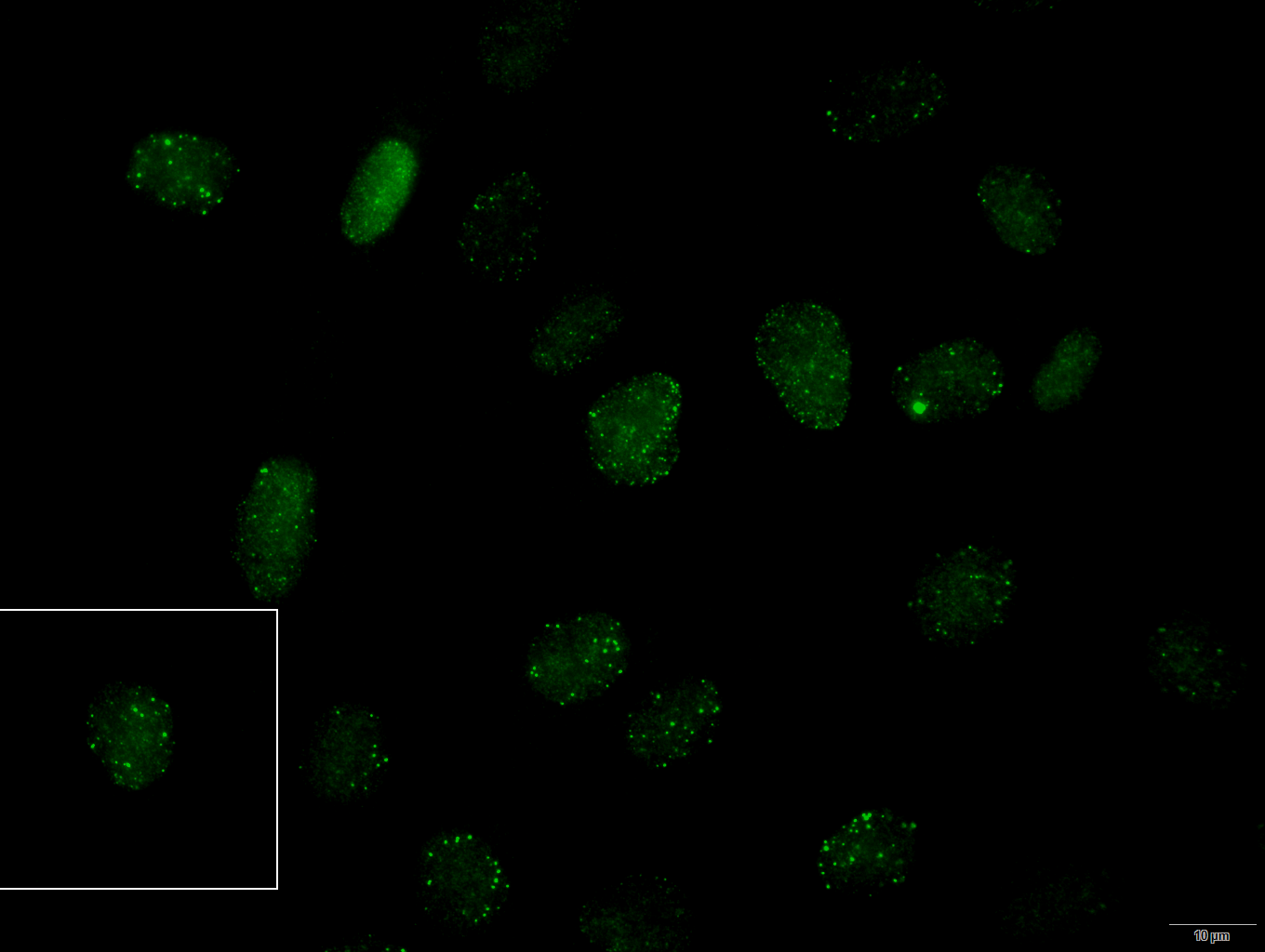

Supplement: Supplementary file 5 — Source data Fig. 3 [file 44319_2024_219_MOESM5_ESM.zip › Figure3/3C/siSPIN1-RAD51.tif]

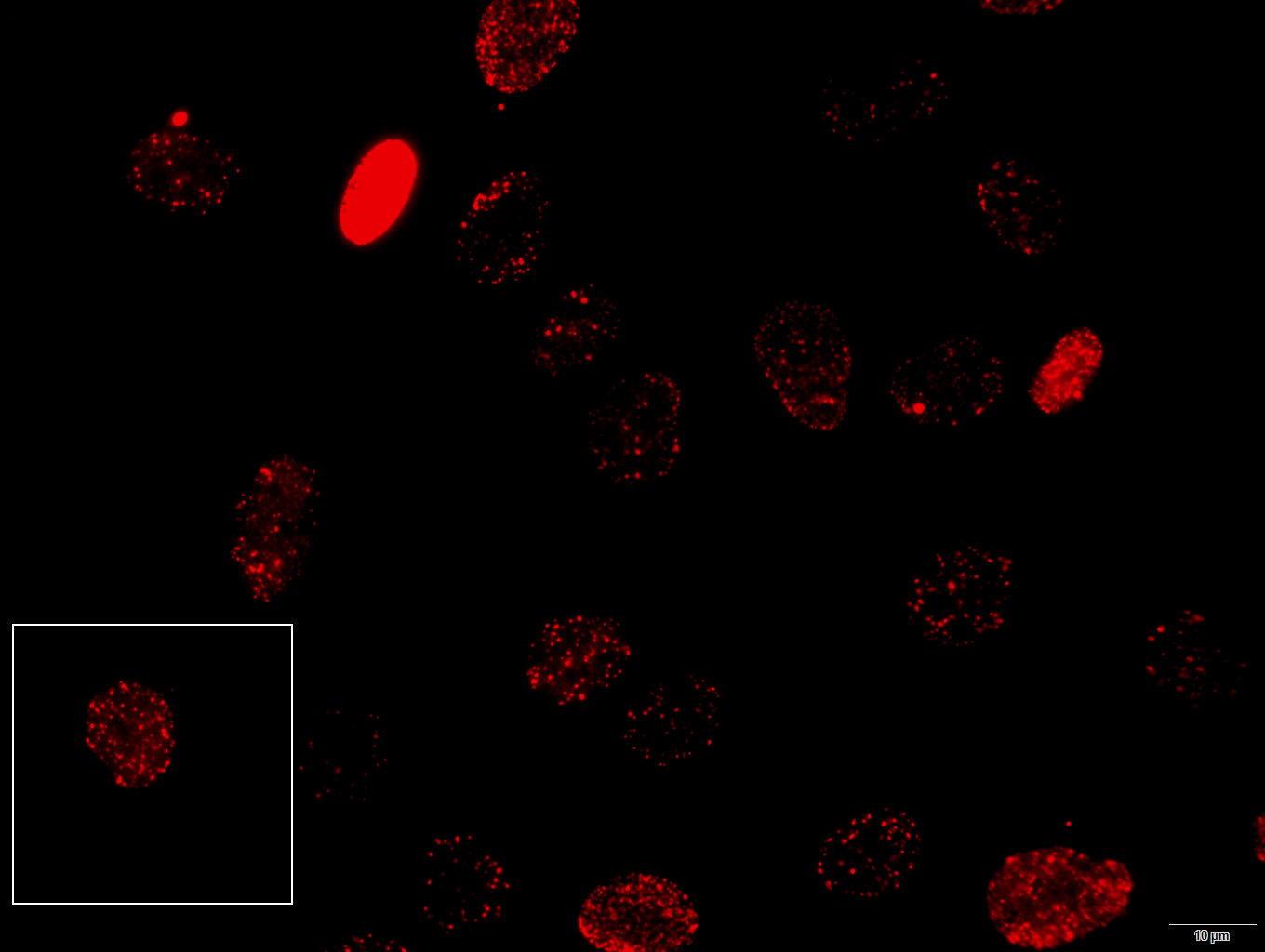

Supplement: Supplementary file 5 — Source data Fig. 3 [file 44319_2024_219_MOESM5_ESM.zip › Figure3/3C/siSPIN1-γH2AX.tif]

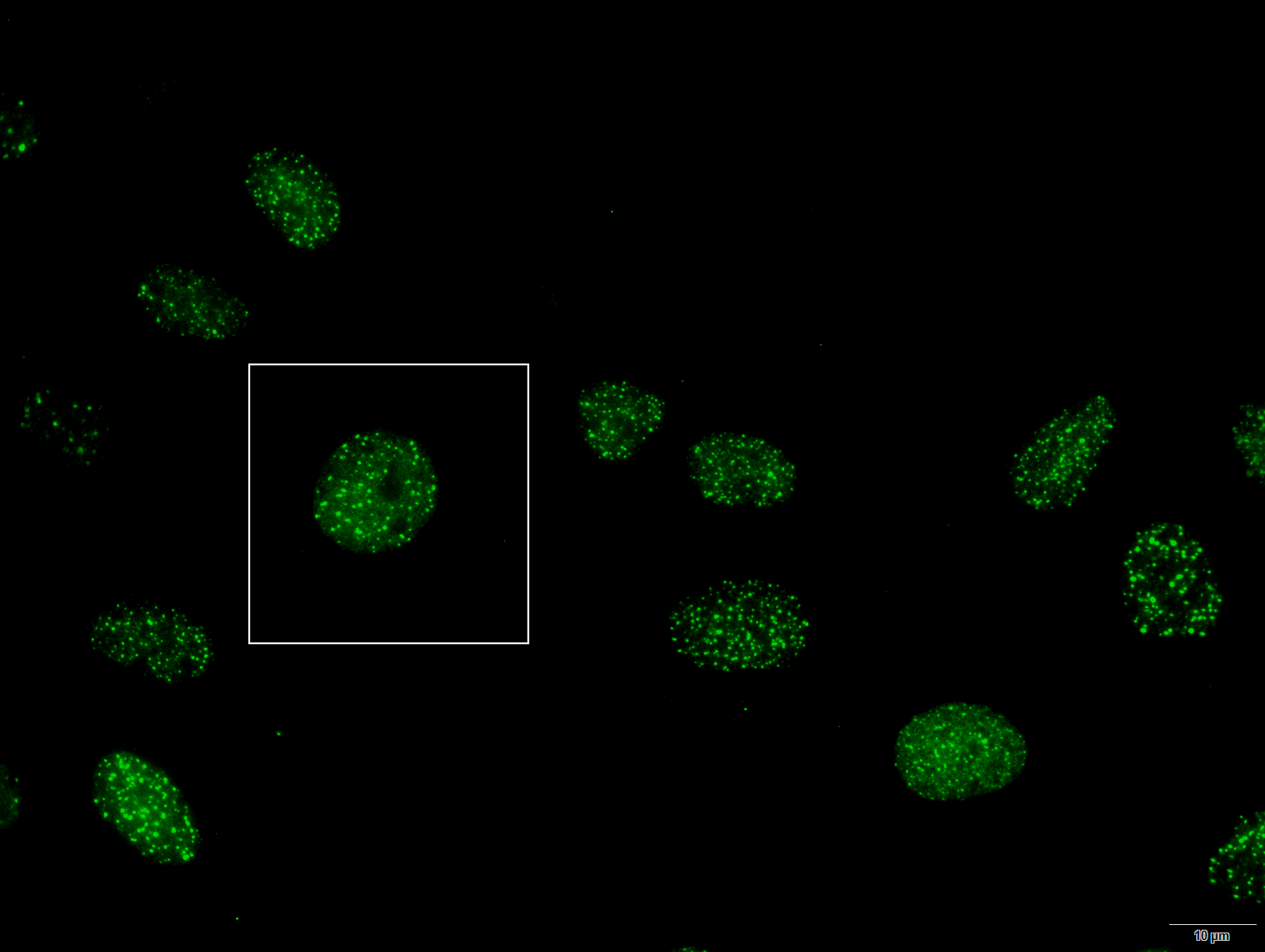

Supplement: Supplementary file 5 — Source data Fig. 3 [file 44319_2024_219_MOESM5_ESM.zip › Figure3/3D/siNC-BRCA1.tif]

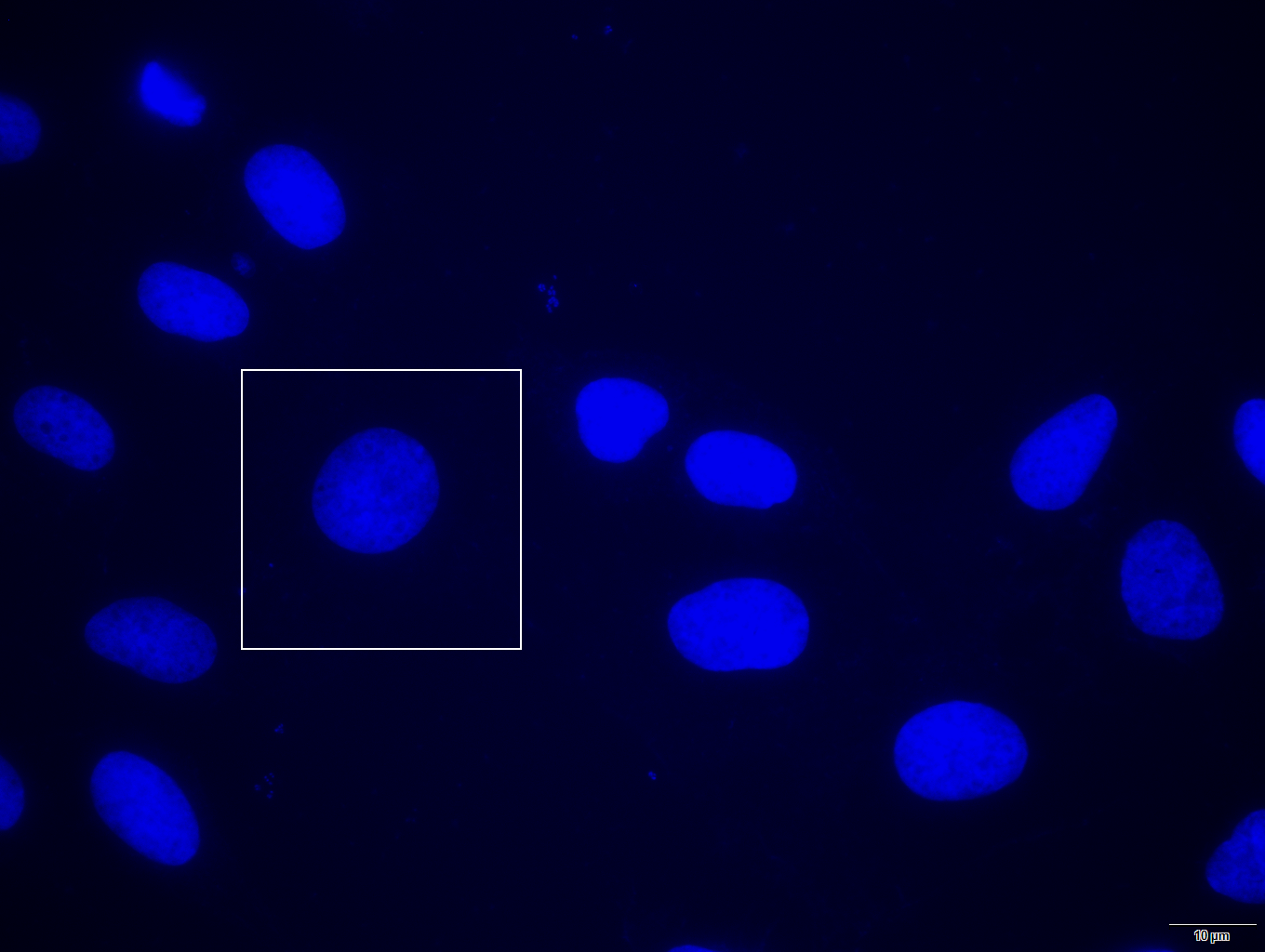

Supplement: Supplementary file 5 — Source data Fig. 3 [file 44319_2024_219_MOESM5_ESM.zip › Figure3/3D/siNC-DAPI.tif]

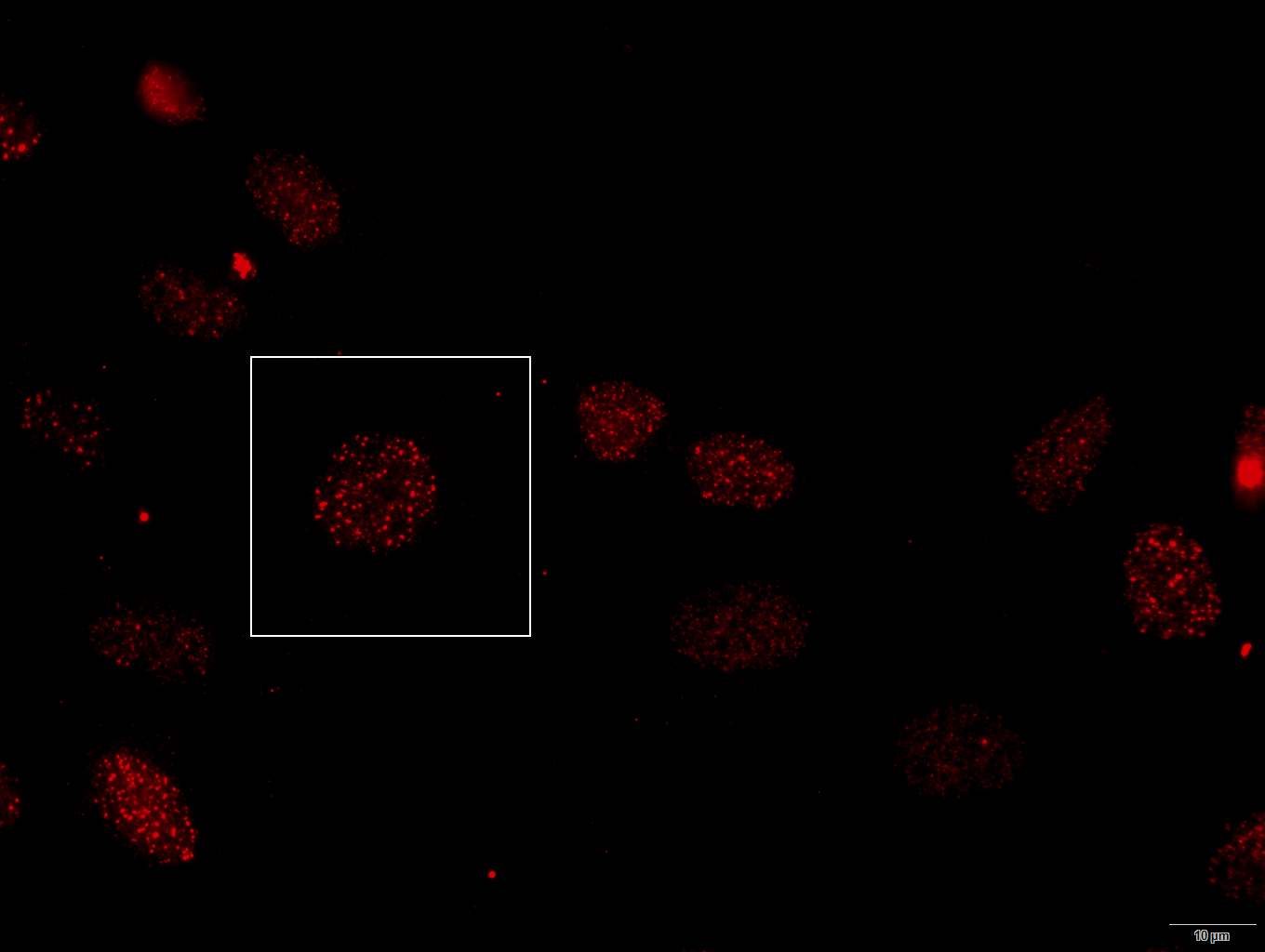

Supplement: Supplementary file 5 — Source data Fig. 3 [file 44319_2024_219_MOESM5_ESM.zip › Figure3/3D/siNC-γH2AX.tif]

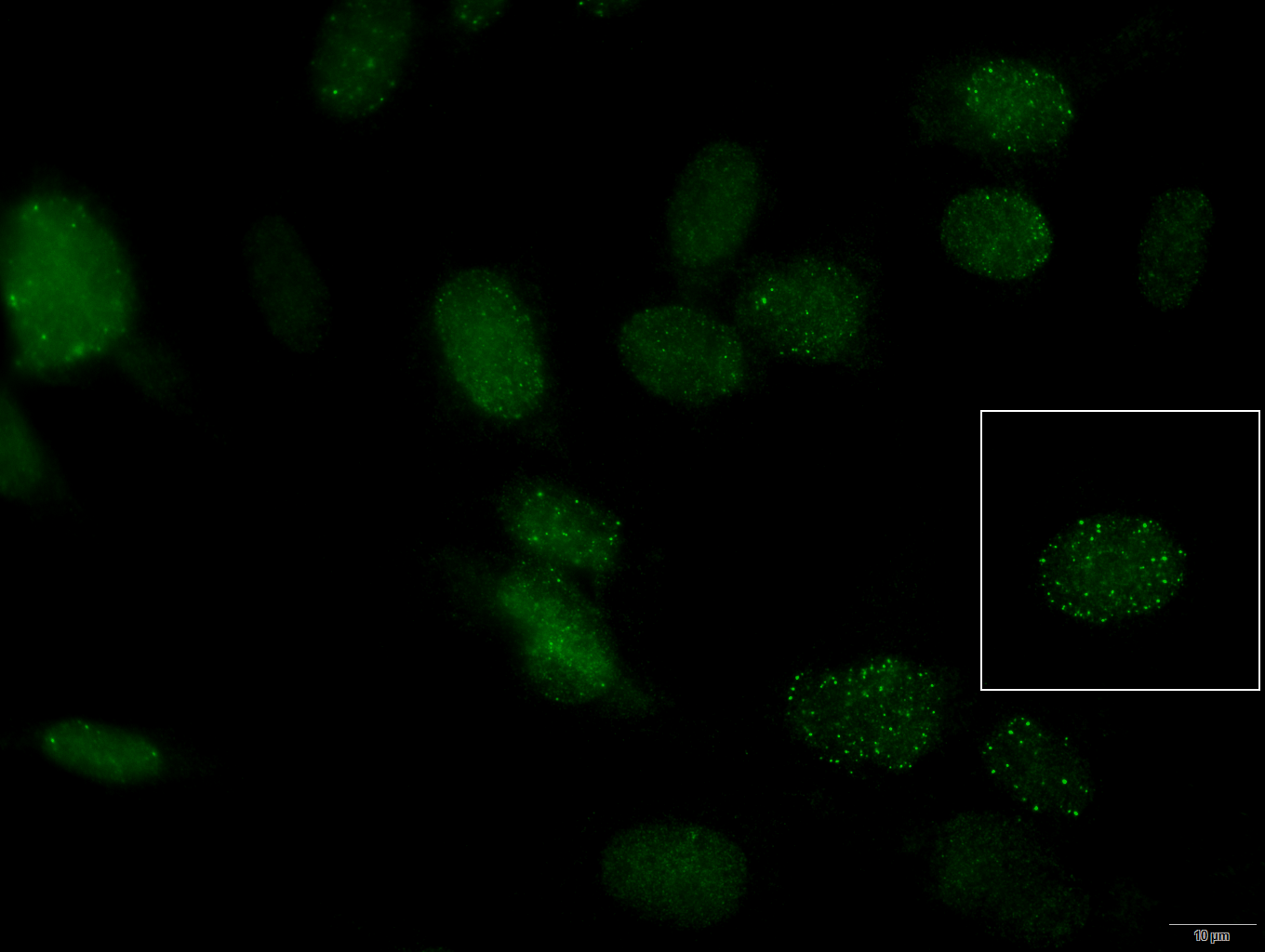

Supplement: Supplementary file 5 — Source data Fig. 3 [file 44319_2024_219_MOESM5_ESM.zip › Figure3/3D/siSPIN1-BRCA1.tif]

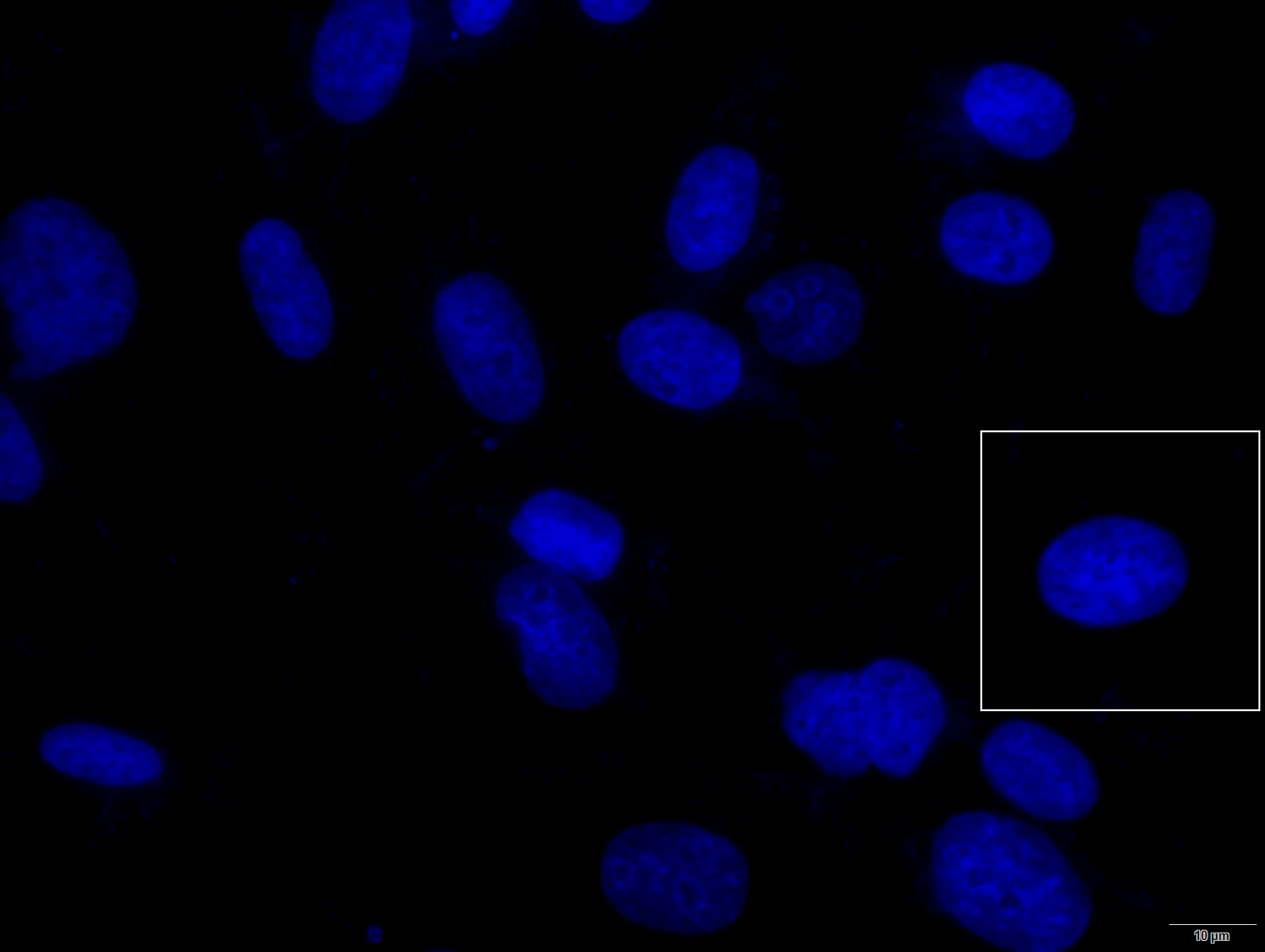

Supplement: Supplementary file 5 — Source data Fig. 3 [file 44319_2024_219_MOESM5_ESM.zip › Figure3/3D/siSPIN1-DAPI.tif]

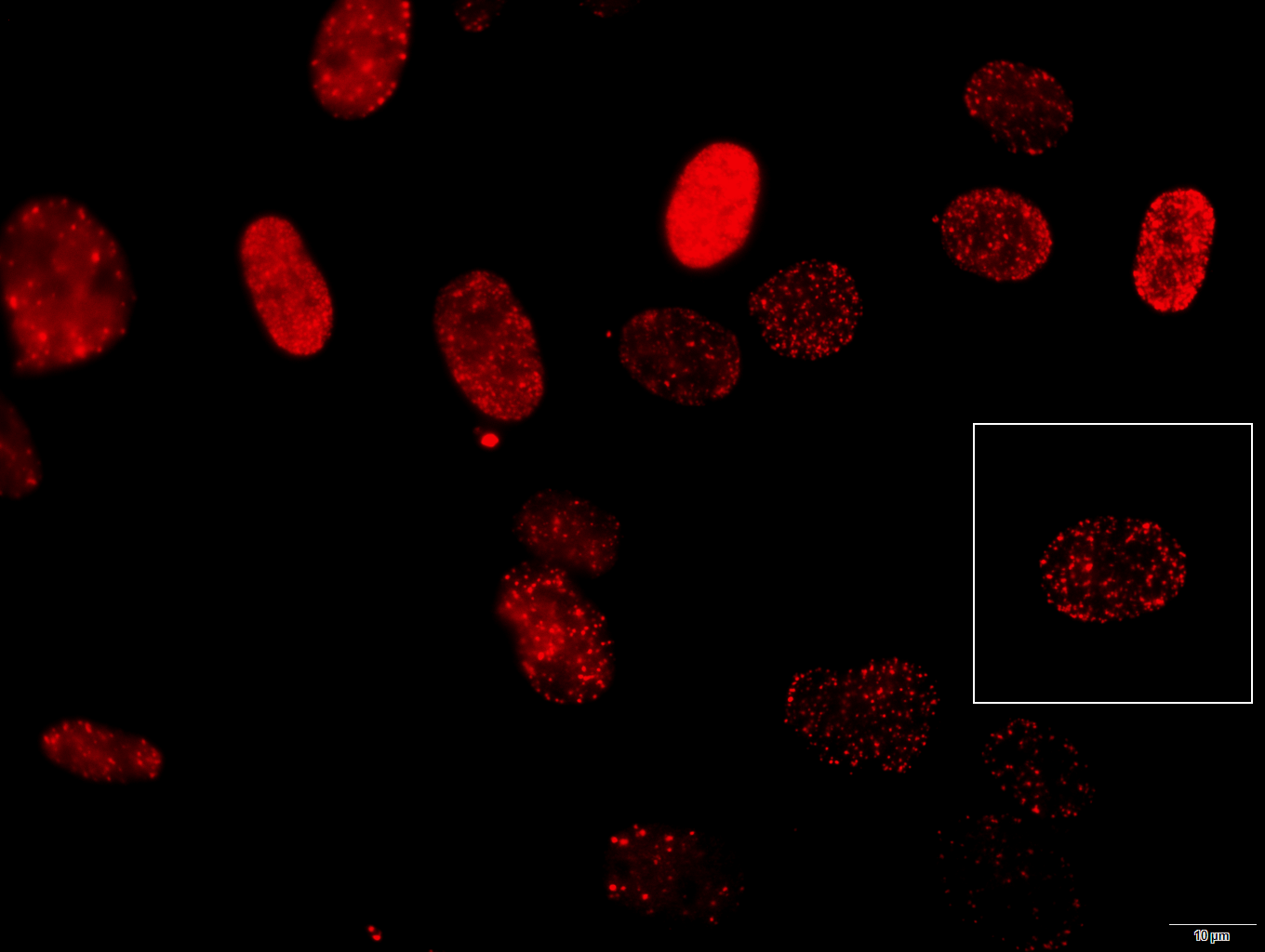

Supplement: Supplementary file 5 — Source data Fig. 3 [file 44319_2024_219_MOESM5_ESM.zip › Figure3/3D/siSPIN1-γH2AX.tif]

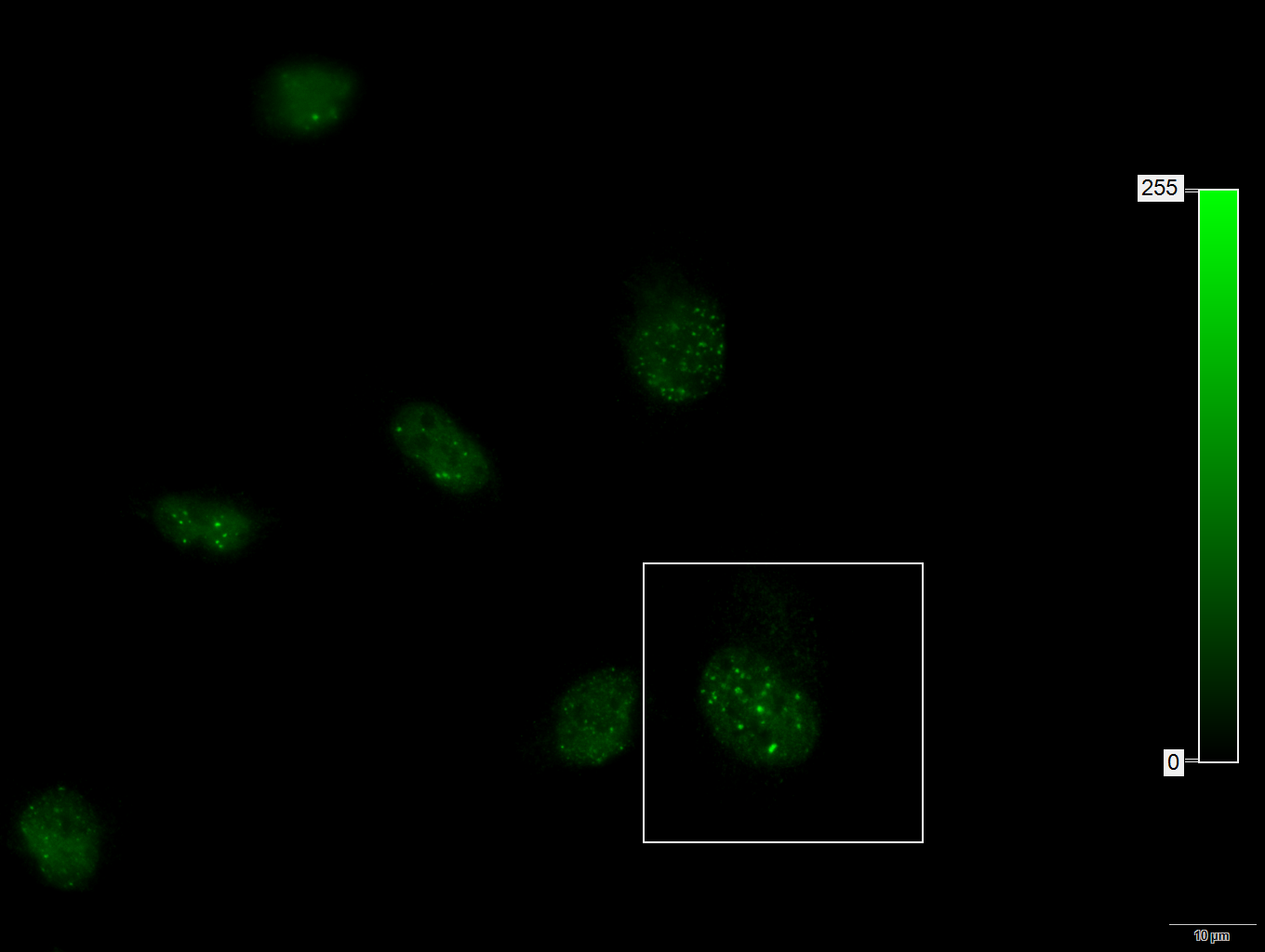

Supplement: Supplementary file 5 — Source data Fig. 3 [file 44319_2024_219_MOESM5_ESM.zip › Figure3/3E/siNC-53BP1.tif]

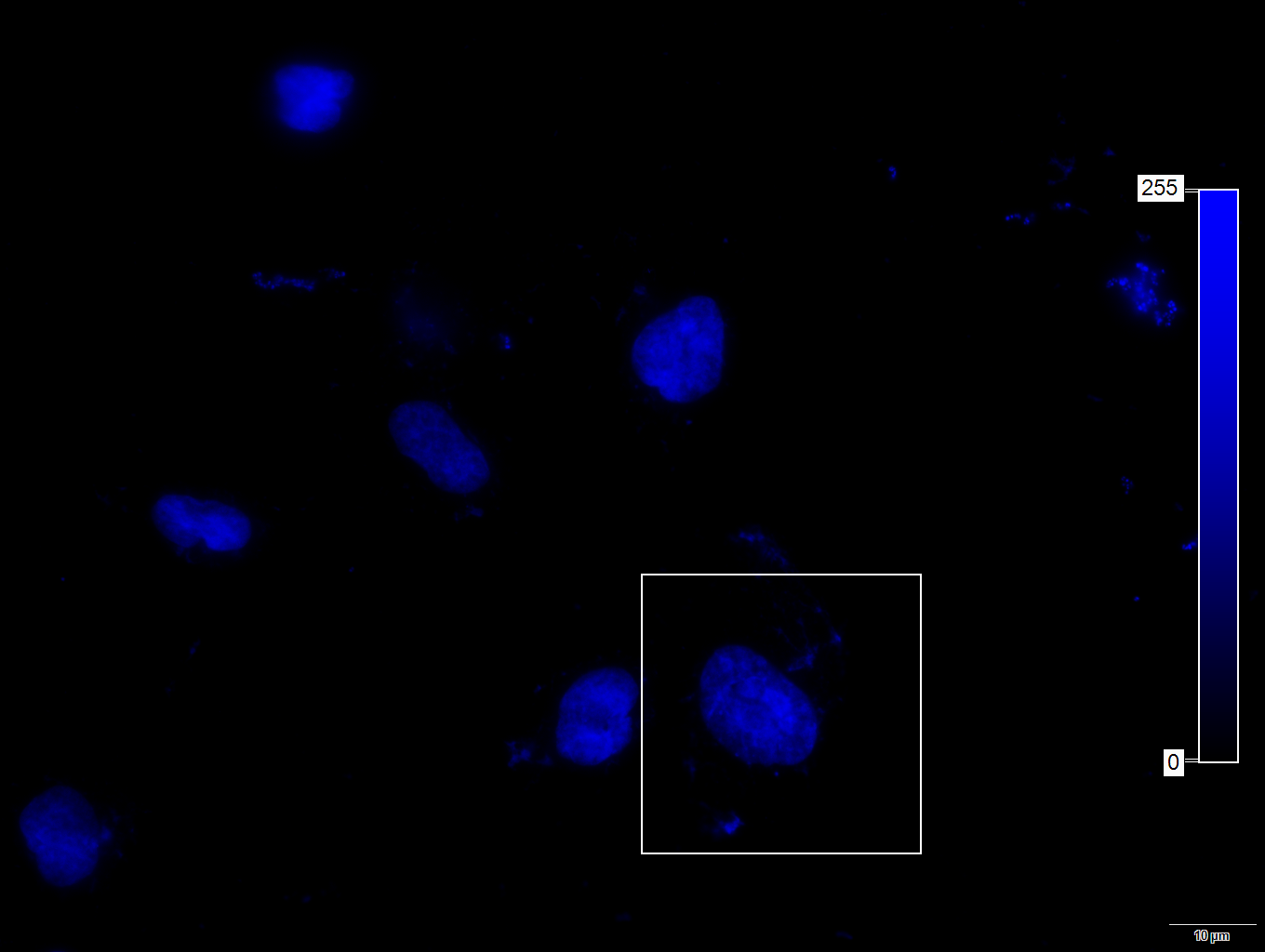

Supplement: Supplementary file 5 — Source data Fig. 3 [file 44319_2024_219_MOESM5_ESM.zip › Figure3/3E/siNC-DAPI.tif]

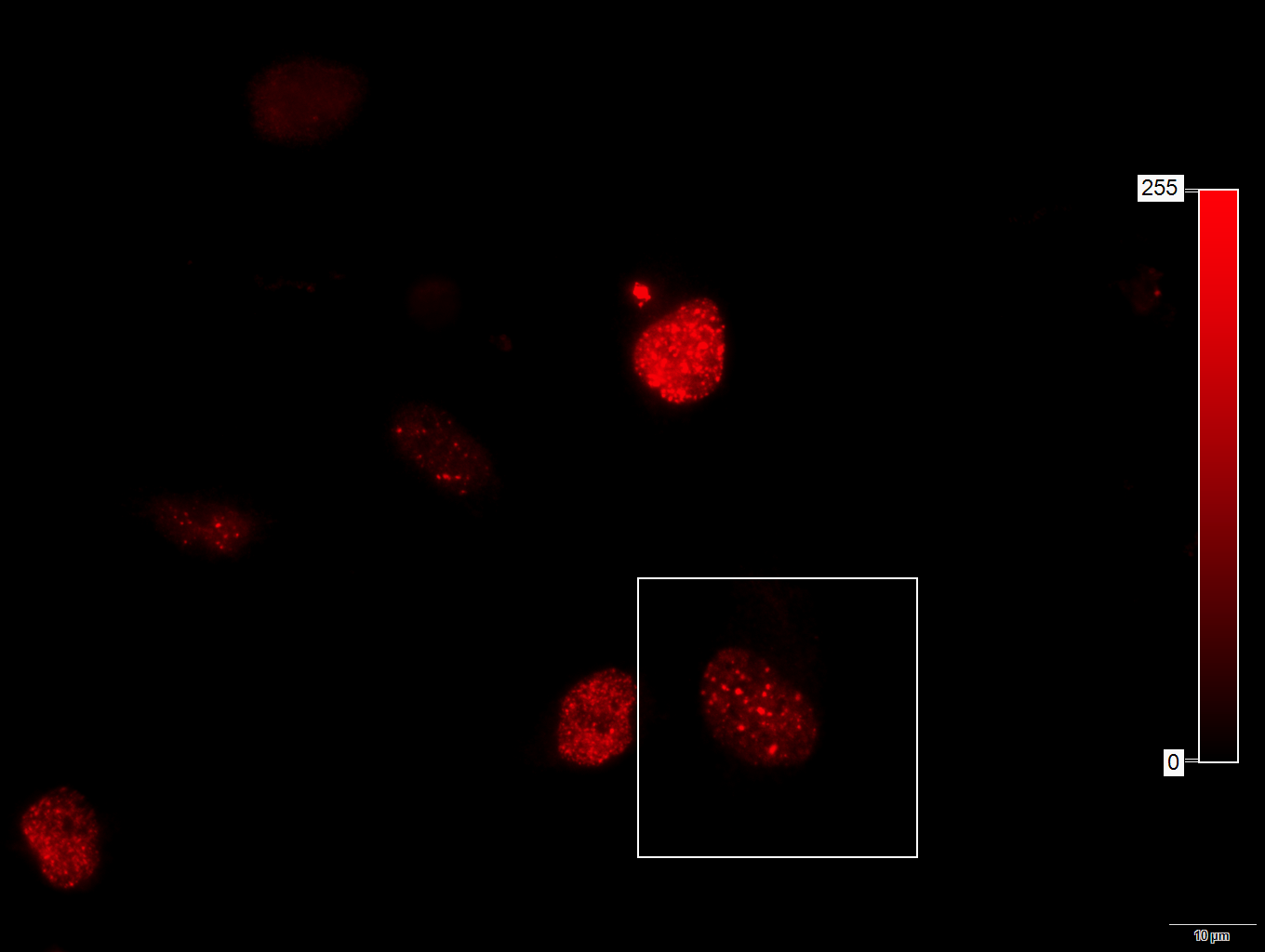

Supplement: Supplementary file 5 — Source data Fig. 3 [file 44319_2024_219_MOESM5_ESM.zip › Figure3/3E/siNC-γH2AX.tif]

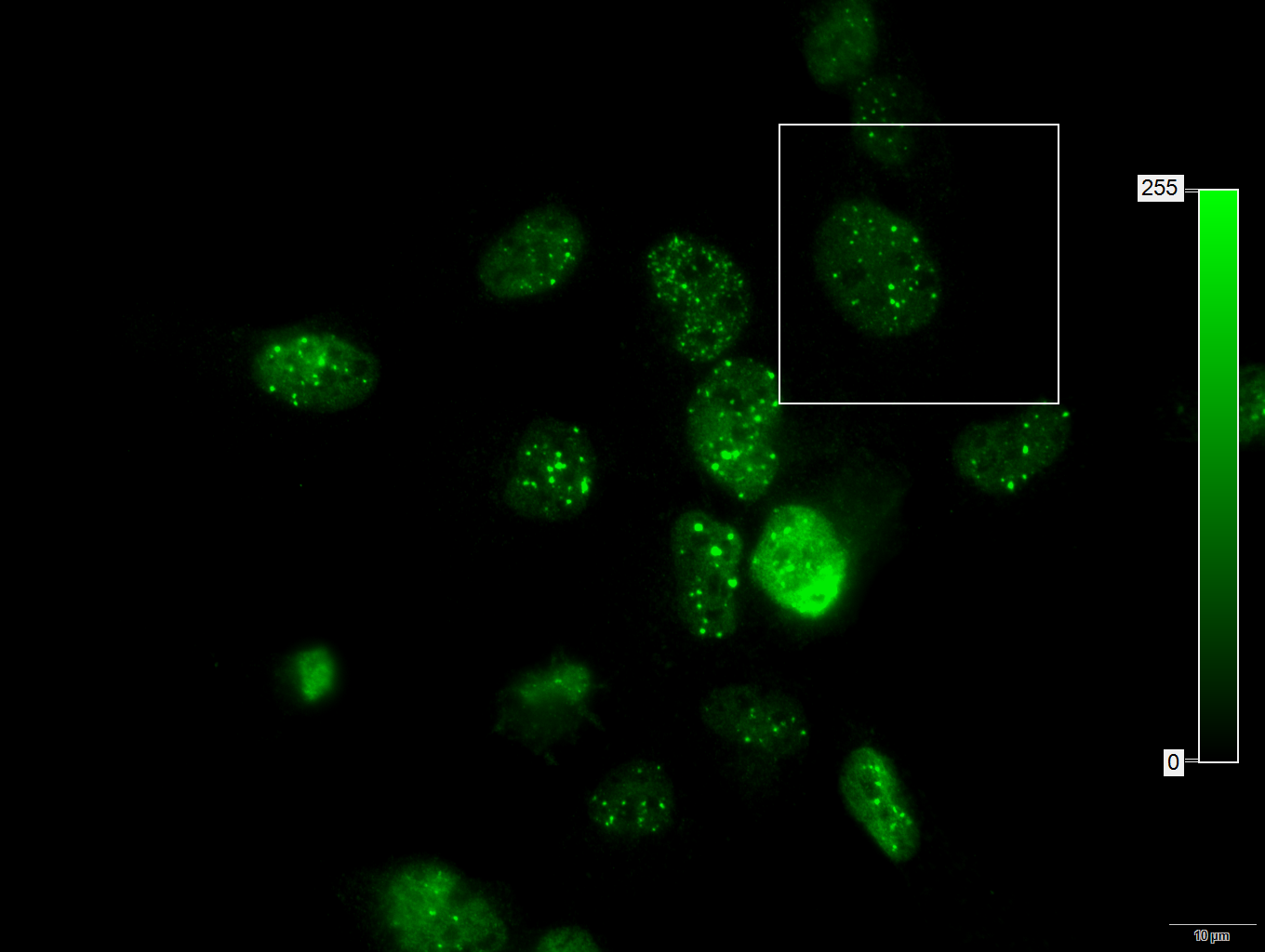

Supplement: Supplementary file 5 — Source data Fig. 3 [file 44319_2024_219_MOESM5_ESM.zip › Figure3/3E/siSPIN1-53BP1.tif]
