# Supplementary figures and images for: SPIN1 facilitates chemoresistance and HR repair by promoting Tip60 binding to H3K9me3 (part 2 of 2)
Source: EMBO Rep. 2024 Aug 1;25(9):15. doi: 10.1038/s44319-024-00219-1 (PMC11387427; doi:10.1038/s44319-024-00219-1)

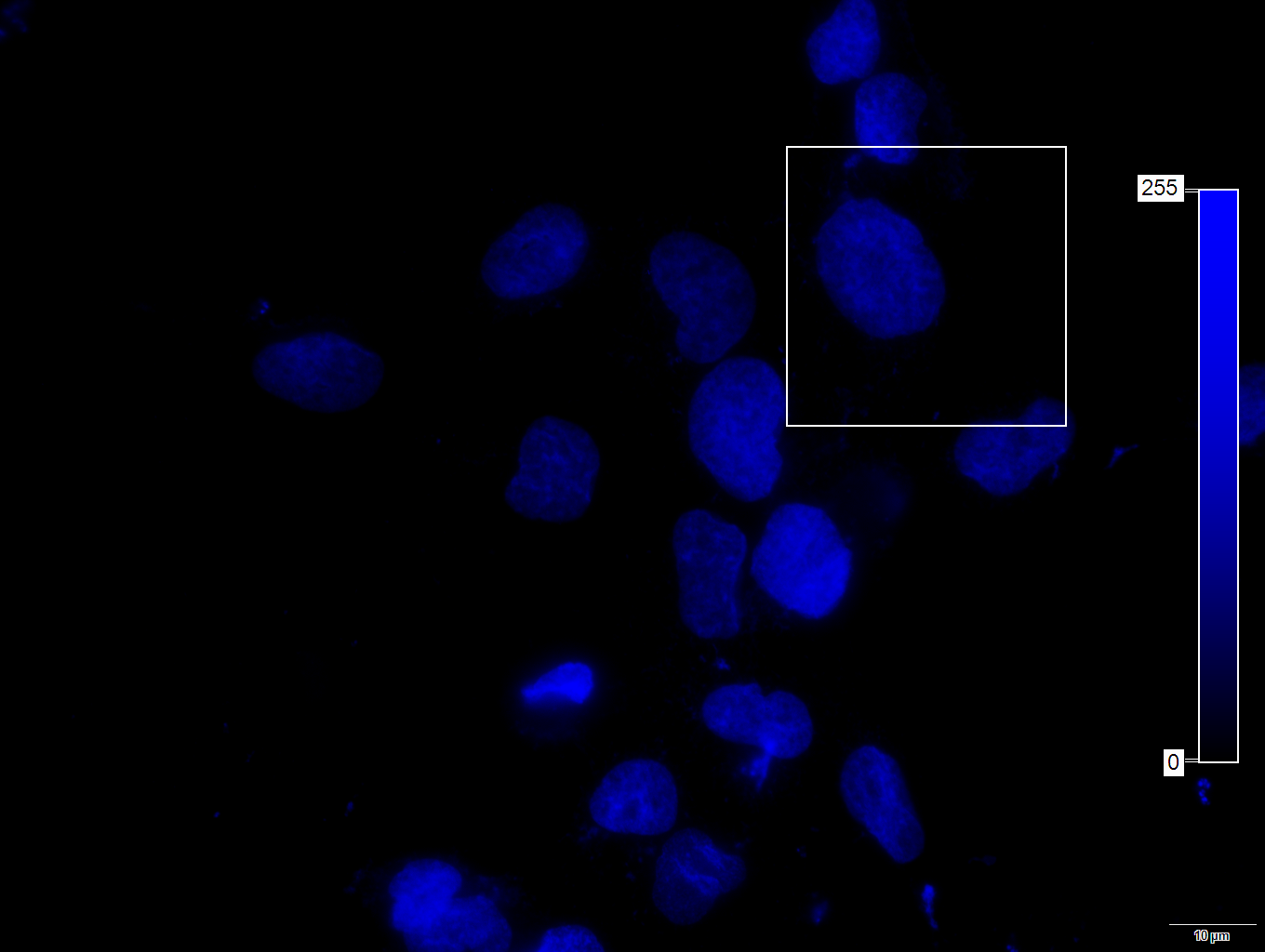

Supplement: Supplementary file 5 — Source data Fig. 3 [file 44319_2024_219_MOESM5_ESM.zip › Figure3/3E/siSPIN1-DAPI.tif]

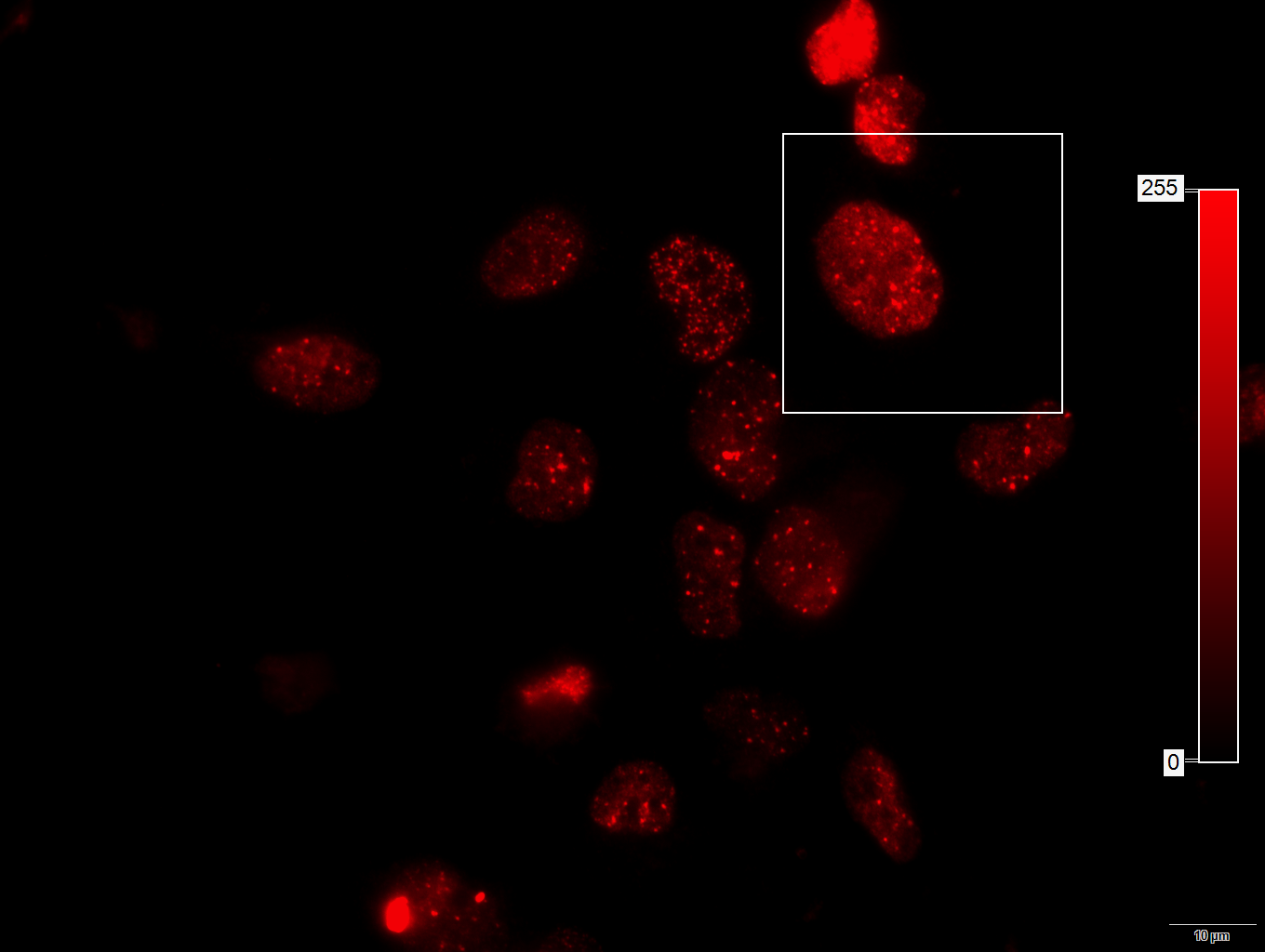

Supplement: Supplementary file 5 — Source data Fig. 3 [file 44319_2024_219_MOESM5_ESM.zip › Figure3/3E/siSPIN1-γH2AX.tif]

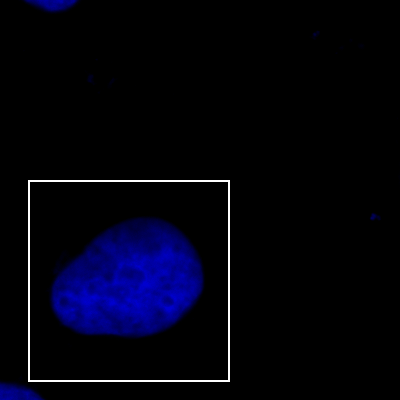

Supplement: Supplementary file 5 — Source data Fig. 3 [file 44319_2024_219_MOESM5_ESM.zip › Figure3/3I/siNC-DAPI.tif]

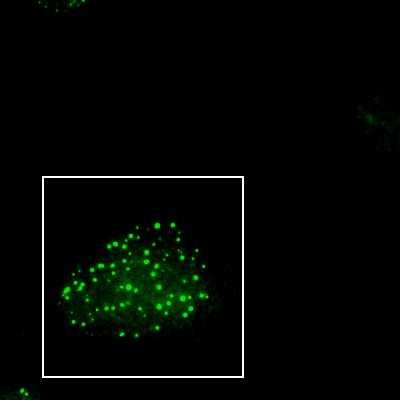

Supplement: Supplementary file 5 — Source data Fig. 3 [file 44319_2024_219_MOESM5_ESM.zip › Figure3/3I/siNC-P-ATM.tif]

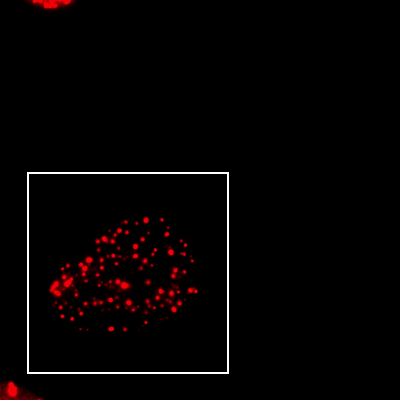

Supplement: Supplementary file 5 — Source data Fig. 3 [file 44319_2024_219_MOESM5_ESM.zip › Figure3/3I/siNC-γH2AX.tif]

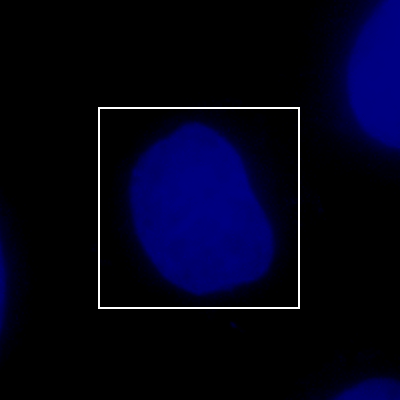

Supplement: Supplementary file 5 — Source data Fig. 3 [file 44319_2024_219_MOESM5_ESM.zip › Figure3/3I/siSPIN1-DAPI.tif]

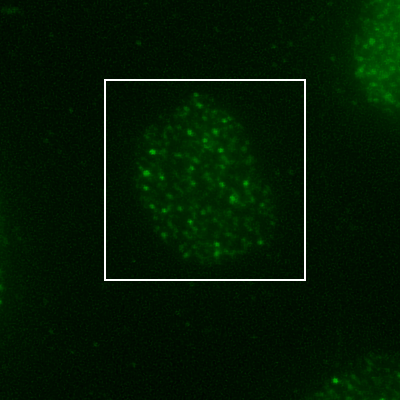

Supplement: Supplementary file 5 — Source data Fig. 3 [file 44319_2024_219_MOESM5_ESM.zip › Figure3/3I/siSPIN1-P-ATM.tif]

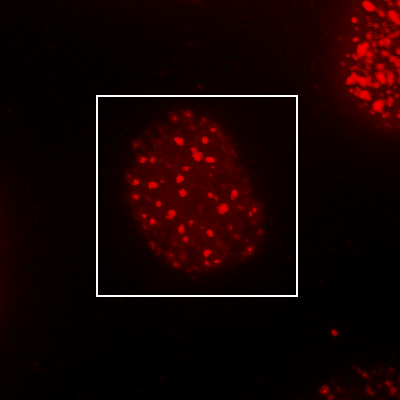

Supplement: Supplementary file 5 — Source data Fig. 3 [file 44319_2024_219_MOESM5_ESM.zip › Figure3/3I/siSPIN1-γH2AX.tif]

Figure 3M

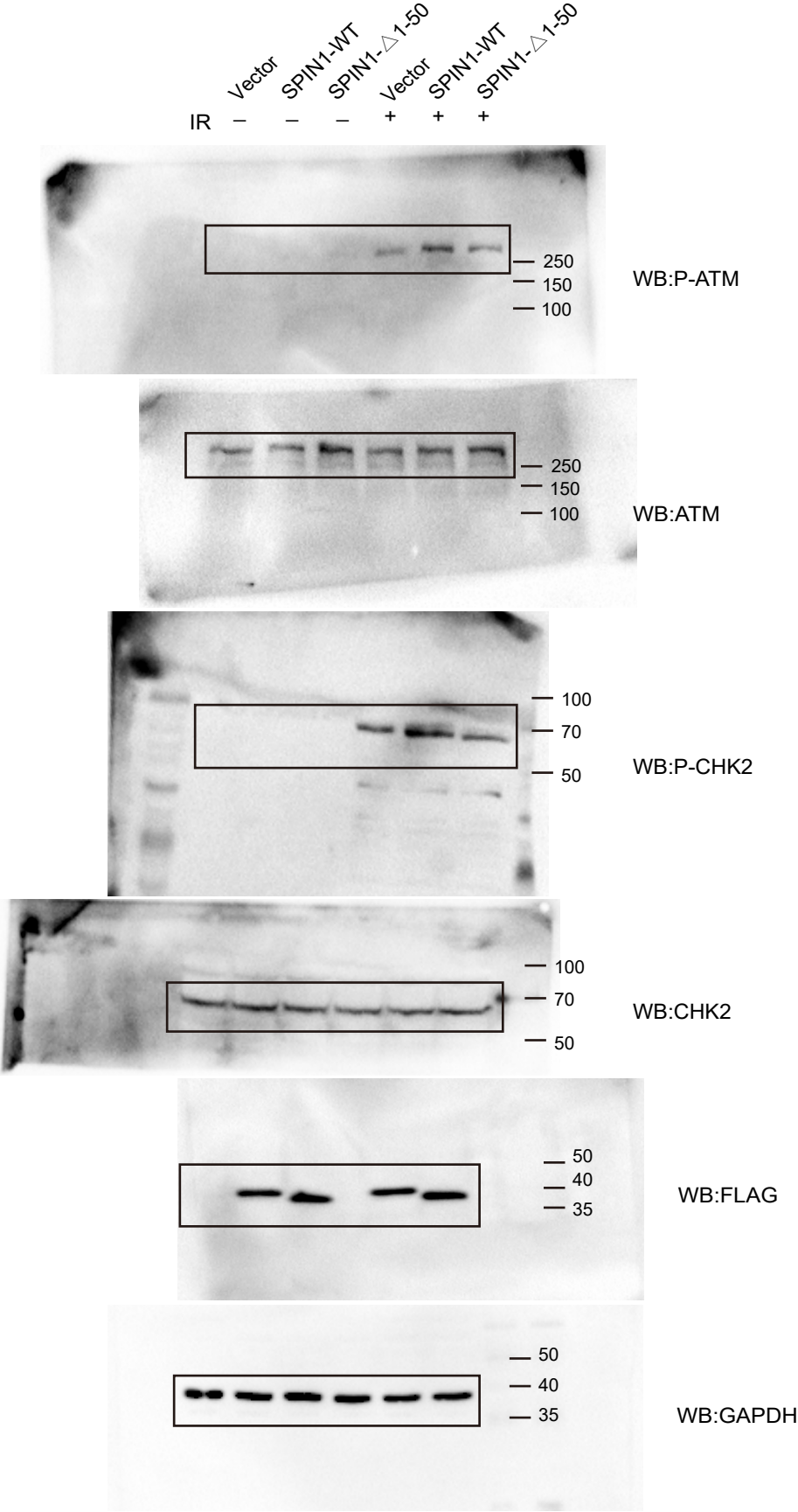

Supplement: Supplementary file 5 — Source data Fig. 3 [file 44319_2024_219_MOESM5_ESM.zip › Figure3/3M/Figure3M.pdf]

Figure 3K

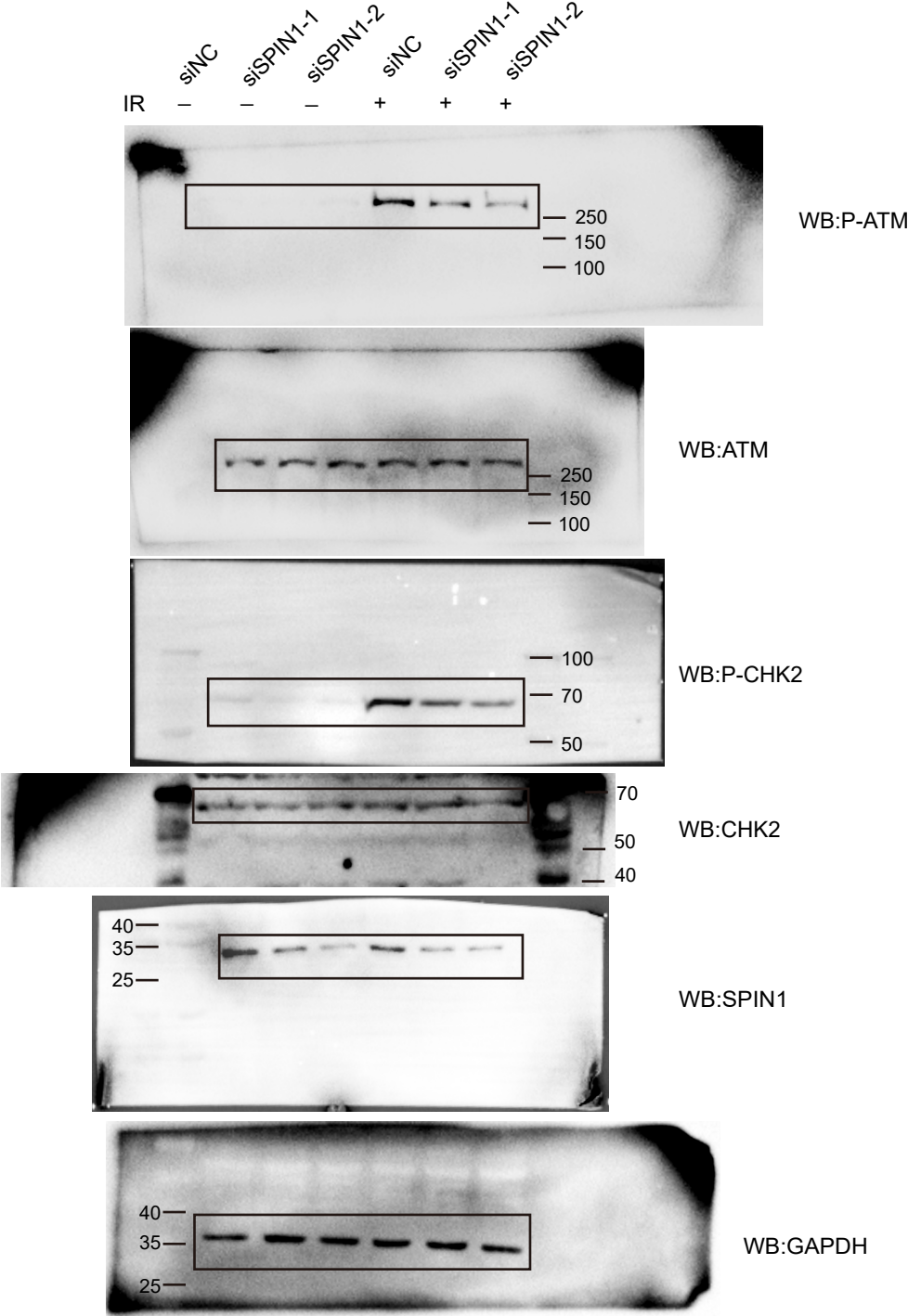

Supplement: Supplementary file 5 — Source data Fig. 3 [file 44319_2024_219_MOESM5_ESM.zip › Figure3/3k/Figure3K.pdf]

Figure 4A

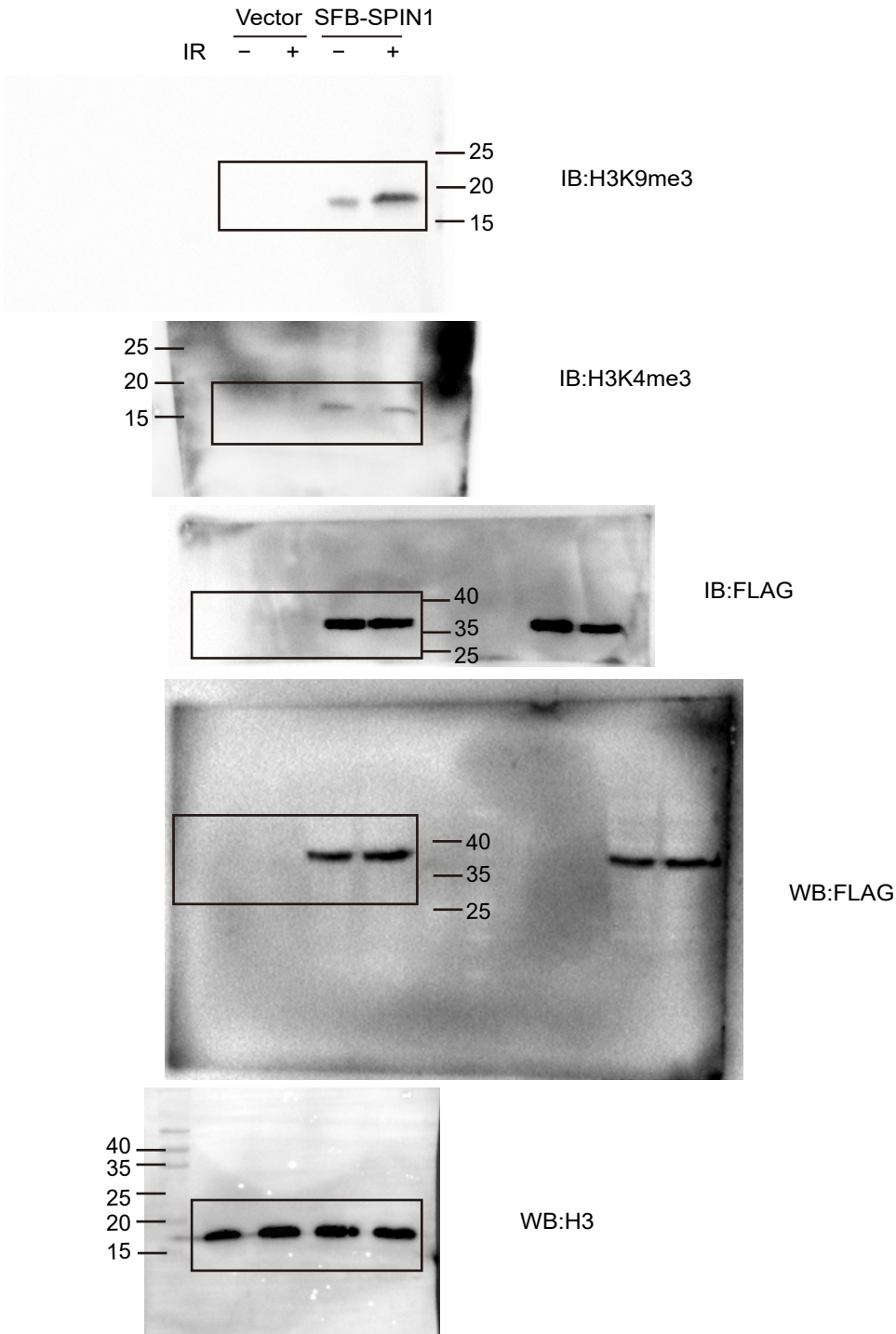

Supplement: Supplementary file 6 — Source data Fig. 4 [file 44319_2024_219_MOESM6_ESM.zip › Figure4/4A/Figure4A.pdf]

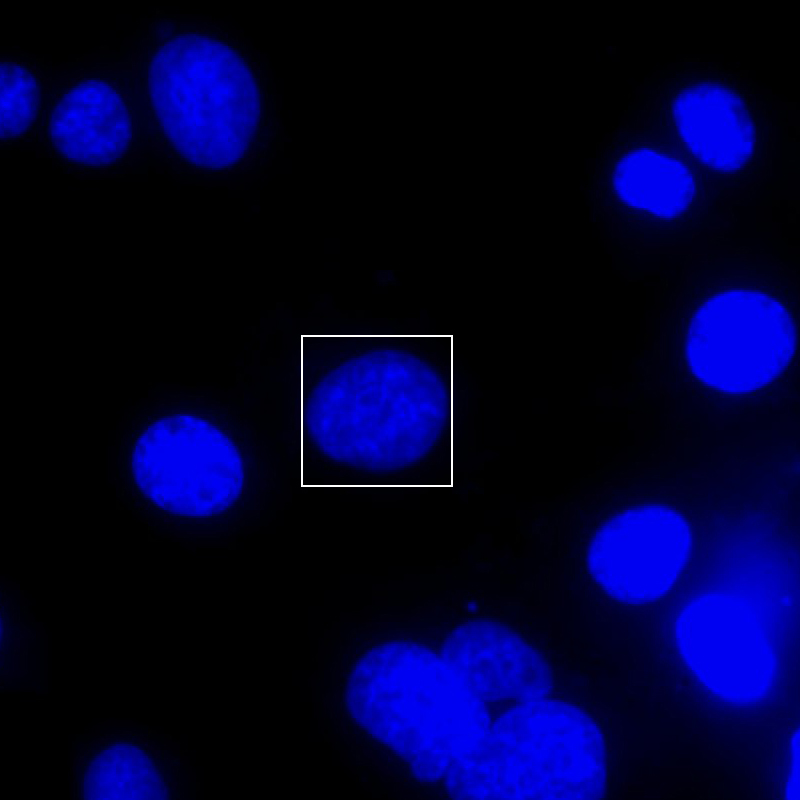

Supplement: Supplementary file 6 — Source data Fig. 4 [file 44319_2024_219_MOESM6_ESM.zip › Figure4/4B/30min/siNC-DAPI.jpg]

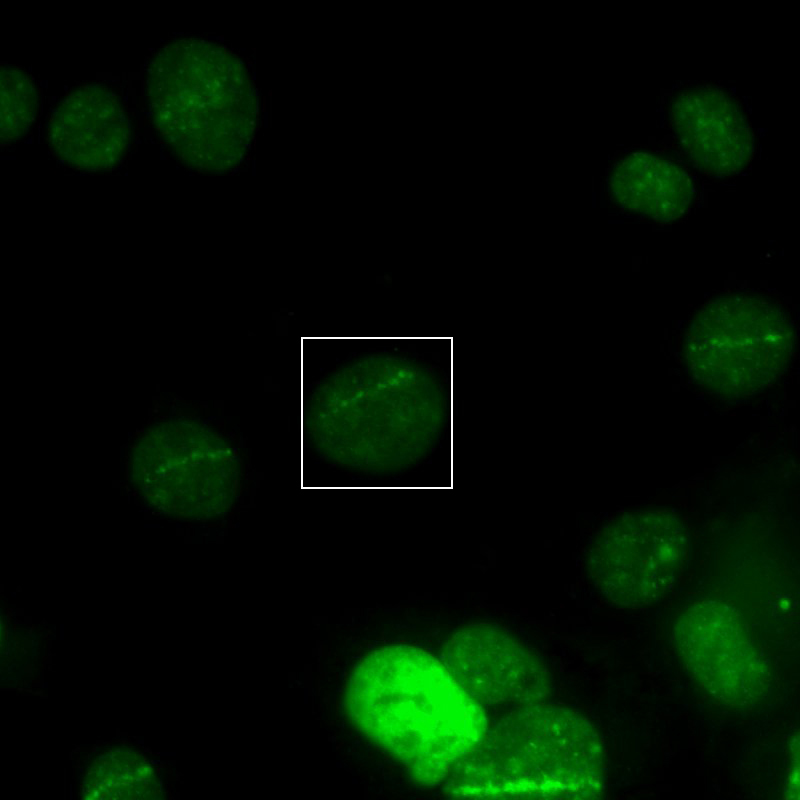

Supplement: Supplementary file 6 — Source data Fig. 4 [file 44319_2024_219_MOESM6_ESM.zip › Figure4/4B/30min/siNC-H3K9me3.jpg]

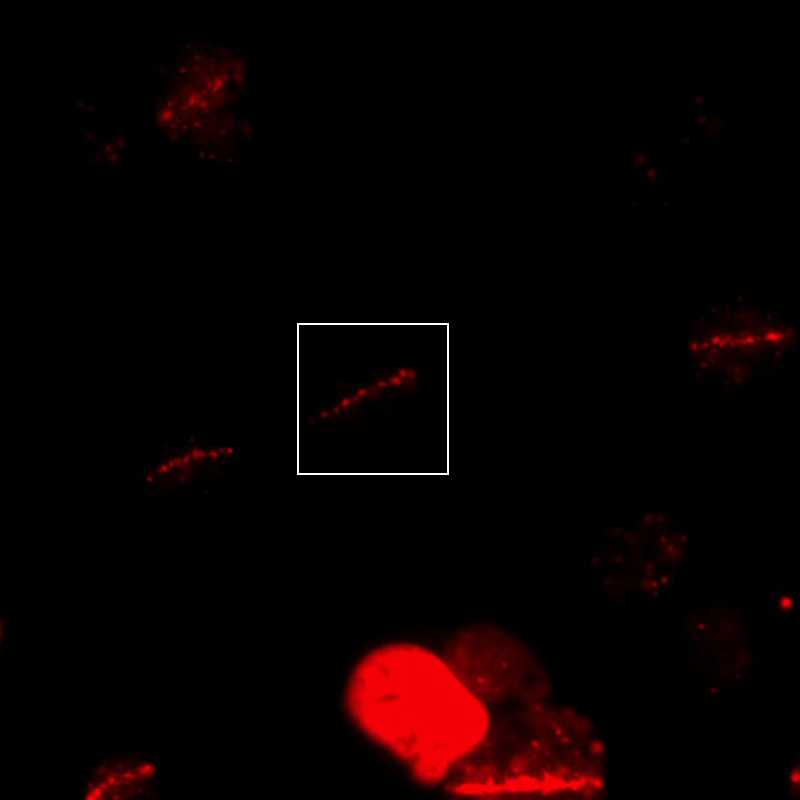

Supplement: Supplementary file 6 — Source data Fig. 4 [file 44319_2024_219_MOESM6_ESM.zip › Figure4/4B/30min/siNC-γH2AX.jpg]

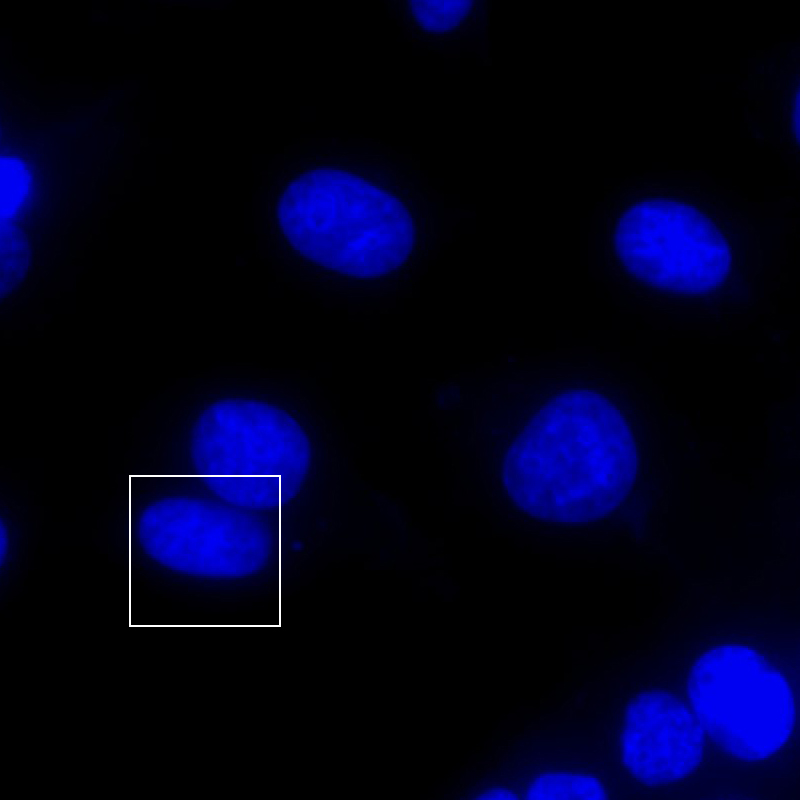

Supplement: Supplementary file 6 — Source data Fig. 4 [file 44319_2024_219_MOESM6_ESM.zip › Figure4/4B/30min/siSPIN1-1-DAPI.jpg]

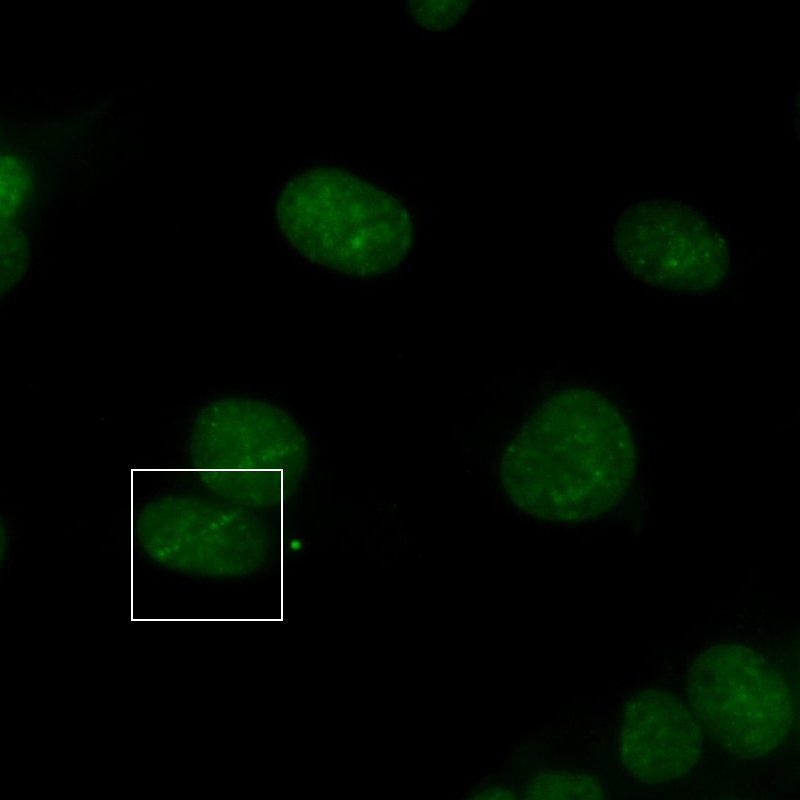

Supplement: Supplementary file 6 — Source data Fig. 4 [file 44319_2024_219_MOESM6_ESM.zip › Figure4/4B/30min/siSPIN1-1-H3K9me3.jpg]

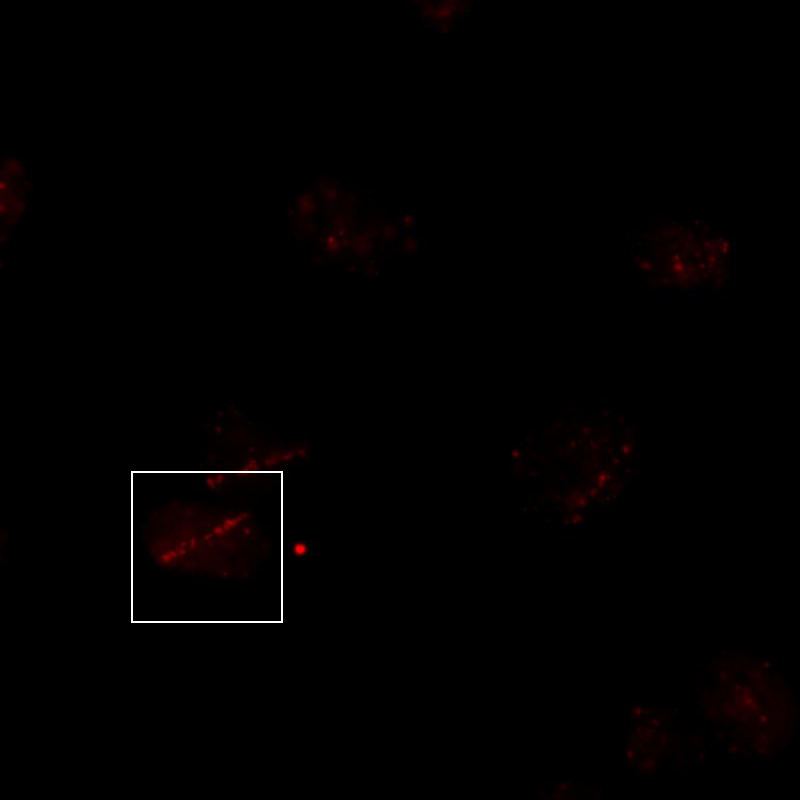

Supplement: Supplementary file 6 — Source data Fig. 4 [file 44319_2024_219_MOESM6_ESM.zip › Figure4/4B/30min/siSPIN1-1-γH2AX.jpg]

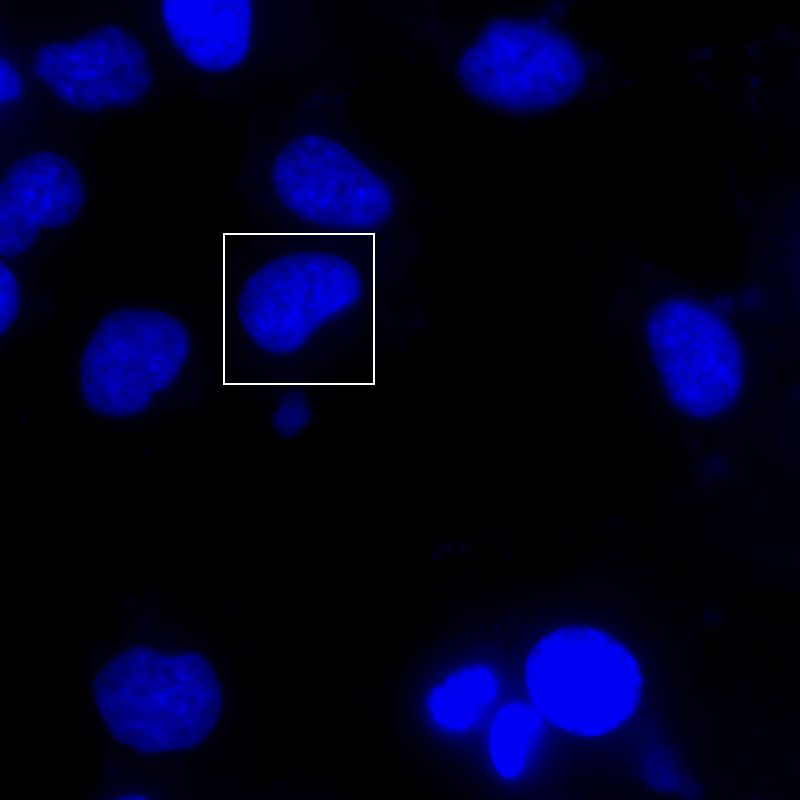

Supplement: Supplementary file 6 — Source data Fig. 4 [file 44319_2024_219_MOESM6_ESM.zip › Figure4/4B/3min/siNC-DAPI.jpg]

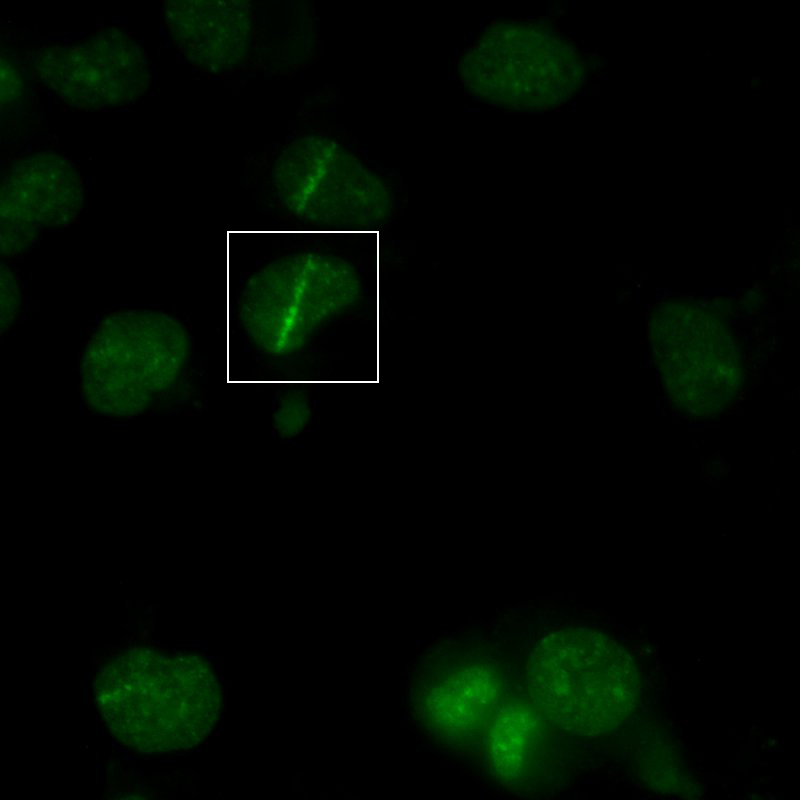

Supplement: Supplementary file 6 — Source data Fig. 4 [file 44319_2024_219_MOESM6_ESM.zip › Figure4/4B/3min/siNC-H3K9me3.jpg]

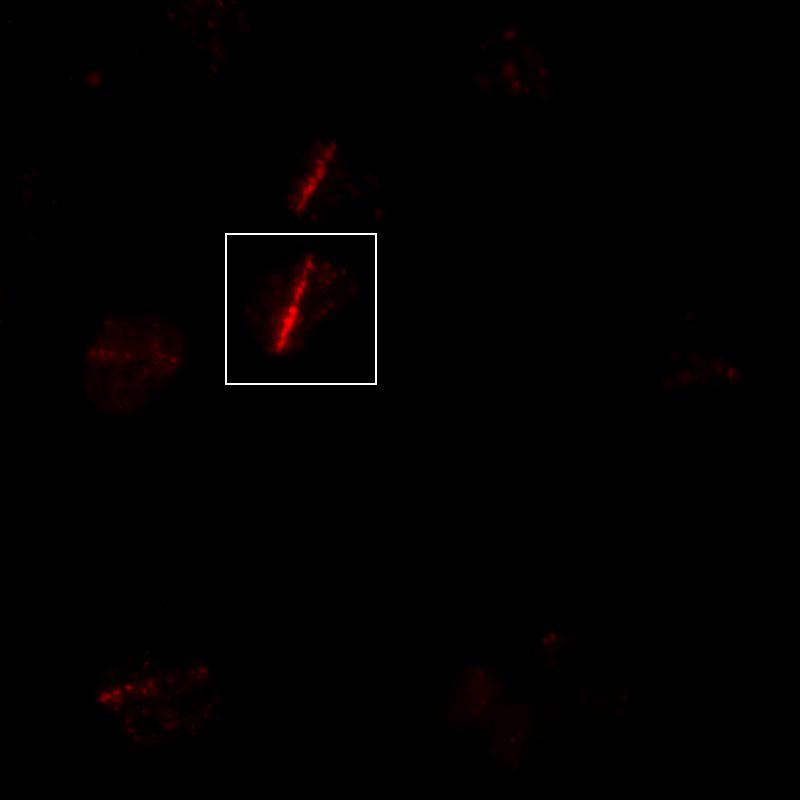

Supplement: Supplementary file 6 — Source data Fig. 4 [file 44319_2024_219_MOESM6_ESM.zip › Figure4/4B/3min/siNC-γH2AX.jpg]

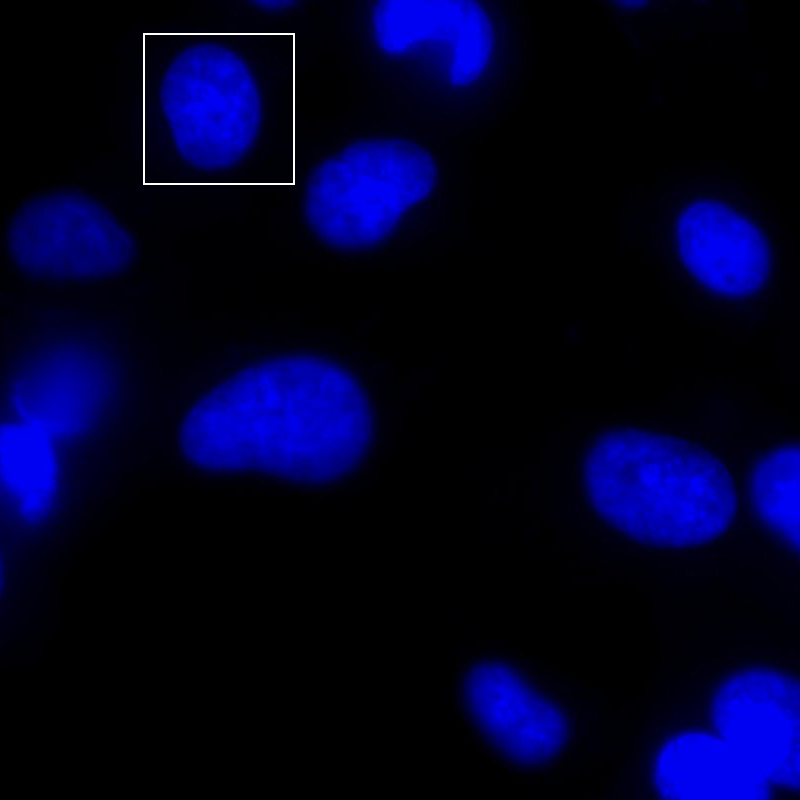

Supplement: Supplementary file 6 — Source data Fig. 4 [file 44319_2024_219_MOESM6_ESM.zip › Figure4/4B/3min/siSPIN1-1-DAPI.jpg]

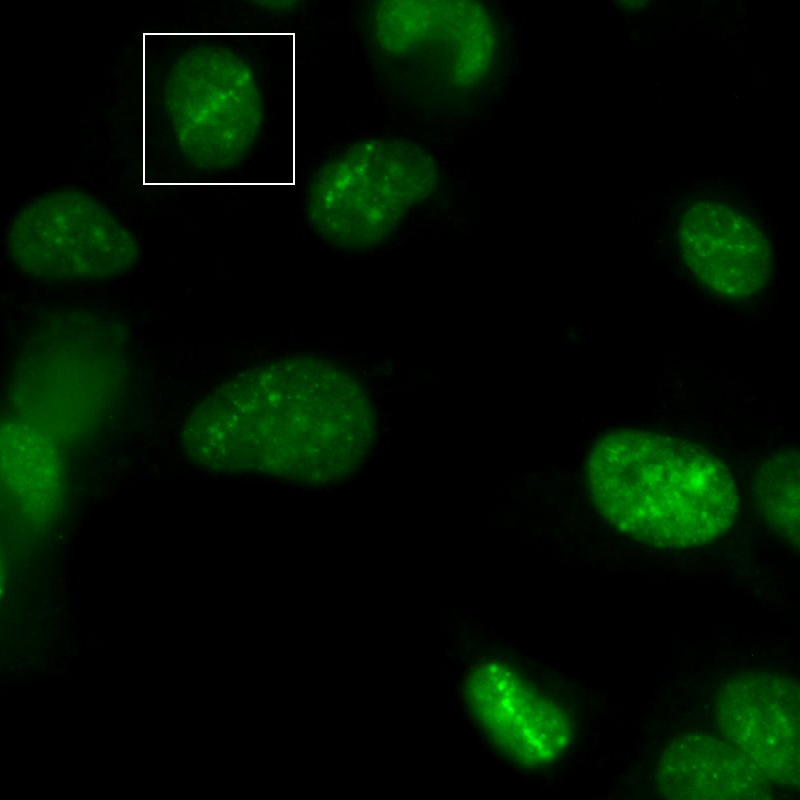

Supplement: Supplementary file 6 — Source data Fig. 4 [file 44319_2024_219_MOESM6_ESM.zip › Figure4/4B/3min/siSPIN1-1-H3K9me3.jpg]

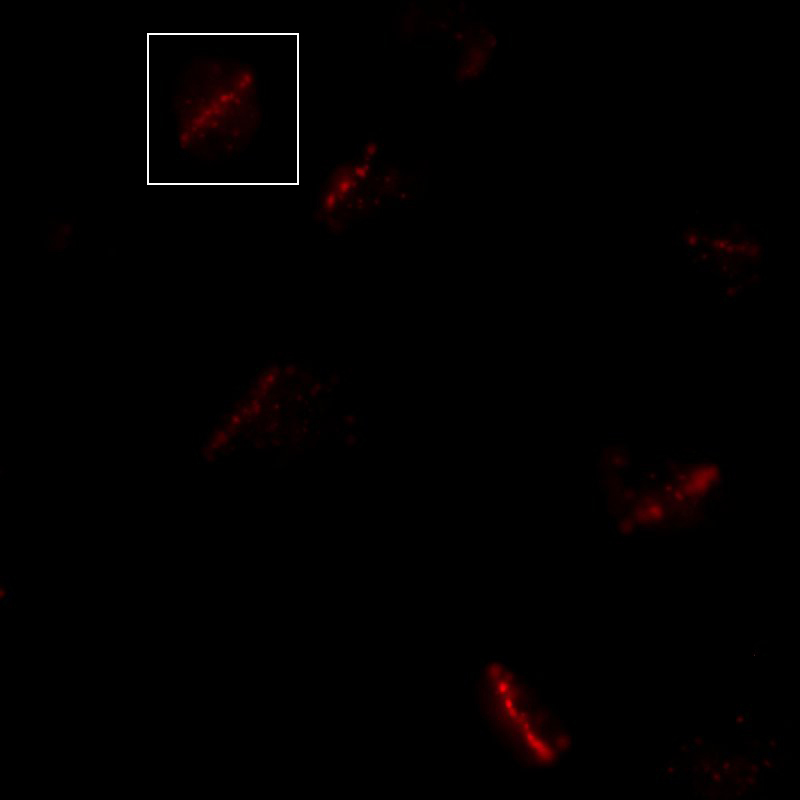

Supplement: Supplementary file 6 — Source data Fig. 4 [file 44319_2024_219_MOESM6_ESM.zip › Figure4/4B/3min/siSPIN1-1-γH2AX.jpg]

Figure4 D

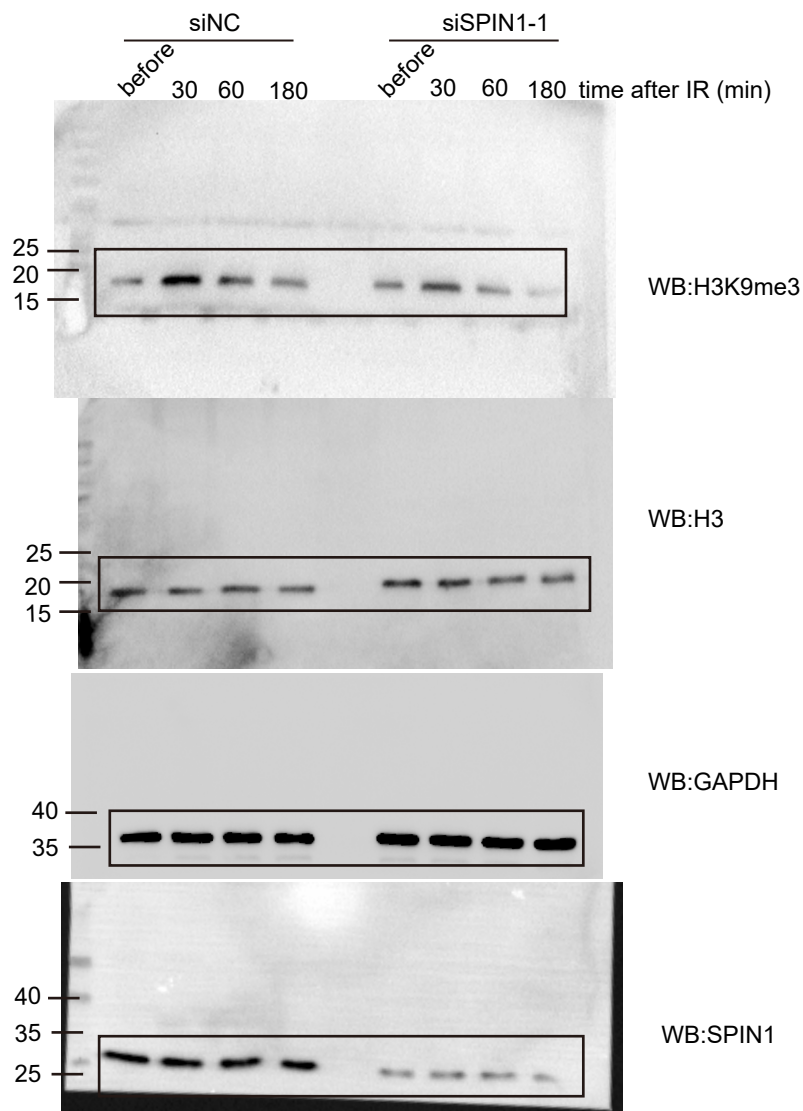

Supplement: Supplementary file 6 — Source data Fig. 4 [file 44319_2024_219_MOESM6_ESM.zip › Figure4/4D/Figure4D.pdf]

Figure4 E

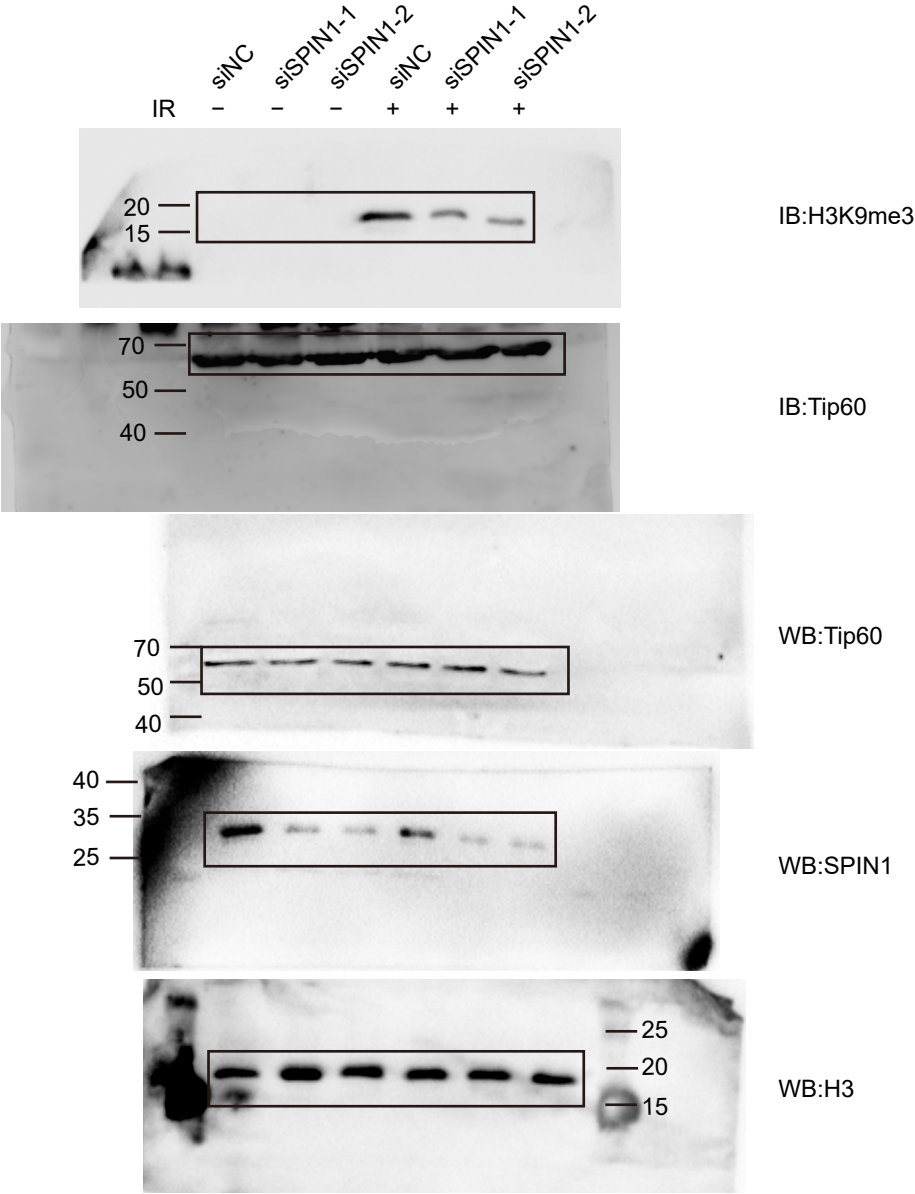

Supplement: Supplementary file 6 — Source data Fig. 4 [file 44319_2024_219_MOESM6_ESM.zip › Figure4/4E/Figure4E.pdf]

Figure4 F

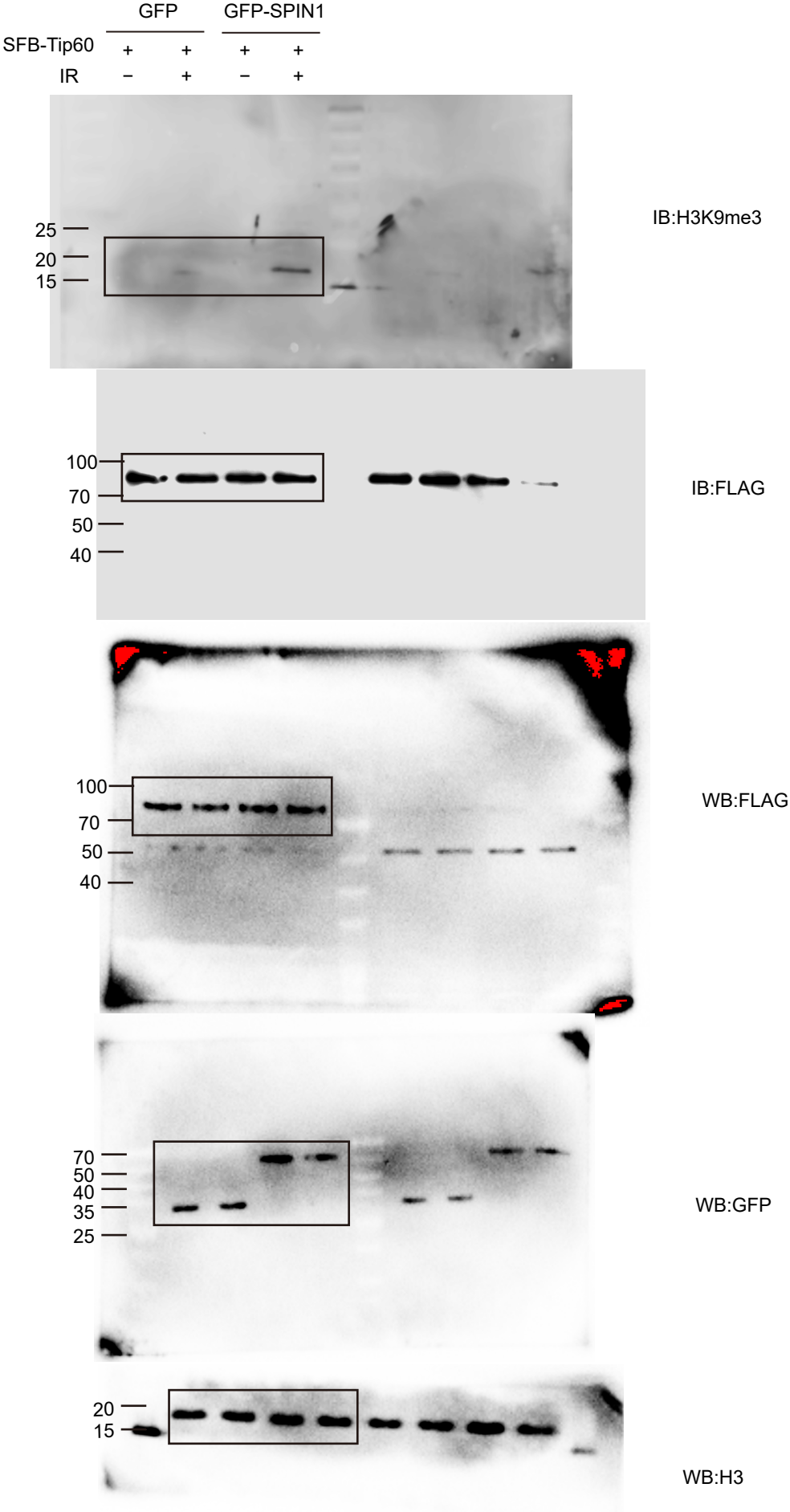

Supplement: Supplementary file 6 — Source data Fig. 4 [file 44319_2024_219_MOESM6_ESM.zip › Figure4/4F/Figure4F.pdf]

Figure4 G

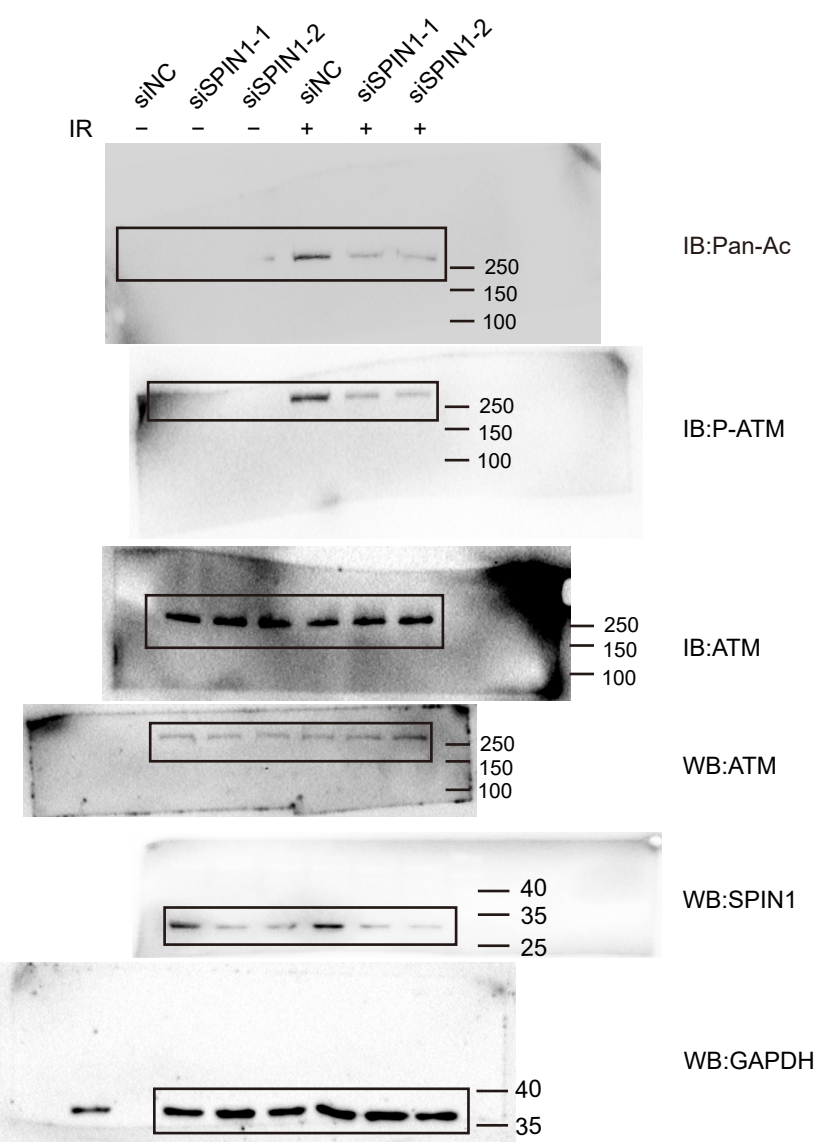

Supplement: Supplementary file 6 — Source data Fig. 4 [file 44319_2024_219_MOESM6_ESM.zip › Figure4/4G/Figure4G.pdf]

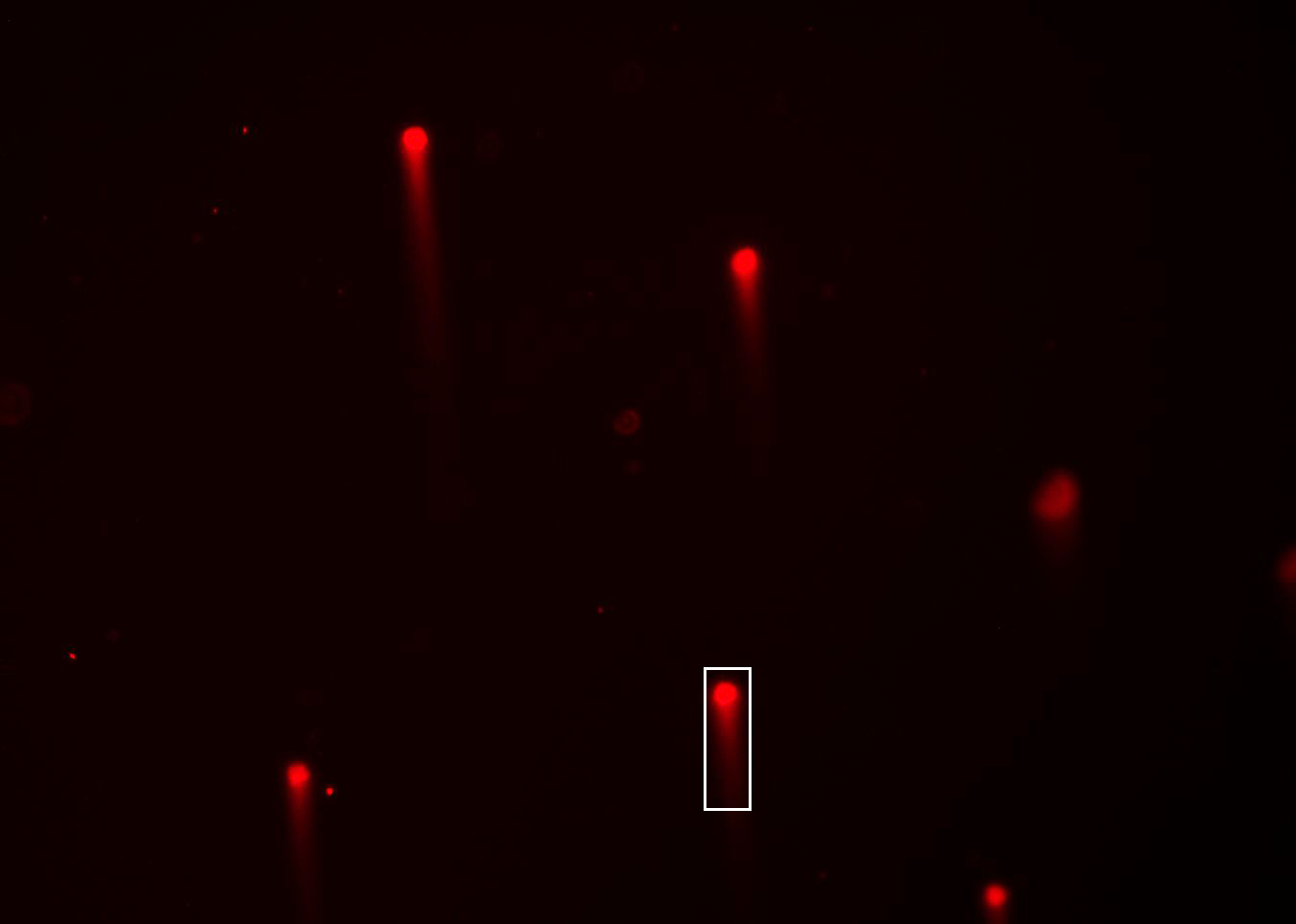

Supplement: Supplementary file 7 — Source data Fig. 5 [file 44319_2024_219_MOESM7_ESM.zip › Figure5/5A/0h/siNC.tif]

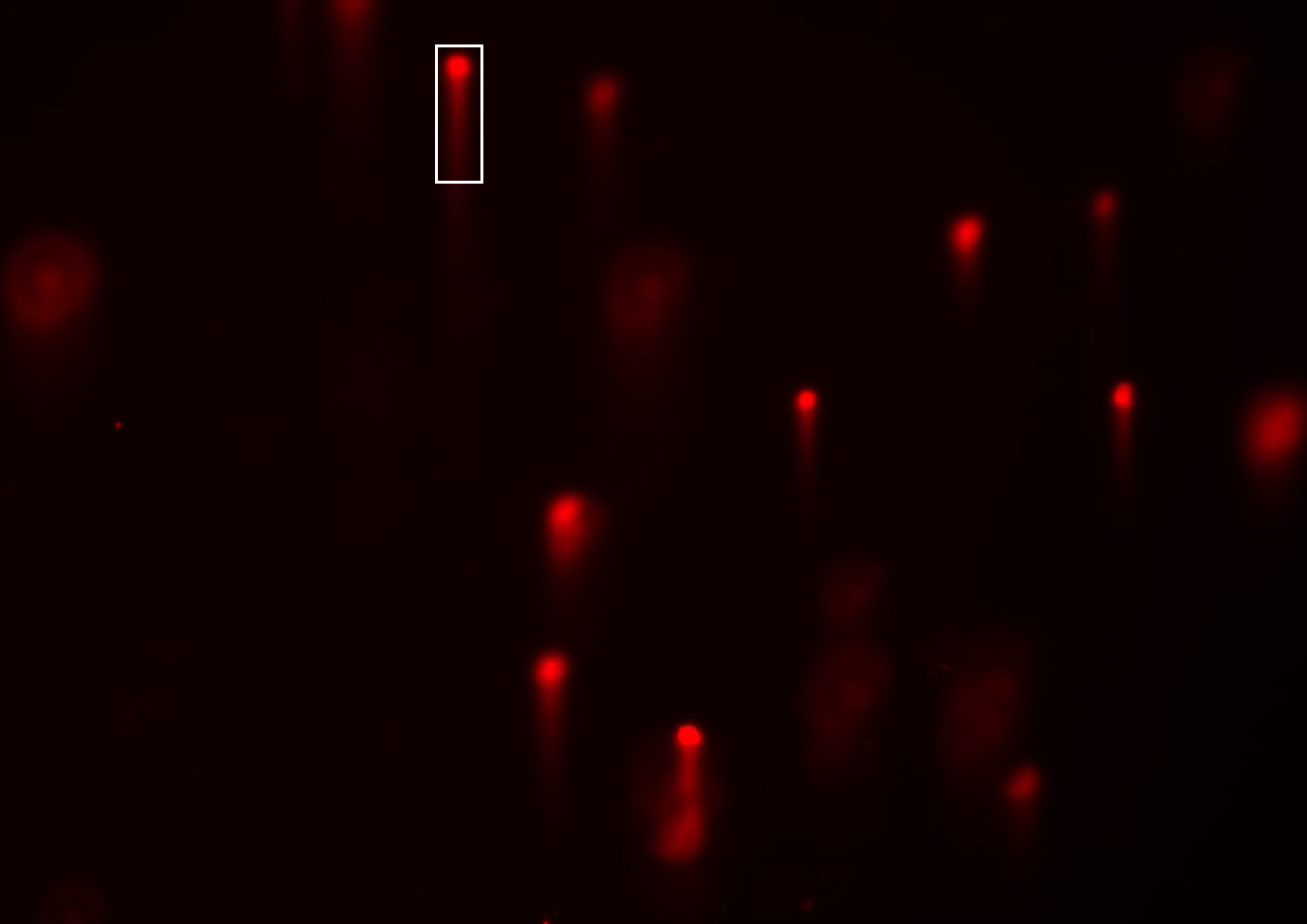

Supplement: Supplementary file 7 — Source data Fig. 5 [file 44319_2024_219_MOESM7_ESM.zip › Figure5/5A/0h/siSPIN1-1+SPIN1-△1-50.tif]

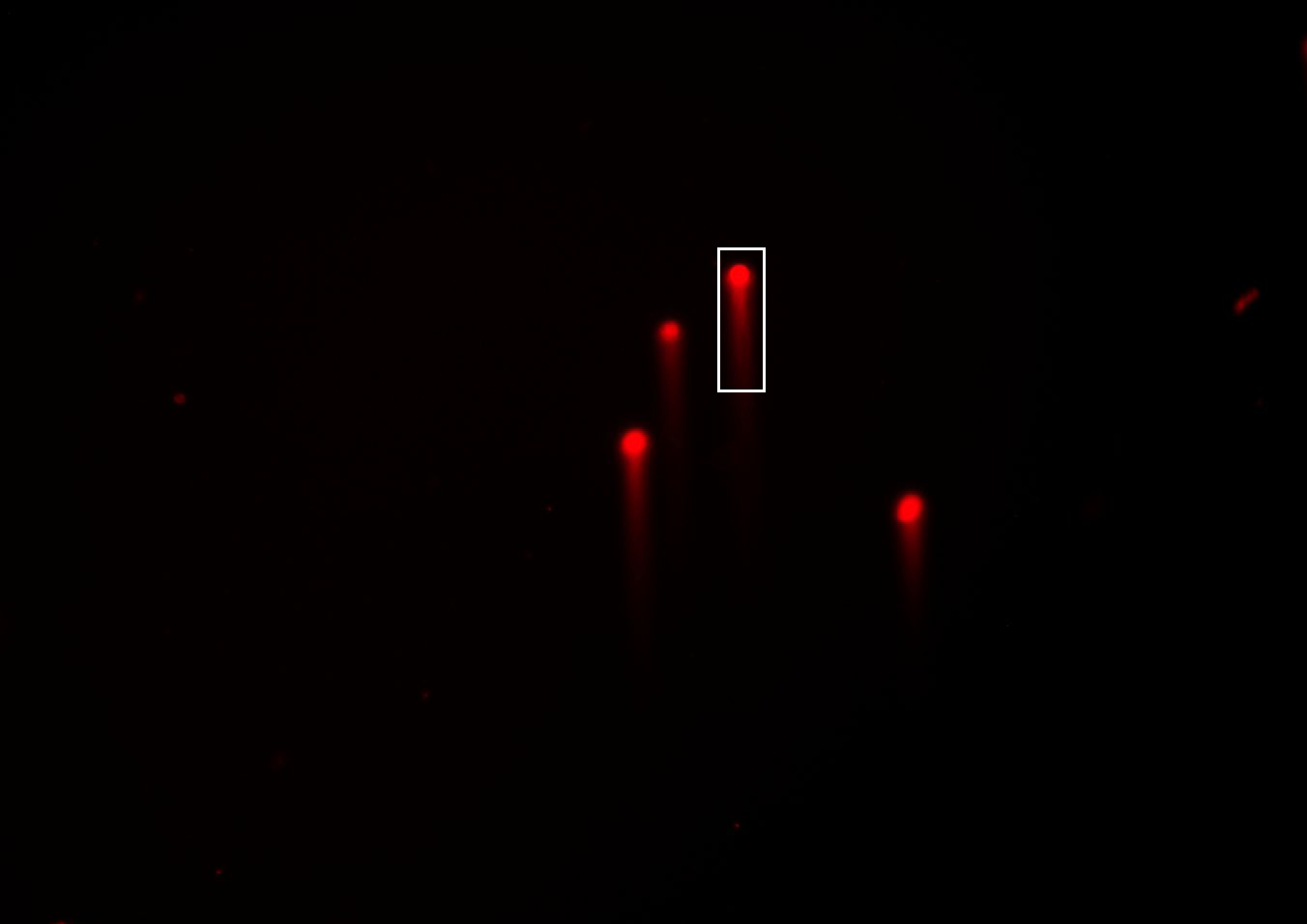

Supplement: Supplementary file 7 — Source data Fig. 5 [file 44319_2024_219_MOESM7_ESM.zip › Figure5/5A/0h/siSPIN1-1+SPIN1-△51-125.tif]

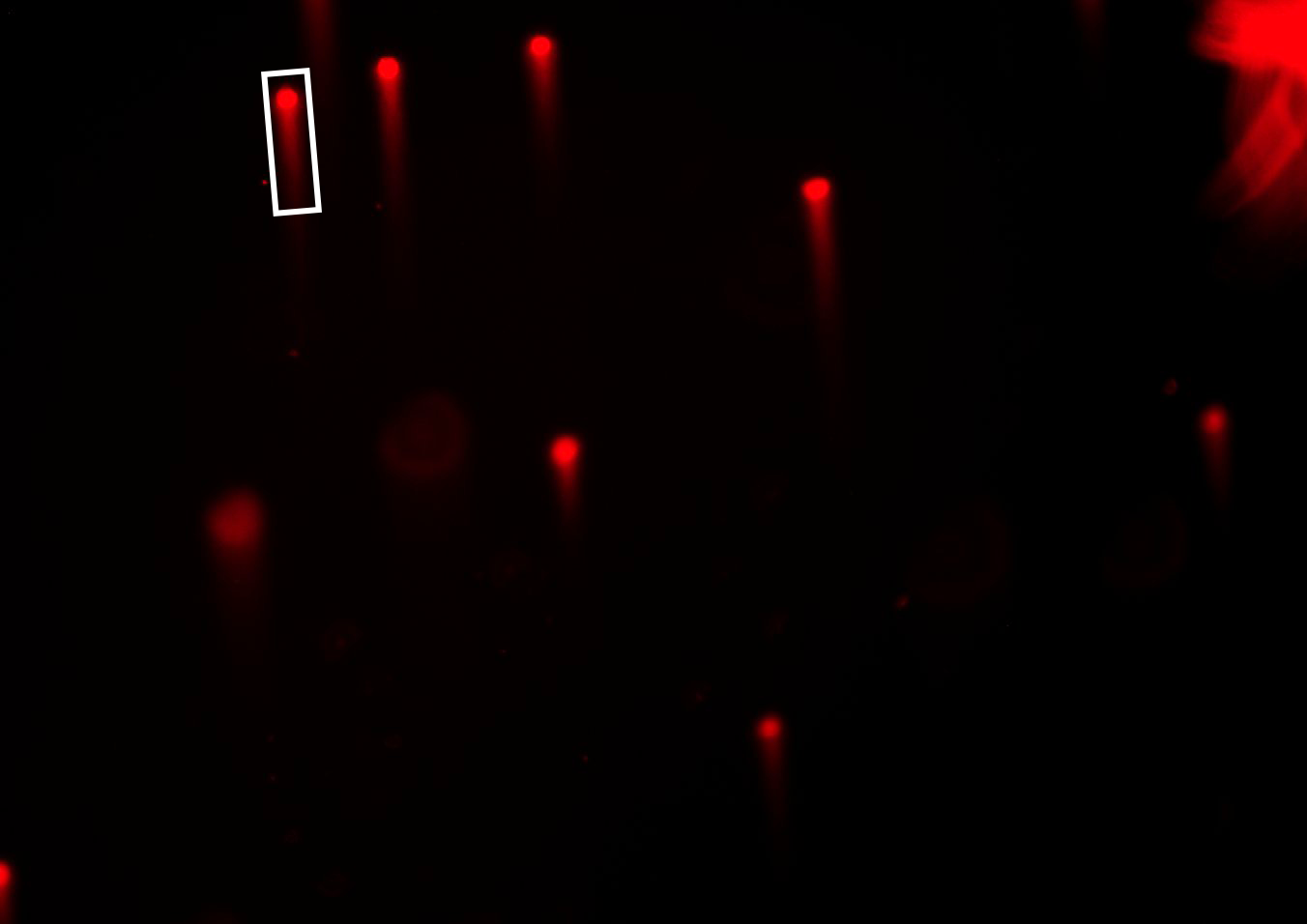

Supplement: Supplementary file 7 — Source data Fig. 5 [file 44319_2024_219_MOESM7_ESM.zip › Figure5/5A/0h/siSPIN1-1+Vector.tif]

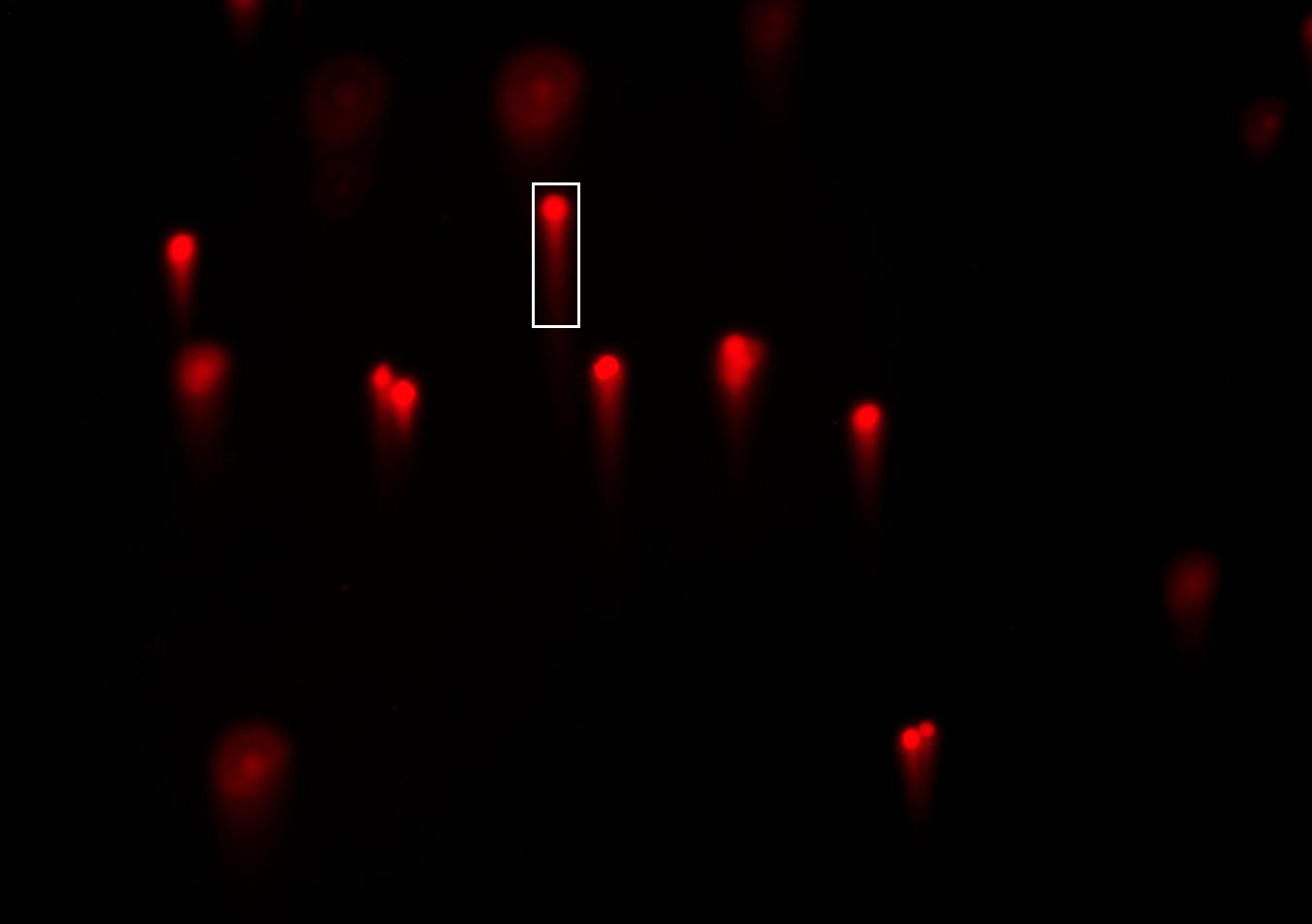

Supplement: Supplementary file 7 — Source data Fig. 5 [file 44319_2024_219_MOESM7_ESM.zip › Figure5/5A/0h/siSPIN1-1+WT.tif]

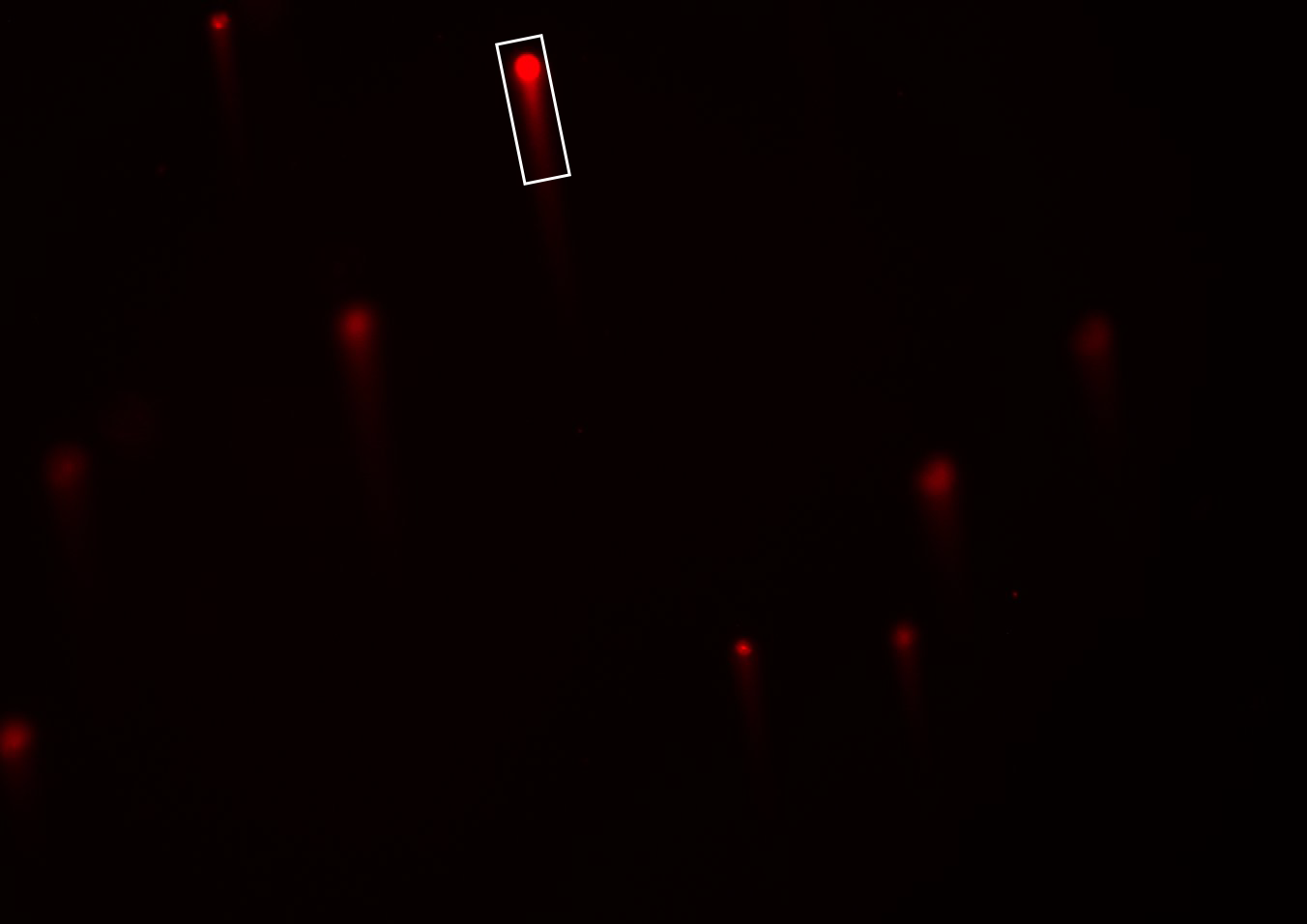

Supplement: Supplementary file 7 — Source data Fig. 5 [file 44319_2024_219_MOESM7_ESM.zip › Figure5/5A/0h/siSPIN1.tif]

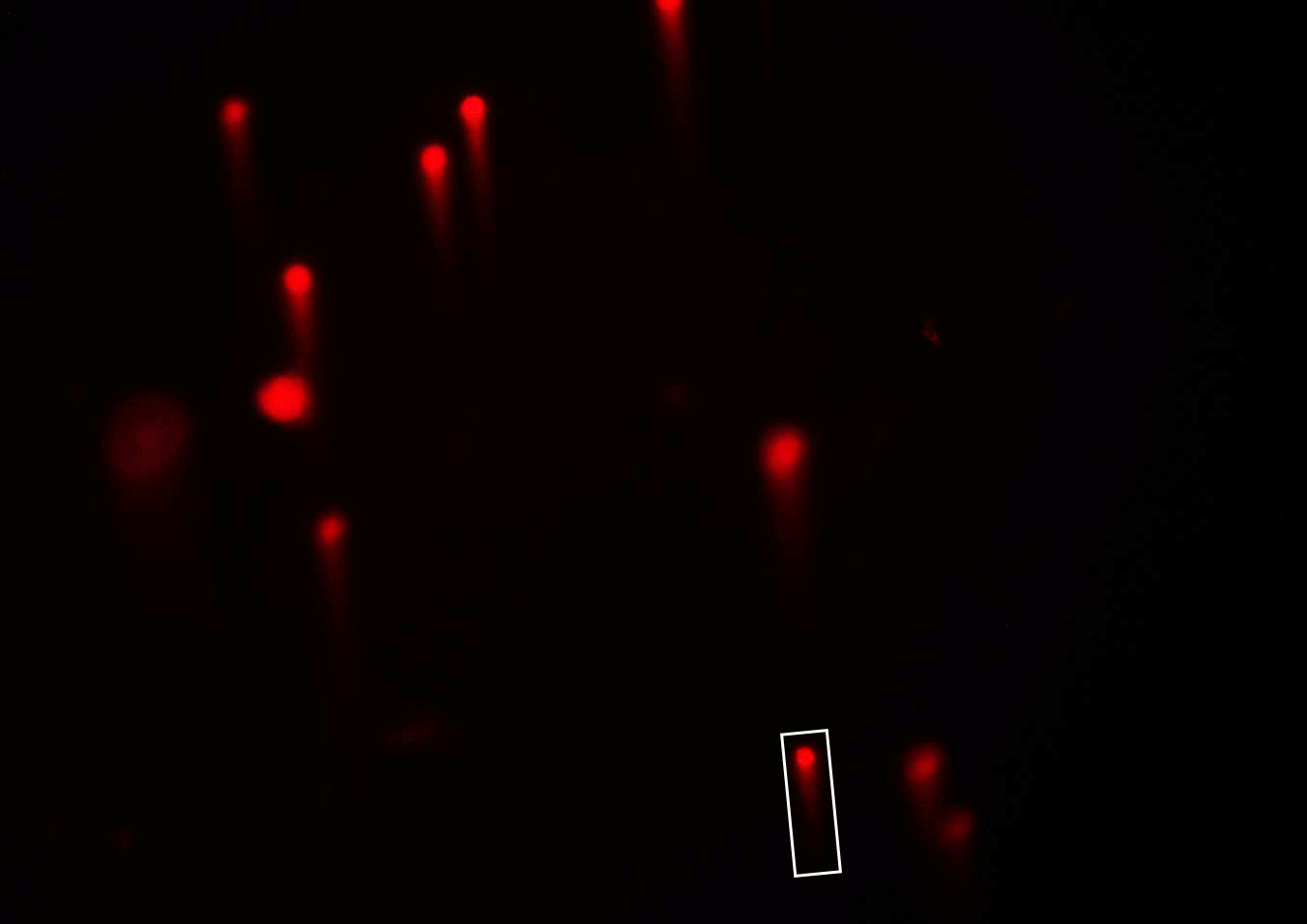

Supplement: Supplementary file 7 — Source data Fig. 5 [file 44319_2024_219_MOESM7_ESM.zip › Figure5/5A/2h/siNC.tif]

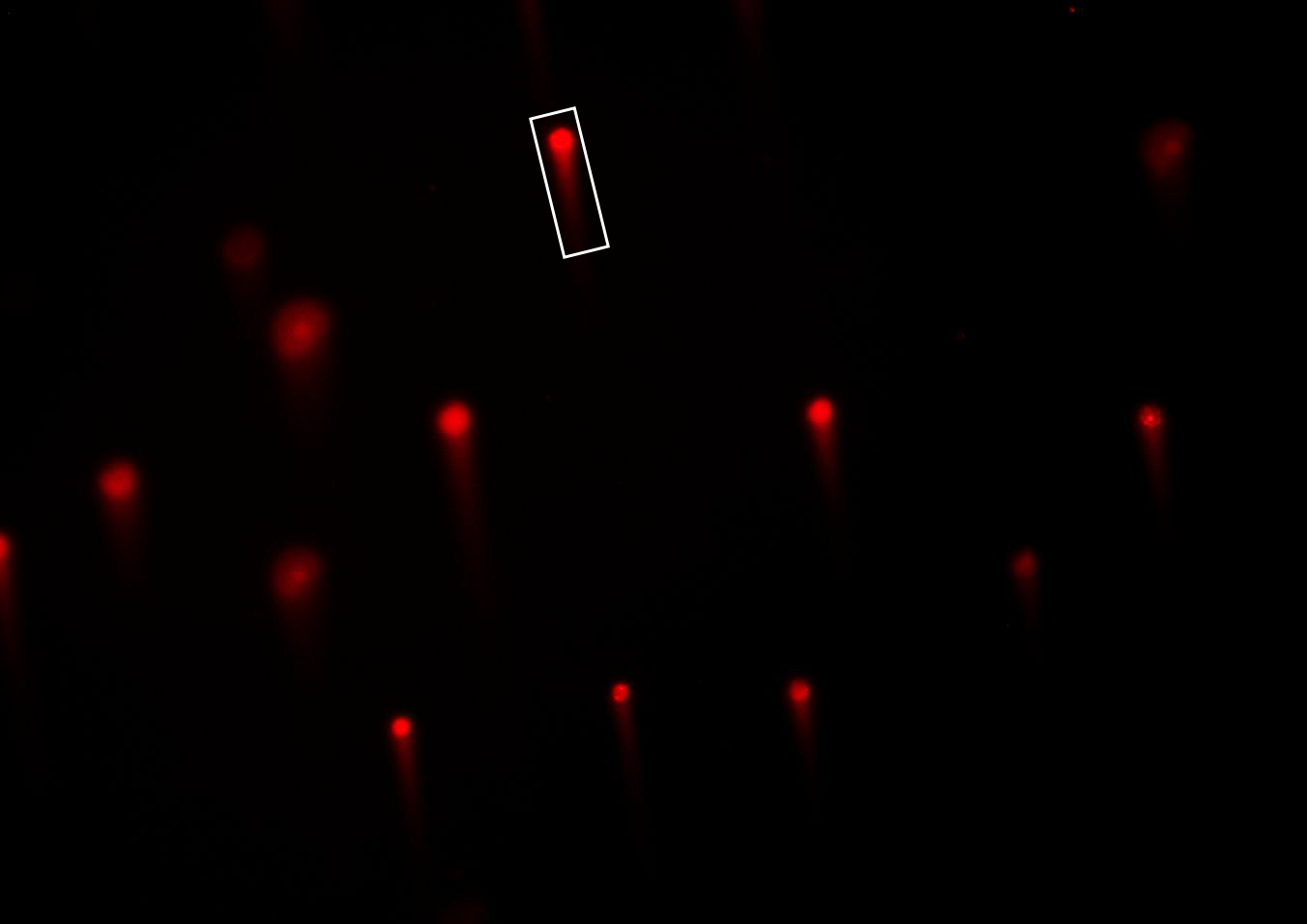

Supplement: Supplementary file 7 — Source data Fig. 5 [file 44319_2024_219_MOESM7_ESM.zip › Figure5/5A/2h/siPSIN1.tif]

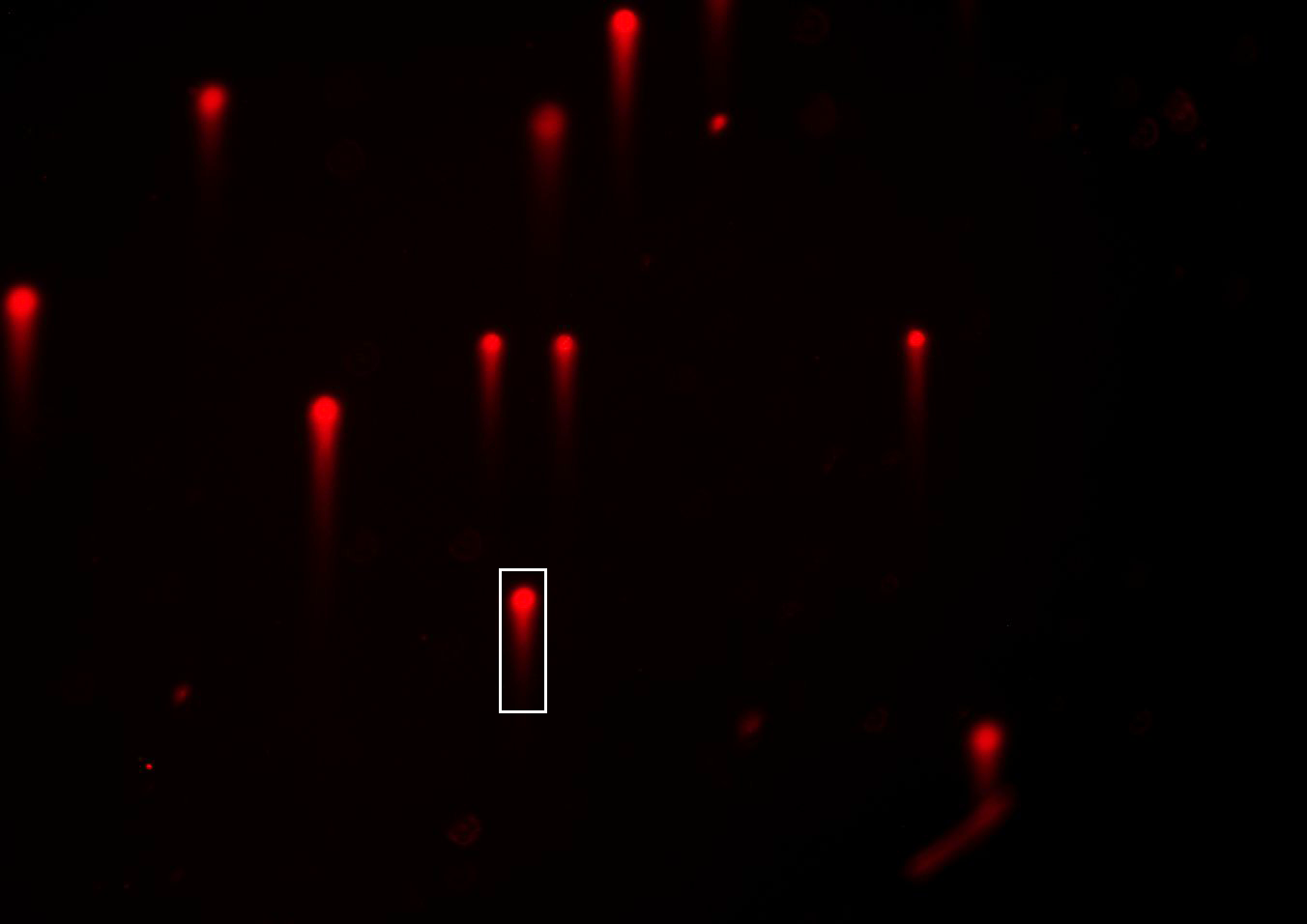

Supplement: Supplementary file 7 — Source data Fig. 5 [file 44319_2024_219_MOESM7_ESM.zip › Figure5/5A/2h/siSPIN1-1+SPIN1-△1-50.tif]

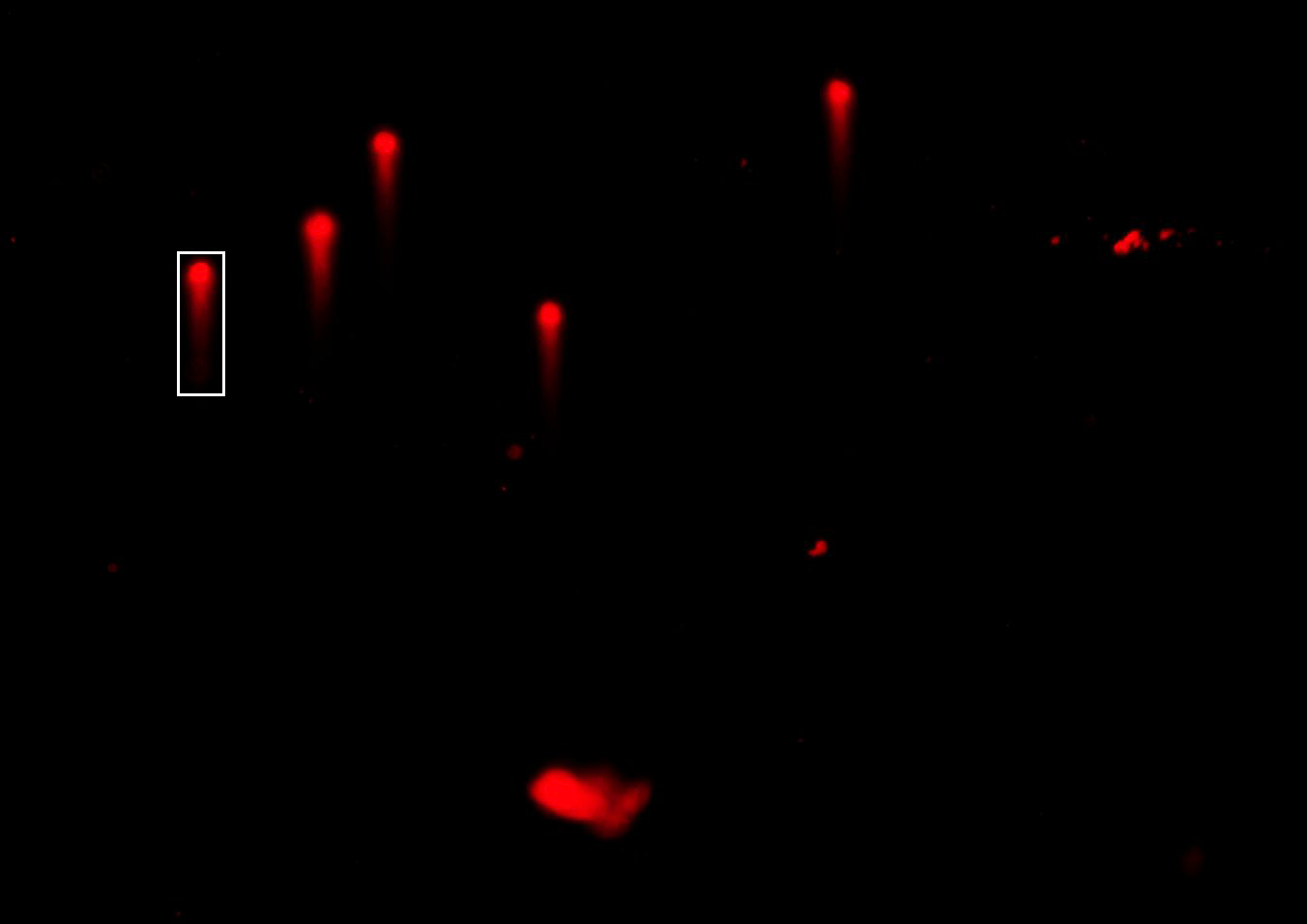

Supplement: Supplementary file 7 — Source data Fig. 5 [file 44319_2024_219_MOESM7_ESM.zip › Figure5/5A/2h/siSPIN1-1+SPIN1-△51-125.tif]

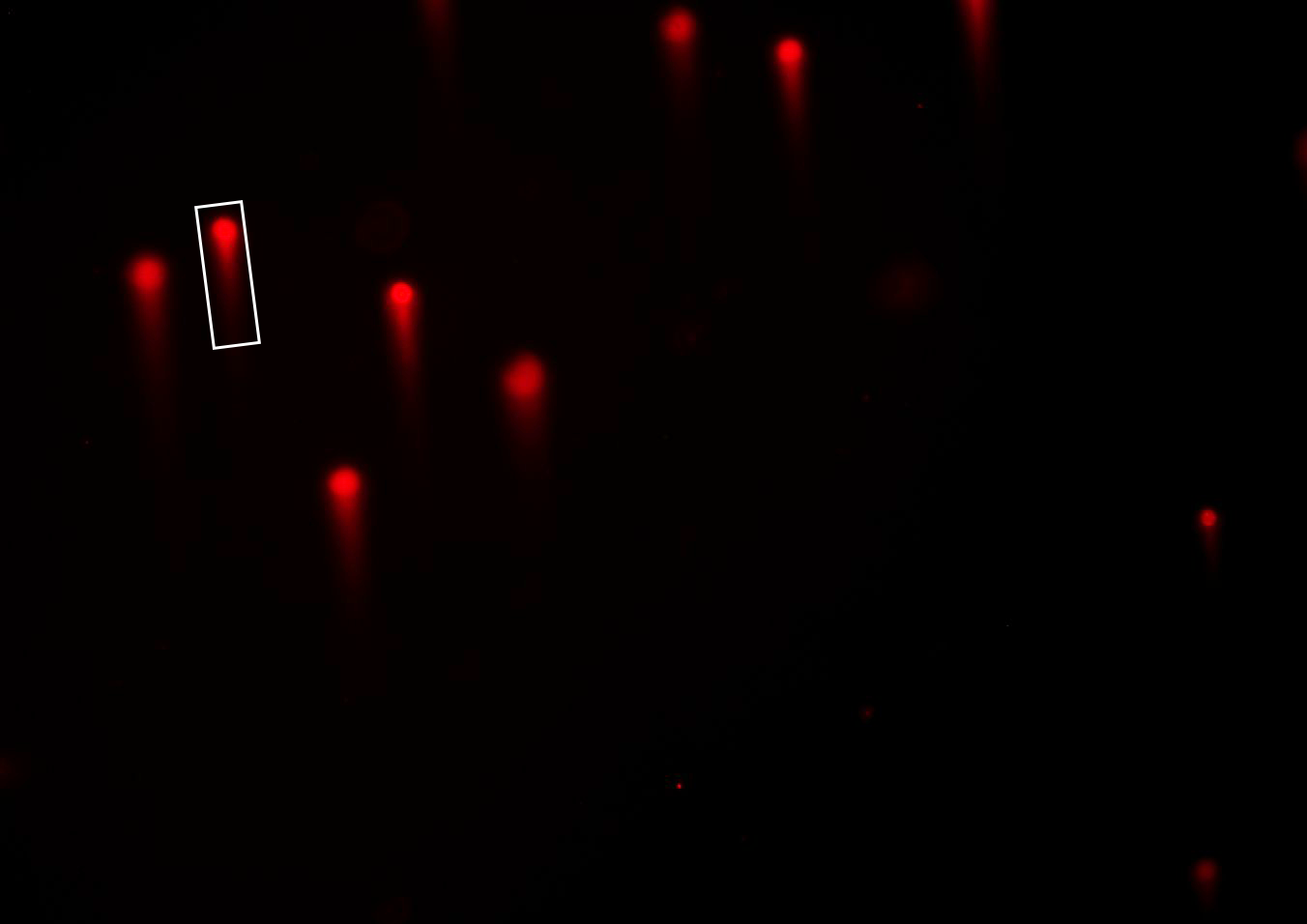

Supplement: Supplementary file 7 — Source data Fig. 5 [file 44319_2024_219_MOESM7_ESM.zip › Figure5/5A/2h/siSPIN1-1+Vector.tif]

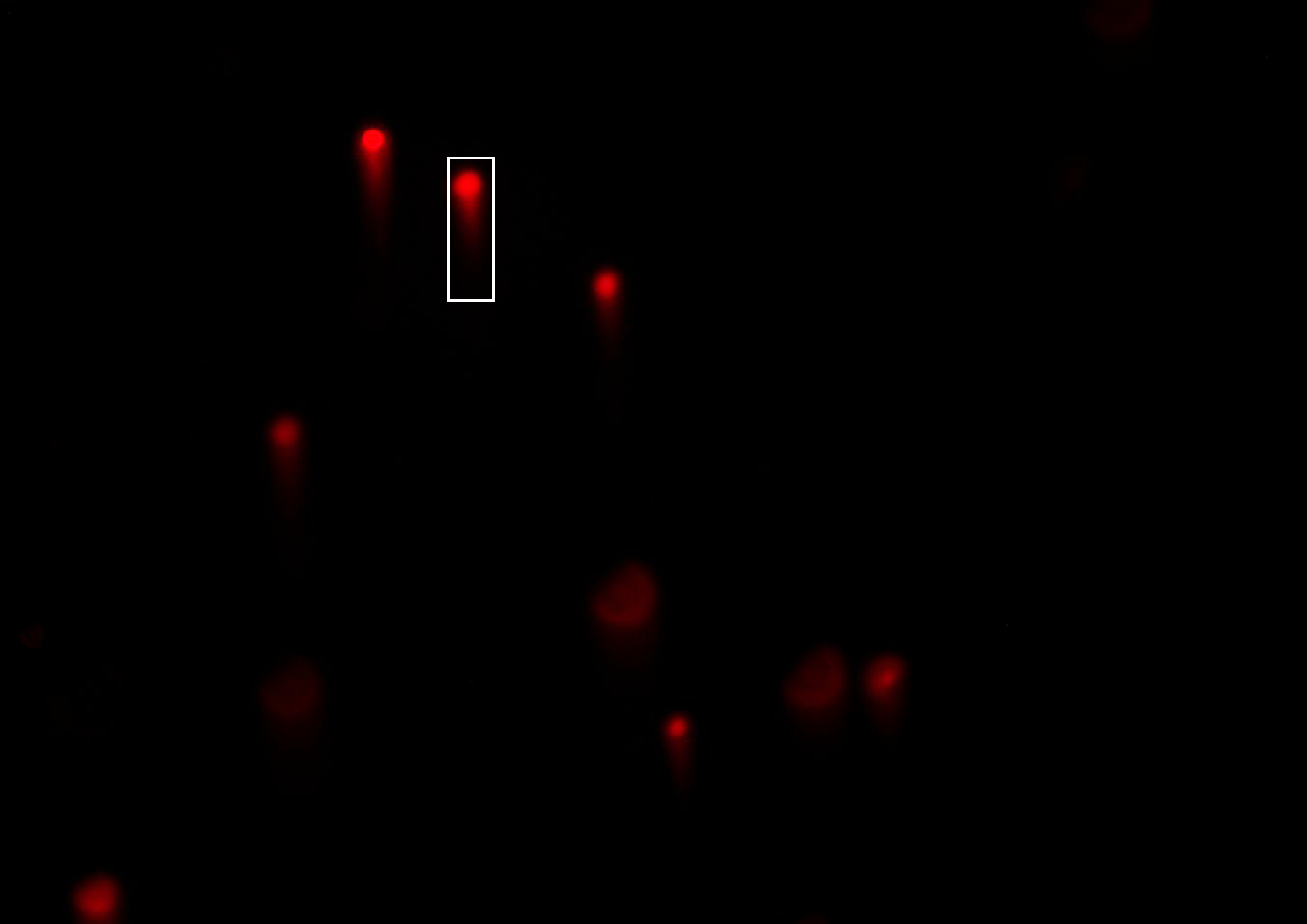

Supplement: Supplementary file 7 — Source data Fig. 5 [file 44319_2024_219_MOESM7_ESM.zip › Figure5/5A/2h/siSPIN1-1+WT.tif]

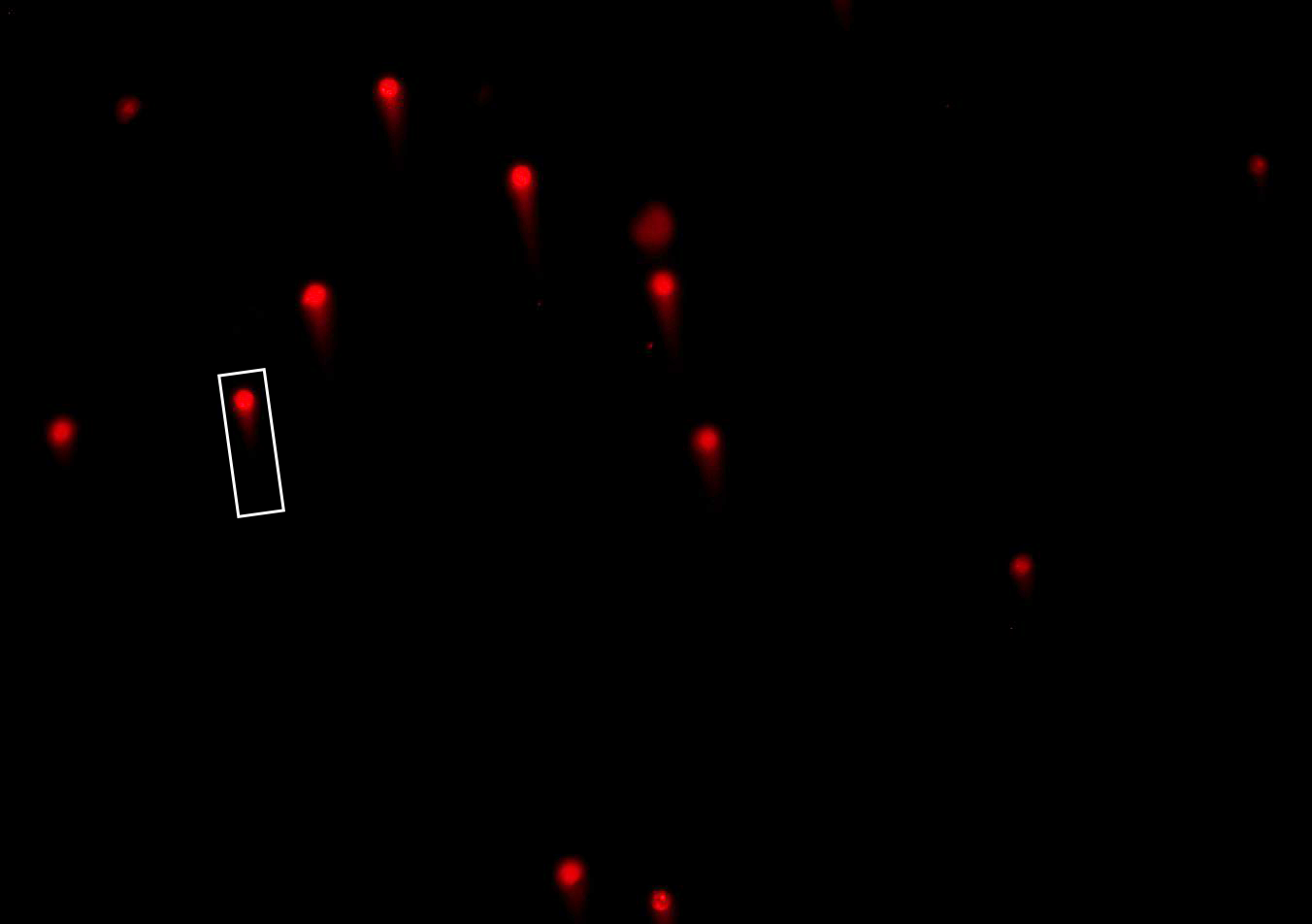

Supplement: Supplementary file 7 — Source data Fig. 5 [file 44319_2024_219_MOESM7_ESM.zip › Figure5/5A/4h/siNC.tif]

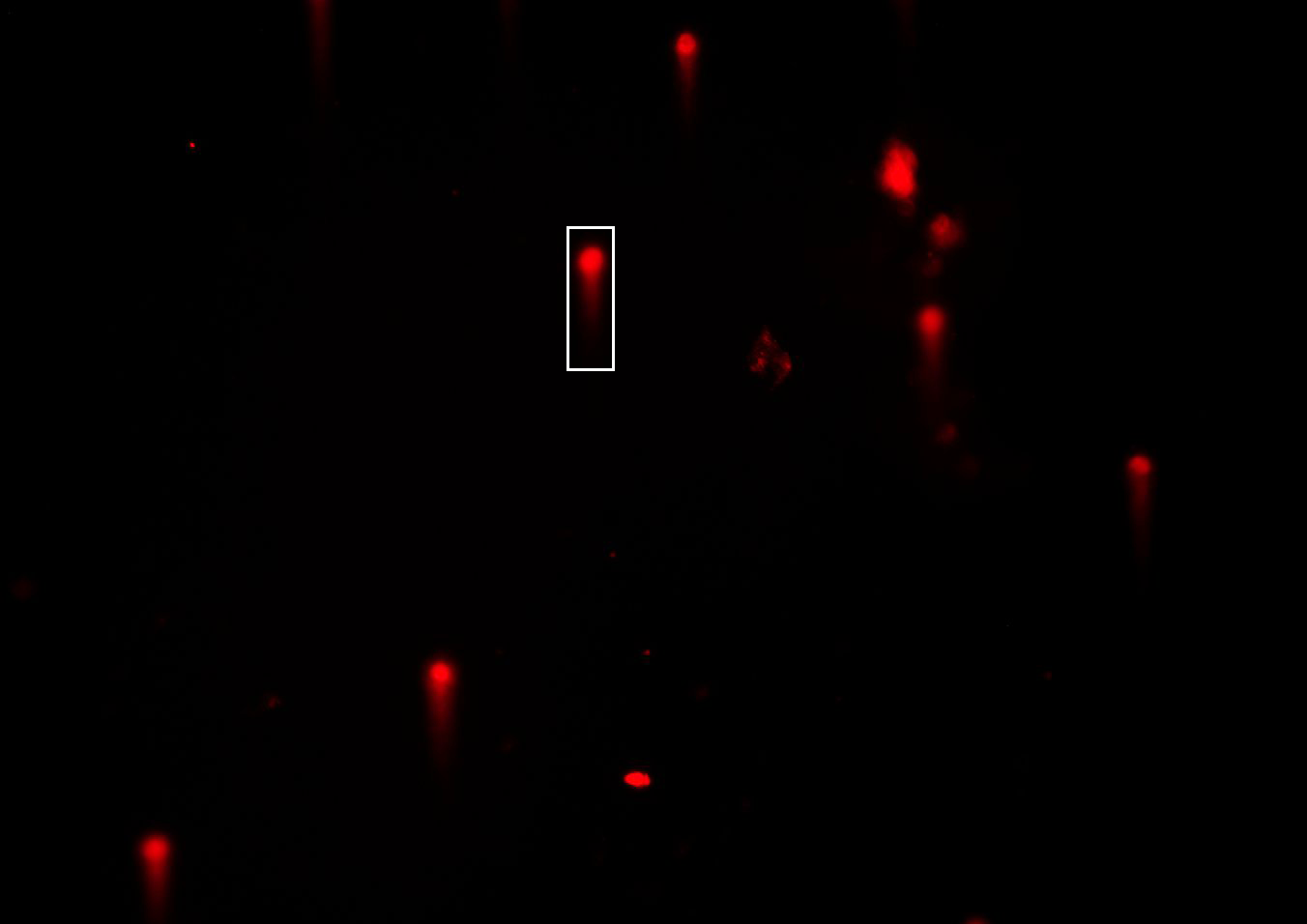

Supplement: Supplementary file 7 — Source data Fig. 5 [file 44319_2024_219_MOESM7_ESM.zip › Figure5/5A/4h/siSPIN1-1+SPIN1-△1-50.tif]

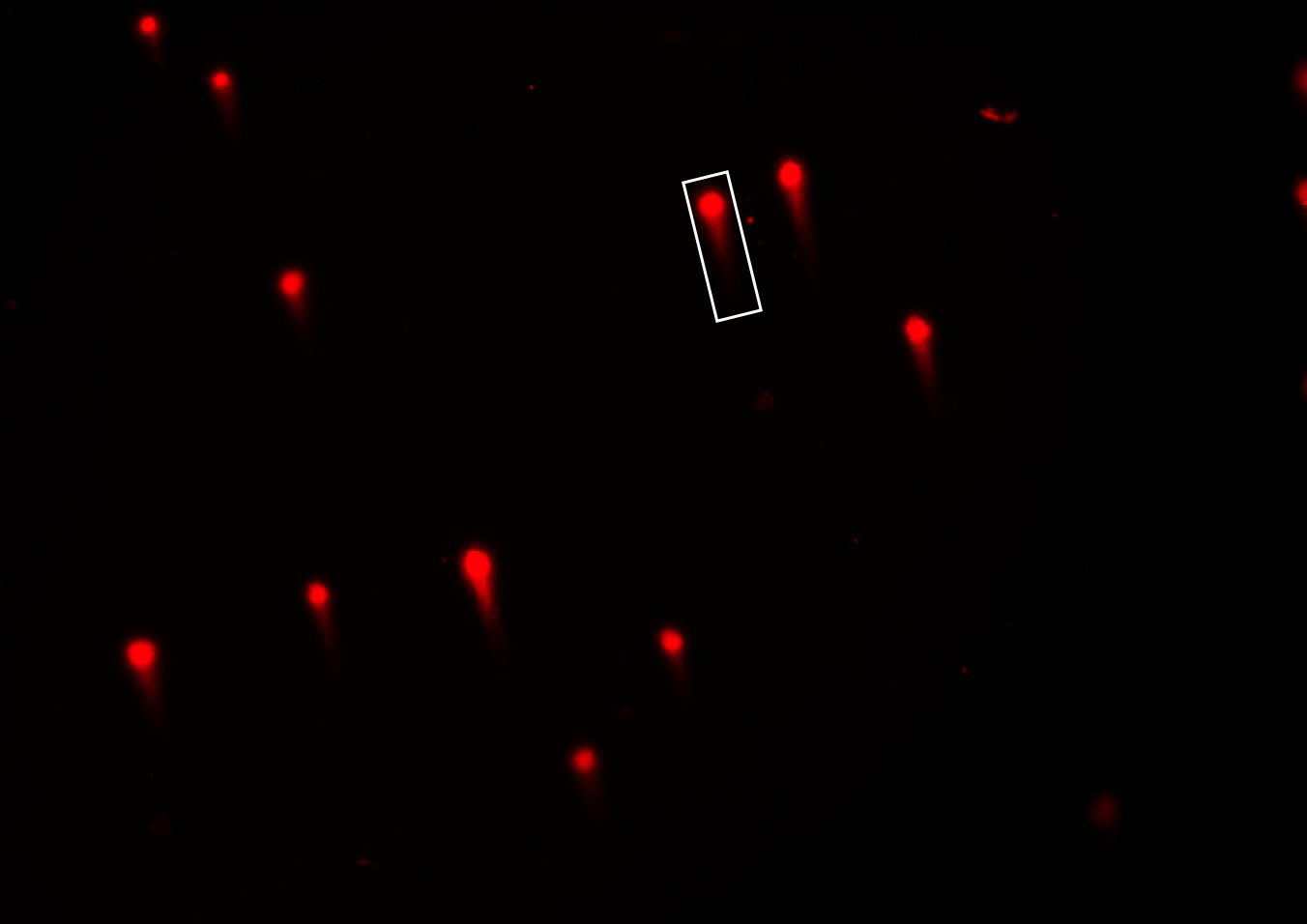

Supplement: Supplementary file 7 — Source data Fig. 5 [file 44319_2024_219_MOESM7_ESM.zip › Figure5/5A/4h/siSPIN1-1+SPIN1-△51-125.tif]

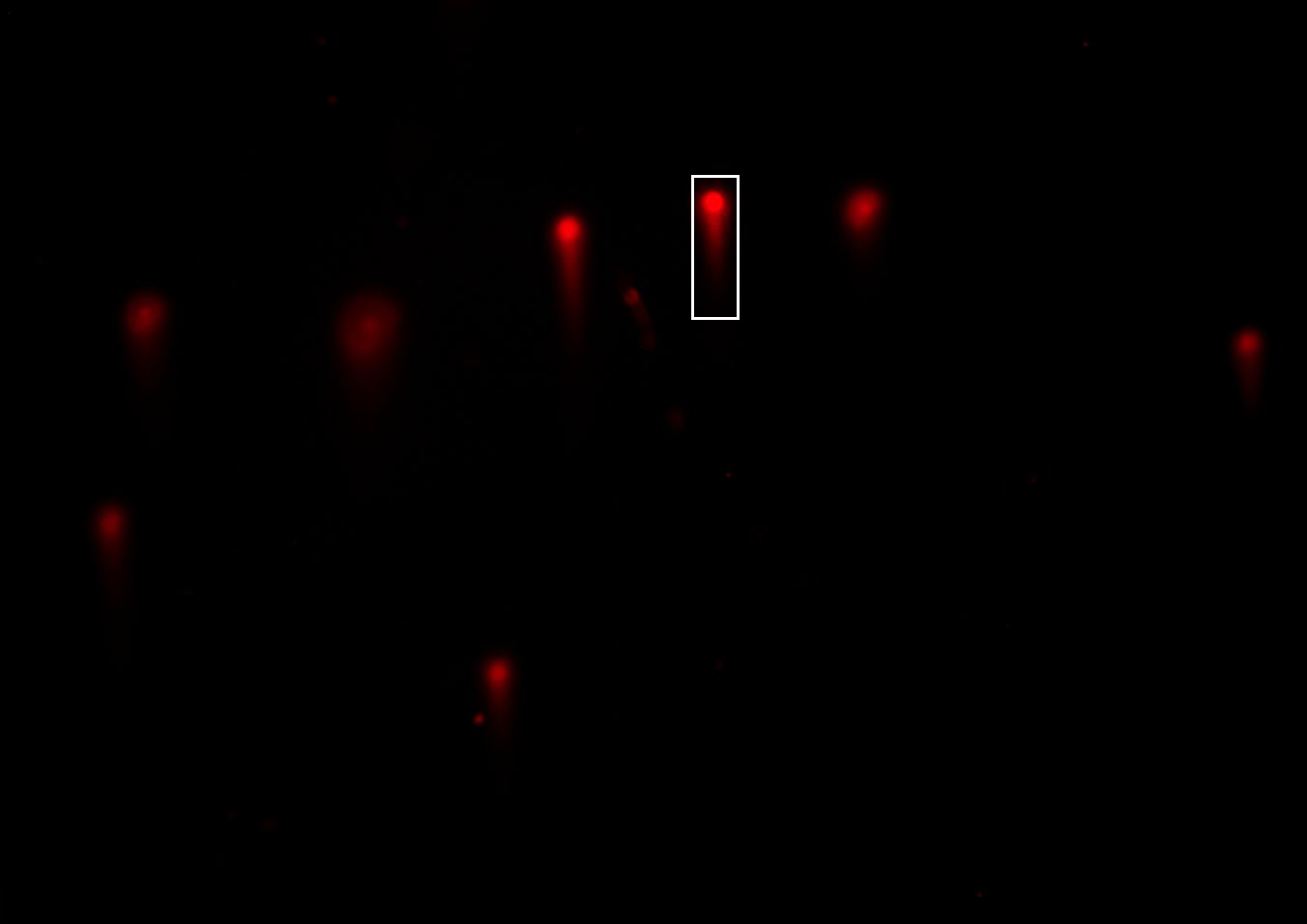

Supplement: Supplementary file 7 — Source data Fig. 5 [file 44319_2024_219_MOESM7_ESM.zip › Figure5/5A/4h/siSPIN1-1+Vector.tif]

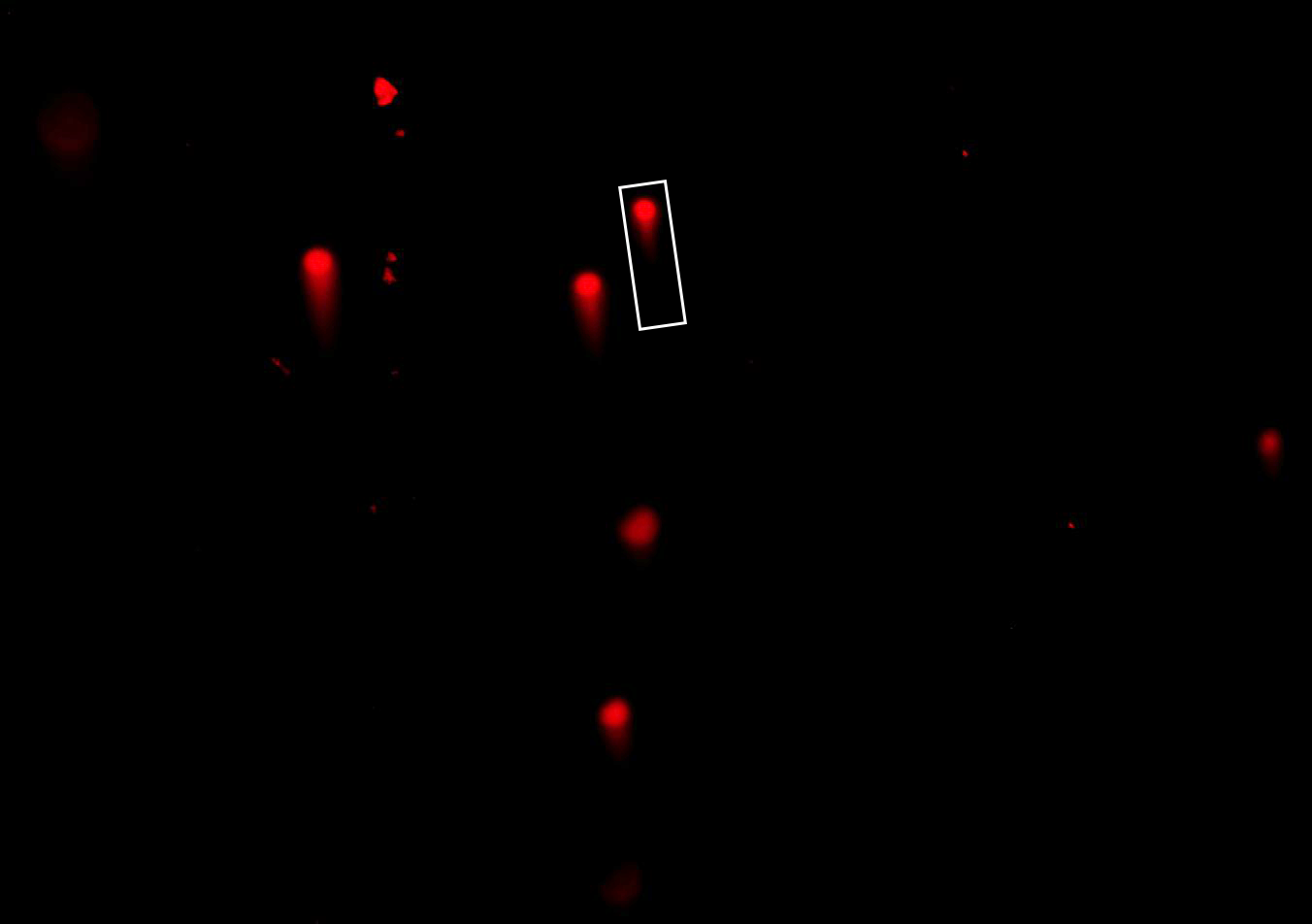

Supplement: Supplementary file 7 — Source data Fig. 5 [file 44319_2024_219_MOESM7_ESM.zip › Figure5/5A/4h/siSPIN1-1+WT.tif]

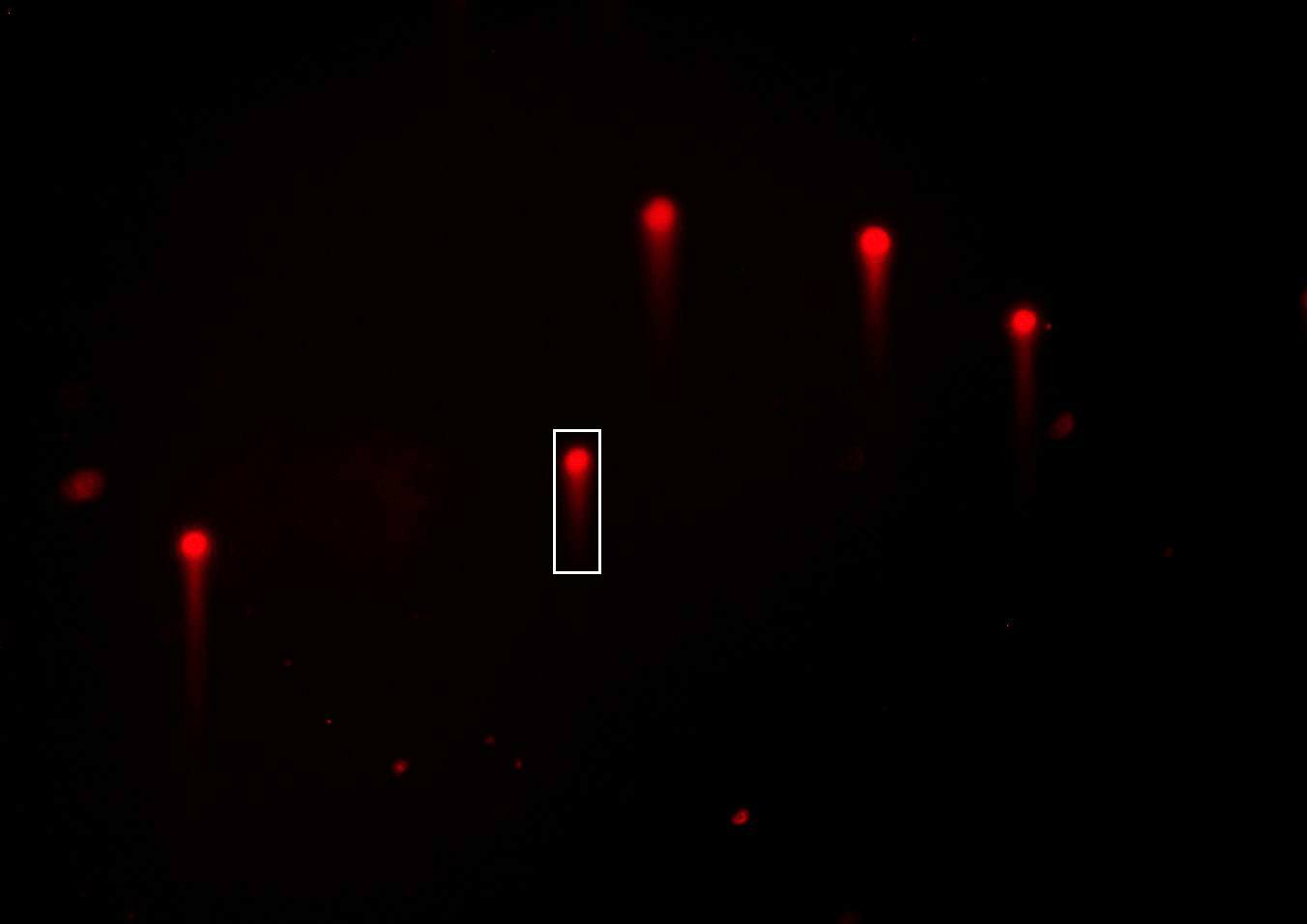

Supplement: Supplementary file 7 — Source data Fig. 5 [file 44319_2024_219_MOESM7_ESM.zip › Figure5/5A/4h/siSPIN1-1.tif]

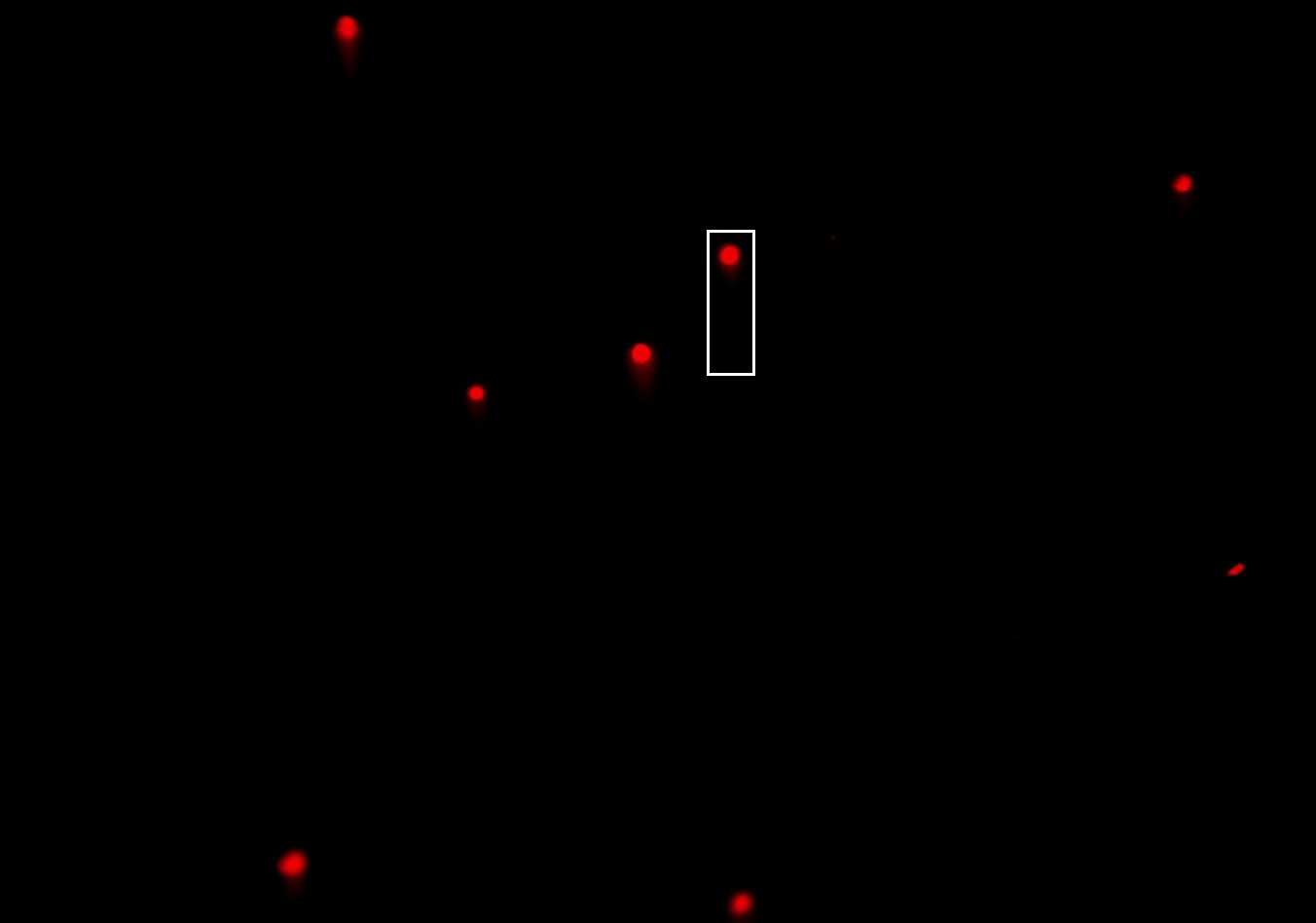

Supplement: Supplementary file 7 — Source data Fig. 5 [file 44319_2024_219_MOESM7_ESM.zip › Figure5/5A/untreated/siNC.tif]

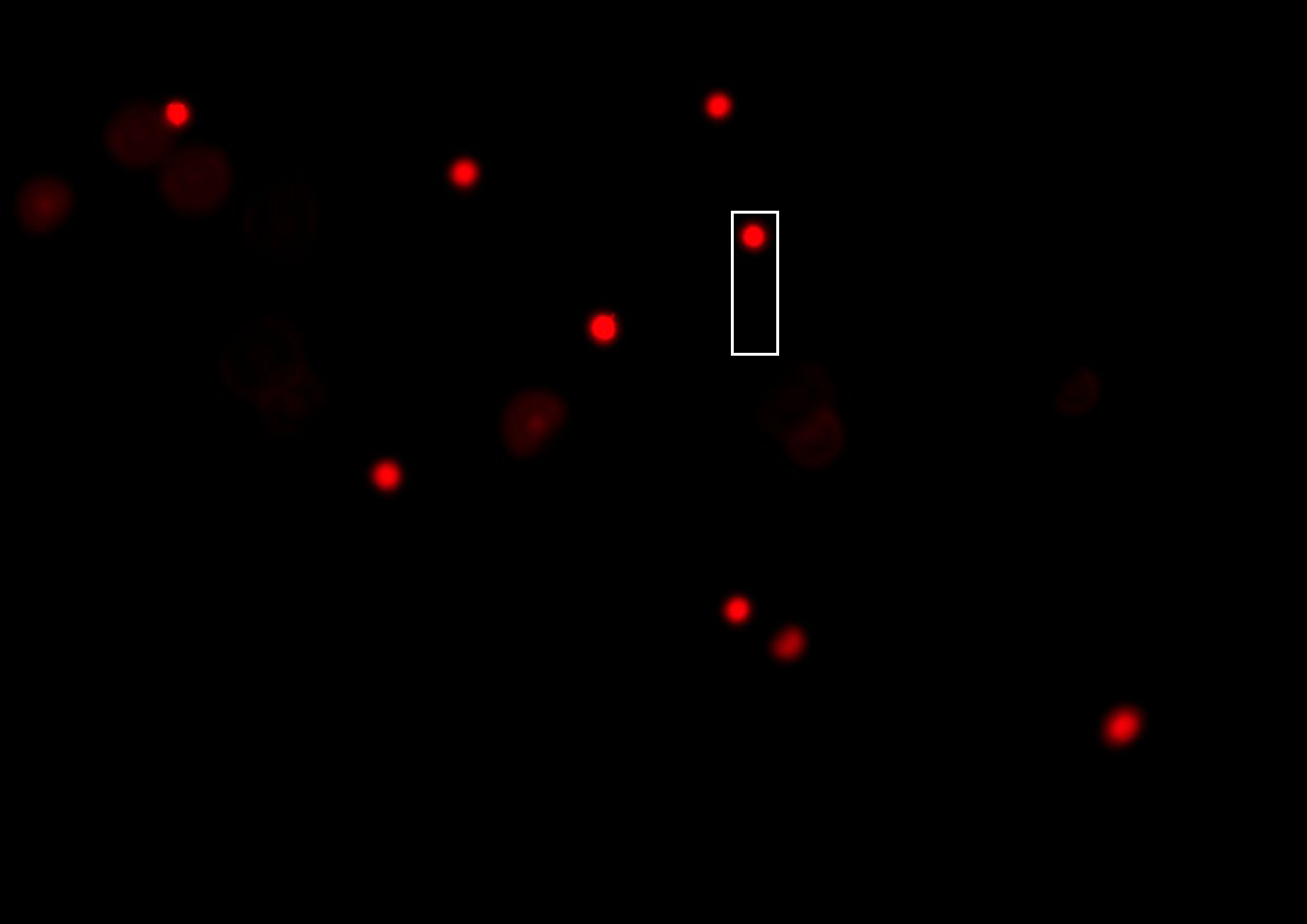

Supplement: Supplementary file 7 — Source data Fig. 5 [file 44319_2024_219_MOESM7_ESM.zip › Figure5/5A/untreated/siSPIN1-1+SPIN1-△1-50.tif]

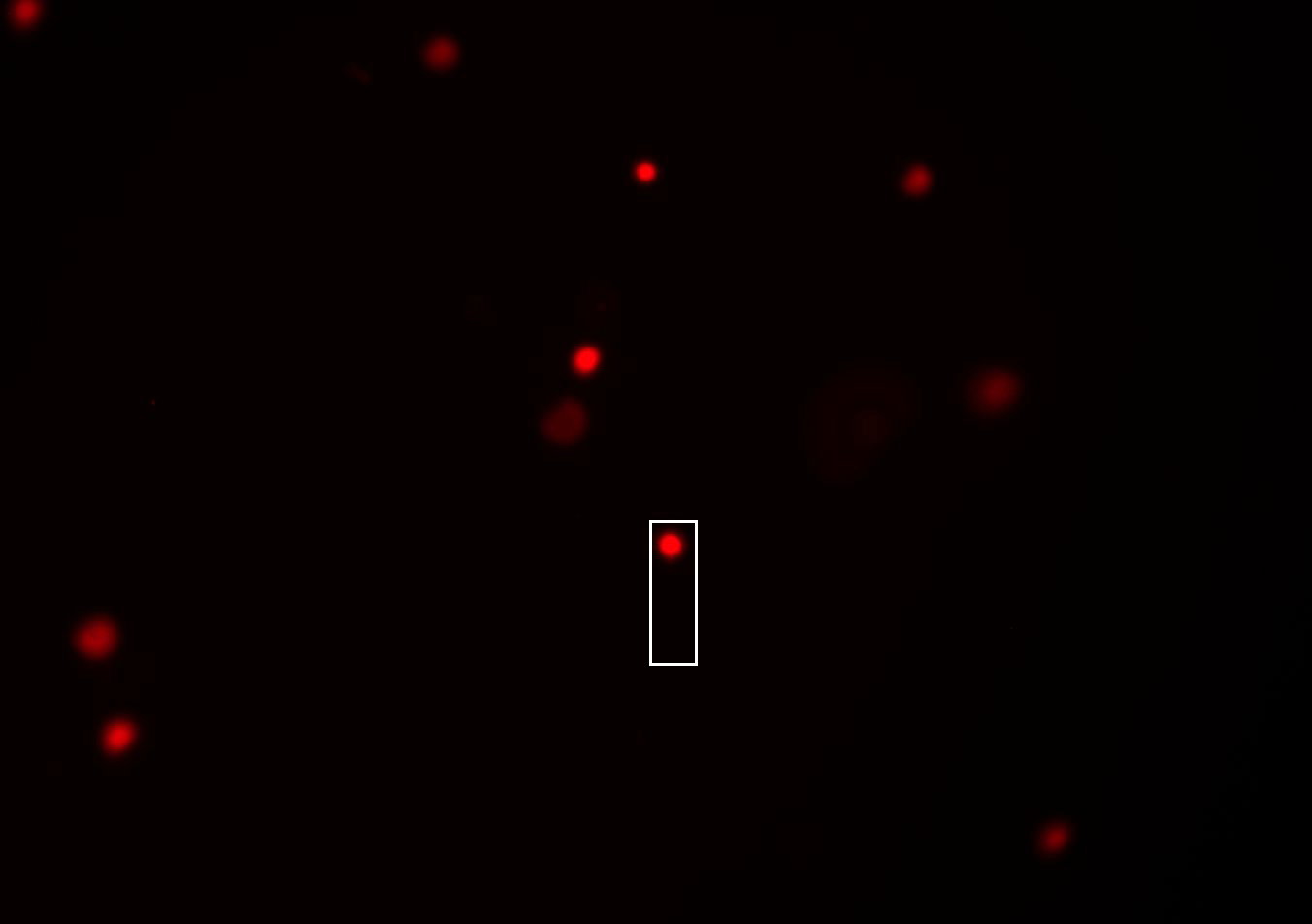

Supplement: Supplementary file 7 — Source data Fig. 5 [file 44319_2024_219_MOESM7_ESM.zip › Figure5/5A/untreated/siSPIN1-1+SPIN1-△51-125.tif]

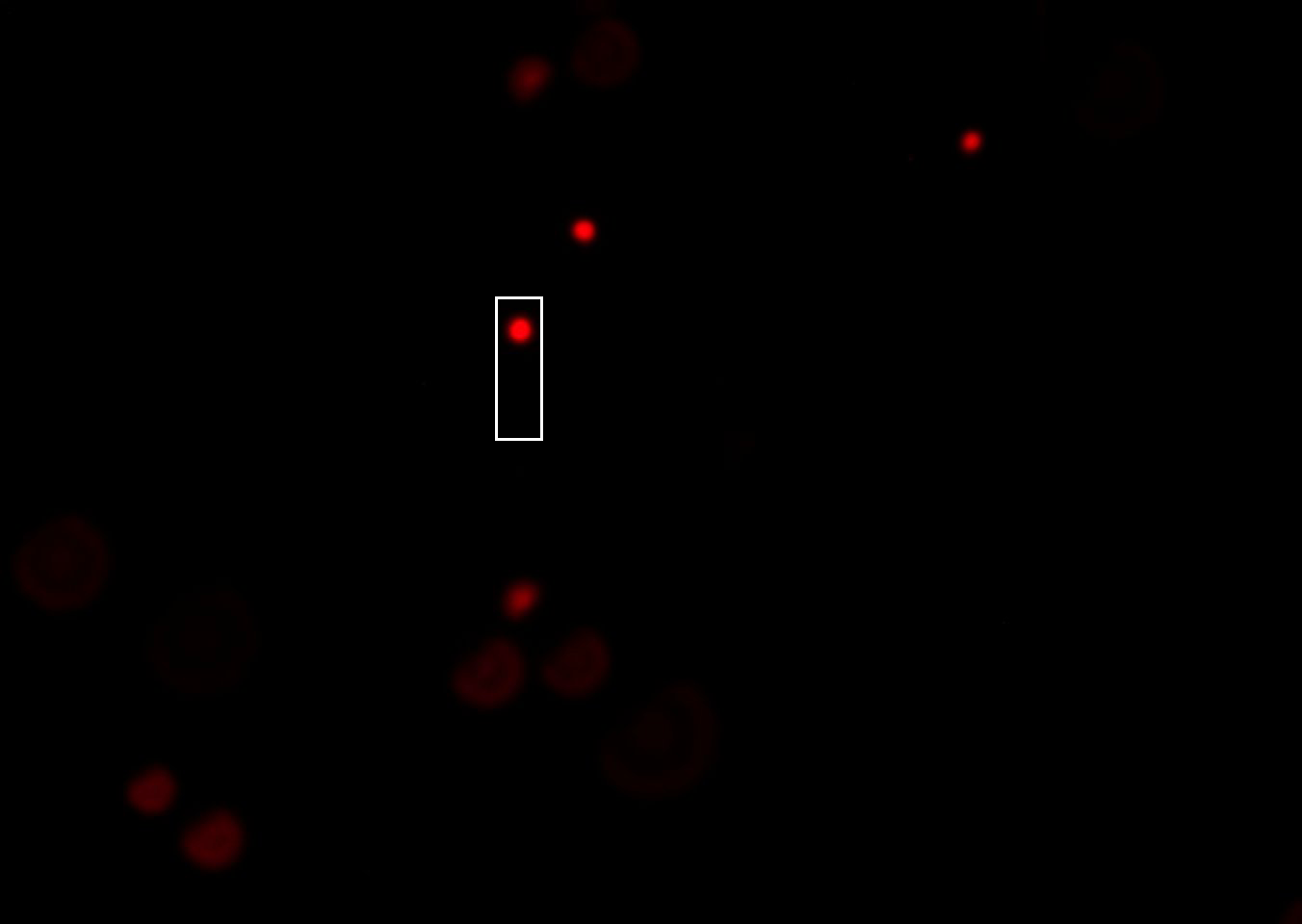

Supplement: Supplementary file 7 — Source data Fig. 5 [file 44319_2024_219_MOESM7_ESM.zip › Figure5/5A/untreated/siSPIN1-1+Vector.tif]

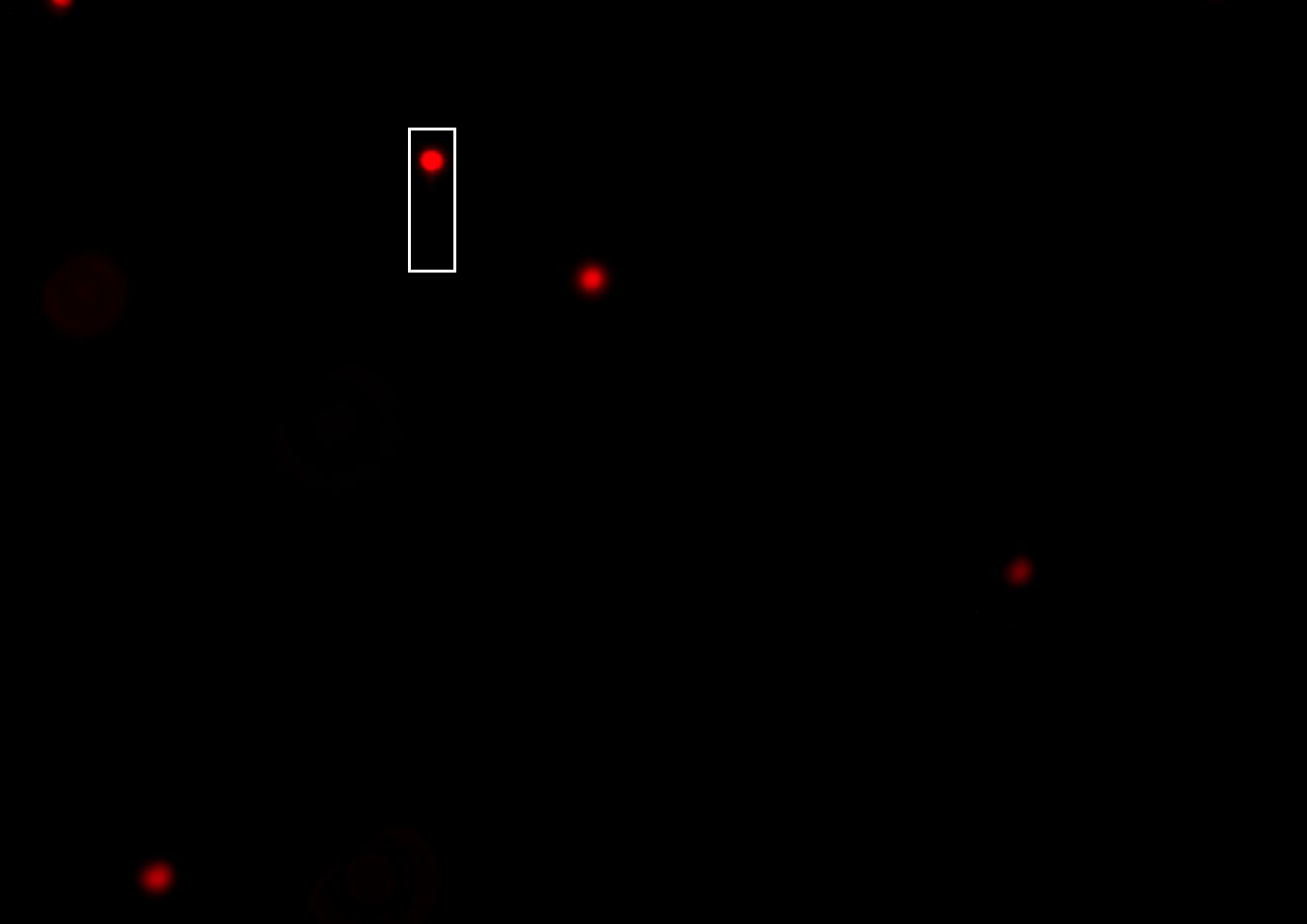

Supplement: Supplementary file 7 — Source data Fig. 5 [file 44319_2024_219_MOESM7_ESM.zip › Figure5/5A/untreated/siSPIN1-1+WT.tif]

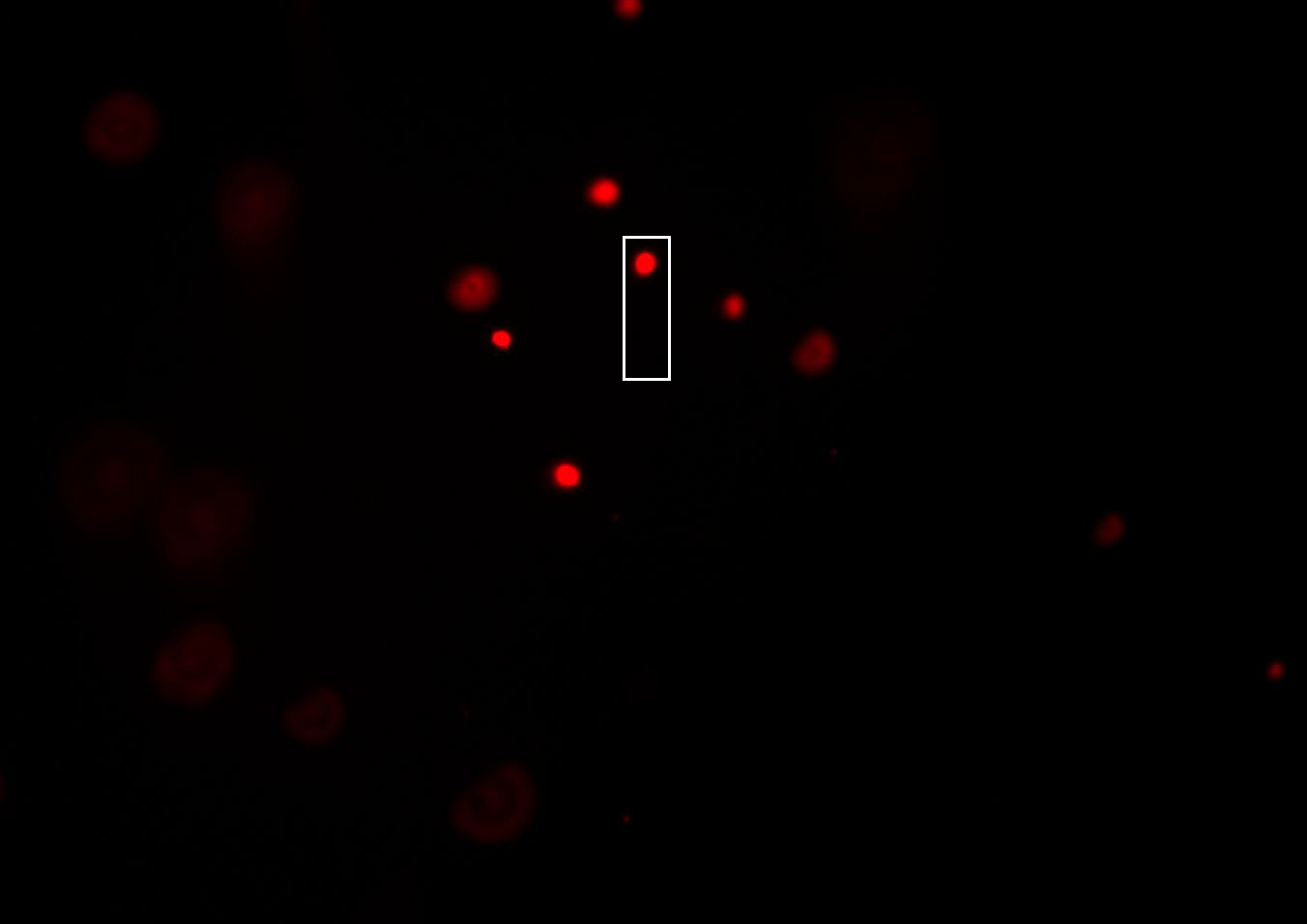

Supplement: Supplementary file 7 — Source data Fig. 5 [file 44319_2024_219_MOESM7_ESM.zip › Figure5/5A/untreated/siSPIN1-1.tif]

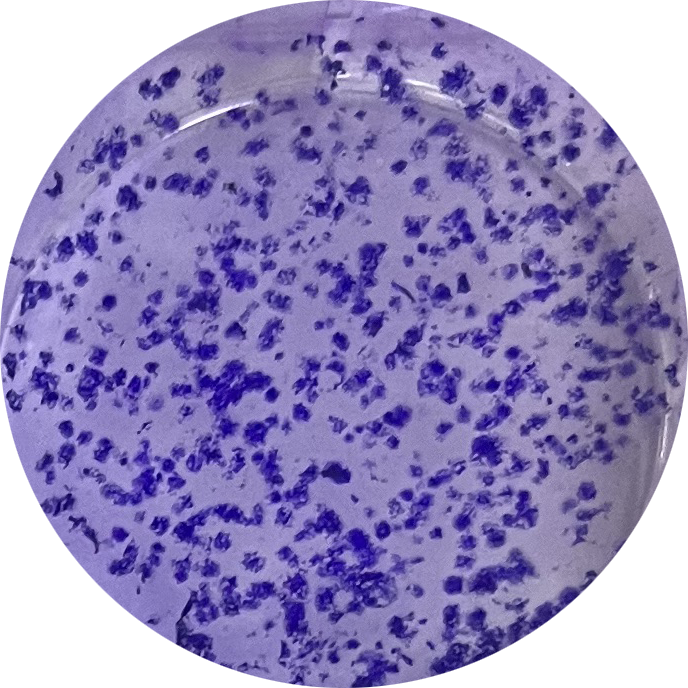

Supplement: Supplementary file 7 — Source data Fig. 5 [file 44319_2024_219_MOESM7_ESM.zip › Figure5/5B/0 Gy/SPIN1-△1-50.tif]

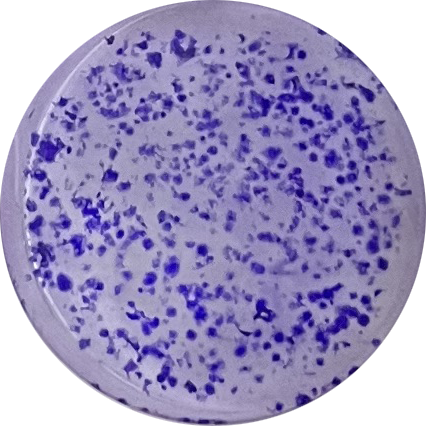

Supplement: Supplementary file 7 — Source data Fig. 5 [file 44319_2024_219_MOESM7_ESM.zip › Figure5/5B/0 Gy/SPIN1-△51-125.tif]

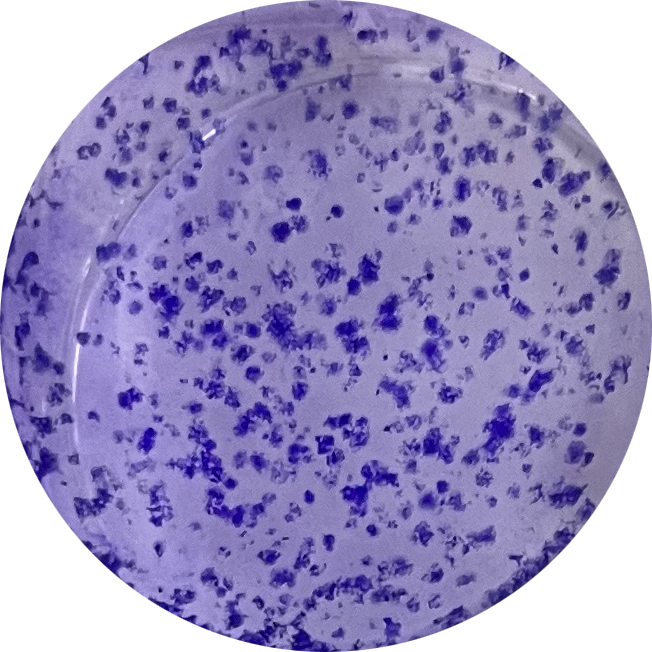

Supplement: Supplementary file 7 — Source data Fig. 5 [file 44319_2024_219_MOESM7_ESM.zip › Figure5/5B/0 Gy/Vector.tif]

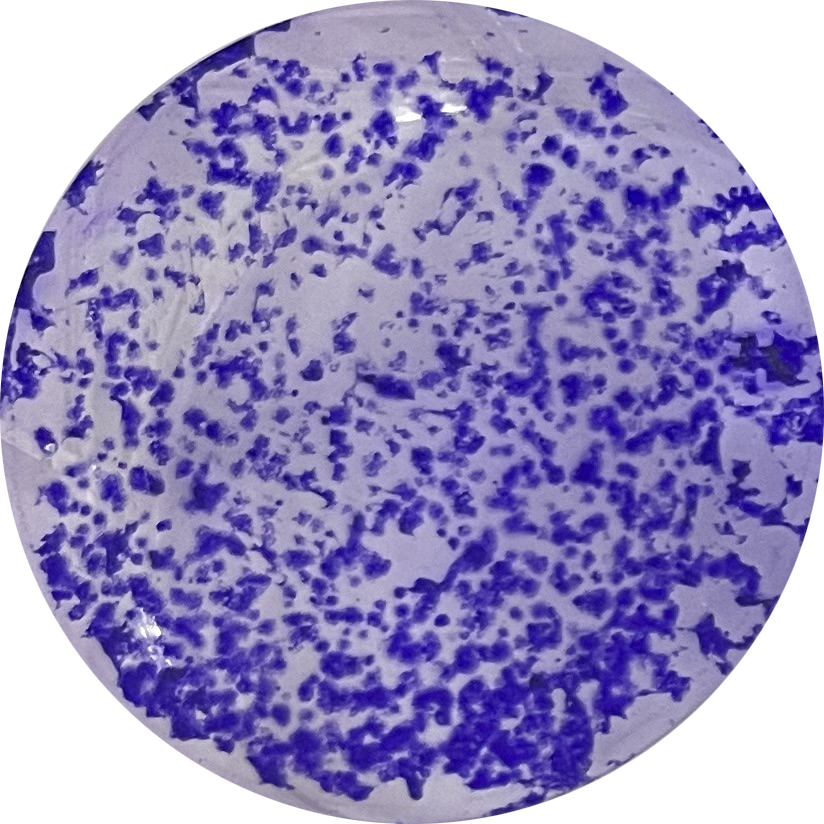

Supplement: Supplementary file 7 — Source data Fig. 5 [file 44319_2024_219_MOESM7_ESM.zip › Figure5/5B/0 Gy/WT.tif]

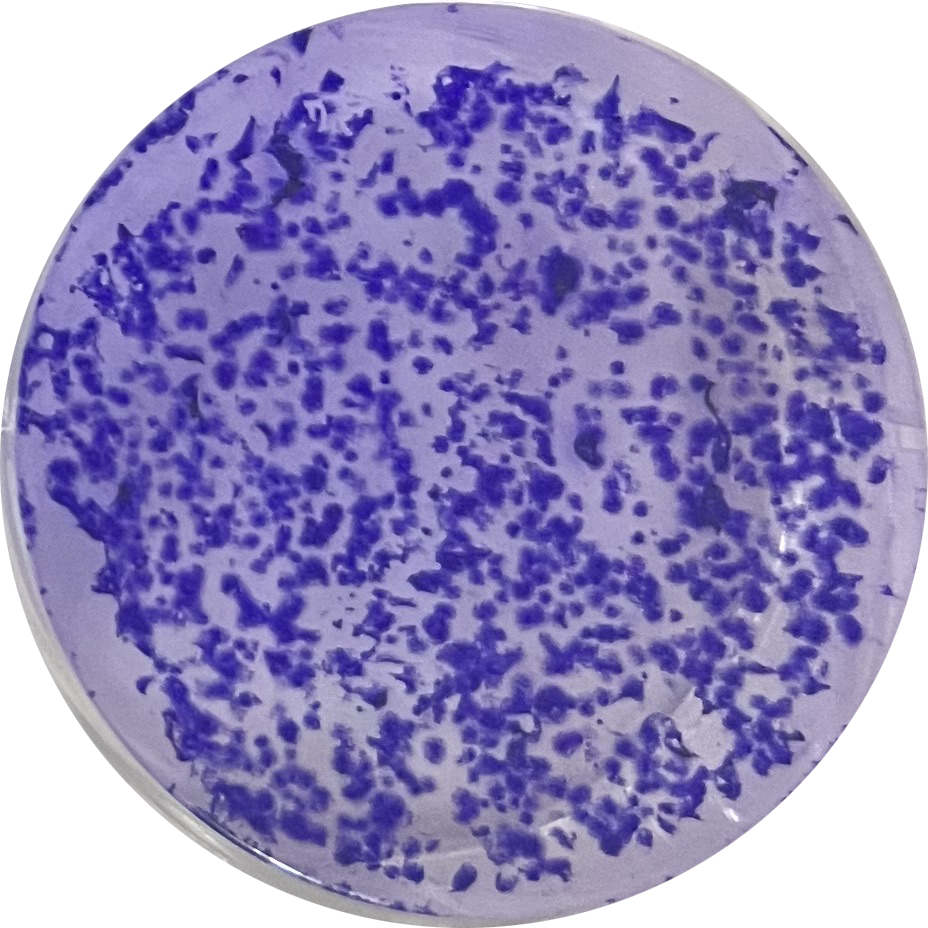

Supplement: Supplementary file 7 — Source data Fig. 5 [file 44319_2024_219_MOESM7_ESM.zip › Figure5/5B/0 Gy/siNC.tif]

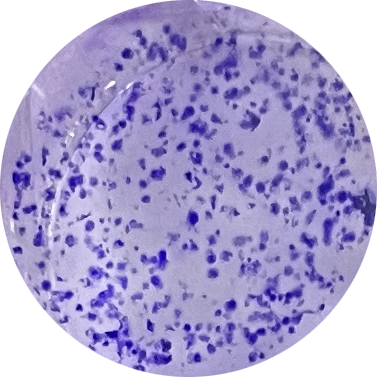

Supplement: Supplementary file 7 — Source data Fig. 5 [file 44319_2024_219_MOESM7_ESM.zip › Figure5/5B/0 Gy/siSPIN1-1.tif]

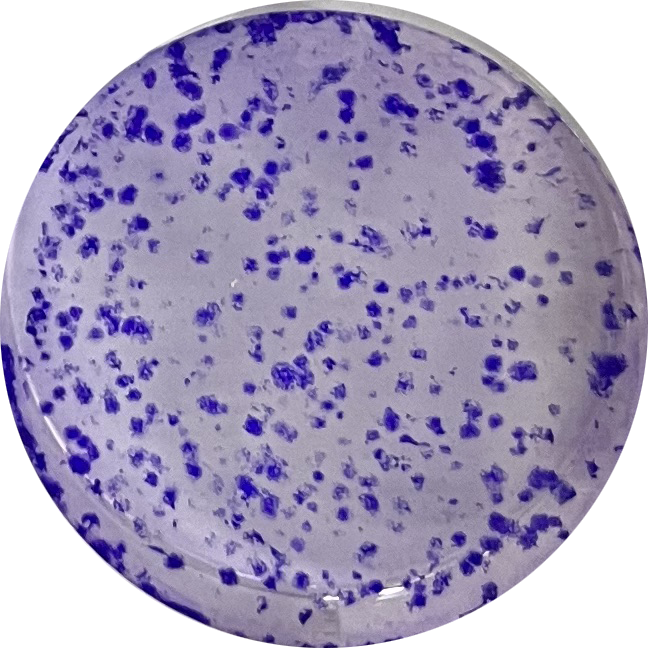

Supplement: Supplementary file 7 — Source data Fig. 5 [file 44319_2024_219_MOESM7_ESM.zip › Figure5/5B/1 Gy/SPIN1-△1-50.tif]

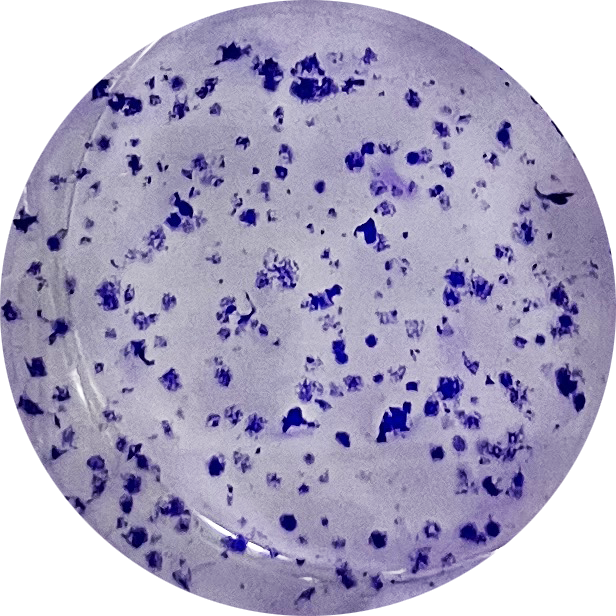

Supplement: Supplementary file 7 — Source data Fig. 5 [file 44319_2024_219_MOESM7_ESM.zip › Figure5/5B/1 Gy/SPIN1-△51-125.tif]

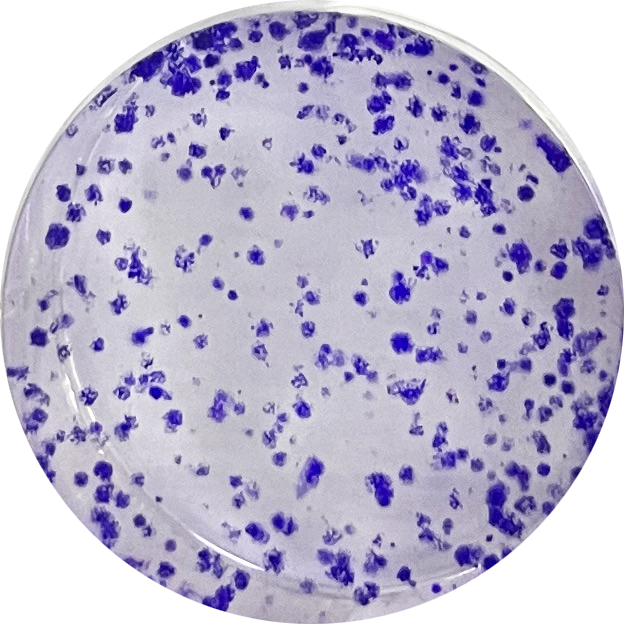

Supplement: Supplementary file 7 — Source data Fig. 5 [file 44319_2024_219_MOESM7_ESM.zip › Figure5/5B/1 Gy/Vector.tif]

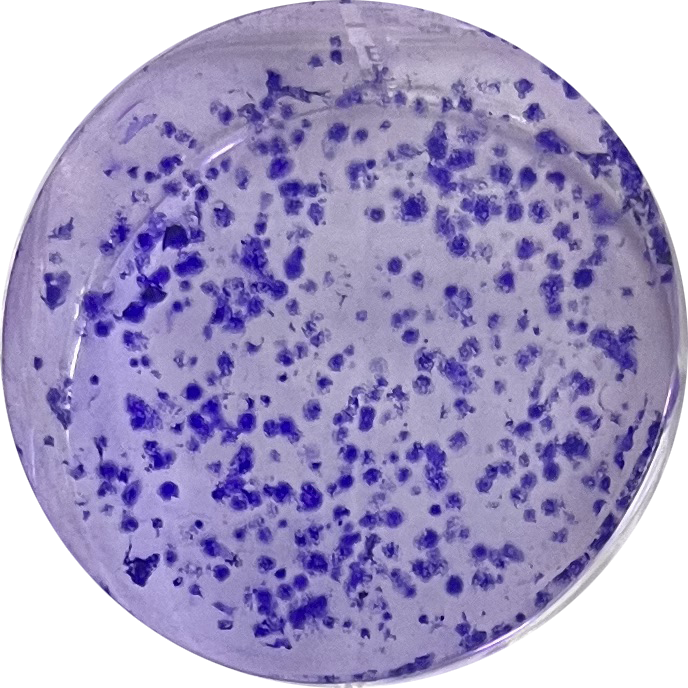

Supplement: Supplementary file 7 — Source data Fig. 5 [file 44319_2024_219_MOESM7_ESM.zip › Figure5/5B/1 Gy/WT.tif]

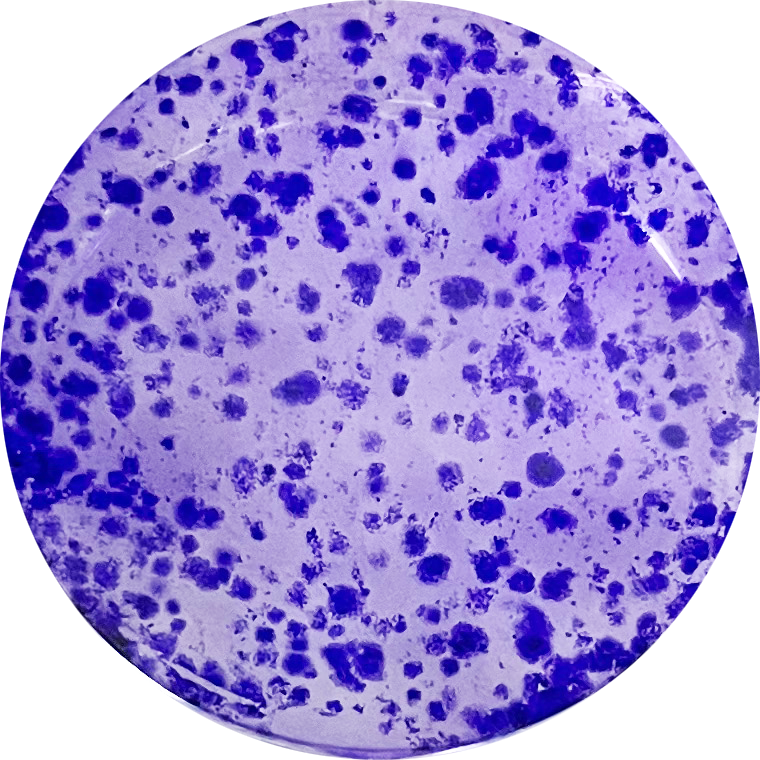

Supplement: Supplementary file 7 — Source data Fig. 5 [file 44319_2024_219_MOESM7_ESM.zip › Figure5/5B/1 Gy/siNC.tif]

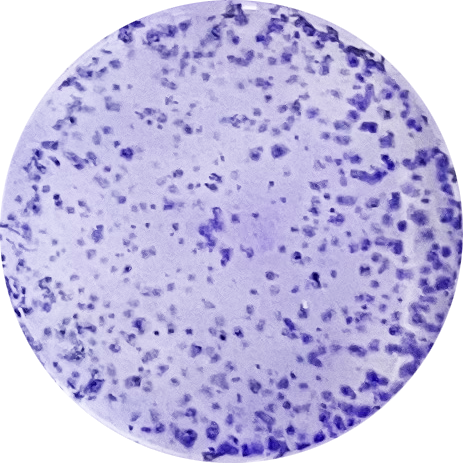

Supplement: Supplementary file 7 — Source data Fig. 5 [file 44319_2024_219_MOESM7_ESM.zip › Figure5/5B/1 Gy/siSPIN1-1.tif]

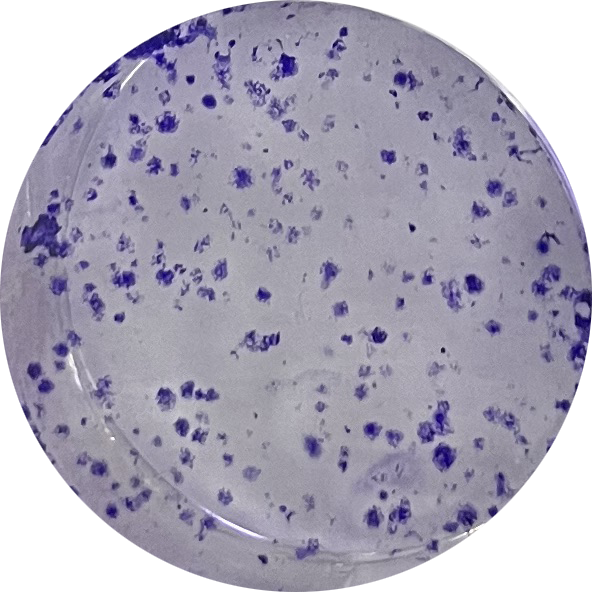

Supplement: Supplementary file 7 — Source data Fig. 5 [file 44319_2024_219_MOESM7_ESM.zip › Figure5/5B/2 Gy/SPIN1-△1-50.tif]

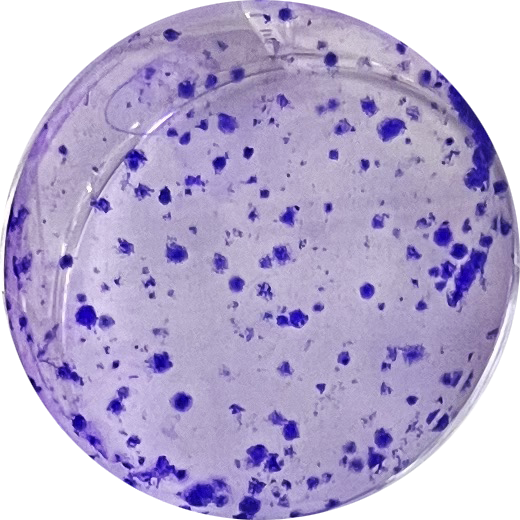

Supplement: Supplementary file 7 — Source data Fig. 5 [file 44319_2024_219_MOESM7_ESM.zip › Figure5/5B/2 Gy/SPIN1-△51-125.tif]

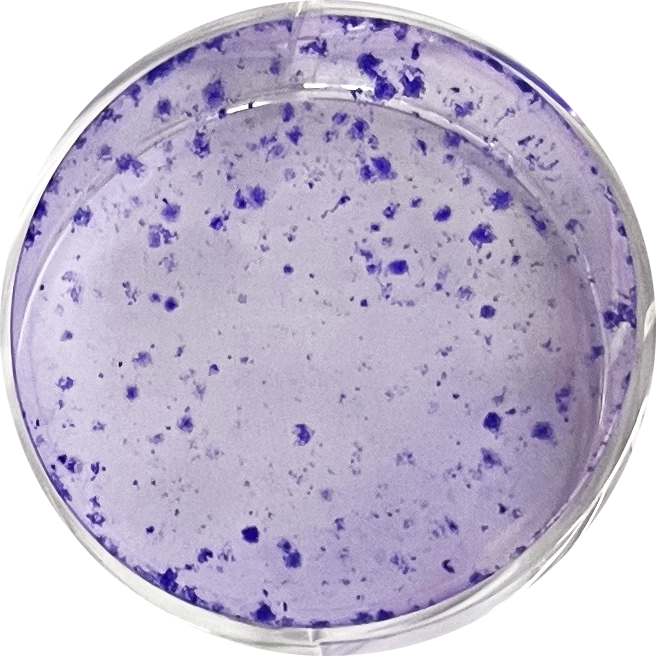

Supplement: Supplementary file 7 — Source data Fig. 5 [file 44319_2024_219_MOESM7_ESM.zip › Figure5/5B/2 Gy/Vector.tif]

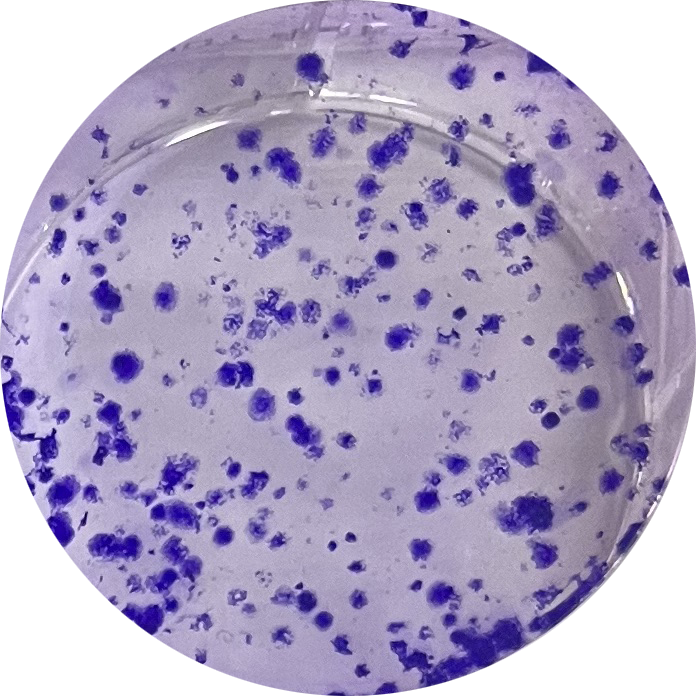

Supplement: Supplementary file 7 — Source data Fig. 5 [file 44319_2024_219_MOESM7_ESM.zip › Figure5/5B/2 Gy/WT.tif]

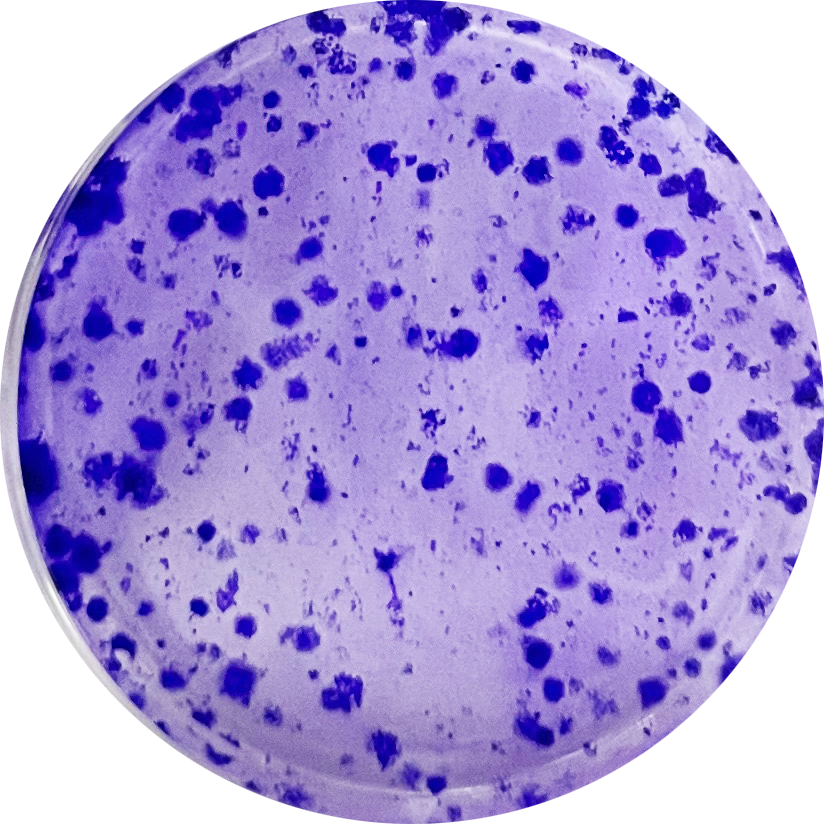

Supplement: Supplementary file 7 — Source data Fig. 5 [file 44319_2024_219_MOESM7_ESM.zip › Figure5/5B/2 Gy/siNC.tif]

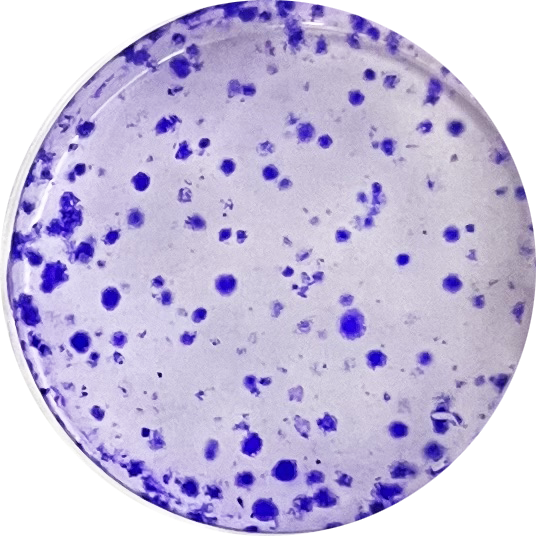

Supplement: Supplementary file 7 — Source data Fig. 5 [file 44319_2024_219_MOESM7_ESM.zip › Figure5/5B/2 Gy/siSPIN1-1.tif]

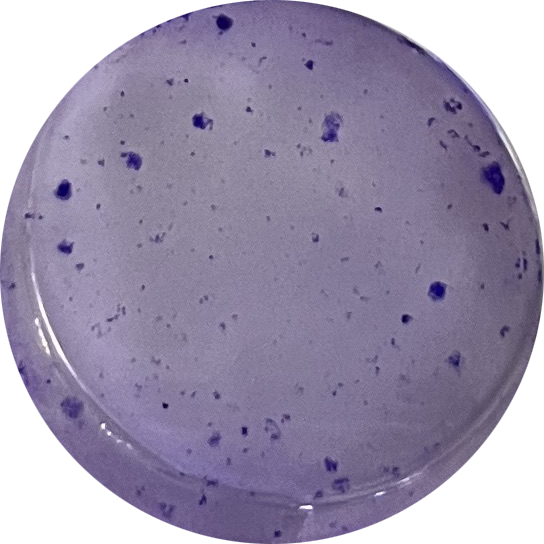

Supplement: Supplementary file 7 — Source data Fig. 5 [file 44319_2024_219_MOESM7_ESM.zip › Figure5/5B/4 Gy/SPIN1-△1-50.tif]

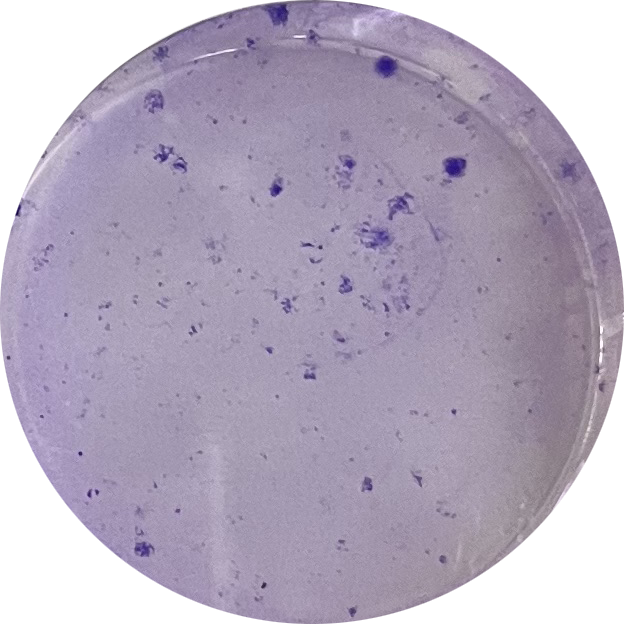

Supplement: Supplementary file 7 — Source data Fig. 5 [file 44319_2024_219_MOESM7_ESM.zip › Figure5/5B/4 Gy/SPIN1-△51-125.tif]

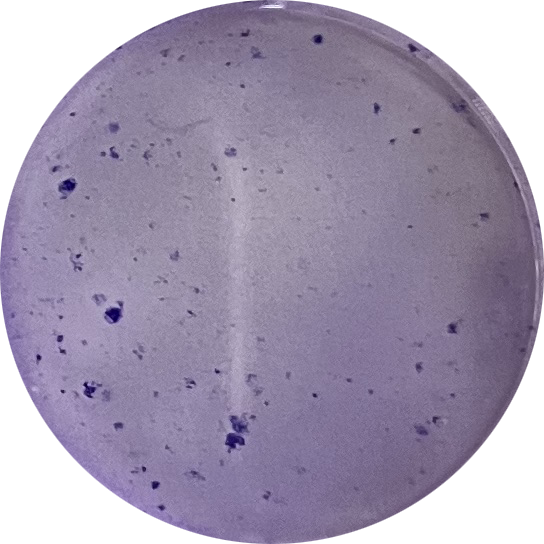

Supplement: Supplementary file 7 — Source data Fig. 5 [file 44319_2024_219_MOESM7_ESM.zip › Figure5/5B/4 Gy/Vector.tif]

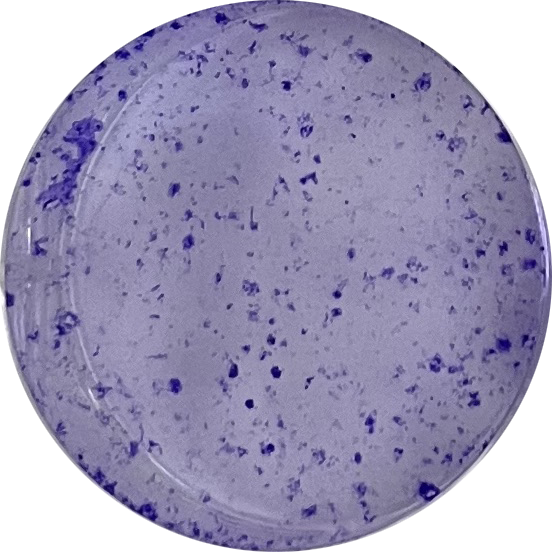

Supplement: Supplementary file 7 — Source data Fig. 5 [file 44319_2024_219_MOESM7_ESM.zip › Figure5/5B/4 Gy/WT.tif]

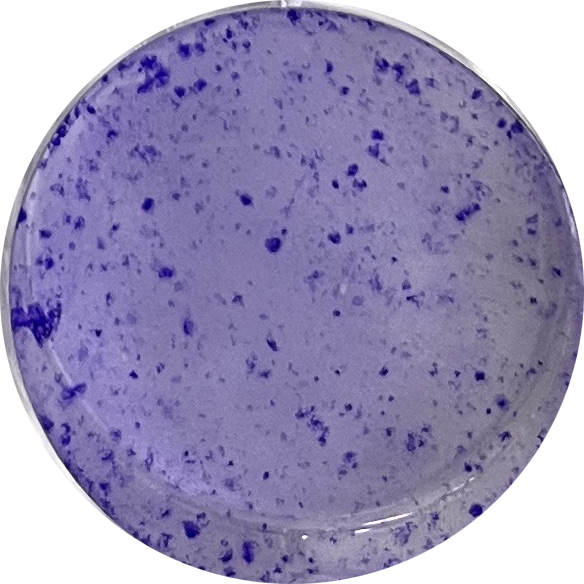

Supplement: Supplementary file 7 — Source data Fig. 5 [file 44319_2024_219_MOESM7_ESM.zip › Figure5/5B/4 Gy/siNC.tif]

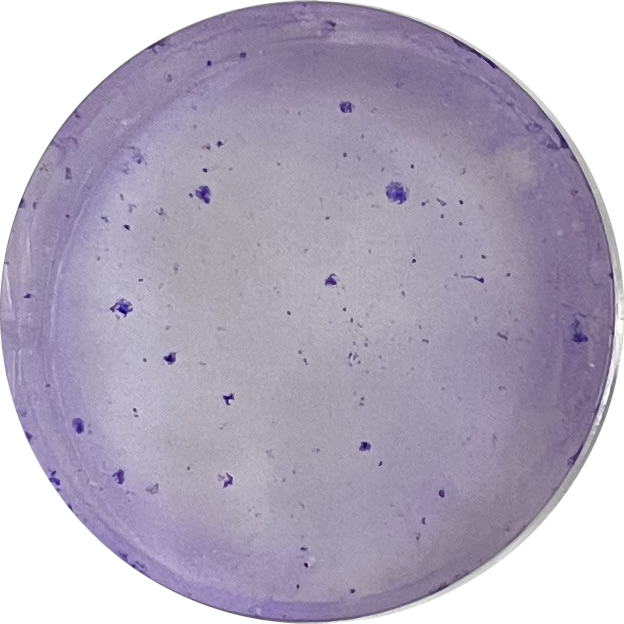

Supplement: Supplementary file 7 — Source data Fig. 5 [file 44319_2024_219_MOESM7_ESM.zip › Figure5/5B/4 Gy/siSPIN1-1.tif]

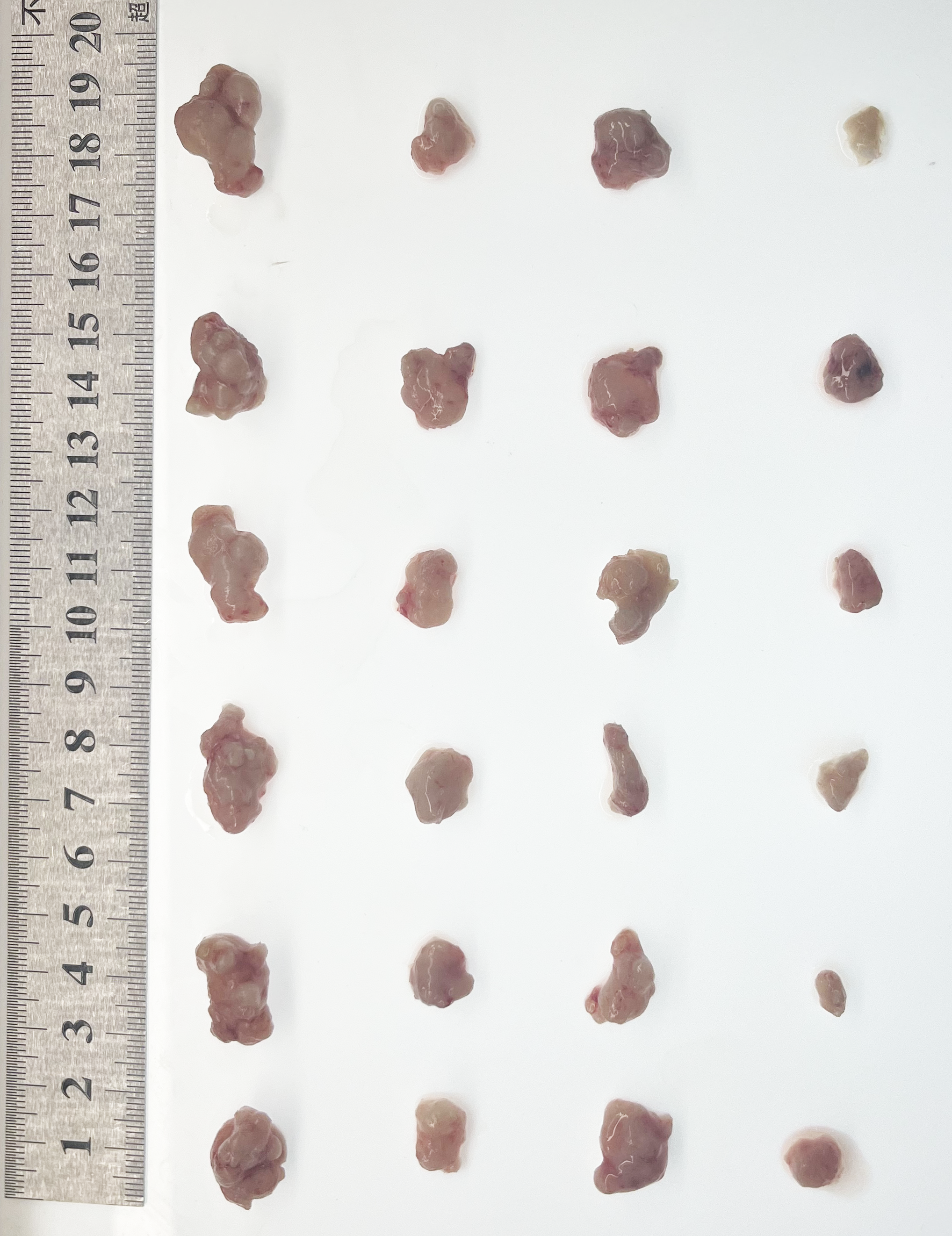

Supplement: Supplementary file 7 — Source data Fig. 5 [file 44319_2024_219_MOESM7_ESM.zip › Figure5/5E/Cisplatin Tumor.tif]

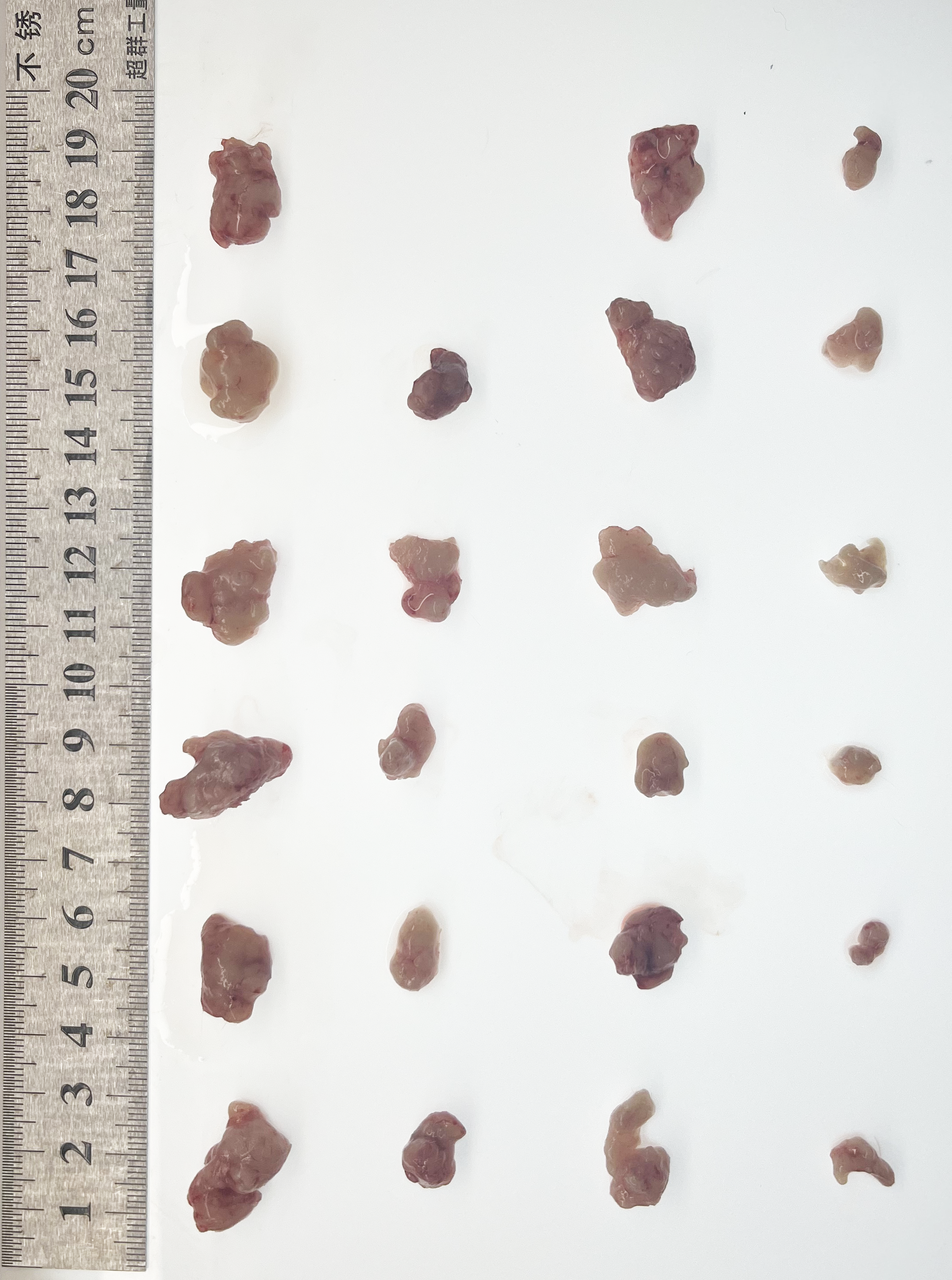

Supplement: Supplementary file 7 — Source data Fig. 5 [file 44319_2024_219_MOESM7_ESM.zip › Figure5/5H/Olaparib Tumor.tif]

Figure EV1

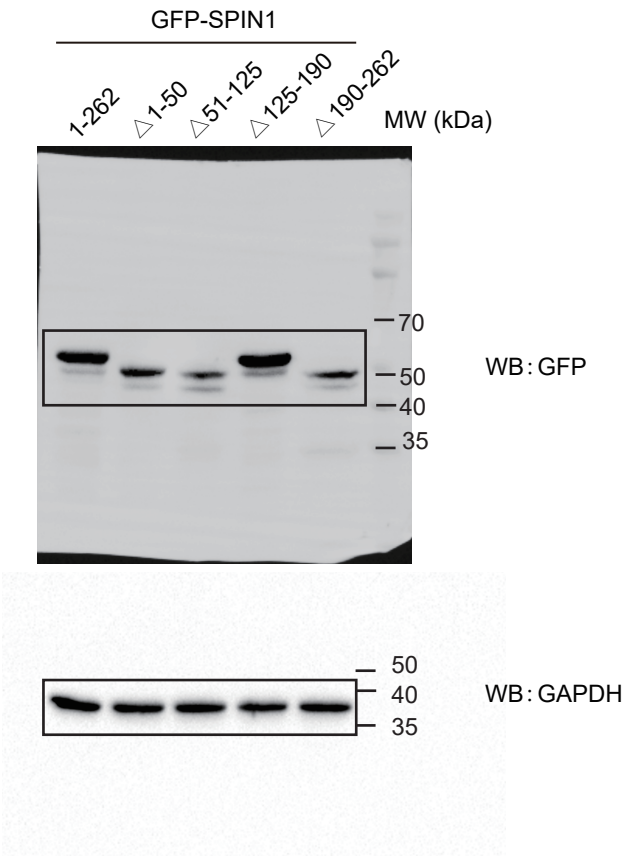

Supplement: Supplementary file 8 — EV Figures Source Data [file 44319_2024_219_MOESM8_ESM.zip › source data for Expanded View figures/Figure EV1/EV1.pdf]

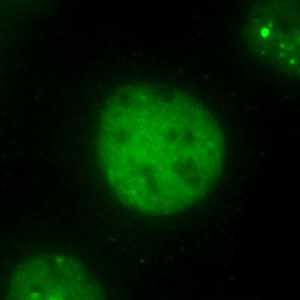

Supplement: Supplementary file 8 — EV Figures Source Data [file 44319_2024_219_MOESM8_ESM.zip › source data for Expanded View figures/Figure EV2/EV2B/siNC/53BP1/53BP1.jpg]

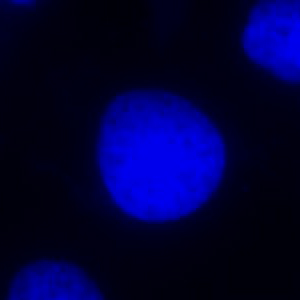

Supplement: Supplementary file 8 — EV Figures Source Data [file 44319_2024_219_MOESM8_ESM.zip › source data for Expanded View figures/Figure EV2/EV2B/siNC/53BP1/DAPI.jpg]

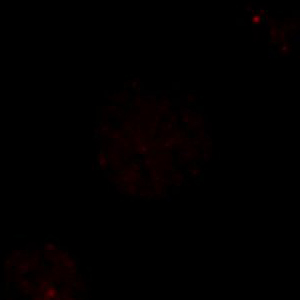

Supplement: Supplementary file 8 — EV Figures Source Data [file 44319_2024_219_MOESM8_ESM.zip › source data for Expanded View figures/Figure EV2/EV2B/siNC/53BP1/γH2AX.jpg]

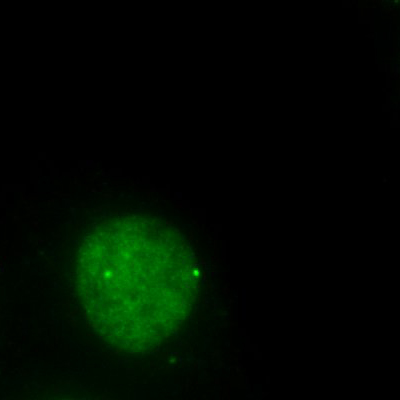

Supplement: Supplementary file 8 — EV Figures Source Data [file 44319_2024_219_MOESM8_ESM.zip › source data for Expanded View figures/Figure EV2/EV2B/siNC/BRCA1/BRCA1.jpg]

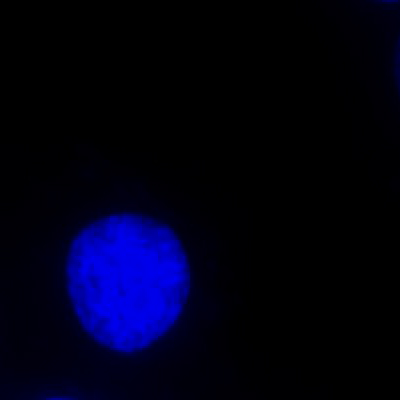

Supplement: Supplementary file 8 — EV Figures Source Data [file 44319_2024_219_MOESM8_ESM.zip › source data for Expanded View figures/Figure EV2/EV2B/siNC/BRCA1/DAPI.jpg]

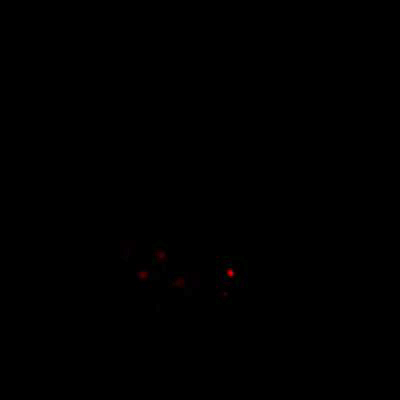

Supplement: Supplementary file 8 — EV Figures Source Data [file 44319_2024_219_MOESM8_ESM.zip › source data for Expanded View figures/Figure EV2/EV2B/siNC/BRCA1/γH2AX.jpg]

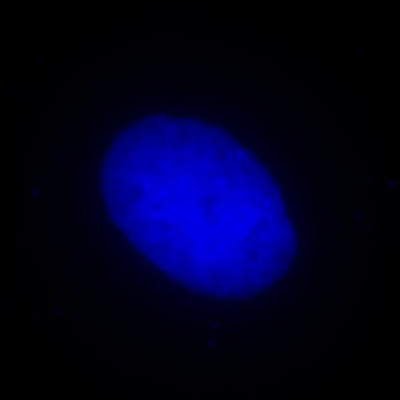

Supplement: Supplementary file 8 — EV Figures Source Data [file 44319_2024_219_MOESM8_ESM.zip › source data for Expanded View figures/Figure EV2/EV2B/siNC/RAD51/DAPI.jpg]

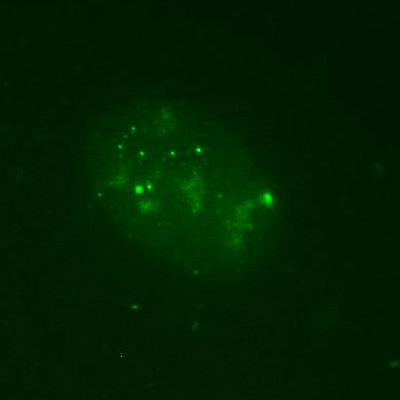

Supplement: Supplementary file 8 — EV Figures Source Data [file 44319_2024_219_MOESM8_ESM.zip › source data for Expanded View figures/Figure EV2/EV2B/siNC/RAD51/RAD51.jpg]

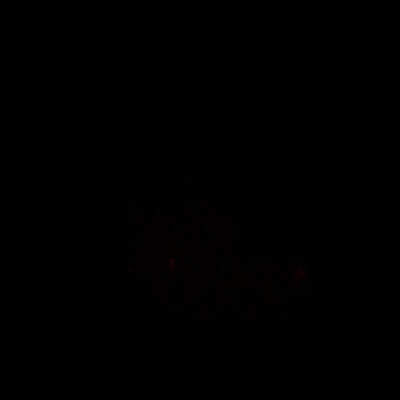

Supplement: Supplementary file 8 — EV Figures Source Data [file 44319_2024_219_MOESM8_ESM.zip › source data for Expanded View figures/Figure EV2/EV2B/siNC/RAD51/γH2AX.jpg]

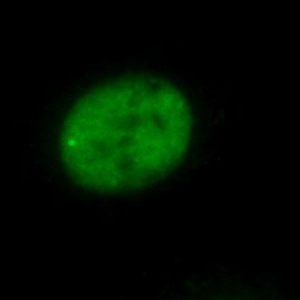

Supplement: Supplementary file 8 — EV Figures Source Data [file 44319_2024_219_MOESM8_ESM.zip › source data for Expanded View figures/Figure EV2/EV2B/siSPIN1/53BP1/53BP1.jpg]

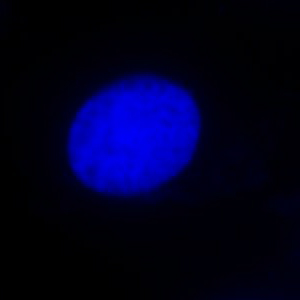

Supplement: Supplementary file 8 — EV Figures Source Data [file 44319_2024_219_MOESM8_ESM.zip › source data for Expanded View figures/Figure EV2/EV2B/siSPIN1/53BP1/DAPI.jpg]

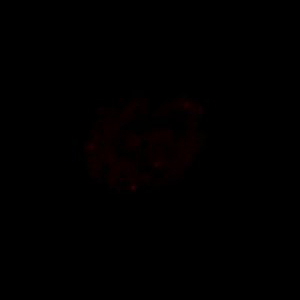

Supplement: Supplementary file 8 — EV Figures Source Data [file 44319_2024_219_MOESM8_ESM.zip › source data for Expanded View figures/Figure EV2/EV2B/siSPIN1/53BP1/γH2AX.jpg]

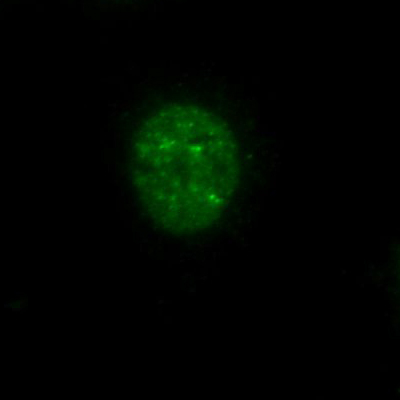

Supplement: Supplementary file 8 — EV Figures Source Data [file 44319_2024_219_MOESM8_ESM.zip › source data for Expanded View figures/Figure EV2/EV2B/siSPIN1/BRCA1/BRCA1.jpg]

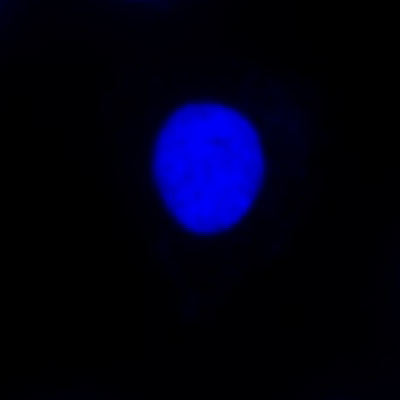

Supplement: Supplementary file 8 — EV Figures Source Data [file 44319_2024_219_MOESM8_ESM.zip › source data for Expanded View figures/Figure EV2/EV2B/siSPIN1/BRCA1/DAPI.jpg]

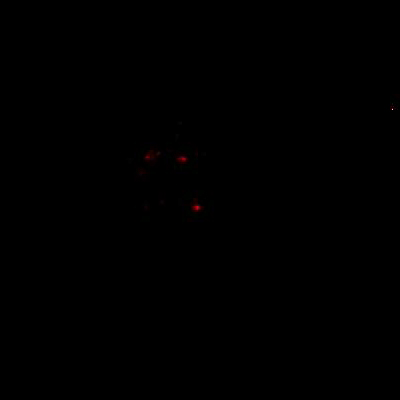

Supplement: Supplementary file 8 — EV Figures Source Data [file 44319_2024_219_MOESM8_ESM.zip › source data for Expanded View figures/Figure EV2/EV2B/siSPIN1/BRCA1/γH2AX.jpg]

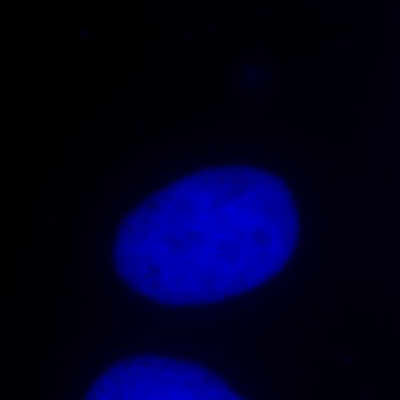

Supplement: Supplementary file 8 — EV Figures Source Data [file 44319_2024_219_MOESM8_ESM.zip › source data for Expanded View figures/Figure EV2/EV2B/siSPIN1/RAD51/DAPI.jpg]

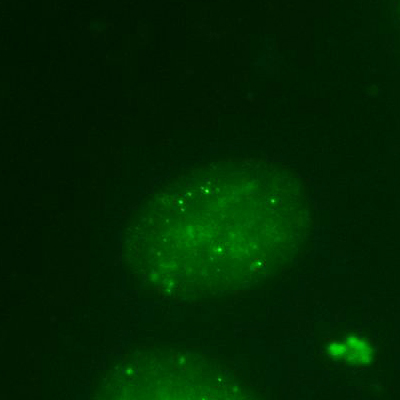

Supplement: Supplementary file 8 — EV Figures Source Data [file 44319_2024_219_MOESM8_ESM.zip › source data for Expanded View figures/Figure EV2/EV2B/siSPIN1/RAD51/RAD51.jpg]

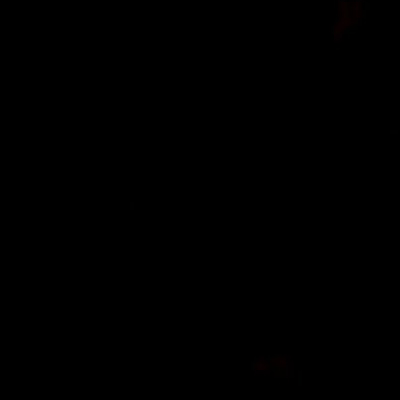

Supplement: Supplementary file 8 — EV Figures Source Data [file 44319_2024_219_MOESM8_ESM.zip › source data for Expanded View figures/Figure EV2/EV2B/siSPIN1/RAD51/γH2AX.jpg]

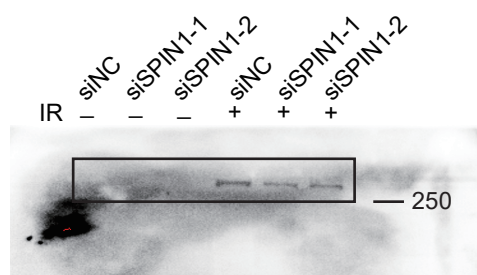

WB:P-ATM

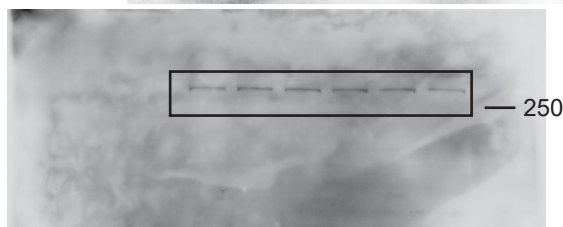

WB:ATM

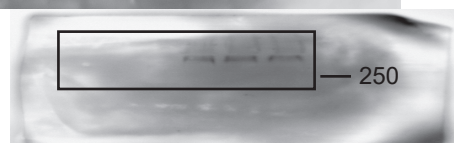

WB:P-ATR

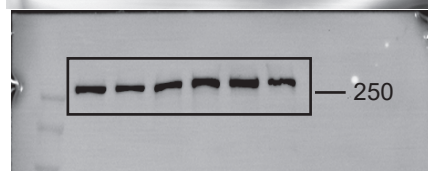

WB:ATR

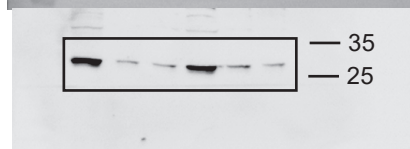

WB:SPIN1

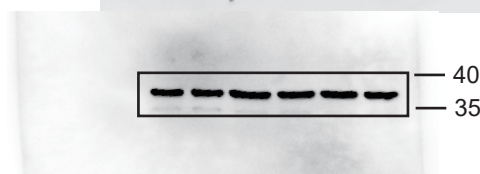

WB:GAPDH

Supplement: Supplementary file 8 — EV Figures Source Data [file 44319_2024_219_MOESM8_ESM.zip › source data for Expanded View figures/Figure EV2/EV2C/EV2C.pdf]

Figure EV3A

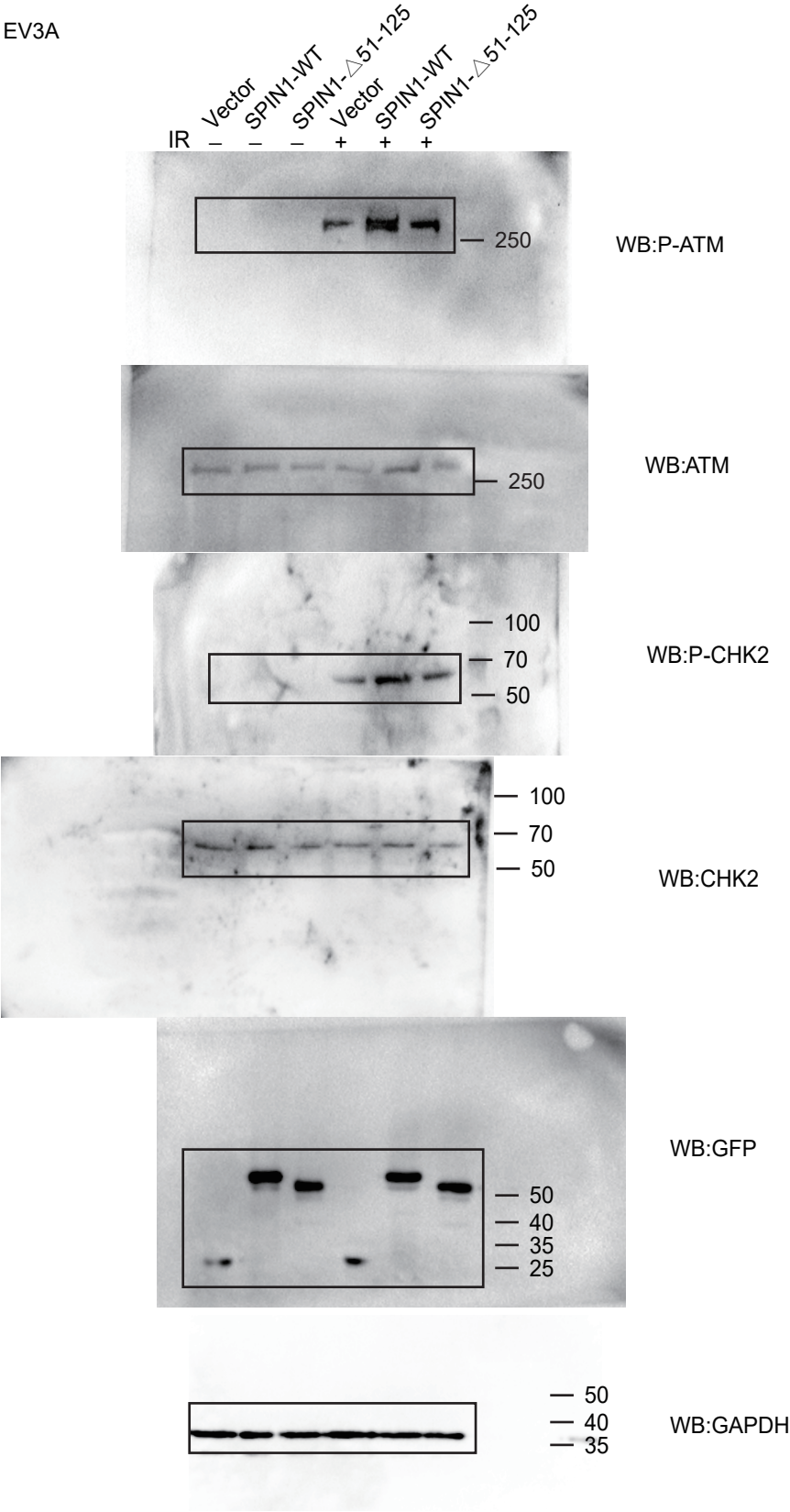

Supplement: Supplementary file 8 — EV Figures Source Data [file 44319_2024_219_MOESM8_ESM.zip › source data for Expanded View figures/Figure EV3/EV3A/EV3A.pdf]

Figure EV4A

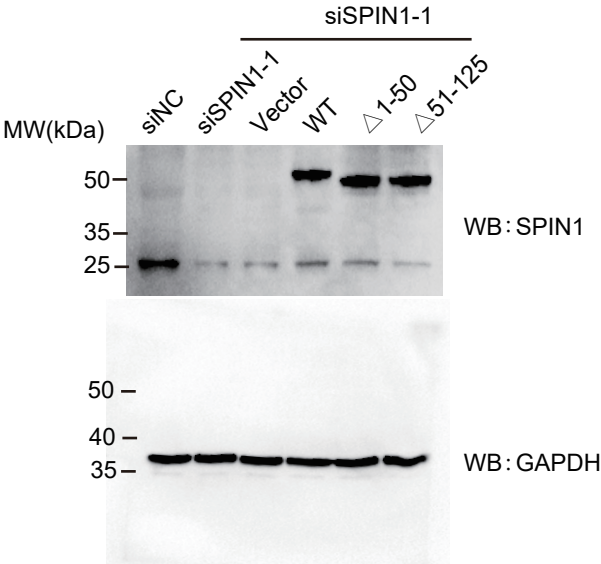

Supplement: Supplementary file 8 — EV Figures Source Data [file 44319_2024_219_MOESM8_ESM.zip › source data for Expanded View figures/Figure EV4/EV4A/EV4A.pdf]

Figure EV4C

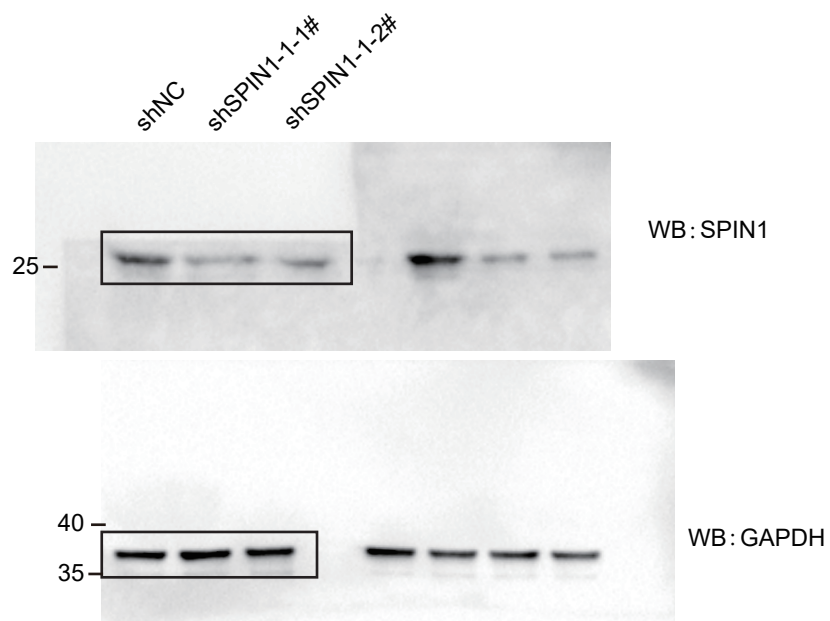

Supplement: Supplementary file 8 — EV Figures Source Data [file 44319_2024_219_MOESM8_ESM.zip › source data for Expanded View figures/Figure EV4/EV4C/EV4C.pdf]
