# Supplementary material for: SPIN1 facilitates chemoresistance and HR repair by promoting Tip60 binding to H3K9me3
Source: EMBO Rep. 2024 Aug 1;25(9):15. doi: 10.1038/s44319-024-00219-1 (PMC11387427; doi:10.1038/s44319-024-00219-1)
Supplement: Supplementary file 9 — Expanded View Figures [file 44319_2024_219_MOESM9_ESM.pdf]

Expanded View Figures

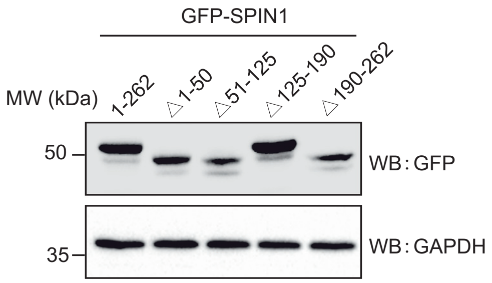

Figure EV1. Protein expressions of GFP-tagged SPIN1 mutants were examined by Western blot analysis.

The indicated mutants of GFP-tagged SPIN1 were transfected to U2OS cells, and Western blot was performed to detect the protein expression levels. Source data are available online for this figure.

© The Author(s)

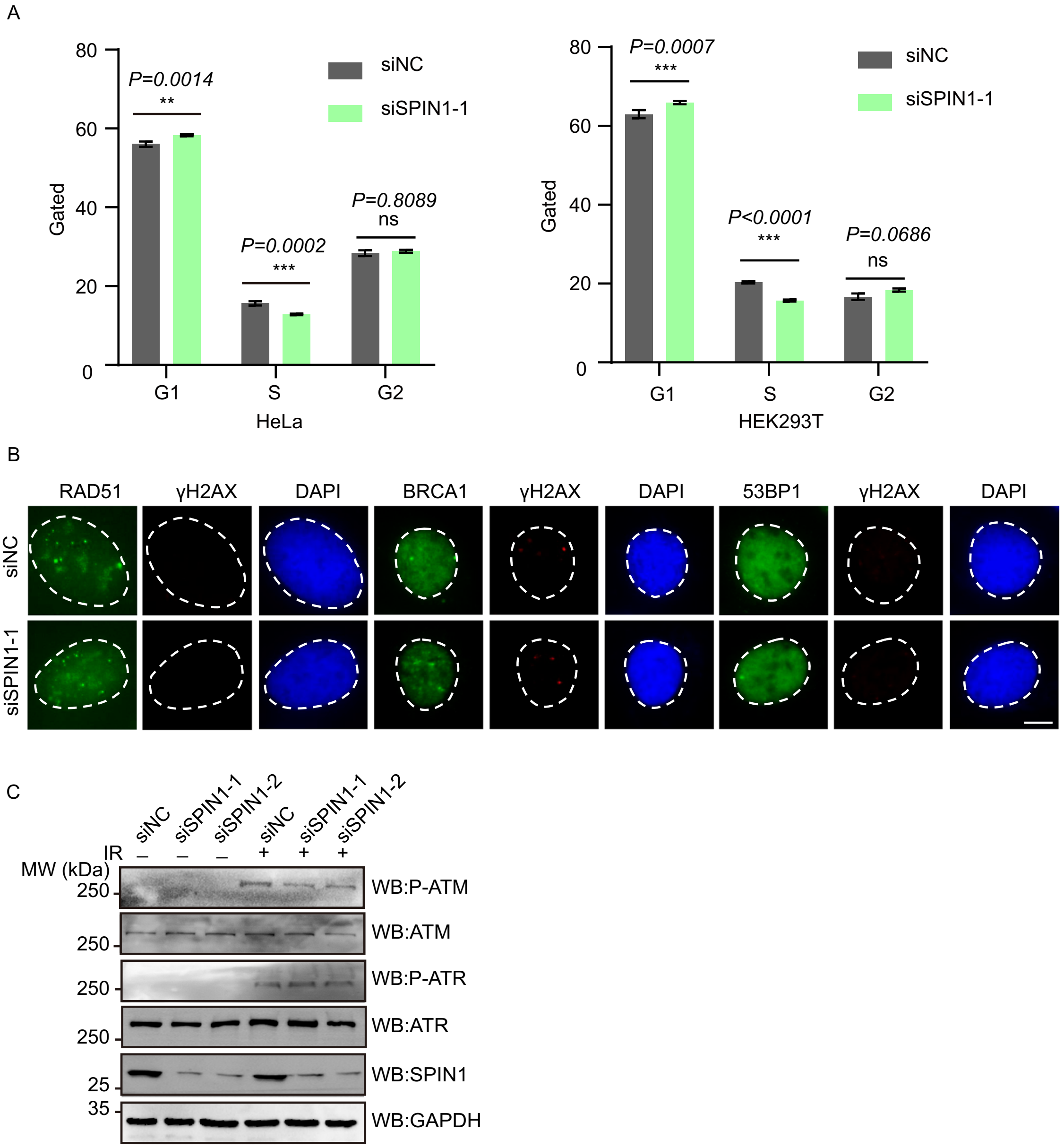

Figure EV2. (**A**) Knockdown of SPIN1 resulted in a slight G1/S shift in HeLa and HEK293T cells. HeLa and HEK293T cells were transfected with the indicated siRNAs and subsequently analyzed by flow cytometry. Three independent experiments were performed. Statistical significance was determined using one-way ANOVA followed by the Tukey Kramer test. The data are represented as the mean ± SD. $^{**}P < 0.01$; $^{***}P < 0.001$; ns, not significant. (**B**) Knockdown of SPIN1 did not induce DNA damage. U2OS cells were transfected with the indicated siRNAs, and the formation of γH2AX foci was examined by immunofluorescent staining. Scale bar = 10 µm. (**C**) Knockdown of SPIN1 resulted in a decrease in the levels of phosphorylated ATM (P-ATM), but not phosphorylated ATR (P-ATR). HEK293T cells were transfected with the indicated siRNAs and either treated or untreated with 10 Gy of IR. Total cell lysates were collected and subjected to immunoblotting using the indicated antibodies. Source data are available online for this figure.

 © The Author(s)

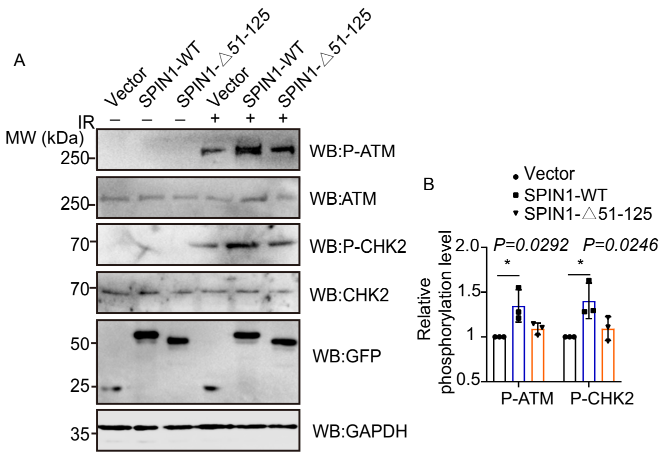

Figure EV3. (**A**) Overexpression of SPIN1-WT, but not the 51–125 amino acid deletion mutant, promoted the activation of ATM upon DNA damage. HEK293T cells expressing the vector, SFB-SPIN1-WT or SFB-SPIN1-△51–125 were treated or untreated with 10 Gy of IR. Total cell lysates were harvested and subjected to immunoblotting with the indicated antibodies. (**B**) Quantitative statistical analysis was performed on the phosphorylation levels of ATM and CHK2 from three independent biological replicates. Statistical significance was determined using the Student's t-test. Data are presented as mean ± SD. *$P < 0.05$. Source data are available online for this figure.

© The Author(s)

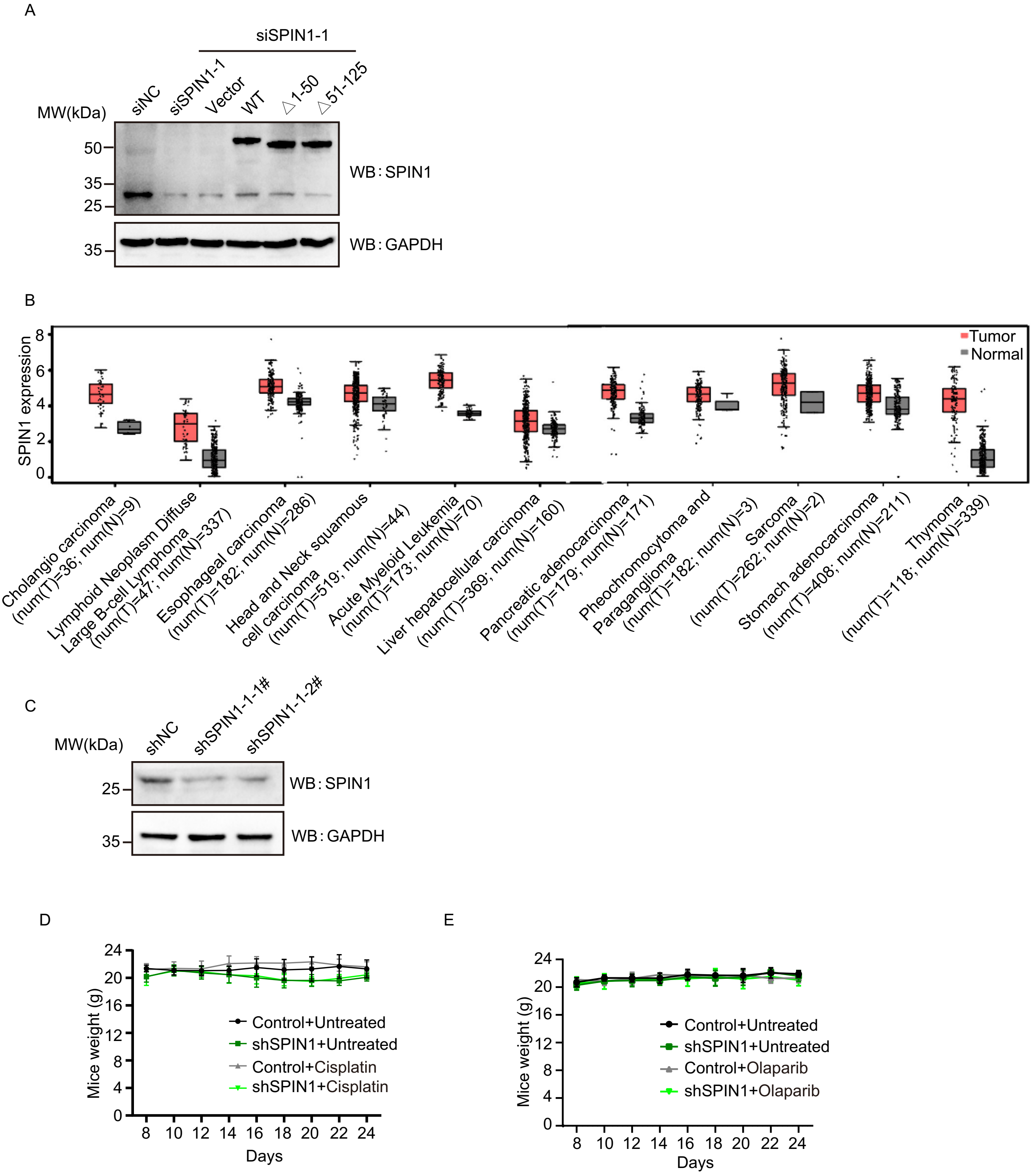

Figure EV4. (**A**) Protein expressions of GFP-tagged SPIN1 mutants were examined by Western blot analysis. The indicated mutants of GFP-tagged SPIN1 were transfected, and Western blot was performed to detect the protein expression levels. (**B**) The comparisons of SPIN1 expression between tumor and normal tissues were conducted using data from the GEPIA database(http://gepia.cancer-pku.cn/). The horizontal line within each box represents the median, and the box boundaries are defined by the 25th and 75th percentiles. The whiskers extend to the minimum and maximum values. (**C**) The protein levels of SPIN1 were analyzed by Western blot in stable shSPIN1 or shNCSGC7901 cells. (**D**, **E**) The body weight of the mice was measured during the treatment, and no significant weight loss was observed. The graphs represent the mean ± SD. *n* = 6/group. Source data are available online for this figure.

 © The Author(s)
